# Transcriptional profiles of pilocytic astrocytoma are related to their three different locations, but not to radiological tumor features

**DOI:** 10.1186/s12885-015-1810-z

**Published:** 2015-10-24

**Authors:** Krzysztof Zakrzewski, Michał Jarząb, Aleksandra Pfeifer, Małgorzata Oczko-Wojciechowska, Barbara Jarząb, Paweł P. Liberski, Magdalena Zakrzewska

**Affiliations:** 1 Department of Neurosurgery, Polish Mother Memorial Hospital Research Institute, Rzgowska 281/289, 93-338 Lodz, Poland; 2 Third Department of Radiotherapy and Chemotherapy, Maria Skłodowska-Curie Memorial Cancer Center and Institute of Oncology, Wybrzeze Armii Krajowej 15, 44-101 Gliwice, Poland; 3 Department of Nuclear Medicine and Endocrine Oncology, Maria Skłodowska-Curie Memorial Cancer Center and Institute of Oncology, Wybrzeze Armii Krajowej 15, 44-101 Gliwice, Poland; 4 Department of Molecular Pathology and Neuropathology, Medical University of Lodz, Pomorska 251, 92-213 Lodz, Poland

**Keywords:** Gene expression profiling, Location, Outcome, Pilocytic astrocytoma, Radiological appearance

## Abstract

**Background:**

Pilocytic astrocytoma is the most common type of brain tumor in the pediatric population, with a generally favorable prognosis, although recurrences or leptomeningeal dissemination are sometimes also observed. For tumors originating in the supra-or infratentorial location, a different molecular background was suggested, but plausible correlations between the transcriptional profile and radiological features and/or clinical course are still undefined. The purpose of this study was to identify gene expression profiles related to the most frequent locations of this tumor, subtypes based on various radiological features, and the clinical pattern of the disease.

**Methods:**

Eighty six children (55 males and 31 females) with histologically verified pilocytic astrocytoma were included in this study. Their age at the time of diagnosis ranged from fourteen months to seventeen years, with a mean age of seven years. There were 40 cerebellar, 23 optic tract/hypothalamic, 21 cerebral hemispheric, and two brainstem tumors. According to the radiological features presented on MRI, all cases were divided into four subtypes: cystic tumor with a non-enhancing cyst wall; cystic tumor with an enhancing cyst wall; solid tumor with central necrosis; and solid or mainly solid tumor. In 81 cases primary surgical resection was the only and curative treatment, and in five cases progression of the disease was observed. In 47 cases the analysis was done by using high density oligonucleotide microarrays (Affymetrix HG-U133 Plus 2.0) with subsequent bioinformatic analyses and confirmation of the results by independent RT-qPCR (on 39 samples).

**Results:**

Bioinformatic analyses showed that the gene expression profile of pilocytic astrocytoma is highly dependent on the tumor location. The most prominent differences were noted for *IRX2*, *PAX3*, *CXCL14*, *LHX2*, *SIX6*, *CNTN1* and *SIX1* genes expression even within different compartments of the supratentorial region. Analysis of the genes potentially associated with radiological features showed much weaker transcriptome differences. Single genes showed association with the tendency to progression.

**Conclusions:**

Here we have shown that pilocytic astrocytomas of three different locations can be precisely differentiated on the basis of their gene expression level, but their transcriptional profiles does not strongly reflect the radiological appearance of the tumor or the course of the disease.

**Electronic supplementary material:**

The online version of this article (doi:10.1186/s12885-015-1810-z) contains supplementary material, which is available to authorized users.

## Background

Pilocytic astrocytoma (PA) is the most common type of brain tumor in the pediatric population, comprising approximately 25 % of all primary tumors, with the most frequent occurrence taking place between 5–10 years of age. Fortunately this tumor has a generally good outcome, however recurrences or leptomeningeal dissemination are also sometimes observed. PAs can affect various anatomical structures, but there are three most common locations: cerebellum, optic tract with hypothalamus, and cerebral hemispheres. Those tumors are mainly sporadic, except for cases occurring in patients with neurofibromatosis type 1 and, less frequently, with Frasier and Noonan syndromes [1–3]. Molecularly, pilocytic astrocytoma is characterized by a relatively small number of chromosomal abnormalities with the most common alteration located at chromosome 7q34 comprising the *BRAF* oncogene [4, 5]. In the high-throughput analysis era, limited reports of this type of tumor were made using expression profiling. The presupposition concerning the molecular heterogeneity of pilocytic astrocytomas was defined previously by Wong et al. as the result of unsupervised hierarchical clustering. Without inference from a clinical outcome, they identified two subgroups of tumors. Such results could be a consequence of including two cases of subtotally resected tumors and, more importantly, the more aggressive variant of astrocytoma with pilomyxoid features [6]. Later, an assumption describing different expression profiles for PAs of various locations was given by Sharma et al., who showed the *LHX2* gene expression to be connected with supratentorial location [7]. A following analysis made by Tchoghandjian et al. showed upregulation of *LHX2* together with *SIX6* in tumors originated from the hypothalamo-chiasmatic region [8]. At the same time, *MATN2* and *ALDH1L1* genes were assumed to be connected with plausible PAs progression despite total surgical resection [9, 10]. Children affected by this tumor usually have a good prognosis, although in some cases recurrence or leptomeningeal dissemination may be observed [11–14]. Thus there is an ongoing need to search for molecular markers influencing the clinical behavior of this tumor. On the basis of observations made to date we verified the hypothesis that the location of pilocytic astrocytomas is the major cause of their genomic differences, and tried to find genes connected with patient outcome and tumor appearance. The aim of this study was to identify gene expression profiles related to the most frequent locations, radiological features, and the clinical course of the disease in a representative group of Polish children with PAs.

## Methods

### Patient samples

**Fig. 1:**
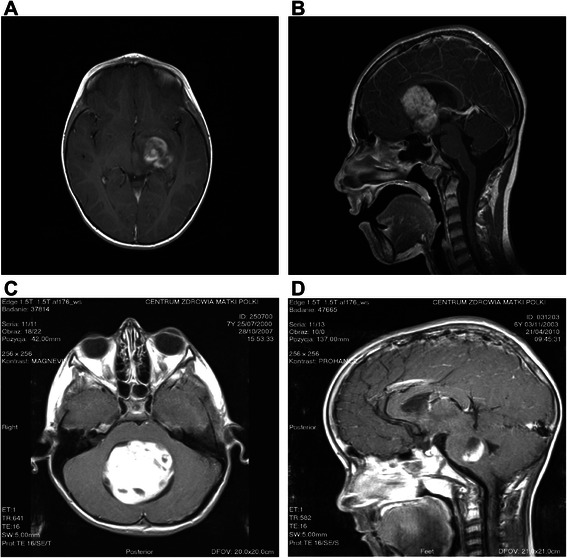
Location of pilocytic astrocytoma. **a** cerebral hemispheric tumor. **b** optic tract and hypothalamic tumor. **c** cerebellar tumor. **d** brainstem tumor. MRI scans after contrast administration

Eighty-six children with pilocytic astrocytoma who were operated on at the Department of Neurosurgery, Polish Mother’s Memorial Hospital, Research Institute in Lodz were included in this study. The group was comprised of 55 males and 31 females. The median age of patients at the time of diagnosis was 7 years (ranging from 14 months to 17 years). There were 40 cerebellar, 23 optic tract and hypothalamic, 21 cerebral hemispheric, and 2 brainstem tumors (Fig. 1). All specimens were diagnosed at the Department of Molecular Pathology and Neuropathology, Medical University of Lodz, according to the WHO criteria [1].

**Fig. 2:**
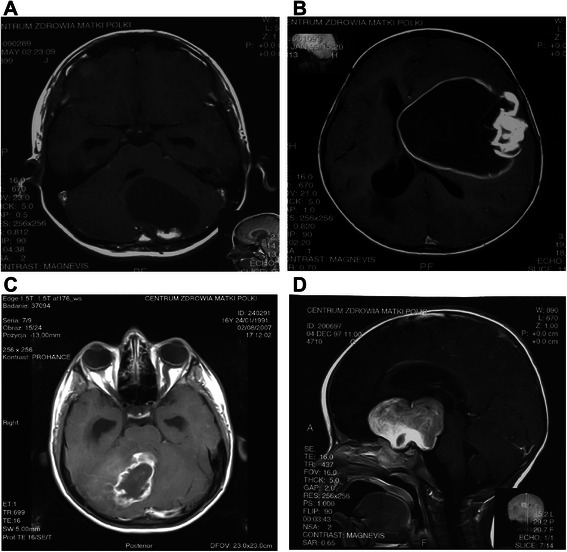
Radiological type of pilocytic astrocytoma. **a** cystic tumor with an enhancing cyst wall, R1. **b** cystic tumor with a non-enhancing cyst wall, R2. **c** solid tumor with central necrosis, R3. **d** solid or mainly solid tumor, R4. MRI scans after contrast administration

In all patients, preoperative MRI scans with and without contrast administration were obtained. For assessing the radiological features of tumors, we adopted the classification of radiological subtypes of PA proposed by Pencalet et al., commonly used in analyses of this tumor [15, 16]. According to the radiological features presented on MRI, all tumors were divided into four subtypes: cystic with a non-enhancing cyst wall, cystic with an enhancing cyst wall, solid with central necrosis, and solid or mainly solid tumors (Fig. 2).

In 81 cases primary surgical resection was the only and curative treatment, while in five cases progression of the disease, requiring additional treatment, was noted. In two cases clinical manifestation of neurofibromatosis type 1 (NF1) was observed. The clinical data of patients included in this study are presented in Table 1. All samples were collected using the protocols approved by the Bioethics Medical University Committee (Approval No. RNN/154/06/KE).

**Table 1:** Clinicopathologic features of pilocytic astrocytoma patients

Variable	Number	Percent
Gender		
Male	55	64 %
Female	31	36 %
Age		
0-9 years	53	62 %
10-17 years	33	38 %
Histopathology		
Pilocytic astrocytoma	86	100 %
Location		
Cerebellum	40	47 %
Optic tracts/Hypothalamus	23	27 %
Hemisphere	21	24 %
Brainstem	2	2 %
Radiological appearance		
Solid or mainly solid	43	50 %
Cystic/Enhanced	24	28 %
Cystic/Non enhanced	10	12 %
Largely necrotic	9	10 %
Extent of resection		
Gross total	63	73 %
Partial	23	27 %
Clinical course and current patient status		
Cured	81	94 %
Alive	80	99 %
Dead	1	1 %
Progressive	5	6 %
Alive	4	99 %
Dead	1	1 %
Genetic conditions		
NF1 excluded	84	98 %
NF1 confirmed	2	2 %
Total	86	100 %

Written informed parental consent was obtained from all patients under 16 (75 patients). In eleven older patients the participants gave their own consent according to the Polish law. All data were processed and stored in compliance with the Helsinki Declaration.

### RNA isolation

Total RNA was extracted from the sna*p-*frozen tumor tissues stored at–80 °C after excision, using the acid phenol-guanidinum extraction method, purified using commercially available sets (RNeasy Mini Kit, Qiagen) and treated with DNAase (Qiagen) [17]. In order to obtain a high amount of RNA, macrodissection was used in all cases. Specimens were visually assessed by the pathologist to confirm that at least 50 % of tumor cells within the sample and areas with highest content of neoplastic tissue were used for direct RNA extraction. The quantity of RNA was measured using the NanoDrop 1000 (Thermo Scientific). RNA samples’ quality was analysed using 2000 Bioanalyzer (Agilent Technologies), and after capillary electrophoresis the RNA integrity number (RIN) was generated by the software for each specimen.

### cRNA synthesis and hybridization

250 ng RNA of each sample selected for array analysis (50 cases) was used for cDNA and subsequent cRNA synthesis (GeneChip® 3′ IVT Expression Kit, Affymetrix). The amplified and biotinylated complementary RNA (cRNA) was purified and fragmented using heat and Mg^2+^, and then underwent hybridization (45 °C, 16 hours) with GeneChip Human Genome U133 Plus 2.0 Array (Affymetrix), followed by array staining (Streptavidin, Alexa Fluor 610-R-phycoerythrin conjugate, Molecular Probes). All procedures were performed according to the manufacturer’s instructions (Affymetrix). Arrays were scanned using the GeneChip Scanner 3000 (Affymetrix).

### Microarray analysis

Quality control of microarray data was carried out according to standard protocols, based on R/Bioconductor packages (ver. 2.3.5). Data were pre-processed using the GC-Robust Multi-array Average (GC-RMA) procedure, Normalized Unscaled Standard Error (NUSE) and Relative Log Expression (RLE) measures were calculated to verify the technical homogeneity of the dataset. On the basis of quality control, 47 out of 50 microarrays were then classified to the bioinformatic analyses.

Transcripts showing minimal variation of expression across the set of arrays were excluded from the analysis. Genes with expression differed by at least 1.5 times from the median in at least 10 % of the arrays, with variance significantly larger than the median variance (*p* ≤ 0.01) retained. For the selection of genes’ differentiating subgroups, the Welch t-test with false discovery rate (FDR) estimation was used. A global test was applied to test whether the expression profiles differed between the classes by permuting the labels of which arrays corresponded to which classes. Biological relevance and contribution in cellular processes of obtained sets was analyzed by Gene Ontology classification on the basis of the Gene Ontology Consortium database (http://www.geneontology.org). For selected genes the gene set enrichment analysis, with curated and motif gene set collections, was performed to analyze the signaling pathways (Molecular Signatures Database v. 3.0, http://www.broadinstitute.org/gsea/msigdb/index.jsp). These analyses were performed using Kolmogorov-Smirnov, the Least Squares test, and the Gene Set Analysis method (*p* ≤ 0.001). Statistical analysis was carried out by BRB-Array Tools (ver. 4.1.0, http://linus.nci.nih.gov/BRB-ArrayTools.html, developed by Dr R. Simon and BRB-Array Tools Development Team) and R/Bioconductor packages (http://www.bioconductor.org).

### Validation of the microarray data

Correlation analysis of RT-qPCR and microarray expression values were carried out for 39 independent samples equally diversified according to the three tumor locations: cerebral hemispheric tumors (M1), optic tract and hypothalamic tumors (M2), cerebellar tumors (M3). TaqMan® Gene Expression Assays by TaqMan® real time PCR with TaqMan® Universal PCR Master Mix (Applied Biosystems, UK) was used following the manufacturer’s instructions on a Rotor Gene 6000 instrument (Qiagene-Corbett Life Science, Sydney, Australia) for selected genes (Additional file 1: Table S1). The PCR reactions for each assay were run in triplicate and the results were averaged. The normalized relative expression level of the genes of interest was calculated according to the method described by Pfaffl and Vandesompele et al., with *GAPDH* used as a reference gene [18, 19]. Statistical comparison of three subgroups was made on the basis of the Kruskal*-*Wallis nonparametric test, with *post hoc* pairwise comparisons using the Dwass-Steel-Critchlow-Fligner test. Statistical significance was assumed for *p* ≤ 0.05.

## Results

We performed bioinformatic analysis of the global gene expression of 47 childhood pilocytic astrocytoma with respect to the selected clinical features. After pre-processing of the data 21,910 probesets showed significant variance and were further analysed. For the purposes of bioinformatic analysis, all analyzed samples were divided, on the basis of pivotal clinical data, into eight subgroups: cerebral hemispheric tumors (M1); optic tract and hypothalamic tumors (M2); cystic cerebellar tumors with an non-enhanced cyst (M3R1); cystic cerebellar tumors with an enhanced cyst (M3R2); solid cerebellar tumors with central necrosis (M3R3); solid or mainly solid cerebellar tumors (M3R4); tumors linked to the neurofibromatosis type 1 (NF1); and progressive tumors (P2).

During the comparison of these eight subgroups using the parametric Welch t-test and *post hoc* class comparison test, we found 345 probesets with significantly changed expression (*p* < 0.001). The observed differences were also strongly significant in the global test (*p* < 0.007) (Additional file 2: Table S2).

The evaluation of biological processes represented within the selected genes was done on the basis of the gene ontology over-representation analysis. The most significantly represented ontology classes were connected with neuronal cells building proteins, adhesion molecules, cell junctions, and hormone and neuropeptides activity (Table 2). Within genes with significantly changed expression, there were some that connected with transcriptional processes and acting during embryogenesis and central nervous system differentiation.

**Table 2:** Gene ontology (GO) analysis of genes selected from transcripts differentiating the clinical subgroups of pilocytic astrocytomas

GO ID	GO Term	Observed in the selected subset	Expected in the selected subset	Observed/Expected
Cellular components				
GO:0031225	Anchored to membrane	6	1.66	3.62
GO:0005925	Focal adhesion	6	1.72	3.48
GO:0009897	External side of plasma membrane	6	1.77	3.4
GO:0005924	Cell-substrate adherens junction	6	1.77	3.4
GO:0031253	Cell projection membrane	5	1.52	3.28
GO:0005912	Adherens junction	9	2.83	3.19
GO:0030055	Cell-substrate junction	6	1.92	3.12
GO:0043235	Receptor complex	5	1.74	2.87
GO:0044297	Cell body	6	2.27	2.64
GO:0043025	Neuronal cell body	6	2.27	2.64
Molecular functions				
GO:0019842	Vitamin binding	6	1.84	3.26
GO:0019900	Kinase binding	9	2.9	3.1
GO:0019901	Protein kinase binding	7	2.3	3.05
GO:0003714	Transcription corepressor activity	6	2.25	2.66
GO:0008234	Cysteine-type peptidase activity	6	2.34	2.56
GO:0042277	Peptide binding	5	2.3	2.18
Biological processes				
GO:0032677	Regulation of interleukin-8 production	6	0.37	16.38
GO:0032637	Interleukin-8 production	6	0.4	15.12
GO:0003081	Regulation of systemic arterial blood pressure by renin-angiotensin	5	0.34	14.89
GO:0001990	Regulation of systemic arterial blood pressure by hormone	5	0.43	11.7
GO:0002221	Pattern recognition receptor signaling pathway	7	0.82	8.49
GO:0003044	Regulation of systemic arterial blood pressure mediated by a chemical signal	5	0.61	8.19
GO:0002758	Innate immune response-activating signal transduction	7	0.89	7.91
GO:0002218	Activation of innate immune response	7	0.89	7.91
GO:0003073	Regulation of systemic arterial blood pressure	5	0.64	7.8
GO:0050886	Endocrine process	5	0.73	6.83
GO:0021953	Central nervous system neuron differentiation	5	0.76	6.55
GO:0050731	Positive regulation of peptidyl-tyrosine phosphorylation	6	0.95	6.34
GO:0050729	Positive regulation of inflammatory response	5	0.82	6.07
GO:0002768	Immune response-regulating cell surface receptor signaling pathway	7	1.19	5.88
GO:0050864	Regulation of B cell activation	6	1.07	5.62
GO:0050671	Positive regulation of lymphocyte proliferation	6	1.07	5.62
GO:0070665	Positive regulation of leukocyte proliferation	6	1.1	5.46
GO:0048839	Inner ear development	8	1.47	5.46
GO:0032946	Positive regulation of mononuclear cell proliferation	6	1.1	5.46
GO:0002429	Immune response-activating cell surface receptor signaling pathway	6	1.13	5.31
GO:0050730	Regulation of peptidyl-tyrosine phosphorylation	7	1.47	4.78
GO:0050670	Regulation of lymphocyte proliferation	8	1.71	4.68
GO:0070663	Regulation of leukocyte proliferation	8	1.74	4.6
GO:0032944	Regulation of mononuclear cell proliferation	8	1.74	4.6
GO:0042129	Regulation of T cell proliferation	5	1.13	4.43
GO:0032103	Positive regulation of response to external stimulus	7	1.62	4.33
GO:0002706	Regulation of lymphocyte mediated immunity	5	1.19	4.2
GO:0050727	Regulation of inflammatory response	6	1.47	4.1
GO:0043583	Ear development	8	1.98	4.03
GO:0009310	Amine catabolic process	8	1.98	4.03
GO:0090047	Positive regulation of transcription regulator activity	5	1.31	3.81
GO:0051091	Positive regulation of transcription factor activity	5	1.31	3.81
GO:0008217	Pegulation of blood pressure	7	1.86	3.76
GO:0051606	Detection of stimulus	6	1.65	3.64
GO:0021537	Telencephalon development	8	2.2	3.64
GO:0009064	Glutamine family amino acid metabolic process	5	1.37	3.64
GO:0002822	Regulation of adaptive immune response based on somatic recombination of immune receptors built from immunoglobulin superfamily domains	5	1.37	3.64
GO:0002703	Regulation of leukocyte mediated immunity	5	1.37	3.64
GO:0050870	Positive regulation of T cell activation	7	1.95	3.58
GO:0002819	Regulation of adaptive immune response	5	1.4	3.56
GO:0043388	Positive regulation of DNA binding	5	1.43	3.49
GO:0051099	Positive regulation of binding	6	1.74	3.45
GO:0009063	Cellular amino acid catabolic process	6	1.74	3.45
GO:0043410	Positive regulation of MAPKKK cascade	5	1.5	3.34
GO:0090046	Regulation of transcription regulator activity	8	2.47	3.24
GO:0051090	Regulation of transcription factor activity	8	2.47	3.24
GO:0048562	Embryonic organ morphogenesis	8	2.53	3.16
GO:0046651	Lymphocyte proliferation	8	2.53	3.16
GO:0042113	B cell activation	8	2.53	3.16
GO:0018108	Peptidyl-tyrosine phosphorylation	8	2.53	3.16
GO:0045927	Positive regulation of growth	5	1.62	3.09
GO:0018212	Peptidyl-tyrosine modification	8	2.59	3.08
GO:0070661	Leukocyte proliferation	8	2.62	3.05
GO:0032943	Mononuclear cell proliferation	8	2.62	3.05
GO:0007187	G-protein signaling, coupled to cyclic nucleotide second messenger	5	1.65	3.03
GO:0002237	Response to molecule of bacterial origin	6	1.98	3.02
GO:0046395	Carboxylic acid catabolic process	8	2.66	3.01
GO:0016054	Organic acid catabolic process	8	2.66	3.01
GO:0042098	T cell proliferation	5	1.68	2.98
GO:0042445	Hormone metabolic process	7	2.38	2.94
GO:0019935	Cyclic-nucleotide-mediated signaling	6	2.04	2.93
GO:0009952	Anterior/posterior pattern formation	6	2.08	2.89
GO:0010001	Glial cell differentiation	5	1.77	2.82
GO:0009266	Response to temperature stimulus	5	1.77	2.82
GO:0071375	Cellular response to peptide hormone stimulus	6	2.14	2.81
GO:0030217	T cell differentiation	9	3.27	2.76
GO:0045664	Regulation of neuron differentiation	9	3.33	2.71
GO:0050863	Regulation of T cell activation	8	2.99	2.67
GO:0001934	Positive regulation of protein amino acid phosphorylation	6	2.29	2.62
GO:0051222	Positive regulation of protein transport	5	1.95	2.56
GO:0002460	Adaptive immune response based on somatic recombination of immune receptors built from immunoglobulin superfamily domains	7	2.87	2.44
GO:0043408	Regulation of MAPKKK cascade	7	2.9	2.41
GO:0042327	Positive regulation of phosphorylation	6	2.5	2.4
GO:0034097	Response to cytokine stimulus	6	2.5	2.4
GO:0002250	Adaptive immune response	7	2.93	2.39
GO:0002449	Lymphocyte mediated immunity	6	2.53	2.37
GO:0050953	Sensory perception of light stimulus	7	3.02	2.32
GO:0007601	Visual perception	7	3.02	2.32
GO:0045937	Positive regulation of phosphate metabolic process	6	2.59	2.31
GO:0042063	Gliogenesis	5	2.17	2.31
GO:0010562	Positive regulation of phosphorus metabolic process	6	2.59	2.31
GO:0007411	Axon guidance	5	2.17	2.31
GO:0043010	Camera-type eye development	5	2.2	2.28
GO:0009617	Response to bacterium	8	3.51	2.28
GO:0002697	Regulation of immune effector process	5	2.2	2.28
GO:0032870	Cellular response to hormone stimulus	8	3.54	2.26
GO:0071495	Cellular response to endogenous stimulus	8	3.57	2.24
GO:0030855	Epithelial cell differentiation	5	2.23	2.24
GO:0030258	Lipid modification	5	2.23	2.24
GO:0043122	Regulation of I-kappaB kinase/NF-kappaB cascade	6	2.78	2.16
GO:0008643	Carbohydrate transport	5	2.32	2.16
GO:0048568	Embryonic organ development	8	3.72	2.15
GO:0051239	Regulation of multicellular organismal process	72	33.82	2.13
GO:0008624	Induction of apoptosis by extracellular signals	5	2.38	2.1
GO:0007399	Nervous system development	84	41.36	2.03
GO:0002376	Immune system process	80	39.37	2.03
GO:0003002	Regionalization	8	3.97	2.02

The number of genes changed in each category was compared with the number of expected occurrences. Only GO classes and parent classes with at least five observations in the selected subset and with an ’observed vs. expected’ ratio of at least two were shown

The analysis of selected genes’ contribution in the signaling pathways revealed changed regulation of 77 within 2131 curated gene sets, and 14 within 179 motif gene sets. The highest statistical significance was obtained for genes functionally connected with immune response pathways, pathways engaged in silencing suppressors during histone methylation and activation of the NFkB pathway. Interesting group consisted of targets for miR324-5p, miR432, miR299-3P, miR486 and miR499 and genes located near promoter regions of *NR6A1*, *POU3F2*, *CUTL1*, *PAX8* and *AHR* transcription factors (Table 3).

**Table 3:** Selected gene sets differentiated between pilocytic astrocytomas of variable clinical features

Broad GeneSets	Number of genes	LS	KS	GSA
		*p-*value	*p-*value	*p-*value
KONDO_PROSTATE_CANCER_HCP_WITH_H3K27ME3	100	0.00001	0.00001	<0.005
LI_CISPLATIN_RESISTANCE_UP	66	0.00001	0.00019	<0.005
REACTOME_ACTIVATED_TLR4_SIGNALLING	25	0.00001	0.00001	<0.005
REACTOME_TOLL_LIKE_RECEPTOR_4_CASCADE	28	0.00001	0.00001	<0.005
SHARMA_PILOCYTIC_ASTROCYTOMA_LOCATION_DN	7	0.00001	0.00001	<0.005
SHARMA_PILOCYTIC_ASTROCYTOMA_LOCATION_UP	31	0.00001	0.00001	<0.005
WATANABE_COLON_CANCER_MSI_VS_MSS_DN	96	0.00001	0.00041	<0.005
ZHAN_MULTIPLE_MYELOMA_PR_DN	69	0.00001	0.00001	<0.005
BIOCARTA_DC_PATHWAY	13	0.00023	0.00079	<0.005
CGGTGTG, MIR-220	8	0.00001	0.09215	<0.005
V$GNCF_01	93	0.00002	0.00239	0.04
GGGATGC, MIR-324-5P	72	0.00003	0.01085	<0.005
CCATCCA, MIR-432	64	0.00006	0.00044	0.015
V$POU3F2_01	94	0.00006	0.02333	0.025
CCANNAGRKGGC_UNKNOWN	89	0.00017	0.00001	0.035
V$CDPCR3_01	39	0.00028	0.02874	0.065
CCCACAT,MIR-299-3P	72	0.00034	0.08243	0.07
V$MYOGNF1_01	57	0.00045	0.01553	0.045
CTGYNNCTYTAA_UNKNOWN	73	0.00055	0.00747	0.09
GTACAGG, MIR-486	72	0.00056	0.00176	0.01
V$PAX8_01	49	0.00058	0.0018	<0.005
V$AHR_01	86	0.00068	0.05371	0.16
CCAATNNSNNNGCG_UNKNOWN	65	0.00407	0.22226	<0.005
AGTCTTA, MIR-499	99	0.02398	0.00033	0.22
V$MEF2_04	24	0.03997	0.01638	<0.005

Three independent tests: LS, KS permutation test, and Efron-Tibshirani’s GSA maxmean test were applied to select significantly affected gene classes

**Fig. 3:**
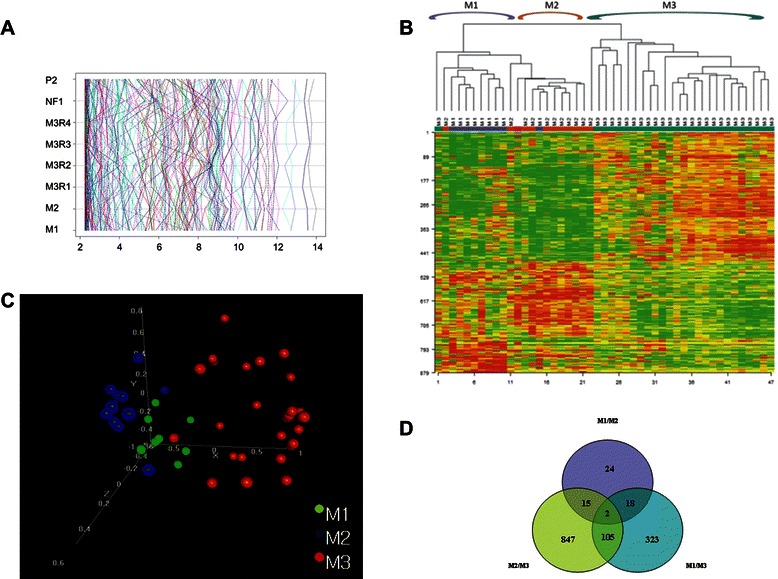
Results of analysis of correlation between gene expression profile and location of childhood pilocytic astrocytomas. **a** results of probesets expression according to clinical subgroup. **b** unsupervised hierarchical clustering demonstrated that PA from the three main locations have a unique transcriptome profile. **c** anatomical location-related subgroups are clearly distinct in fully unsupervised multidimensional scaling analysis. **d** Venn diagram showing numbers of location-related probesets. M1, cerebral hemispheric tumor; M2, optic tract and hypothalamic tumor; M3, cerebellar tumor; M3R1, cystic cerebellar tumor with an non-enhanced cyst; M3R2, cystic cerebellar tumor with enhanced cyst; M3R3, solid cerebellar tumor with central necrosis; M3R4, solid or mainly solid cerebellar tumor; NF1, tumor linked to the neurofibromatosis type 1; P2, progressive tumor

In the next step we analyzed the expression values of genes differentiating clinical subgroups of PA. Genes with highest amplitude were chosen for hierarchical clustering of samples (Fig. 3a). On the basis of such analysis we obtained three distinct clusters showing almost perfect classification of samples, which revealed that the main source of variability is related to the location of the tumors. The cerebellar tumors consist of a homogenic cluster, while the supratentorial samples showed single outlier specimens (Fig. 3b). Within tumors of optic tract and hypothalamus there was also a sample of brain stem PA, which presented a low correlation of gene expression with the supratentorial cases (*r* = 0,4). This sample was excluded from statistical analyses because of its low RNA quality, and as a consequence both cases of PA located within the brain stem were used only during data visualization.

**Fig. 4:**
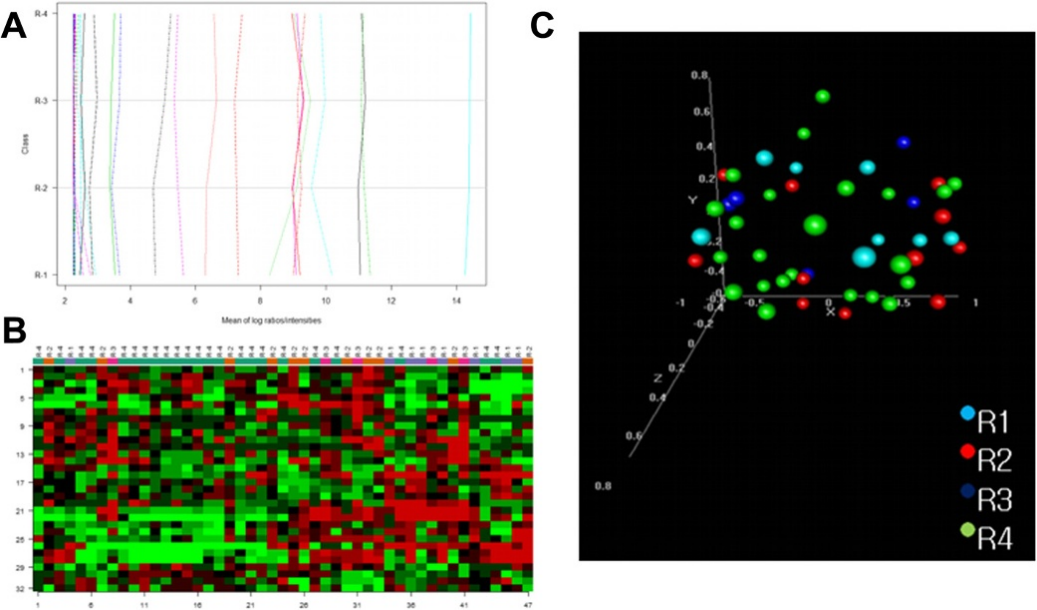
Results of association of radiological features with global gene expression profile. **a** probesets expression showing a relatively small amplitude of differences radiological subgroups. **b** unsupervised hierarchical clustering did not show specific gene expression signature correlated with radiological features (32 probesets, FDR adjusted *p* < 0.005). **c** radiological-related subgroups are not distinct in fully unsupervised multidimensional scaling analysis. R1, cystic tumor without an enhanced cyst wall; R2, cystic tumor with enhanced cyst; R3, solid tumor with central necrosis; R4, solid or mainly solid tumor

Bioinformatic analysis of our dataset revealed that 32 probesets showed different expression pattern according to radiological subclasses (*p* < 0.005). Unfortunately these genes demonstrated weak transcriptome differences (Fig. 4a), with borderline significance in the global test of association (p = 0,88). Hierarchical clustering and PCA analyses taking into account the radiological features of tumors did not show a specific gene expression signature correlated with the radiological features of analyzed PA (Fig. 4b, c).

Here we verified the hypothesis that the location of PAs is the major cause of their genomic differences. Analyses of three main anatomical subclasses (M1, M2, M3) using the parametric Welch t-test were very prominent and revealed statistically significant differences for 862 probesets based on the false discovery rate (FDR adjusted *p-*value of 0.001).

In the global test the differences were also strongly significant (*p* < 0.001). The comparisons of pairs (M1vsM3; M2vsM3; M1vsM2) using the *post hoc* test (BRB ArrayTools) revealed that the majority of genes showed different expression for the M2vsM3 and M1vsM3 (847 and 323 genes respectively), while 105 genes showed differences both for M1vsM3 and M2vsM3 tumors. The most similarities (24 strongly differentiating genes) were noted for two analyzed supratentorial subgroups (Table 4, Fig. 3d, Additional file 3: Table S3 and Additional file 4: Table S4). These comparisons were also repeated with more restricted statistical criteria using the Benjamini-Hochberg multiple comparisons correction, with the criterion of FDR < 1 %. After comparison of the M3 and combined M1 and M2 subgroups, a list of 348 probesets was obtained. The probability of proper classification of tumors on the basis of gene expression profile reached a range of 80 % accuracy.

**Table 4:** List of genes differentiated between pilocytic astrocytomas of three different locations

Probe set	Gene symbol	Description	Parametric *p-*value	FDR	Geom mean of intensities in class M1	Geom mean of intensities in class M2	Geom mean of intensities in class M3	Pairwise significant
228462_at	IRX2	Iroquois homeobox 2	<1e-07	<1e-07	7.81	36.27	470.74	(1, 2), (1, 3), (2, 3)
231666_at	PAX3	Paired box 3	<1e-07	<1e-07	4.97	7.59	279.28	(1, 3), (2, 3)
207250_at	SIX6	SIX homeobox 6	<1e-07	<1e-07	7.32	246.4	4.75	(1, 2), (3, 2)
206140_at	LHX2	LIM homeobox 2	<1e-07	<1e-07	1680.77	2642.73	11.02	(3, 1), (3, 2)
223582_at	GPR98	G protein-coupled receptor 98	<1e-07	<1e-07	50.94	146.32	15.08	(1, 2), (3, 1), (3, 2)
230720_at	RNF182	Ring finger protein 182	<1e-07	<1e-07	171.48	1797.68	2061.29	(1, 2), (1, 3)
210239_at	IRX5	Iroquois homeobox 5	<1e-07	<1e-07	7.84	25.89	100.36	(1, 2), (1, 3), (2, 3)
213285_at	TMEM30B	Transmembrane protein 30B	<1e-07	<1e-07	7.23	26.44	5.08	(1, 2), (3, 2)
230472_at	IRX1	Iroquois homeobox 1	<1e-07	<1e-07	10.8	66.79	205.19	(1, 2), (1, 3), (2, 3)
227202_at	CNTN1	Contactin 1	<1e-07	<1e-07	262.72	51.46	1717.72	(2, 1), (1, 3), (2, 3)
214954_at	SUSD5	Sushi domain containing 5	<1e-07	<1e-07	125.14	1108.04	1603.52	(1, 2), (1, 3)
206018_at	FOXG1	Forkhead box G1	<1e-07	<1e-07	1197.42	11.25	4.93	(2, 1), (3, 1)
208221_s_at	SLIT1	Slit homolog 1 (Drosophila)	<1e-07	<1e-07	8.42	17.8	6	(1, 2), (3, 2)
238021_s_at	CRNDE	Colorectal neoplasia differentially expressed (non-protein coding)	<1e-07	<1e-07	51.09	382.35	1216.52	(1, 2), (1, 3), (2, 3)
226448_at	FAM89A	Family with sequence similarity 89, member A	<1e-07	<1e-07	104	625.78	808.9	(1, 2), (1, 3)
229831_at	CNTN3	Contactin 3 (plasmacytoma associated)	<1e-07	<1e-07	21.57	9.97	176.8	(1, 3), (2, 3)
228347_at	SIX1	SIX homeobox 1	<1e-07	<1e-07	13.8	421.71	9.17	(1, 2), (3, 2)
206634_at	SIX3	SIX homeobox 3	<1e-07	<1e-07	4.86	49.47	4.75	(1, 2), (3, 2)
238022_at	CRNDE	Colorectal neoplasia differentially expressed (non-protein coding)	<1e-07	<1e-07	14	51.83	134.25	(1, 2), (1, 3), (2, 3)
238878_at	ARX	Aristaless related homeobox	<1e-07	<1e-07	102.99	6.88	4.85	(2, 1), (3, 1)
227614_at	HKDC1	Hexokinase domain containing 1	<1e-07	<1e-07	30.54	9.56	8.08	(2, 1), (3, 1)
205858_at	NGFR	Nerve growth factor receptor (TNFR superfamily, member 16)	<1e-07	<1e-07	135.44	53.07	14.67	(3, 1), (3, 2)

M1-cerebral hemispheric tumor; M2-optic tract and hypothalamic tumor; M3-cerebellar tumor

In order to exclude the potential influence of other clinical variables on the obtained results, additional analysis was also performed for infratentorial cases, which included all four radiological types of PA. This approach confirmed our observations.

**Fig. 5:**
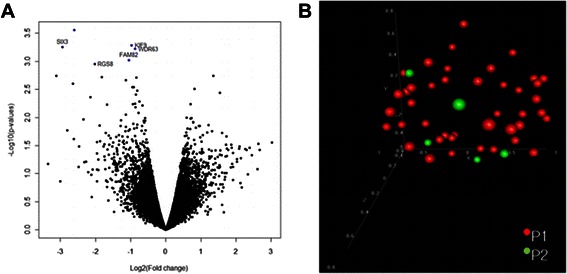
Progression of the disease was not associated with global gene expression profile. **a** only five genes were down regulated in group of tumors without progression (*p* < 0.005). **b** clinical course of the disease was not distinct in fully unsupervised multidimensional scaling analysis. P1, favorable outcome; P2, progressive disease

Our analyses of the transcriptome profile of five cases with progressive disease did not show any correlation with a worse outcome. Only five genes (*SIX3, RGS8, FAM82, KIF9, WDR63*) reached statistical significance (*p* = 0.001) when the univariate model was used, but the global test revealed that this association did not meet the criteria of statistical significance (*p* = 0.83) (Fig. 5a). Cases with neurofibromatosis type 1 had no connection with expression profile.

In the final stage of analysis we applied an unsupervised method (Principal Component Analysis, PCA) to determine the sources of variability in our group of samples according to the clinical data, and that analysis also indicated that the gene expression profile of pilocyticastrocytomas highly depends on the tumor’s location (*p* = 0.001), but not on other clinical features (Figs. 3c, 4c and 5b).

As the analysis of the most important determinants of gene expression (including location and radiological appearance) include the potential for multivariate associations, we carried out a two-way analysis of variance. In this multivariate approach two variables were taken into account (location: supratentorial vs infratentorial, i.e. M1 + M2 vs M3 + M4, and radiology: cystic vs solid, i.e. R1 + R2 vs R3 + R4). In the multivariate analysis only location seemed to be associated with gene expression (702 probesets with non-corrected *p-*value < 0.001, 1007 probesets with FDR < 10 %), while radiological appearance was almost without influence on gene expression (5 probesets with non-corrected *p* < 0.001, no probesets with FDR below 10 %).

**Fig. 6:**
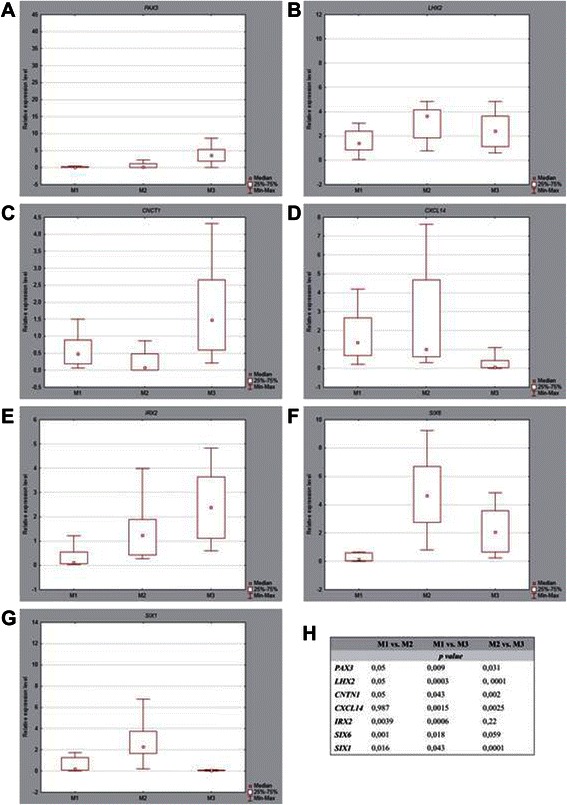
Results of microarray data validation performed by independent RT-qPCR on 39 samples, equally diversified according to the three main locations. **a-g** values of *PAX3*, *LHX2*, *CNCT1*, *CXCL14*, *IRX2*, *SIX6*, *SIX1* genes expression in pilocytic astrocytoma of different locations, presented as medians with respective inter-quartile ranges. **h** *p-*values obtained for site-specific subgroups compared using the Kruskal-Wallis test with *post hoc* pairwise comparisons using Dwass-Steel-Critchlow-Fligner test. Statistical significance was assumed as *p* ≤ 0.05. M1, cerebral hemispheric tumor (13 cases); M2, optic tract and hypothalamic tumor (13 cases); M3, cerebellar tumor (13 cases)

During our analysis the most prominent differences connected with the location of the tumor were noted for the *IRX2*, *PAX3*, *CXCL14*, *LHX2, SIX6*, *CNTN1* and *SIX1* genes. For all these genes we performed validation results by independent RT-qPCR on 39 cases of PA, which confirmed the data obtained during microarray analysis with similar expression differences. For the *PAX3, LHX2, CNCT1* and *CXCL14* genes we obtained results which converged with the microarray results and these discriminants were the best to differentiate M1 and M2 from M3 tumors. For *PAX3*, *LHX2*, *IRX2* and *CNTN1* genes similar as in microarray analysis, the major expression differences for M1/M2 and M3 tumors was showen. The gene with a statistically significant level of expression for all three subgroups of tumors was *SIX1* (Fig. 6).

The data discussed in this publication have been deposited in the NCBI’s Gene Expression Omnibus and are accessible through the GEO Series accession number GSE73066 (http://www.ncbi.nlm.nih.gov/geo/query/acc.cgi?acc=GSE73066) [20].

## Discussion

In the current study we performed expression analysis of PAs diversified according to the main clinical discriminants, from which the tumor location was the most important. We also tried to verify if and how the transcriptional profile is connected with the radiological appearance of the tumor. Moreover we attempted to identify the expression profiles related to the clinical course of the disease.

Unsupervised clustering analysis revealed differences between PAs located in three anatomical regions, which was also well confirmed by decomposition into principal components (Fig. 3b, c). The specific transcriptional profile for hemispheric tumors was mainly characterized by upregulation of *FOXG1*, *NEDD4L*, *L1CAM* and *CXCL14* genes. The *FOXG1* and *NEDD4L* have a functional connection with the TGF-beta signaling pathway which is involved in cells’ proliferation and differentiation irregularities noted in other brain tumors, including gliomas, medulloblastomas and supratentorial ependymomas [21–23]. For the following *L1CAM*, acting during brain development and having an unknowable role in the adult nervous system, the correlation with tumor grade in several other solid human cancers was noted [22, 24]. In turn, expression of *CXCL14* chemokine with enigmatic physiological function is connected with the tendency of tumor infiltration described on the basis of in vitro studies [25, 26]. Taken together, these genes are closely related not only to brain development but also to brain tumor growth and expansion. In this subgroup we also found the moderate overexpression of the *LHX2* gene. Alterations of *LHX2* expression were previously found in all supratentorial or hypothalamo-chiasmatic region tumors [8, 9]. Here we confirmed significantly higher levels of gene expression for PAs located within the hypothalamo-chiasmatic region. The validation of array data reached statistical significance and allowed us to distinguish these two subgroups of supratentorial tumors on the basis of *LHX2* activity. The *LHX2* gene has a known function in brain development during embryogenesis among different organisms. Previous functional studies on model organisms showed the relationship between *lhx2* and two *six3* genes in forebrain development. Recent analyses of human embryonic stem cells demonstrated that it also has a crucial role in human brain morphogenesis [27, 28]. There is evidence that *LHX2* is expressed in the early neural lineage, which affects signaling pathways fundamental for early lineage differentiation in the human brain via regulation of the neural transcription factors from the SIX gene family [28, 29]. Our results constitute confirmation of this observation. The unique transcriptional profile of our hypothalamo-chiasmatic region tumors was characterized by the overrepresentation of several SIX homeobox transcription factors, *SIX1*, *SIX3* and *SIX6*.

The upregulation of other homeobox transcription factors was also observed by us in cases of infratentorial tumor, which was shown to be connected with Iroquois homeobox transcription factors (*IRX1*, *IRX2*, *IRX3*, *IRX5*) and the *PAX3* gene. A similar trend was noted by Sharma et al. [9]. These genes play a crucial role in early brain regionalization, including patterning of the anterior-posterior and dorso-ventral axis, as well as the differentiation of its specific regions. On model organisms its homologs overexpression induces the occurrence of ectopic neural tissue and causes inhibition of neuronal differentiation [30, 31].

Contactin family genes (*CNTN1*, *CNTN3*), which function as cell adhesion molecules with an essential role in later stages of cerebellar morphogenesis and differentiation, were also significantly overrepresentated in our cohort of cerebellar tumors [32, 33]. Moreover, recent experimental analysis of developing mice cerebellum suggests the cooperation of CNTN proteins in modulating SHH-induced neuronal precursor proliferation and its connection with foliation of the cerebellum [32]. The deregulation of the SHH signaling pathway in cerebellar granule neuron precursors is one of the crucial causes of the SHH subgroup of medulloblastoma, but recent observations also associate this signaling pathway with other central nervous system tumors, including gliomas [34]. This is the first report where *CNTN1* and *CNTN3* gene expression were shown to be connected with cerebellar pilocytic astrocytomas biology. Also our following observation concerning misregulation of miR-324-5p, engaged in the tumorigenesis of various cancers including glial tumors, suggests SHH contribution in PA biology as probably the consequence of changed progenitor differentiation [35–37].

Another interesting finding concerns the *RUNX1T1* transcript upregulation observed *in silico* study performed for pilocytic astrocytomas by Deshmukh et al. On the basis of the transcriptional regulatory network, they indicated that *RUNX1T1*, together with four other transcription factors, is within alteration common for pilocytic astrocytomas and glioblastomas [38]. This is not the only relationship between those histologically different glial tumors. Our group of cerebellar PAs was also characterized by elevated levels of *PROM1* expression, which in glioblastoma is considered as a molecular factor of poor prognosis, and in proneural glioblastoma seems to be the potential target of anti-angiogenic therapy [5, 39]. One could consider such observations as the molecular explanation of poor outcomes observed in some cases of PAs, but in light of findings showing gene expression also in normal adult brain astrocytes, such connection should be rather be regarded as additional indication of a gene with a nearly unknown function. Aggressive clinical behavior is more frequently observed in pilocytic astrocytoma located within the hypothalamo-chiasmatic region, and up to now is regarded as the result of limited surgical excision. However, the relation of an unfavorable disease outcome and overexpression of genes contributes to cell growth, proliferation, and differentiation, and hence it could not be excluded that it also promotes the cancerogenesis and tumor metastasis observed in PAs [40–43]. On the other hand, the frequently observed (in our study) alteration of genes involved in brain structures’ development like homeobox transcription factors, contactins and miRNAs inducing neurogenesis, together with the generally benign behavior of PAs, lead to the statement that these tumors could be rather recognized by some scientists as a neurodevelopmental disorder [7, 27, 28, 32, 44]. Another argument for this is the coexistence of neurodevelopmental disorders and pilocytic astrocytomas [45–47]. Such observations, which suggest the plausible association of autism and cerebellar PAs, also raise the question of their similar genetic background.

Until the problem of the biological characteristics of pilocytic astrocytoma will be solved, and the degree of surgical resection remains the only clinically confirmed factor affecting recurrence or dissemination, there is still a need to identify the molecular markers influencing the clinical behavior of this tumor. Taking this into account, we tried to search how gene expression profiling is correlated with the clinical outcomes of children with PA. An additional incentive for us to perform such analyses was the limited studies on this subject [48–50]. Unfortunately in our study we didn’t observe a correlation between a tumor’s global gene expression profile and the clinical course of the disease. In the analysis of the univariate test of progressive and low-risk disease, only five genes differentiated between the subgroups with progressive disease and the others, but in the global test there were no real differences between the analyzed classes.

Hence our unsupervised analysis didn’t disclose any differences between PA of different outcomes. The problem with revealing genes connected with the progressive disease during expression analyses was also noted in other studies, where such correlation were excluded or postulated only for an extremely limited number of genes. Sharma et al. and Rodriguez et al. assumed that the *MATN2* and *ALDH1L1* genes are connected with the progression of PA, but it seems to be more an accidental occurrence than crucial molecular changes. This observation was confirmed in the following studies [9, 10]. In our opinion this could be an effect of the low biological aggressiveness of Pas, and finding molecular factors usable in clinical practice seems very unlikely. Moreover, malignant transformation of PAs is really a very rare phenomenon and possible histological misdiagnosis cannot be excluded.

We also tried to find any correlations between molecular profile and radiological features, a subject which was completely unknown for PAs. Such plausible connections were noted in other glial brain tumors such as glioblastoma and oligodendroglioma, but this was the first attempt to find them for PAs [8, 9, 51, 52]. Major attention was directed on tumor borders and enhancement patterns after contrast administration. Here we adapted the division of predominant imaging patterns of pilocytic astrocytoma first proposed by Pencalet et al. and modified by others [15, 16]. In our study radiological features presented by pilocytic astrocytoma were not associated with global gene expression profile. In the univariate test only four genes were differentiated between the subgroups, with no differences observed in the global test. Moreover, the genes which best discriminated between different radiological features of tumors had a relatively small amplitude of differences and were not good discriminators between the groups in clustering (Fig. 4). Previous studies indicated that radiological appearance could be a derivative of tumor location; solid tumors were frequently observed infratentorially, and optic tract tumors rarely consist of cystic elements [14–16, 53]. Therefore we performed additional analyses for a cohesive subgroup of infratentorial lesions, which also didn’t reveal any correlations. Similarly, analysis of two subgroups of tumors with mainly solid and mostly cystic features showed no associations with transcriptional pattern. Our results show that different radiological patterns of PA are not determined by trancriptional changes.

## Conclusions

In our comprehensive study we showed the differences between pilocytic astrocytoma of different locations and pointed out the heterogeneity of a tumor coming from the supratentorial region (Table 5). We may state that gene expression profile in pilocytic astrocytomas is connected only with tumor location, which suggests a different origin of PA arising within various anatomical brain structures. This observation is complementary to current knowledge of pilocytic astrocytoma’s biology and indicates that for targeted therapy three molecular subgroups of the tumor must be considered, particularly in the case of optimization of the treatment of optic nerve PAs. Our observation, combined with the similar results of a recent methylation study performed by Lambert et al., suggests that the molecular variation of PAs is probably the consequence of region-specific cells of origin’s presence, as is suggested in other brain tumors [54]. We pointed out the difficulties with finding the molecular alterations connected with worse clinical course of the disease, and found no connection between radiological features and global gene expression.

**Table 5:** Summary of expression analysis of pilocytic astrocytomas

Tumor Location	Hemisphere	Optic tracts	Cerebellum
Gene Family	FOXO	LHX SIX	IRX POU
Signaling Pathway	TLR		PTEN
	PI3/AKT MAPK TGFβ		
miRNA	miR-220 miR-299-3p		miR-486 miR-324-5p

## Additional files

Additional file 1: Table S1. Characteristics of probes used for validation of microarray data (DOCX 15 kb)
Additional file 2: Table S2. List of genes differentiating between pilocytic astrocytomas by different clinical features (DOCX 117 kb)
Additional file 3: Table S3. List of genes differentiating between pilocytic astrocytomas in the three most frequently observed locations. Comparison of three subgroups. FDR, false discovery rate; M1, cerebral hemispheric tumor; M2, optic tract and hypothalamic tumor; M3, cerebellar tumor (DOCX 169 kb)
Additional file 4: Table S4. List of genes differentiating between pilocytic astrocytomas in the three most frequently observed locations. Comparison of all supratentorial and infratentorial tumors. M1/ M2, supratentorial tumors; M3, infratentorial tumors. The microarray data discussed in this publication have been deposited in the NCBI’s Gene Expression Omnibus. The accession number is GSE73066 and the link to freely access is is http://www.ncbi.nlm.nih.gov/geo/query/acc.cgi?acc=GSE73066 (DOCX 72 kb)

## Abbreviations

FDR: False discovery rate
GCMRA: GC-robust multi-array
NF1: Neurofibromatosis type 1
NUSE: Normalized unsealed standard error
PA: Pilocytic astrocytoma
PCA: Principal component analysis
RT-qPCR: Quantitative real-time polymerase chain reaction
RIN: RNA integrity number
RLE: Relative log expression
