# Supplementary material for: Transcriptional profiles of pilocytic astrocytoma are related to their three different locations, but not to radiological tumor features
Source: BMC Cancer. 2015 Oct 24;15:778. doi: 10.1186/s12885-015-1810-z (PMC4619381; doi:10.1186/s12885-015-1810-z)
Supplement: Additional file 1: Table S1. — Characteristics of probes used for validation of microarray data (DOCX 15 kb) [file 12885_2015_1810_MOESM1_ESM.docx]

| **Gene symbol** | **Assay ID** | | **Entrez Gene ID** | | **Exon Boundary** | | **Amplicon lenght** |
| --- | --- | --- | --- | --- | --- | --- | --- |
| VALIDATED GENES | | | | | | | |
| ***IRX2*** | Hs01383002_m1 | 153572 | | 1-2 | | 85 | |
| ***PAX3*** | Hs00240950_m1 | 5077 | | 2-3 | | 145 | |
| ***CXCL14*** | Hs00171135_m1 | 9547 | | 3-4 | | 73 | |
| ***LHX2*** | Hs00180351_m1 | 9355 | | 3-4 | | 49 | |
| ***SIX6*** | Hs00201310_m1 | 4990 | | 1-2 | | 115 | |
| ***CNTN1*** | Hs00169986_m1 | 1272 | | 14-15 | | 112 | |
| ***SIX1*** | Hs00195590_m1 | 6495 | | 1-2 | | 79 | |
| REFERENCE GENE | | | | | | | |
| ***GAPDH*** | Hs99999905_m1 | | 2597 | | 3-3 | | 122 |
