# Supplementary material for: Transcriptional profiles of pilocytic astrocytoma are related to their three different locations, but not to radiological tumor features
Source: BMC Cancer. 2015 Oct 24;15:778. doi: 10.1186/s12885-015-1810-z (PMC4619381; doi:10.1186/s12885-015-1810-z)
Supplement: Additional file 2: Table S2. — List of genes differentiating between pilocytic astrocytomas by different clinical features (DOCX 117 kb) [file 12885_2015_1810_MOESM2_ESM.docx]

| **Probe set** | **Gene symbol** | **Description** | ***p*-value** | **FDR** | **1:  M1** | **2:  M2** | **3:  M3R1** | **4:  M3R2** | **5:  M3R3** | **6:  M3R4** | **7: NF1** | **8: P2** | **Pairwise significant** |
| --- | --- | --- | --- | --- | --- | --- | --- | --- | --- | --- | --- | --- | --- |
| [1561985_at](https://www.affymetrix.com/LinkServlet?probeset=1561985_at) | [C14orf39](http://www.ncbi.nlm.nih.gov/entrez/query.fcgi?cmd=search&db=gene&term=C14orf39) | chromosome 14 open reading frame 39 | < 1e-07 | < 1e-07 | 7.57 | 166.42 | 4.75 | 4.77 | 4.75 | 4.75 | 4.75 | 9.98 | (1, 2), (3, 2), (4, 2), (5, 2), (6, 2), (7, 2), (8, 2) |
| [226448_at](https://www.affymetrix.com/LinkServlet?probeset=226448_at) | [FAM89A](http://www.ncbi.nlm.nih.gov/entrez/query.fcgi?cmd=search&db=gene&term=FAM89A) | family with sequence similarity 89, member A | < 1e-07 | < 1e-07 | 66.93 | 760.13 | 626.27 | 1024.7 | 747.15 | 1083.92 | 299.7 | 488.54 | (1, 2), (1, 3), (1, 4), (1, 5), (1, 6), (1, 7), (1, 8), (7, 4), (7, 6) |
| [207250_at](https://www.affymetrix.com/LinkServlet?probeset=207250_at) | [SIX6](http://www.ncbi.nlm.nih.gov/entrez/query.fcgi?cmd=search&db=gene&term=SIX6) | SIX homeobox 6 | < 1e-07 | < 1e-07 | 8.29 | 384.78 | 4.75 | 4.75 | 4.75 | 4.75 | 4.75 | 24.89 | (1, 2), (3, 2), (4, 2), (5, 2), (6, 2), (7, 2), (8, 2), (3, 8), (4, 8), (5, 8), (6, 8) |
| [207963_at](https://www.affymetrix.com/LinkServlet?probeset=207963_at) | [C6orf54](http://www.ncbi.nlm.nih.gov/entrez/query.fcgi?cmd=search&db=gene&term=C6orf54) | chromosome 6 open reading frame 54 | < 1e-07 | < 1e-07 | 5.41 | 5.25 | 4.86 | 5.31 | 5.83 | 5.07 | 59.02 | 6.51 | (1, 7), (2, 7), (3, 7), (4, 7), (5, 7), (6, 7), (8, 7) |
| [211111_at](https://www.affymetrix.com/LinkServlet?probeset=211111_at) | [HGC6.3](http://www.ncbi.nlm.nih.gov/entrez/query.fcgi?cmd=search&db=gene&term=HGC6.3) | similar to HGC6.3 | < 1e-07 | < 1e-07 | 5.77 | 5.34 | 5.38 | 6.91 | 5.47 | 5.39 | 23.9 | 6.07 | (1, 7), (2, 7), (3, 7), (4, 7), (5, 7), (6, 7), (8, 7) |
| [228462_at](https://www.affymetrix.com/LinkServlet?probeset=228462_at) | [IRX2](http://www.ncbi.nlm.nih.gov/entrez/query.fcgi?cmd=search&db=gene&term=IRX2) | iroquois homeobox 2 | < 1e-07 | < 1e-07 | 8.65 | 46.07 | 471.6 | 632.71 | 376.91 | 428.5 | 70.56 | 25.39 | (1, 2), (1, 3), (1, 4), (1, 5), (1, 6), (2, 3), (2, 4), (2, 5), (2, 6), (8, 3), (7, 4), (8, 4), (8, 5), (8, 6) |
| [1554784_at](https://www.affymetrix.com/LinkServlet?probeset=1554784_at) | [CNTN1](http://www.ncbi.nlm.nih.gov/entrez/query.fcgi?cmd=search&db=gene&term=CNTN1) | contactin 1 | < 1e-07 | < 1e-07 | 61.8 | 11.93 | 282.68 | 416.51 | 331.65 | 413.62 | 720.42 | 68.01 | (2, 1), (1, 3), (1, 4), (1, 5), (1, 6), (1, 7), (2, 3), (2, 4), (2, 5), (2, 6), (2, 7), (2, 8), (8, 4), (8, 5), (8, 6), (8, 7) |
| [227202_at](https://www.affymetrix.com/LinkServlet?probeset=227202_at) | [CNTN1](http://www.ncbi.nlm.nih.gov/entrez/query.fcgi?cmd=search&db=gene&term=CNTN1) | contactin 1 | < 1e-07 | < 1e-07 | 222.8 | 27.13 | 1410.45 | 1886.29 | 1457.58 | 1954.74 | 2143.38 | 374.5 | (2, 1), (1, 3), (1, 4), (1, 5), (1, 6), (2, 3), (2, 4), (2, 5), (2, 6), (2, 7), (2, 8) |
| [213285_at](https://www.affymetrix.com/LinkServlet?probeset=213285_at) | [TMEM30B](http://www.ncbi.nlm.nih.gov/entrez/query.fcgi?cmd=search&db=gene&term=TMEM30B) | transmembrane protein 30B | < 1e-07 | < 1e-07 | 7.94 | 34.41 | 5.15 | 5.24 | 4.97 | 4.94 | 5.22 | 8.98 | (1, 2), (3, 2), (4, 2), (5, 2), (6, 2), (7, 2), (8, 2) |
| [231666_at](https://www.affymetrix.com/LinkServlet?probeset=231666_at) | [PAX3](http://www.ncbi.nlm.nih.gov/entrez/query.fcgi?cmd=search&db=gene&term=PAX3) | paired box 3 | < 1e-07 | < 1e-07 | 5.04 | 8.56 | 167.66 | 185.45 | 414.26 | 333.68 | 82.98 | 12.8 | (1, 3), (1, 4), (1, 5), (1, 6), (1, 7), (2, 3), (2, 4), (2, 5), (2, 6), (8, 3), (8, 4), (8, 5), (8, 6) |
| [219885_at](https://www.affymetrix.com/LinkServlet?probeset=219885_at) | [SLFN12](http://www.ncbi.nlm.nih.gov/entrez/query.fcgi?cmd=search&db=gene&term=SLFN12) | schlafen family member 12 | 1e-07 | 0.000156 | 16.77 | 59.9 | 12.14 | 14.73 | 16.96 | 15.73 | 33.17 | 26.9 | (1, 2), (3, 2), (4, 2), (5, 2), (6, 2), (8, 2), (3, 7), (3, 8) |
| [223582_at](https://www.affymetrix.com/LinkServlet?probeset=223582_at) | [GPR98](http://www.ncbi.nlm.nih.gov/entrez/query.fcgi?cmd=search&db=gene&term=GPR98) | G protein-coupled receptor 98 | 1e-07 | 0.000156 | 43.59 | 170.97 | 12.92 | 12.12 | 18.15 | 17.78 | 46.46 | 61.05 | (1, 2), (3, 1), (4, 1), (3, 2), (4, 2), (5, 2), (6, 2), (3, 8), (4, 8), (5, 8), (6, 8) |
| [220117_at](https://www.affymetrix.com/LinkServlet?probeset=220117_at) | [ZNF385D](http://www.ncbi.nlm.nih.gov/entrez/query.fcgi?cmd=search&db=gene&term=ZNF385D) | zinc finger protein 385D | 1e-07 | 0.000156 | 21.7 | 60.59 | 119.85 | 125.33 | 82.96 | 142.62 | 122.87 | 64.75 | (1, 2), (1, 3), (1, 4), (1, 5), (1, 6), (1, 7), (1, 8), (2, 3), (2, 4), (2, 6), (8, 6) |
| [206140_at](https://www.affymetrix.com/LinkServlet?probeset=206140_at) | [LHX2](http://www.ncbi.nlm.nih.gov/entrez/query.fcgi?cmd=search&db=gene&term=LHX2) | LIM homeobox 2 | 1e-07 | 0.000156 | 1592.51 | 2617.53 | 13.37 | 7.73 | 14.57 | 12.57 | 118.57 | 691.6 | (3, 1), (4, 1), (5, 1), (6, 1), (3, 2), (4, 2), (5, 2), (6, 2), (3, 8), (4, 8), (5, 8), (6, 8) |
| [227614_at](https://www.affymetrix.com/LinkServlet?probeset=227614_at) | [HKDC1](http://www.ncbi.nlm.nih.gov/entrez/query.fcgi?cmd=search&db=gene&term=HKDC1) | hexokinase domain containing 1 | 4e-07 | 0.000584 | 28.97 | 9.17 | 8.52 | 7.9 | 7.05 | 7.74 | 47.47 | 10.31 | (2, 1), (3, 1), (4, 1), (5, 1), (6, 1), (8, 1), (2, 7), (3, 7), (4, 7), (5, 7), (6, 7), (8, 7) |
| [217498_at](https://www.affymetrix.com/LinkServlet?probeset=217498_at) | [NA](http://www.ncbi.nlm.nih.gov/entrez/query.fcgi?cmd=search&db=gene&term=NA) | NA | 5e-07 | 0.000644 | 9.53 | 7.22 | 11.24 | 9.96 | 10.62 | 12.46 | 73.41 | 12.59 | (1, 7), (2, 6), (2, 7), (2, 8), (3, 7), (4, 7), (5, 7), (6, 7), (8, 7) |
| [228307_at](https://www.affymetrix.com/LinkServlet?probeset=228307_at) | [EMILIN3](http://www.ncbi.nlm.nih.gov/entrez/query.fcgi?cmd=search&db=gene&term=EMILIN3) | elastin microfibril interfacer 3 | 5e-07 | 0.000644 | 23.53 | 53.27 | 73.4 | 287.73 | 212.1 | 240.14 | 83.83 | 57.99 | (1, 3), (1, 4), (1, 5), (1, 6), (2, 4), (2, 5), (2, 6), (3, 4), (3, 6), (8, 4), (8, 5), (8, 6) |
| [227209_at](https://www.affymetrix.com/LinkServlet?probeset=227209_at) | [CNTN1](http://www.ncbi.nlm.nih.gov/entrez/query.fcgi?cmd=search&db=gene&term=CNTN1) | contactin 1 | 7e-07 | 0.000852 | 163.29 | 20.67 | 644.33 | 771.15 | 1248.09 | 1047.36 | 1676.02 | 243.57 | (2, 1), (1, 5), (1, 6), (2, 3), (2, 4), (2, 5), (2, 6), (2, 7), (2, 8) |
| [224215_s_at](https://www.affymetrix.com/LinkServlet?probeset=224215_s_at) | [DLL1](http://www.ncbi.nlm.nih.gov/entrez/query.fcgi?cmd=search&db=gene&term=DLL1) | delta-like 1 (Drosophila) | 8e-07 | 0.000923 | 32.48 | 83.36 | 122.89 | 292.53 | 218.29 | 434.72 | 94.15 | 39.24 | (1, 3), (1, 4), (1, 5), (1, 6), (2, 4), (2, 6), (3, 6), (8, 4), (8, 5), (8, 6) |
| [232275_s_at](https://www.affymetrix.com/LinkServlet?probeset=232275_s_at) | [HS6ST3](http://www.ncbi.nlm.nih.gov/entrez/query.fcgi?cmd=search&db=gene&term=HS6ST3) | heparan sulfate 6-O-sulfotransferase 3 | 1e-06 | 0.0011 | 7.1 | 6.64 | 25.25 | 62.18 | 24.87 | 58.96 | 73.76 | 24.63 | (1, 3), (1, 4), (1, 5), (1, 6), (1, 7), (1, 8), (2, 3), (2, 4), (2, 5), (2, 6), (2, 7), (2, 8) |
| [1563480_at](https://www.affymetrix.com/LinkServlet?probeset=1563480_at) | [NA](http://www.ncbi.nlm.nih.gov/entrez/query.fcgi?cmd=search&db=gene&term=NA) | NA | 1.3e-06 | 0.00136 | 5.54 | 4.81 | 6.51 | 12.08 | 5.43 | 5.26 | 4.83 | 4.82 | (1, 4), (2, 4), (3, 4), (5, 4), (6, 4), (7, 4), (8, 4) |
| [230720_at](https://www.affymetrix.com/LinkServlet?probeset=230720_at) | [RNF182](http://www.ncbi.nlm.nih.gov/entrez/query.fcgi?cmd=search&db=gene&term=RNF182) | ring finger protein 182 | 1.4e-06 | 0.00139 | 152.91 | 2152.78 | 1857.68 | 2167.82 | 1666.66 | 2327.4 | 448.02 | 1353.52 | (1, 2), (1, 3), (1, 4), (1, 5), (1, 6), (1, 8) |
| [228347_at](https://www.affymetrix.com/LinkServlet?probeset=228347_at) | [SIX1](http://www.ncbi.nlm.nih.gov/entrez/query.fcgi?cmd=search&db=gene&term=SIX1) | SIX homeobox 1 | 1.7e-06 | 0.00162 | 16.15 | 697.28 | 12.37 | 7.67 | 11.54 | 6.34 | 5.17 | 49.54 | (1, 2), (3, 2), (4, 2), (5, 2), (6, 2), (7, 2), (8, 2) |
| [238727_at](https://www.affymetrix.com/LinkServlet?probeset=238727_at) | [LOC440934](http://www.ncbi.nlm.nih.gov/entrez/query.fcgi?cmd=search&db=gene&term=LOC440934) | hypothetical LOC440934 | 2.1e-06 | 0.00192 | 22.16 | 14.66 | 128.59 | 194.37 | 386.13 | 446.67 | 85.4 | 32.45 | (1, 3), (1, 4), (1, 5), (1, 6), (2, 3), (2, 4), (2, 5), (2, 6), (8, 5), (8, 6) |
| [214954_at](https://www.affymetrix.com/LinkServlet?probeset=214954_at) | [SUSD5](http://www.ncbi.nlm.nih.gov/entrez/query.fcgi?cmd=search&db=gene&term=SUSD5) | sushi domain containing 5 | 2.2e-06 | 0.00193 | 112.28 | 1255.24 | 1206.37 | 2031.7 | 1652.75 | 2014.84 | 549.72 | 526.2 | (1, 2), (1, 3), (1, 4), (1, 5), (1, 6), (1, 8) |
| [236692_at](https://www.affymetrix.com/LinkServlet?probeset=236692_at) | [LOC729839](http://www.ncbi.nlm.nih.gov/entrez/query.fcgi?cmd=search&db=gene&term=LOC729839) | similar to DTW domain containing 2 | 2.3e-06 | 0.00194 | 8.27 | 24.8 | 7.67 | 7.94 | 8.4 | 8.03 | 14.03 | 9.23 | (1, 2), (3, 2), (4, 2), (5, 2), (6, 2), (8, 2) |
| [211219_s_at](https://www.affymetrix.com/LinkServlet?probeset=211219_s_at) | [LHX2](http://www.ncbi.nlm.nih.gov/entrez/query.fcgi?cmd=search&db=gene&term=LHX2) | LIM homeobox 2 | 2.5e-06 | 0.00203 | 81.14 | 85.02 | 7 | 4.82 | 4.91 | 5.72 | 27.22 | 50.64 | (3, 1), (4, 1), (5, 1), (6, 1), (3, 2), (4, 2), (5, 2), (6, 2), (3, 8), (4, 8), (5, 8), (6, 8) |
| [242670_at](https://www.affymetrix.com/LinkServlet?probeset=242670_at) | [LGI4](http://www.ncbi.nlm.nih.gov/entrez/query.fcgi?cmd=search&db=gene&term=LGI4) | leucine-rich repeat LGI family, member 4 | 2.7e-06 | 0.00211 | 64.55 | 65.18 | 64.09 | 14.77 | 21.35 | 10.99 | 295.8 | 44.04 | (4, 1), (5, 1), (6, 1), (4, 2), (5, 2), (6, 2), (4, 3), (6, 3), (4, 7), (5, 7), (6, 7), (6, 8), (8, 7) |
| [208464_at](https://www.affymetrix.com/LinkServlet?probeset=208464_at) | [GRIA4](http://www.ncbi.nlm.nih.gov/entrez/query.fcgi?cmd=search&db=gene&term=GRIA4) | glutamate receptor, ionotrophic, AMPA 4 | 3.5e-06 | 0.00264 | 5.55 | 19.4 | 33.21 | 47.22 | 38.84 | 43.45 | 43.9 | 10.41 | (1, 2), (1, 3), (1, 4), (1, 5), (1, 6), (1, 7), (8, 3), (8, 4), (8, 5), (8, 6) |
| [228720_at](https://www.affymetrix.com/LinkServlet?probeset=228720_at) | [SORCS2](http://www.ncbi.nlm.nih.gov/entrez/query.fcgi?cmd=search&db=gene&term=SORCS2) | sortilin-related VPS10 domain containing receptor 2 | 3.7e-06 | 0.0027 | 30.82 | 15.43 | 17.04 | 14.73 | 19.65 | 13.86 | 189.86 | 21.07 | (2, 1), (4, 1), (6, 1), (1, 7), (2, 7), (3, 7), (4, 7), (5, 7), (6, 7), (8, 7) |
| [225666_at](https://www.affymetrix.com/LinkServlet?probeset=225666_at) | [TMTC4](http://www.ncbi.nlm.nih.gov/entrez/query.fcgi?cmd=search&db=gene&term=TMTC4) | transmembrane and tetratricopeptide repeat containing 4 | 4e-06 | 0.00283 | 737.39 | 260.1 | 193.04 | 98.37 | 177.46 | 134.81 | 1023.08 | 364.36 | (2, 1), (3, 1), (4, 1), (5, 1), (6, 1), (4, 2), (2, 7), (3, 7), (4, 7), (4, 8), (5, 7), (6, 7) |
| [226743_at](https://www.affymetrix.com/LinkServlet?probeset=226743_at) | [SLFN11](http://www.ncbi.nlm.nih.gov/entrez/query.fcgi?cmd=search&db=gene&term=SLFN11) | schlafen family member 11 | 5.9e-06 | 0.00404 | 40.39 | 220.31 | 25.19 | 25.43 | 50.07 | 32.8 | 117.69 | 82.37 | (1, 2), (3, 2), (4, 2), (5, 2), (6, 2), (3, 7), (3, 8), (4, 8) |
| [229714_at](https://www.affymetrix.com/LinkServlet?probeset=229714_at) | [HS6ST3](http://www.ncbi.nlm.nih.gov/entrez/query.fcgi?cmd=search&db=gene&term=HS6ST3) | heparan sulfate 6-O-sulfotransferase 3 | 6.9e-06 | 0.00458 | 14.51 | 14.8 | 46.59 | 106.16 | 56.38 | 93.31 | 137.71 | 51.33 | (1, 3), (1, 4), (1, 5), (1, 6), (1, 7), (1, 8), (2, 3), (2, 4), (2, 5), (2, 6), (2, 7), (2, 8) |
| [238878_at](https://www.affymetrix.com/LinkServlet?probeset=238878_at) | [ARX](http://www.ncbi.nlm.nih.gov/entrez/query.fcgi?cmd=search&db=gene&term=ARX) | aristaless related homeobox | 8e-06 | 0.00516 | 132.42 | 6.43 | 4.76 | 4.8 | 5.07 | 4.81 | 42.45 | 6.63 | (2, 1), (3, 1), (4, 1), (5, 1), (6, 1), (8, 1) |
| [205593_s_at](https://www.affymetrix.com/LinkServlet?probeset=205593_s_at) | [PDE9A](http://www.ncbi.nlm.nih.gov/entrez/query.fcgi?cmd=search&db=gene&term=PDE9A) | phosphodiesterase 9A | 9.3e-06 | 0.00582 | 79.88 | 15.65 | 64.52 | 146.83 | 91.81 | 113.19 | 160.32 | 51.14 | (2, 1), (2, 3), (2, 4), (2, 5), (2, 6), (2, 7), (2, 8) |
| [1554507_at](https://www.affymetrix.com/LinkServlet?probeset=1554507_at) | [NAALAD2](http://www.ncbi.nlm.nih.gov/entrez/query.fcgi?cmd=search&db=gene&term=NAALAD2) | N-acetylated alpha-linked acidic dipeptidase 2 | 1.01e-05 | 0.00615 | 8.95 | 8.04 | 50.97 | 98.85 | 62.94 | 71.33 | 55.97 | 16.4 | (1, 3), (1, 4), (1, 5), (1, 6), (2, 3), (2, 4), (2, 5), (2, 6), (8, 4) |
| [222895_s_at](https://www.affymetrix.com/LinkServlet?probeset=222895_s_at) | [BCL11B](http://www.ncbi.nlm.nih.gov/entrez/query.fcgi?cmd=search&db=gene&term=BCL11B) | B-cell CLL/lymphoma 11B (zinc finger protein) | 1.13e-05 | 0.00669 | 13.02 | 13.52 | 20.91 | 57.18 | 51.46 | 62.81 | 7.38 | 8.83 | (1, 4), (1, 5), (1, 6), (2, 4), (2, 5), (2, 6), (7, 4), (8, 4), (7, 5), (8, 5), (7, 6), (8, 6) |
| [208359_s_at](https://www.affymetrix.com/LinkServlet?probeset=208359_s_at) | [KCNJ4](http://www.ncbi.nlm.nih.gov/entrez/query.fcgi?cmd=search&db=gene&term=KCNJ4) | potassium inwardly-rectifying channel, subfamily J, member 4 | 1.34e-05 | 0.00773 | 11.1 | 4.75 | 4.75 | 4.75 | 4.75 | 4.75 | 21.71 | 4.75 | (2, 1), (3, 1), (4, 1), (5, 1), (6, 1), (8, 1), (2, 7), (3, 7), (4, 7), (5, 7), (6, 7), (8, 7) |
| [230960_at](https://www.affymetrix.com/LinkServlet?probeset=230960_at) | [IGDCC3](http://www.ncbi.nlm.nih.gov/entrez/query.fcgi?cmd=search&db=gene&term=IGDCC3) | immunoglobulin superfamily, DCC subclass, member 3 | 1.45e-05 | 0.00791 | 47.24 | 9.69 | 24.72 | 19.04 | 18.22 | 19 | 80.83 | 19.09 | (2, 1), (4, 1), (5, 1), (6, 1), (8, 1), (2, 3), (2, 7), (3, 7), (4, 7), (5, 7), (6, 7), (8, 7) |
| [222482_at](https://www.affymetrix.com/LinkServlet?probeset=222482_at) | [NA](http://www.ncbi.nlm.nih.gov/entrez/query.fcgi?cmd=search&db=gene&term=NA) | NA | 1.45e-05 | 0.00791 | 252.7 | 64.2 | 322.03 | 242.65 | 246.8 | 266.62 | 453.74 | 144.06 | (2, 1), (2, 3), (2, 4), (2, 5), (2, 6), (2, 7) |
| [205817_at](https://www.affymetrix.com/LinkServlet?probeset=205817_at) | [SIX1](http://www.ncbi.nlm.nih.gov/entrez/query.fcgi?cmd=search&db=gene&term=SIX1) | SIX homeobox 1 | 1.48e-05 | 0.00791 | 7.56 | 51.08 | 5.8 | 4.75 | 5.56 | 4.75 | 4.75 | 9.13 | (1, 2), (3, 2), (4, 2), (5, 2), (6, 2), (7, 2), (8, 2) |
| [227821_at](https://www.affymetrix.com/LinkServlet?probeset=227821_at) | [LGI4](http://www.ncbi.nlm.nih.gov/entrez/query.fcgi?cmd=search&db=gene&term=LGI4) | leucine-rich repeat LGI family, member 4 | 1.52e-05 | 0.00793 | 116.25 | 83.88 | 91.19 | 19.91 | 25.26 | 17.71 | 363.15 | 86.58 | (4, 1), (5, 1), (6, 1), (4, 2), (5, 2), (6, 2), (4, 3), (5, 3), (6, 3), (4, 7), (4, 8), (5, 7), (6, 7), (6, 8) |
| [227297_at](https://www.affymetrix.com/LinkServlet?probeset=227297_at) | [ITGA9](http://www.ncbi.nlm.nih.gov/entrez/query.fcgi?cmd=search&db=gene&term=ITGA9) | integrin, alpha 9 | 1.58e-05 | 0.00805 | 47.44 | 191.45 | 109.94 | 221.6 | 217.39 | 260.19 | 84.31 | 79.35 | (1, 2), (1, 4), (1, 5), (1, 6), (8, 2), (8, 4), (8, 5), (8, 6) |
| [229545_at](https://www.affymetrix.com/LinkServlet?probeset=229545_at) | [FERMT1](http://www.ncbi.nlm.nih.gov/entrez/query.fcgi?cmd=search&db=gene&term=FERMT1) | fermitin family homolog 1 (Drosophila) | 1.81e-05 | 0.00901 | 15.91 | 13.74 | 29.69 | 65.25 | 31.2 | 175.08 | 49.05 | 13.82 | (1, 4), (1, 6), (2, 4), (2, 6), (3, 6), (8, 4), (5, 6), (8, 6) |
| [202834_at](https://www.affymetrix.com/LinkServlet?probeset=202834_at) | [AGT](http://www.ncbi.nlm.nih.gov/entrez/query.fcgi?cmd=search&db=gene&term=AGT) | angiotensinogen (serpin peptidase inhibitor, clade A, member 8) | 1.9e-05 | 0.00917 | 898.02 | 692.69 | 3216.96 | 3413.4 | 4446.67 | 5449.34 | 1706.89 | 2316.98 | (1, 3), (1, 4), (1, 5), (1, 6), (2, 3), (2, 4), (2, 5), (2, 6), (2, 8) |
| [220595_at](https://www.affymetrix.com/LinkServlet?probeset=220595_at) | [PDZRN4](http://www.ncbi.nlm.nih.gov/entrez/query.fcgi?cmd=search&db=gene&term=PDZRN4) | PDZ domain containing ring finger 4 | 1.94e-05 | 0.00917 | 11.97 | 23.74 | 58.91 | 171 | 110.44 | 149.97 | 220.25 | 27.28 | (1, 3), (1, 4), (1, 5), (1, 6), (1, 7), (2, 4), (2, 5), (2, 6), (2, 7), (8, 4), (8, 6) |
| [235494_at](https://www.affymetrix.com/LinkServlet?probeset=235494_at) | [NA](http://www.ncbi.nlm.nih.gov/entrez/query.fcgi?cmd=search&db=gene&term=NA) | NA | 1.98e-05 | 0.00917 | 1128.46 | 1368.91 | 2737.68 | 2998.85 | 1991.5 | 2771.71 | 2643.13 | 2093.77 | (1, 3), (1, 4), (1, 5), (1, 6), (1, 7), (1, 8), (2, 3), (2, 4), (2, 6) |
| [225504_at](https://www.affymetrix.com/LinkServlet?probeset=225504_at) | [NA](http://www.ncbi.nlm.nih.gov/entrez/query.fcgi?cmd=search&db=gene&term=NA) | NA | 2.01e-05 | 0.00917 | 285.23 | 157.46 | 324.02 | 496.99 | 410.29 | 409.6 | 497.23 | 236.33 | (2, 1), (2, 3), (2, 4), (2, 5), (2, 6), (2, 7), (8, 4) |
| [203820_s_at](https://www.affymetrix.com/LinkServlet?probeset=203820_s_at) | [IGF2BP3](http://www.ncbi.nlm.nih.gov/entrez/query.fcgi?cmd=search&db=gene&term=IGF2BP3) | insulin-like growth factor 2 mRNA binding protein 3 | 2.33e-05 | 0.0104 | 22.26 | 379.27 | 8.87 | 16.74 | 27.79 | 23.7 | 25.9 | 34.21 | (1, 2), (3, 2), (4, 2), (5, 2), (6, 2), (7, 2), (8, 2) |
| [241396_at](https://www.affymetrix.com/LinkServlet?probeset=241396_at) | [NEDD4L](http://www.ncbi.nlm.nih.gov/entrez/query.fcgi?cmd=search&db=gene&term=NEDD4L) | neural precursor cell expressed, developmentally down-regulated 4-like | 2.38e-05 | 0.0104 | 46.39 | 11.8 | 13.1 | 10.98 | 11.64 | 11.09 | 25.06 | 14.75 | (2, 1), (3, 1), (4, 1), (5, 1), (6, 1), (8, 1) |
| [214460_at](https://www.affymetrix.com/LinkServlet?probeset=214460_at) | [LSAMP](http://www.ncbi.nlm.nih.gov/entrez/query.fcgi?cmd=search&db=gene&term=LSAMP) | limbic system-associated membrane protein | 2.47e-05 | 0.0106 | 201.19 | 172.83 | 389.34 | 442.51 | 412.26 | 608.05 | 385.55 | 280.97 | (1, 3), (1, 4), (1, 5), (1, 6), (2, 3), (2, 4), (2, 5), (2, 6), (8, 6) |
| [208221_s_at](https://www.affymetrix.com/LinkServlet?probeset=208221_s_at) | [SLIT1](http://www.ncbi.nlm.nih.gov/entrez/query.fcgi?cmd=search&db=gene&term=SLIT1) | slit homolog 1 (Drosophila) | 2.61e-05 | 0.011 | 8.78 | 18.92 | 5.86 | 6.54 | 6.16 | 5.77 | 5.6 | 10.98 | (1, 2), (3, 2), (4, 2), (5, 2), (6, 2), (7, 2) |
| [230458_at](https://www.affymetrix.com/LinkServlet?probeset=230458_at) | [SLC45A1](http://www.ncbi.nlm.nih.gov/entrez/query.fcgi?cmd=search&db=gene&term=SLC45A1) | solute carrier family 45, member 1 | 2.87e-05 | 0.0119 | 27.86 | 29.38 | 74.46 | 49.03 | 52.36 | 65.9 | 41.01 | 38.23 | (1, 3), (1, 4), (1, 5), (1, 6), (2, 3), (2, 4), (2, 5), (2, 6), (8, 3) |
| [1552634_a_at](https://www.affymetrix.com/LinkServlet?probeset=1552634_a_at) | [ZNF101](http://www.ncbi.nlm.nih.gov/entrez/query.fcgi?cmd=search&db=gene&term=ZNF101) | zinc finger protein 101 | 2.99e-05 | 0.0121 | 10.54 | 15.45 | 10.33 | 7.85 | 9.39 | 8.04 | 7.34 | 10.78 | (1, 2), (3, 2), (4, 2), (5, 2), (6, 2), (7, 2), (8, 2) |
| [218974_at](https://www.affymetrix.com/LinkServlet?probeset=218974_at) | [SOBP](http://www.ncbi.nlm.nih.gov/entrez/query.fcgi?cmd=search&db=gene&term=SOBP) | sine oculis binding protein homolog (Drosophila) | 3.03e-05 | 0.0121 | 1459.08 | 1064.2 | 2369.88 | 2768.31 | 2245.98 | 2687.66 | 2943.02 | 1528.67 | (1, 4), (1, 6), (2, 3), (2, 4), (2, 5), (2, 6), (2, 7), (8, 4) |
| [232244_at](https://www.affymetrix.com/LinkServlet?probeset=232244_at) | [KIAA1161](http://www.ncbi.nlm.nih.gov/entrez/query.fcgi?cmd=search&db=gene&term=KIAA1161) | KIAA1161 | 3.31e-05 | 0.0125 | 45.09 | 18.44 | 22.48 | 22.17 | 32.66 | 24.38 | 116.87 | 29.24 | (2, 1), (3, 1), (4, 1), (1, 7), (2, 7), (3, 7), (4, 7), (5, 7), (6, 7), (8, 7) |
| [209472_at](https://www.affymetrix.com/LinkServlet?probeset=209472_at) | [CCBL2](http://www.ncbi.nlm.nih.gov/entrez/query.fcgi?cmd=search&db=gene&term=CCBL2) | cysteine conjugate-beta lyase 2 | 3.31e-05 | 0.0125 | 562.92 | 475.65 | 355.07 | 263.06 | 375.49 | 295.36 | 309.4 | 506.25 | (3, 1), (4, 1), (5, 1), (6, 1), (7, 1), (4, 2), (6, 2), (4, 8), (6, 8) |
| [226145_s_at](https://www.affymetrix.com/LinkServlet?probeset=226145_s_at) | [FRAS1](http://www.ncbi.nlm.nih.gov/entrez/query.fcgi?cmd=search&db=gene&term=FRAS1) | Fraser syndrome 1 | 3.32e-05 | 0.0125 | 14.43 | 21.32 | 6.18 | 4.83 | 5.91 | 4.75 | 661.17 | 7.15 | (1, 7), (2, 7), (3, 7), (4, 7), (5, 7), (6, 7), (8, 7) |
| [213373_s_at](https://www.affymetrix.com/LinkServlet?probeset=213373_s_at) | [CASP8](http://www.ncbi.nlm.nih.gov/entrez/query.fcgi?cmd=search&db=gene&term=CASP8) | caspase 8, apoptosis-related cysteine peptidase | 3.48e-05 | 0.0129 | 122.78 | 199.69 | 51.71 | 76.19 | 122.62 | 76.17 | 37.94 | 125.75 | (3, 1), (7, 1), (3, 2), (4, 2), (6, 2), (7, 2), (3, 5), (3, 8), (7, 5), (7, 8) |
| [244764_at](https://www.affymetrix.com/LinkServlet?probeset=244764_at) | [NA](http://www.ncbi.nlm.nih.gov/entrez/query.fcgi?cmd=search&db=gene&term=NA) | NA | 3.9e-05 | 0.0142 | 104.32 | 203.88 | 86.35 | 69.75 | 71.27 | 75.4 | 95.14 | 160.32 | (1, 2), (3, 2), (4, 2), (5, 2), (6, 2), (4, 8), (5, 8), (6, 8) |
| [218796_at](https://www.affymetrix.com/LinkServlet?probeset=218796_at) | [FERMT1](http://www.ncbi.nlm.nih.gov/entrez/query.fcgi?cmd=search&db=gene&term=FERMT1) | fermitin family homolog 1 (Drosophila) | 4.05e-05 | 0.0144 | 32.47 | 57.26 | 139.64 | 542.92 | 165.93 | 356.41 | 148.63 | 60.57 | (1, 3), (1, 4), (1, 5), (1, 6), (2, 4), (2, 6), (8, 4), (8, 6) |
| [229831_at](https://www.affymetrix.com/LinkServlet?probeset=229831_at) | [CNTN3](http://www.ncbi.nlm.nih.gov/entrez/query.fcgi?cmd=search&db=gene&term=CNTN3) | contactin 3 (plasmacytoma associated) | 4.07e-05 | 0.0144 | 20.67 | 10.08 | 100.6 | 251.26 | 194.29 | 199.23 | 58.68 | 22 | (1, 4), (1, 5), (1, 6), (2, 3), (2, 4), (2, 5), (2, 6), (8, 4), (8, 5), (8, 6) |
| [1558508_a_at](https://www.affymetrix.com/LinkServlet?probeset=1558508_a_at) | [C1orf53](http://www.ncbi.nlm.nih.gov/entrez/query.fcgi?cmd=search&db=gene&term=C1orf53) | chromosome 1 open reading frame 53 | 4.15e-05 | 0.0144 | 57.59 | 46.26 | 11.92 | 9.93 | 10.01 | 10.53 | 14.36 | 28.88 | (3, 1), (4, 1), (5, 1), (6, 1), (3, 2), (4, 2), (5, 2), (6, 2) |
| [226487_at](https://www.affymetrix.com/LinkServlet?probeset=226487_at) | [C12orf34](http://www.ncbi.nlm.nih.gov/entrez/query.fcgi?cmd=search&db=gene&term=C12orf34) | chromosome 12 open reading frame 34 | 4.54e-05 | 0.0155 | 57.84 | 52.46 | 159.01 | 132.21 | 133.89 | 155.85 | 163.77 | 60.37 | (1, 3), (1, 4), (1, 5), (1, 6), (2, 3), (2, 4), (2, 5), (2, 6), (2, 7), (8, 3), (8, 5), (8, 6) |
| [1558388_a_at](https://www.affymetrix.com/LinkServlet?probeset=1558388_a_at) | [LOC643763](http://www.ncbi.nlm.nih.gov/entrez/query.fcgi?cmd=search&db=gene&term=LOC643763) | hypothetical LOC643763 | 4.78e-05 | 0.016 | 611.84 | 24.88 | 594.5 | 816.81 | 728.83 | 604.72 | 2817.91 | 283.9 | (2, 1), (2, 3), (2, 4), (2, 5), (2, 6), (2, 7), (2, 8) |
| [238076_at](https://www.affymetrix.com/LinkServlet?probeset=238076_at) | [GATAD2B](http://www.ncbi.nlm.nih.gov/entrez/query.fcgi?cmd=search&db=gene&term=GATAD2B) | GATA zinc finger domain containing 2B | 4.81e-05 | 0.016 | 490.21 | 355.22 | 625.17 | 836.96 | 494.05 | 643.96 | 955.84 | 437.19 | (1, 4), (1, 7), (2, 3), (2, 4), (2, 6), (2, 7), (5, 4), (8, 4), (5, 7), (8, 7) |
| [225746_at](https://www.affymetrix.com/LinkServlet?probeset=225746_at) | [RAB11FIP4](http://www.ncbi.nlm.nih.gov/entrez/query.fcgi?cmd=search&db=gene&term=RAB11FIP4) | RAB11 family interacting protein 4 (class II) | 5.16e-05 | 0.0167 | 32.07 | 9.13 | 13.95 | 12.8 | 13.17 | 9.7 | 75.11 | 15.08 | (2, 1), (3, 1), (4, 1), (5, 1), (6, 1), (2, 7), (3, 7), (4, 7), (5, 7), (6, 7), (8, 7) |
| [208296_x_at](https://www.affymetrix.com/LinkServlet?probeset=208296_x_at) | [TNFAIP8](http://www.ncbi.nlm.nih.gov/entrez/query.fcgi?cmd=search&db=gene&term=TNFAIP8) | tumor necrosis factor, alpha-induced protein 8 | 5.19e-05 | 0.0167 | 106.65 | 166.92 | 38.29 | 48.97 | 100.27 | 55.72 | 34.05 | 92.62 | (3, 1), (7, 1), (3, 2), (4, 2), (6, 2), (7, 2), (3, 5), (3, 8) |
| [1556627_at](https://www.affymetrix.com/LinkServlet?probeset=1556627_at) | [DRP2](http://www.ncbi.nlm.nih.gov/entrez/query.fcgi?cmd=search&db=gene&term=DRP2) | dystrophin related protein 2 | 5.36e-05 | 0.0168 | 13.98 | 22.64 | 42.97 | 105.68 | 53.4 | 89.5 | 34.07 | 42.45 | (1, 3), (1, 4), (1, 5), (1, 6), (1, 8), (2, 4), (2, 6) |
| [207705_s_at](https://www.affymetrix.com/LinkServlet?probeset=207705_s_at) | [NINL](http://www.ncbi.nlm.nih.gov/entrez/query.fcgi?cmd=search&db=gene&term=NINL) | ninein-like | 5.37e-05 | 0.0168 | 98.56 | 95.71 | 197.06 | 218.2 | 133.55 | 145.8 | 159.74 | 101.18 | (1, 3), (1, 4), (2, 3), (2, 4), (8, 3), (5, 4), (8, 4) |
| [60474_at](https://www.affymetrix.com/LinkServlet?probeset=60474_at) | [FERMT1](http://www.ncbi.nlm.nih.gov/entrez/query.fcgi?cmd=search&db=gene&term=FERMT1) | fermitin family homolog 1 (Drosophila) | 5.57e-05 | 0.0172 | 41.61 | 63.02 | 152.01 | 589.46 | 197.4 | 448.46 | 217.26 | 77.18 | (1, 4), (1, 5), (1, 6), (2, 4), (2, 6), (8, 4), (8, 6) |
| [220979_s_at](https://www.affymetrix.com/LinkServlet?probeset=220979_s_at) | [ST6GALNAC5](http://www.ncbi.nlm.nih.gov/entrez/query.fcgi?cmd=search&db=gene&term=ST6GALNAC5) | ST6 (alpha-N-acetyl-neuraminyl-2,3-beta-galactosyl-1,3)-N-acetylgalactosaminide alpha-2,6-sialyltransferase 5 | 5.84e-05 | 0.0176 | 15.78 | 4.75 | 4.75 | 4.76 | 4.75 | 4.78 | 5.69 | 4.97 | (2, 1), (3, 1), (4, 1), (5, 1), (6, 1), (7, 1), (8, 1) |
| [219331_s_at](https://www.affymetrix.com/LinkServlet?probeset=219331_s_at) | [KLHDC8A](http://www.ncbi.nlm.nih.gov/entrez/query.fcgi?cmd=search&db=gene&term=KLHDC8A) | kelch domain containing 8A | 5.9e-05 | 0.0176 | 54.19 | 63.57 | 32.47 | 18.13 | 21.65 | 18.01 | 175.98 | 55.2 | (4, 1), (6, 1), (4, 2), (5, 2), (6, 2), (3, 7), (4, 7), (4, 8), (5, 7), (6, 7), (6, 8) |
| [229437_at](https://www.affymetrix.com/LinkServlet?probeset=229437_at) | [MIR155HG](http://www.ncbi.nlm.nih.gov/entrez/query.fcgi?cmd=search&db=gene&term=MIR155HG) | MIR155 host gene (non-protein coding) | 6e-05 | 0.0176 | 13.83 | 55.23 | 7.1 | 5.86 | 7.5 | 7.8 | 5.63 | 8.8 | (1, 2), (3, 2), (4, 2), (5, 2), (6, 2), (7, 2), (8, 2) |
| [230008_at](https://www.affymetrix.com/LinkServlet?probeset=230008_at) | [THSD7A](http://www.ncbi.nlm.nih.gov/entrez/query.fcgi?cmd=search&db=gene&term=THSD7A) | thrombospondin, type I, domain containing 7A | 6.04e-05 | 0.0176 | 53.37 | 68.41 | 199.53 | 451.83 | 228.86 | 372.25 | 167.55 | 106.5 | (1, 3), (1, 4), (1, 5), (1, 6), (2, 4), (2, 5), (2, 6), (8, 4) |
| [210137_s_at](https://www.affymetrix.com/LinkServlet?probeset=210137_s_at) | [DCTD](http://www.ncbi.nlm.nih.gov/entrez/query.fcgi?cmd=search&db=gene&term=DCTD) | dCMP deaminase | 6.12e-05 | 0.0176 | 266.36 | 244.83 | 114.62 | 138.66 | 193.57 | 173.41 | 221.75 | 207.89 | (3, 1), (4, 1), (3, 2), (4, 2), (3, 5), (3, 7), (3, 8) |
| [203300_x_at](https://www.affymetrix.com/LinkServlet?probeset=203300_x_at) | [AP1S2](http://www.ncbi.nlm.nih.gov/entrez/query.fcgi?cmd=search&db=gene&term=AP1S2) | adaptor-related protein complex 1, sigma 2 subunit | 6.3e-05 | 0.0179 | 1922.95 | 391.28 | 439.95 | 297.8 | 567.11 | 355.8 | 466.9 | 550.97 | (2, 1), (3, 1), (4, 1), (5, 1), (6, 1), (7, 1), (8, 1) |
| [206018_at](https://www.affymetrix.com/LinkServlet?probeset=206018_at) | [FOXG1](http://www.ncbi.nlm.nih.gov/entrez/query.fcgi?cmd=search&db=gene&term=FOXG1) | forkhead box G1 | 6.53e-05 | 0.0183 | 965.5 | 5.94 | 4.75 | 5.59 | 4.75 | 4.75 | 185.23 | 63.1 | (2, 1), (3, 1), (4, 1), (5, 1), (6, 1) |
| [232276_at](https://www.affymetrix.com/LinkServlet?probeset=232276_at) | [HS6ST3](http://www.ncbi.nlm.nih.gov/entrez/query.fcgi?cmd=search&db=gene&term=HS6ST3) | heparan sulfate 6-O-sulfotransferase 3 | 6.8e-05 | 0.0189 | 39.42 | 37.73 | 155.31 | 363.84 | 101.92 | 220.34 | 254.4 | 122.61 | (1, 3), (1, 4), (1, 6), (1, 7), (2, 3), (2, 4), (2, 6), (2, 7) |
| [242319_at](https://www.affymetrix.com/LinkServlet?probeset=242319_at) | [DGKG](http://www.ncbi.nlm.nih.gov/entrez/query.fcgi?cmd=search&db=gene&term=DGKG) | diacylglycerol kinase, gamma 90kDa | 7.16e-05 | 0.0192 | 8.25 | 6.09 | 31.42 | 37.92 | 10.26 | 34.53 | 19.71 | 6.87 | (1, 3), (1, 4), (1, 6), (2, 3), (2, 4), (2, 6), (8, 3), (5, 4), (8, 4), (5, 6), (8, 6) |
| [209310_s_at](https://www.affymetrix.com/LinkServlet?probeset=209310_s_at) | [CASP4](http://www.ncbi.nlm.nih.gov/entrez/query.fcgi?cmd=search&db=gene&term=CASP4) | caspase 4, apoptosis-related cysteine peptidase | 7.19e-05 | 0.0192 | 62.42 | 161.09 | 24 | 31.17 | 76.79 | 38.65 | 102.5 | 76.48 | (1, 2), (3, 2), (4, 2), (6, 2), (3, 5), (3, 7), (3, 8) |
| [230472_at](https://www.affymetrix.com/LinkServlet?probeset=230472_at) | [IRX1](http://www.ncbi.nlm.nih.gov/entrez/query.fcgi?cmd=search&db=gene&term=IRX1) | iroquois homeobox 1 | 7.49e-05 | 0.0192 | 12.12 | 72.26 | 163.12 | 192.2 | 238.45 | 228.5 | 43.6 | 47 | (1, 2), (1, 3), (1, 4), (1, 5), (1, 6) |
| [236828_at](https://www.affymetrix.com/LinkServlet?probeset=236828_at) | [NA](http://www.ncbi.nlm.nih.gov/entrez/query.fcgi?cmd=search&db=gene&term=NA) | NA | 7.58e-05 | 0.0192 | 6.84 | 22.81 | 5.79 | 5.08 | 4.76 | 4.81 | 4.75 | 8.14 | (1, 2), (3, 2), (4, 2), (5, 2), (6, 2), (7, 2), (8, 2) |
| [214920_at](https://www.affymetrix.com/LinkServlet?probeset=214920_at) | [THSD7A](http://www.ncbi.nlm.nih.gov/entrez/query.fcgi?cmd=search&db=gene&term=THSD7A) | thrombospondin, type I, domain containing 7A | 7.74e-05 | 0.0192 | 76.08 | 109.98 | 243.21 | 566.05 | 376.99 | 526.2 | 256.03 | 128.99 | (1, 3), (1, 4), (1, 5), (1, 6), (2, 4), (2, 5), (2, 6), (8, 4), (8, 6) |
| [213752_at](https://www.affymetrix.com/LinkServlet?probeset=213752_at) | [RP1-21O18.1](http://www.ncbi.nlm.nih.gov/entrez/query.fcgi?cmd=search&db=gene&term=RP1-21O18.1) | kazrin | 7.88e-05 | 0.0192 | 64.31 | 92.67 | 204.22 | 221.95 | 172.83 | 250.7 | 247.52 | 131.8 | (1, 3), (1, 4), (1, 5), (1, 6), (1, 7), (2, 3), (2, 4), (2, 6) |
| [223422_s_at](https://www.affymetrix.com/LinkServlet?probeset=223422_s_at) | [ARHGAP24](http://www.ncbi.nlm.nih.gov/entrez/query.fcgi?cmd=search&db=gene&term=ARHGAP24) | Rho GTPase activating protein 24 | 7.9e-05 | 0.0192 | 214.94 | 260.22 | 45.42 | 66.64 | 93.33 | 64.87 | 63.94 | 113.43 | (3, 1), (4, 1), (6, 1), (3, 2), (4, 2), (5, 2), (6, 2), (7, 2) |
| [244868_at](https://www.affymetrix.com/LinkServlet?probeset=244868_at) | [NA](http://www.ncbi.nlm.nih.gov/entrez/query.fcgi?cmd=search&db=gene&term=NA) | NA | 8.03e-05 | 0.0192 | 8.59 | 7.86 | 11.72 | 33.74 | 8.97 | 8.65 | 5.99 | 8.95 | (1, 4), (2, 4), (3, 4), (5, 4), (6, 4), (7, 4), (8, 4) |
| [207231_at](https://www.affymetrix.com/LinkServlet?probeset=207231_at) | [DZIP3](http://www.ncbi.nlm.nih.gov/entrez/query.fcgi?cmd=search&db=gene&term=DZIP3) | DAZ interacting protein 3, zinc finger | 8.05e-05 | 0.0192 | 115.36 | 79.9 | 155.83 | 168.45 | 140.27 | 178.71 | 167.42 | 96.38 | (2, 3), (2, 4), (2, 5), (2, 6), (2, 7), (8, 4), (8, 6) |
| [223842_s_at](https://www.affymetrix.com/LinkServlet?probeset=223842_s_at) | [SCARA3](http://www.ncbi.nlm.nih.gov/entrez/query.fcgi?cmd=search&db=gene&term=SCARA3) | scavenger receptor class A, member 3 | 8.08e-05 | 0.0192 | 30.2 | 21.5 | 48.23 | 76.67 | 111.64 | 119.34 | 34.61 | 57.01 | (1, 5), (1, 6), (2, 4), (2, 5), (2, 6) |
| [226589_at](https://www.affymetrix.com/LinkServlet?probeset=226589_at) | [TMEM192](http://www.ncbi.nlm.nih.gov/entrez/query.fcgi?cmd=search&db=gene&term=TMEM192) | transmembrane protein 192 | 8.1e-05 | 0.0192 | 107.91 | 62.65 | 118.5 | 104.65 | 121.73 | 156.25 | 148.59 | 82.58 | (2, 1), (2, 3), (2, 4), (2, 5), (2, 6), (2, 7), (8, 6) |
| [222787_s_at](https://www.affymetrix.com/LinkServlet?probeset=222787_s_at) | [TMEM106B](http://www.ncbi.nlm.nih.gov/entrez/query.fcgi?cmd=search&db=gene&term=TMEM106B) | transmembrane protein 106B | 8.14e-05 | 0.0192 | 707.46 | 738.75 | 1309.83 | 1495.54 | 874.53 | 1695.88 | 923.62 | 868.71 | (1, 3), (1, 4), (1, 6), (2, 3), (2, 4), (2, 6), (5, 4), (5, 6), (8, 6) |
| [236536_at](https://www.affymetrix.com/LinkServlet?probeset=236536_at) | [GALNT13](http://www.ncbi.nlm.nih.gov/entrez/query.fcgi?cmd=search&db=gene&term=GALNT13) | UDP-N-acetyl-alpha-D-galactosamine:polypeptide N-acetylgalactosaminyltransferase 13 (GalNAc-T13) | 8.21e-05 | 0.0192 | 23.54 | 67.9 | 144.05 | 242.7 | 121.44 | 209.02 | 264.76 | 105.89 | (1, 3), (1, 4), (1, 5), (1, 6), (1, 7), (1, 8), (2, 4) |
| [210239_at](https://www.affymetrix.com/LinkServlet?probeset=210239_at) | [IRX5](http://www.ncbi.nlm.nih.gov/entrez/query.fcgi?cmd=search&db=gene&term=IRX5) | iroquois homeobox 5 | 8.22e-05 | 0.0192 | 9.03 | 21.29 | 93.42 | 105.13 | 102.54 | 91.47 | 38.71 | 29.63 | (1, 3), (1, 4), (1, 5), (1, 6), (2, 3), (2, 4), (2, 5), (2, 6) |
| [1553997_a_at](https://www.affymetrix.com/LinkServlet?probeset=1553997_a_at) | [ASPHD1](http://www.ncbi.nlm.nih.gov/entrez/query.fcgi?cmd=search&db=gene&term=ASPHD1) | aspartate beta-hydroxylase domain containing 1 | 8.22e-05 | 0.0192 | 222.08 | 36.38 | 98.27 | 25.7 | 54.41 | 26.8 | 88.79 | 83.72 | (2, 1), (4, 1), (5, 1), (6, 1), (4, 3), (6, 3) |
| [229163_at](https://www.affymetrix.com/LinkServlet?probeset=229163_at) | [CAMK2N1](http://www.ncbi.nlm.nih.gov/entrez/query.fcgi?cmd=search&db=gene&term=CAMK2N1) | calcium/calmodulin-dependent protein kinase II inhibitor 1 | 8.43e-05 | 0.0194 | 224.03 | 147.84 | 419.81 | 339.41 | 273.38 | 393.27 | 226.75 | 185.76 | (1, 3), (2, 3), (2, 4), (2, 5), (2, 6), (8, 3), (8, 6) |
| [229890_at](https://www.affymetrix.com/LinkServlet?probeset=229890_at) | [PRRT1](http://www.ncbi.nlm.nih.gov/entrez/query.fcgi?cmd=search&db=gene&term=PRRT1) | proline-rich transmembrane protein 1 | 8.79e-05 | 0.0201 | 218.35 | 68.73 | 341.99 | 168.36 | 198.86 | 199.06 | 450.87 | 169.26 | (2, 1), (2, 3), (2, 4), (2, 5), (2, 6), (2, 7), (2, 8) |
| [204352_at](https://www.affymetrix.com/LinkServlet?probeset=204352_at) | [TRAF5](http://www.ncbi.nlm.nih.gov/entrez/query.fcgi?cmd=search&db=gene&term=TRAF5) | TNF receptor-associated factor 5 | 9e-05 | 0.0201 | 52.03 | 160.97 | 44.88 | 64.02 | 78.16 | 55.86 | 61.32 | 66.68 | (1, 2), (3, 2), (4, 2), (5, 2), (6, 2), (7, 2), (8, 2) |
| [219687_at](https://www.affymetrix.com/LinkServlet?probeset=219687_at) | [HHAT](http://www.ncbi.nlm.nih.gov/entrez/query.fcgi?cmd=search&db=gene&term=HHAT) | hedgehog acyltransferase | 9.01e-05 | 0.0201 | 19.01 | 10.62 | 12.15 | 16.78 | 16.4 | 14.77 | 35.5 | 13.24 | (2, 1), (3, 1), (1, 7), (2, 4), (2, 5), (2, 7), (3, 7), (4, 7), (5, 7), (6, 7), (8, 7) |
| [227481_at](https://www.affymetrix.com/LinkServlet?probeset=227481_at) | [CNKSR3](http://www.ncbi.nlm.nih.gov/entrez/query.fcgi?cmd=search&db=gene&term=CNKSR3) | CNKSR family member 3 | 9.26e-05 | 0.0203 | 190.02 | 201.85 | 302.5 | 461.17 | 363.34 | 476.77 | 443.07 | 236.01 | (1, 4), (1, 5), (1, 6), (1, 7), (2, 4), (2, 5), (2, 6), (8, 4), (8, 6) |
| [220104_at](https://www.affymetrix.com/LinkServlet?probeset=220104_at) | [ZC3HAV1](http://www.ncbi.nlm.nih.gov/entrez/query.fcgi?cmd=search&db=gene&term=ZC3HAV1) | zinc finger CCCH-type, antiviral 1 | 9.27e-05 | 0.0203 | 25.34 | 47.51 | 31.63 | 29.29 | 52.69 | 47.4 | 10.43 | 27.98 | (1, 2), (1, 5), (1, 6), (7, 1), (7, 2), (7, 3), (7, 4), (7, 5), (7, 6), (7, 8) |
| [210260_s_at](https://www.affymetrix.com/LinkServlet?probeset=210260_s_at) | [TNFAIP8](http://www.ncbi.nlm.nih.gov/entrez/query.fcgi?cmd=search&db=gene&term=TNFAIP8) | tumor necrosis factor, alpha-induced protein 8 | 9.37e-05 | 0.0203 | 142.32 | 255.51 | 49.08 | 61.28 | 150.29 | 81.05 | 54.24 | 135.57 | (3, 1), (3, 2), (4, 2), (6, 2), (7, 2), (3, 5), (3, 8) |
| [226237_at](https://www.affymetrix.com/LinkServlet?probeset=226237_at) | [NA](http://www.ncbi.nlm.nih.gov/entrez/query.fcgi?cmd=search&db=gene&term=NA) | NA | 9.5e-05 | 0.0204 | 500.35 | 1796.95 | 344.51 | 341.84 | 646.61 | 749.21 | 31.25 | 1119.13 | (1, 2), (7, 1), (3, 2), (4, 2), (7, 2), (7, 3), (7, 4), (7, 5), (7, 6), (7, 8) |
| [221030_s_at](https://www.affymetrix.com/LinkServlet?probeset=221030_s_at) | [ARHGAP24](http://www.ncbi.nlm.nih.gov/entrez/query.fcgi?cmd=search&db=gene&term=ARHGAP24) | Rho GTPase activating protein 24 | 9.68e-05 | 0.0206 | 35.12 | 52.62 | 12.52 | 17.82 | 17.93 | 16.16 | 15.45 | 25.04 | (3, 1), (6, 1), (3, 2), (4, 2), (5, 2), (6, 2), (7, 2) |
| [231223_at](https://www.affymetrix.com/LinkServlet?probeset=231223_at) | [CSMD1](http://www.ncbi.nlm.nih.gov/entrez/query.fcgi?cmd=search&db=gene&term=CSMD1) | CUB and Sushi multiple domains 1 | 9.84e-05 | 0.0207 | 80.54 | 150.47 | 354.95 | 611.87 | 286.22 | 509.85 | 261.33 | 123.72 | (1, 3), (1, 4), (1, 5), (1, 6), (2, 4), (2, 6), (8, 4), (8, 6) |
| [239525_at](https://www.affymetrix.com/LinkServlet?probeset=239525_at) | [CTTNBP2NL](http://www.ncbi.nlm.nih.gov/entrez/query.fcgi?cmd=search&db=gene&term=CTTNBP2NL) | CTTNBP2 N-terminal like | 0.000104 | 0.0212 | 77.61 | 155.56 | 86.46 | 116.56 | 95.81 | 113.36 | 61.46 | 88.57 | (1, 2), (1, 4), (3, 2), (5, 2), (7, 2), (8, 2), (7, 4), (7, 6) |
| [208025_s_at](https://www.affymetrix.com/LinkServlet?probeset=208025_s_at) | [HMGA2](http://www.ncbi.nlm.nih.gov/entrez/query.fcgi?cmd=search&db=gene&term=HMGA2) | high mobility group AT-hook 2 | 0.0001042 | 0.0212 | 7.79 | 58.95 | 5.22 | 6.28 | 11.35 | 7.31 | 4.95 | 8.02 | (1, 2), (3, 2), (4, 2), (5, 2), (6, 2), (7, 2), (8, 2) |
| [208017_s_at](https://www.affymetrix.com/LinkServlet?probeset=208017_s_at) | [MCF2](http://www.ncbi.nlm.nih.gov/entrez/query.fcgi?cmd=search&db=gene&term=MCF2) | MCF.2 cell line derived transforming sequence | 0.0001058 | 0.0212 | 16.69 | 28.3 | 166.73 | 186.4 | 102.45 | 182.43 | 59.82 | 70.45 | (1, 3), (1, 4), (1, 5), (1, 6), (2, 3), (2, 4), (2, 6) |
| [225018_at](https://www.affymetrix.com/LinkServlet?probeset=225018_at) | [SPIRE1](http://www.ncbi.nlm.nih.gov/entrez/query.fcgi?cmd=search&db=gene&term=SPIRE1) | spire homolog 1 (Drosophila) | 0.0001071 | 0.0212 | 266.14 | 162.25 | 284.68 | 280.4 | 301.41 | 281.44 | 548.59 | 222.4 | (2, 1), (1, 7), (2, 3), (2, 4), (2, 5), (2, 6), (2, 7), (3, 7), (4, 7), (6, 7), (8, 7) |
| [212448_at](https://www.affymetrix.com/LinkServlet?probeset=212448_at) | [NEDD4L](http://www.ncbi.nlm.nih.gov/entrez/query.fcgi?cmd=search&db=gene&term=NEDD4L) | neural precursor cell expressed, developmentally down-regulated 4-like | 0.0001075 | 0.0212 | 194.97 | 19.83 | 34.98 | 23.35 | 29.06 | 17.03 | 205.99 | 47.76 | (2, 1), (3, 1), (4, 1), (5, 1), (6, 1), (2, 7), (4, 7), (6, 7) |
| [205528_s_at](https://www.affymetrix.com/LinkServlet?probeset=205528_s_at) | [RUNX1T1](http://www.ncbi.nlm.nih.gov/entrez/query.fcgi?cmd=search&db=gene&term=RUNX1T1) | runt-related transcription factor 1; translocated to, 1 (cyclin D-related) | 0.000108 | 0.0212 | 72.59 | 74.05 | 235.25 | 265.15 | 172.3 | 222.21 | 269.58 | 144.37 | (1, 3), (1, 4), (1, 5), (1, 6), (1, 7), (2, 3), (2, 4), (2, 5), (2, 6), (2, 7) |
| [230482_at](https://www.affymetrix.com/LinkServlet?probeset=230482_at) | [ST6GALNAC5](http://www.ncbi.nlm.nih.gov/entrez/query.fcgi?cmd=search&db=gene&term=ST6GALNAC5) | ST6 (alpha-N-acetyl-neuraminyl-2,3-beta-galactosyl-1,3)-N-acetylgalactosaminide alpha-2,6-sialyltransferase 5 | 0.0001089 | 0.0212 | 10.69 | 4.75 | 4.85 | 4.75 | 4.77 | 4.75 | 5.28 | 5.1 | (2, 1), (3, 1), (4, 1), (5, 1), (6, 1), (7, 1), (8, 1) |
| [209789_at](https://www.affymetrix.com/LinkServlet?probeset=209789_at) | [CORO2B](http://www.ncbi.nlm.nih.gov/entrez/query.fcgi?cmd=search&db=gene&term=CORO2B) | coronin, actin binding protein, 2B | 0.0001097 | 0.0212 | 1092.34 | 266.01 | 417.34 | 152.99 | 268.94 | 183.66 | 1736.74 | 509.12 | (2, 1), (4, 1), (5, 1), (6, 1), (2, 7), (4, 7), (5, 7), (6, 7) |
| [243932_at](https://www.affymetrix.com/LinkServlet?probeset=243932_at) | [NA](http://www.ncbi.nlm.nih.gov/entrez/query.fcgi?cmd=search&db=gene&term=NA) | NA | 0.0001098 | 0.0212 | 7.5 | 14.26 | 94.4 | 63.06 | 40.8 | 50.21 | 22.98 | 18.6 | (1, 3), (1, 4), (1, 5), (1, 6), (2, 3), (2, 4), (8, 3) |
| [232037_at](https://www.affymetrix.com/LinkServlet?probeset=232037_at) | [IGDCC3](http://www.ncbi.nlm.nih.gov/entrez/query.fcgi?cmd=search&db=gene&term=IGDCC3) | immunoglobulin superfamily, DCC subclass, member 3 | 0.0001104 | 0.0212 | 77.17 | 13.89 | 33.53 | 41.58 | 23.15 | 28.89 | 112.49 | 30.34 | (2, 1), (5, 1), (6, 1), (2, 4), (2, 7), (5, 7), (6, 7) |
| [1554807_a_at](https://www.affymetrix.com/LinkServlet?probeset=1554807_a_at) | [SPIRE1](http://www.ncbi.nlm.nih.gov/entrez/query.fcgi?cmd=search&db=gene&term=SPIRE1) | spire homolog 1 (Drosophila) | 0.0001177 | 0.0224 | 69.11 | 43.89 | 60.61 | 54.36 | 78.9 | 77.09 | 168.36 | 53.99 | (2, 1), (1, 7), (2, 5), (2, 6), (2, 7), (3, 7), (4, 7), (5, 7), (6, 7), (8, 7) |
| [240312_at](https://www.affymetrix.com/LinkServlet?probeset=240312_at) | [LOC389895](http://www.ncbi.nlm.nih.gov/entrez/query.fcgi?cmd=search&db=gene&term=LOC389895) | hypothetical LOC389895 | 0.0001196 | 0.0225 | 10.04 | 6.84 | 18.2 | 42.14 | 29.53 | 31.74 | 21.5 | 17.68 | (1, 4), (1, 5), (1, 6), (2, 3), (2, 4), (2, 5), (2, 6) |
| [219537_x_at](https://www.affymetrix.com/LinkServlet?probeset=219537_x_at) | [DLL3](http://www.ncbi.nlm.nih.gov/entrez/query.fcgi?cmd=search&db=gene&term=DLL3) | delta-like 3 (Drosophila) | 0.000121 | 0.0225 | 17.16 | 20.09 | 38.19 | 98.14 | 86.81 | 147.58 | 40.89 | 22.19 | (1, 4), (1, 5), (1, 6), (2, 4), (2, 5), (2, 6), (3, 6), (8, 4), (8, 6) |
| [1570255_s_at](https://www.affymetrix.com/LinkServlet?probeset=1570255_s_at) | [NA](http://www.ncbi.nlm.nih.gov/entrez/query.fcgi?cmd=search&db=gene&term=NA) | NA | 0.000122 | 0.0225 | 11.83 | 63.23 | 49.36 | 67.73 | 35.11 | 89.57 | 21.06 | 24.62 | (1, 2), (1, 3), (1, 4), (1, 5), (1, 6), (8, 6) |
| [206302_s_at](https://www.affymetrix.com/LinkServlet?probeset=206302_s_at) | [NA](http://www.ncbi.nlm.nih.gov/entrez/query.fcgi?cmd=search&db=gene&term=NA) | NA | 0.0001223 | 0.0225 | 647.92 | 1350.09 | 339.27 | 548.2 | 721.64 | 667.63 | 183.53 | 602.97 | (1, 2), (7, 1), (3, 2), (4, 2), (7, 2), (8, 2), (7, 5), (7, 6), (7, 8) |
| [213015_at](https://www.affymetrix.com/LinkServlet?probeset=213015_at) | [BBX](http://www.ncbi.nlm.nih.gov/entrez/query.fcgi?cmd=search&db=gene&term=BBX) | bobby sox homolog (Drosophila) | 0.0001234 | 0.0225 | 425.07 | 276.57 | 466.33 | 622.78 | 459.13 | 603.81 | 487.67 | 307.65 | (2, 1), (2, 3), (2, 4), (2, 5), (2, 6), (8, 4), (8, 6) |
| [206622_at](https://www.affymetrix.com/LinkServlet?probeset=206622_at) | [TRH](http://www.ncbi.nlm.nih.gov/entrez/query.fcgi?cmd=search&db=gene&term=TRH) | thyrotropin-releasing hormone | 0.00013 | 0.0235 | 8.52 | 15.49 | 20.65 | 68.21 | 62.59 | 205.95 | 7.09 | 7.36 | (1, 4), (1, 5), (1, 6), (2, 6), (3, 6), (8, 4), (8, 5), (7, 6), (8, 6) |
| [203917_at](https://www.affymetrix.com/LinkServlet?probeset=203917_at) | [CXADR](http://www.ncbi.nlm.nih.gov/entrez/query.fcgi?cmd=search&db=gene&term=CXADR) | coxsackie virus and adenovirus receptor | 0.0001377 | 0.0247 | 4100.88 | 1162.29 | 1444.07 | 980.93 | 1296.95 | 1758.64 | 2011.74 | 1930.53 | (2, 1), (3, 1), (4, 1), (5, 1), (6, 1) |
| [205498_at](https://www.affymetrix.com/LinkServlet?probeset=205498_at) | [GHR](http://www.ncbi.nlm.nih.gov/entrez/query.fcgi?cmd=search&db=gene&term=GHR) | growth hormone receptor | 0.0001441 | 0.0254 | 42.59 | 222.35 | 132.07 | 178.71 | 132.68 | 209.09 | 67.78 | 96.19 | (1, 2), (1, 3), (1, 4), (1, 5), (1, 6) |
| [1562648_at](https://www.affymetrix.com/LinkServlet?probeset=1562648_at) | [CCDC88A](http://www.ncbi.nlm.nih.gov/entrez/query.fcgi?cmd=search&db=gene&term=CCDC88A) | coiled-coil domain containing 88A | 0.0001464 | 0.0254 | 6.84 | 7.01 | 7.75 | 9.82 | 8.21 | 15.15 | 5.92 | 7.15 | (1, 6), (2, 6), (3, 6), (5, 6), (7, 6), (8, 6) |
| [204689_at](https://www.affymetrix.com/LinkServlet?probeset=204689_at) | [HHEX](http://www.ncbi.nlm.nih.gov/entrez/query.fcgi?cmd=search&db=gene&term=HHEX) | hematopoietically expressed homeobox | 0.0001465 | 0.0254 | 36.7 | 68.69 | 18.8 | 29.04 | 33.55 | 32.41 | 29.06 | 52.66 | (1, 2), (3, 1), (3, 2), (4, 2), (5, 2), (6, 2), (3, 8) |
| [206693_at](https://www.affymetrix.com/LinkServlet?probeset=206693_at) | [IL7](http://www.ncbi.nlm.nih.gov/entrez/query.fcgi?cmd=search&db=gene&term=IL7) | interleukin 7 | 0.0001469 | 0.0254 | 8.41 | 41.01 | 9.32 | 9.74 | 13.66 | 15.87 | 6.35 | 16.28 | (1, 2), (3, 2), (4, 2), (5, 2), (6, 2), (7, 2) |
| [243139_at](https://www.affymetrix.com/LinkServlet?probeset=243139_at) | [NA](http://www.ncbi.nlm.nih.gov/entrez/query.fcgi?cmd=search&db=gene&term=NA) | NA | 0.0001473 | 0.0254 | 9.26 | 12.94 | 60.71 | 41.71 | 25.17 | 52.16 | 6.89 | 18.79 | (1, 3), (1, 4), (1, 6), (2, 3), (2, 4), (2, 6), (7, 3), (7, 4), (7, 6) |
| [205240_at](https://www.affymetrix.com/LinkServlet?probeset=205240_at) | [GPSM2](http://www.ncbi.nlm.nih.gov/entrez/query.fcgi?cmd=search&db=gene&term=GPSM2) | G-protein signaling modulator 2 (AGS3-like, C. elegans) | 0.0001513 | 0.0259 | 72.89 | 118.18 | 138.04 | 210.86 | 213.98 | 262.45 | 174.51 | 107.3 | (1, 4), (1, 5), (1, 6), (2, 6), (8, 6) |
| [266_s_at](https://www.affymetrix.com/LinkServlet?probeset=266_s_at) | [CD24](http://www.ncbi.nlm.nih.gov/entrez/query.fcgi?cmd=search&db=gene&term=CD24) | CD24 molecule | 0.0001558 | 0.0265 | 86.52 | 7.67 | 11.33 | 11.96 | 9.52 | 6.74 | 19.35 | 73.04 | (2, 1), (3, 1), (4, 1), (5, 1), (6, 1), (2, 8), (3, 8), (4, 8), (5, 8), (6, 8) |
| [227282_at](https://www.affymetrix.com/LinkServlet?probeset=227282_at) | [PCDH19](http://www.ncbi.nlm.nih.gov/entrez/query.fcgi?cmd=search&db=gene&term=PCDH19) | protocadherin 19 | 0.0001595 | 0.0269 | 97.47 | 117.9 | 341.32 | 642.05 | 394.47 | 740.99 | 351.01 | 272.93 | (1, 3), (1, 4), (1, 5), (1, 6), (2, 4), (2, 5), (2, 6) |
| [231790_at](https://www.affymetrix.com/LinkServlet?probeset=231790_at) | [DMGDH](http://www.ncbi.nlm.nih.gov/entrez/query.fcgi?cmd=search&db=gene&term=DMGDH) | dimethylglycine dehydrogenase | 0.0001628 | 0.0271 | 12.21 | 9.04 | 8.47 | 9.38 | 8.05 | 7.38 | 22.34 | 9.47 | (5, 1), (6, 1), (1, 7), (2, 7), (3, 7), (4, 7), (5, 7), (6, 7), (8, 7) |
| [1552263_at](https://www.affymetrix.com/LinkServlet?probeset=1552263_at) | [MAPK1](http://www.ncbi.nlm.nih.gov/entrez/query.fcgi?cmd=search&db=gene&term=MAPK1) | mitogen-activated protein kinase 1 | 0.0001665 | 0.0271 | 38.91 | 62.58 | 19.44 | 22.52 | 36.8 | 30.42 | 18.45 | 33.82 | (3, 1), (3, 2), (4, 2), (6, 2), (7, 2) |
| [243061_at](https://www.affymetrix.com/LinkServlet?probeset=243061_at) | [C14orf23](http://www.ncbi.nlm.nih.gov/entrez/query.fcgi?cmd=search&db=gene&term=C14orf23) | chromosome 14 open reading frame 23 | 0.0001666 | 0.0271 | 18.64 | 4.75 | 4.75 | 4.75 | 4.75 | 4.75 | 8.13 | 6.03 | (2, 1), (3, 1), (4, 1), (5, 1), (6, 1), (8, 1) |
| [203789_s_at](https://www.affymetrix.com/LinkServlet?probeset=203789_s_at) | [SEMA3C](http://www.ncbi.nlm.nih.gov/entrez/query.fcgi?cmd=search&db=gene&term=SEMA3C) | sema domain, immunoglobulin domain (Ig), short basic domain, secreted, (semaphorin) 3C | 0.0001683 | 0.0271 | 310.25 | 481.6 | 107.82 | 17.94 | 105.03 | 83.71 | 33.68 | 229.07 | (4, 1), (4, 2), (6, 2), (7, 2), (4, 3), (4, 5), (4, 8) |
| [244420_at](https://www.affymetrix.com/LinkServlet?probeset=244420_at) | [NA](http://www.ncbi.nlm.nih.gov/entrez/query.fcgi?cmd=search&db=gene&term=NA) | NA | 0.000169 | 0.0271 | 10.68 | 8.59 | 60.74 | 84.8 | 22.69 | 38.79 | 25.61 | 21.99 | (1, 3), (1, 4), (2, 3), (2, 4), (2, 6) |
| [57588_at](https://www.affymetrix.com/LinkServlet?probeset=57588_at) | [SLC24A3](http://www.ncbi.nlm.nih.gov/entrez/query.fcgi?cmd=search&db=gene&term=SLC24A3) | solute carrier family 24 (sodium/potassium/calcium exchanger), member 3 | 0.0001694 | 0.0271 | 83.39 | 96.34 | 325.42 | 362.49 | 352.15 | 444.81 | 524.85 | 223.86 | (1, 3), (1, 4), (1, 5), (1, 6), (1, 7), (2, 3), (2, 4), (2, 5), (2, 6), (2, 7) |
| [204897_at](https://www.affymetrix.com/LinkServlet?probeset=204897_at) | [PTGER4](http://www.ncbi.nlm.nih.gov/entrez/query.fcgi?cmd=search&db=gene&term=PTGER4) | prostaglandin E receptor 4 (subtype EP4) | 0.0001696 | 0.0271 | 445.16 | 733.76 | 124.47 | 158.34 | 453.82 | 225.06 | 107.11 | 757.75 | (3, 1), (3, 2), (4, 2), (6, 2), (7, 2), (3, 5), (3, 8), (4, 8), (7, 8) |
| [224995_at](https://www.affymetrix.com/LinkServlet?probeset=224995_at) | [SPIRE1](http://www.ncbi.nlm.nih.gov/entrez/query.fcgi?cmd=search&db=gene&term=SPIRE1) | spire homolog 1 (Drosophila) | 0.0001808 | 0.0282 | 1797.72 | 1075.65 | 2014.99 | 2001.56 | 1703.95 | 1900.42 | 3641.43 | 1501.36 | (2, 1), (1, 7), (2, 3), (2, 4), (2, 5), (2, 6), (2, 7), (5, 7), (6, 7), (8, 7) |
| [226197_at](https://www.affymetrix.com/LinkServlet?probeset=226197_at) | [NA](http://www.ncbi.nlm.nih.gov/entrez/query.fcgi?cmd=search&db=gene&term=NA) | NA | 0.0001813 | 0.0282 | 37.07 | 209.99 | 100.25 | 153.46 | 124.23 | 164.35 | 58.52 | 158.72 | (1, 2), (1, 3), (1, 4), (1, 5), (1, 6), (1, 8) |
| [204513_s_at](https://www.affymetrix.com/LinkServlet?probeset=204513_s_at) | [ELMO1](http://www.ncbi.nlm.nih.gov/entrez/query.fcgi?cmd=search&db=gene&term=ELMO1) | engulfment and cell motility 1 | 0.0001814 | 0.0282 | 264.81 | 91.8 | 290.18 | 250.67 | 274.65 | 279.89 | 333.75 | 215.11 | (2, 1), (2, 3), (2, 4), (2, 5), (2, 6), (2, 7), (2, 8) |
| [235591_at](https://www.affymetrix.com/LinkServlet?probeset=235591_at) | [SSTR1](http://www.ncbi.nlm.nih.gov/entrez/query.fcgi?cmd=search&db=gene&term=SSTR1) | somatostatin receptor 1 | 0.0001816 | 0.0282 | 27.7 | 21.26 | 103.58 | 333.08 | 137.69 | 400.22 | 176.18 | 64.8 | (1, 4), (1, 6), (2, 4), (2, 5), (2, 6) |
| [204068_at](https://www.affymetrix.com/LinkServlet?probeset=204068_at) | [STK3](http://www.ncbi.nlm.nih.gov/entrez/query.fcgi?cmd=search&db=gene&term=STK3) | serine/threonine kinase 3 (STE20 homolog, yeast) | 0.0001861 | 0.0287 | 324.09 | 483.28 | 232.54 | 237.63 | 256.11 | 284.85 | 203.32 | 365.37 | (3, 2), (4, 2), (5, 2), (6, 2), (7, 2) |
| [221044_s_at](https://www.affymetrix.com/LinkServlet?probeset=221044_s_at) | [NA](http://www.ncbi.nlm.nih.gov/entrez/query.fcgi?cmd=search&db=gene&term=NA) | NA | 0.0001928 | 0.0295 | 72.28 | 114.86 | 29.81 | 30.9 | 57.78 | 46.69 | 55.68 | 80.38 | (3, 1), (4, 1), (3, 2), (4, 2), (6, 2), (3, 8), (4, 8) |
| [210347_s_at](https://www.affymetrix.com/LinkServlet?probeset=210347_s_at) | [BCL11A](http://www.ncbi.nlm.nih.gov/entrez/query.fcgi?cmd=search&db=gene&term=BCL11A) | B-cell CLL/lymphoma 11A (zinc finger protein) | 0.0002006 | 0.0304 | 42.38 | 26.79 | 6.17 | 7.06 | 8.1 | 5.28 | 13.97 | 8.25 | (3, 1), (4, 1), (5, 1), (6, 1), (8, 1), (3, 2), (4, 2), (6, 2) |
| [230550_at](https://www.affymetrix.com/LinkServlet?probeset=230550_at) | [MS4A6A](http://www.ncbi.nlm.nih.gov/entrez/query.fcgi?cmd=search&db=gene&term=MS4A6A) | membrane-spanning 4-domains, subfamily A, member 6A | 0.0002015 | 0.0304 | 157.07 | 426.32 | 72.42 | 91.33 | 178.97 | 137.37 | 49.7 | 198.56 | (1, 2), (3, 2), (4, 2), (6, 2), (7, 2) |
| [203732_at](https://www.affymetrix.com/LinkServlet?probeset=203732_at) | [TRIP4](http://www.ncbi.nlm.nih.gov/entrez/query.fcgi?cmd=search&db=gene&term=TRIP4) | thyroid hormone receptor interactor 4 | 0.0002044 | 0.0307 | 199.5 | 255.84 | 134.23 | 122.31 | 186.3 | 159.33 | 141.06 | 212.37 | (4, 1), (3, 2), (4, 2), (6, 2), (7, 2), (3, 8), (4, 8) |
| [210985_s_at](https://www.affymetrix.com/LinkServlet?probeset=210985_s_at) | [SP100](http://www.ncbi.nlm.nih.gov/entrez/query.fcgi?cmd=search&db=gene&term=SP100) | SP100 nuclear antigen | 0.000209 | 0.031 | 17.58 | 31.32 | 14.1 | 13.88 | 14.39 | 19.31 | 9.48 | 18.99 | (1, 2), (3, 2), (4, 2), (5, 2), (7, 2) |
| [242054_s_at](https://www.affymetrix.com/LinkServlet?probeset=242054_s_at) | [NA](http://www.ncbi.nlm.nih.gov/entrez/query.fcgi?cmd=search&db=gene&term=NA) | NA | 0.0002097 | 0.031 | 5.08 | 46.72 | 5.15 | 5.03 | 4.75 | 4.75 | 4.75 | 65.69 | (1, 2), (1, 8), (3, 2), (4, 2), (5, 2), (6, 2), (3, 8), (4, 8), (5, 8), (6, 8) |
| [216086_at](https://www.affymetrix.com/LinkServlet?probeset=216086_at) | [SV2C](http://www.ncbi.nlm.nih.gov/entrez/query.fcgi?cmd=search&db=gene&term=SV2C) | synaptic vesicle glycoprotein 2C | 0.0002119 | 0.0312 | 9.24 | 13 | 63.02 | 62.88 | 26.75 | 74.39 | 7.09 | 18.26 | (1, 3), (1, 4), (1, 6), (2, 3), (2, 4), (2, 6), (7, 3), (7, 4), (7, 6) |
| [1560025_at](https://www.affymetrix.com/LinkServlet?probeset=1560025_at) | [NA](http://www.ncbi.nlm.nih.gov/entrez/query.fcgi?cmd=search&db=gene&term=NA) | NA | 0.0002147 | 0.0313 | 32.05 | 129.79 | 149.13 | 264.2 | 70.68 | 274.52 | 55.25 | 66.03 | (1, 2), (1, 3), (1, 4), (1, 6), (5, 4), (8, 4), (5, 6), (8, 6) |
| [228728_at](https://www.affymetrix.com/LinkServlet?probeset=228728_at) | [C7orf58](http://www.ncbi.nlm.nih.gov/entrez/query.fcgi?cmd=search&db=gene&term=C7orf58) | chromosome 7 open reading frame 58 | 0.0002165 | 0.0313 | 376.06 | 631.52 | 133.12 | 212.63 | 205.82 | 197.98 | 110.66 | 321.47 | (3, 1), (3, 2), (4, 2), (5, 2), (6, 2), (7, 2) |
| [229310_at](https://www.affymetrix.com/LinkServlet?probeset=229310_at) | [KLHL29](http://www.ncbi.nlm.nih.gov/entrez/query.fcgi?cmd=search&db=gene&term=KLHL29) | kelch-like 29 (Drosophila) | 0.000217 | 0.0313 | 77.94 | 17.02 | 21.27 | 25.98 | 45.46 | 18.13 | 213.58 | 47 | (2, 1), (3, 1), (6, 1), (2, 7), (3, 7), (4, 7), (6, 7) |
| [229796_at](https://www.affymetrix.com/LinkServlet?probeset=229796_at) | [SIX4](http://www.ncbi.nlm.nih.gov/entrez/query.fcgi?cmd=search&db=gene&term=SIX4) | SIX homeobox 4 | 0.0002202 | 0.0313 | 25.18 | 136.68 | 45 | 45.58 | 43.55 | 62.85 | 11.41 | 29.81 | (1, 2), (3, 2), (4, 2), (5, 2), (7, 2), (8, 2), (7, 6) |
| [236373_at](https://www.affymetrix.com/LinkServlet?probeset=236373_at) | [NA](http://www.ncbi.nlm.nih.gov/entrez/query.fcgi?cmd=search&db=gene&term=NA) | NA | 0.0002218 | 0.0313 | 28.16 | 31.97 | 98.82 | 201.38 | 227.76 | 359.44 | 33.57 | 138.25 | (1, 4), (1, 5), (1, 6), (2, 4), (2, 5), (2, 6), (7, 6) |
| [230561_s_at](https://www.affymetrix.com/LinkServlet?probeset=230561_s_at) | [NA](http://www.ncbi.nlm.nih.gov/entrez/query.fcgi?cmd=search&db=gene&term=NA) | NA | 0.0002246 | 0.0313 | 131.64 | 385.08 | 296 | 417.11 | 289.56 | 479.86 | 312.09 | 240.62 | (1, 2), (1, 3), (1, 4), (1, 5), (1, 6) |
| [203819_s_at](https://www.affymetrix.com/LinkServlet?probeset=203819_s_at) | [NA](http://www.ncbi.nlm.nih.gov/entrez/query.fcgi?cmd=search&db=gene&term=NA) | NA | 0.0002248 | 0.0313 | 28.58 | 461.57 | 14.78 | 31.52 | 35.41 | 30.53 | 28.62 | 58.24 | (1, 2), (3, 2), (4, 2), (5, 2), (6, 2), (7, 2), (8, 2) |
| [1552689_at](https://www.affymetrix.com/LinkServlet?probeset=1552689_at) | [CASKIN1](http://www.ncbi.nlm.nih.gov/entrez/query.fcgi?cmd=search&db=gene&term=CASKIN1) | CASK interacting protein 1 | 0.0002256 | 0.0313 | 104.04 | 49.6 | 196.73 | 211.29 | 108.03 | 165.99 | 717.85 | 122.38 | (1, 7), (2, 3), (2, 4), (2, 6), (2, 7), (5, 7), (8, 7) |
| [203299_s_at](https://www.affymetrix.com/LinkServlet?probeset=203299_s_at) | [AP1S2](http://www.ncbi.nlm.nih.gov/entrez/query.fcgi?cmd=search&db=gene&term=AP1S2) | adaptor-related protein complex 1, sigma 2 subunit | 0.000226 | 0.0313 | 1359.64 | 512.1 | 489.11 | 316.89 | 409.78 | 427.33 | 447.68 | 650.32 | (2, 1), (3, 1), (4, 1), (5, 1), (6, 1), (7, 1) |
| [219197_s_at](https://www.affymetrix.com/LinkServlet?probeset=219197_s_at) | [SCUBE2](http://www.ncbi.nlm.nih.gov/entrez/query.fcgi?cmd=search&db=gene&term=SCUBE2) | signal peptide, CUB domain, EGF-like 2 | 0.0002347 | 0.0318 | 35.97 | 47.19 | 159.77 | 158.33 | 178.63 | 234.9 | 241.92 | 46.55 | (1, 3), (1, 4), (1, 5), (1, 6), (1, 7), (2, 3), (2, 4), (2, 5), (2, 6), (8, 5), (8, 6) |
| [229459_at](https://www.affymetrix.com/LinkServlet?probeset=229459_at) | [FAM19A5](http://www.ncbi.nlm.nih.gov/entrez/query.fcgi?cmd=search&db=gene&term=FAM19A5) | family with sequence similarity 19 (chemokine (C-C motif)-like), member A5 | 0.0002367 | 0.0318 | 884.48 | 308.74 | 1321.71 | 1150.1 | 811.45 | 971.7 | 1493.61 | 571.16 | (2, 1), (2, 3), (2, 4), (2, 5), (2, 6), (2, 7) |
| [228737_at](https://www.affymetrix.com/LinkServlet?probeset=228737_at) | [TOX2](http://www.ncbi.nlm.nih.gov/entrez/query.fcgi?cmd=search&db=gene&term=TOX2) | TOX high mobility group box family member 2 | 0.0002369 | 0.0318 | 14.7 | 69.43 | 79.75 | 70.56 | 81.65 | 85.08 | 51.76 | 60.41 | (1, 2), (1, 3), (1, 4), (1, 5), (1, 6), (1, 8) |
| [212239_at](https://www.affymetrix.com/LinkServlet?probeset=212239_at) | [PIK3R1](http://www.ncbi.nlm.nih.gov/entrez/query.fcgi?cmd=search&db=gene&term=PIK3R1) | phosphoinositide-3-kinase, regulatory subunit 1 (alpha) | 0.0002386 | 0.0318 | 2421.85 | 3025.95 | 4296.27 | 5539.21 | 4046.67 | 5561.82 | 4063.54 | 2674.99 | (1, 3), (1, 4), (1, 5), (1, 6), (2, 4), (2, 6), (8, 4), (8, 6) |
| [233815_at](https://www.affymetrix.com/LinkServlet?probeset=233815_at) | [NAALAD2](http://www.ncbi.nlm.nih.gov/entrez/query.fcgi?cmd=search&db=gene&term=NAALAD2) | N-acetylated alpha-linked acidic dipeptidase 2 | 0.0002405 | 0.0318 | 5.09 | 4.75 | 6.65 | 17.23 | 6.55 | 8.51 | 4.75 | 4.78 | (1, 4), (2, 4), (3, 4), (5, 4), (7, 4), (8, 4) |
| [243879_at](https://www.affymetrix.com/LinkServlet?probeset=243879_at) | [NA](http://www.ncbi.nlm.nih.gov/entrez/query.fcgi?cmd=search&db=gene&term=NA) | NA | 0.0002415 | 0.0318 | 37.47 | 87.24 | 243.59 | 234.1 | 183.61 | 252.88 | 205.04 | 89.49 | (1, 3), (1, 4), (1, 5), (1, 6), (1, 7) |
| [221773_at](https://www.affymetrix.com/LinkServlet?probeset=221773_at) | [ELK3](http://www.ncbi.nlm.nih.gov/entrez/query.fcgi?cmd=search&db=gene&term=ELK3) | ELK3, ETS-domain protein (SRF accessory protein 2) | 0.0002425 | 0.0318 | 1276.57 | 2407.72 | 923.32 | 1011.99 | 1291.5 | 1508.37 | 1031.77 | 1693.54 | (1, 2), (3, 2), (4, 2), (5, 2), (7, 2), (3, 8) |
| [235236_at](https://www.affymetrix.com/LinkServlet?probeset=235236_at) | [LOC100131897](http://www.ncbi.nlm.nih.gov/entrez/query.fcgi?cmd=search&db=gene&term=LOC100131897) | Uncharacterized protein LOC100131897 | 0.0002425 | 0.0318 | 42.82 | 14.26 | 159.04 | 169.59 | 93.69 | 172.64 | 422.32 | 36.2 | (2, 3), (2, 4), (2, 5), (2, 6), (2, 7) |
| [203870_at](https://www.affymetrix.com/LinkServlet?probeset=203870_at) | [USP46](http://www.ncbi.nlm.nih.gov/entrez/query.fcgi?cmd=search&db=gene&term=USP46) | ubiquitin specific peptidase 46 | 0.0002427 | 0.0318 | 1964.86 | 1377.62 | 1865.17 | 2745.6 | 2068.84 | 2267.88 | 3761.4 | 2513.58 | (1, 7), (2, 4), (2, 5), (2, 6), (2, 7), (2, 8), (3, 7) |
| [202949_s_at](https://www.affymetrix.com/LinkServlet?probeset=202949_s_at) | [FHL2](http://www.ncbi.nlm.nih.gov/entrez/query.fcgi?cmd=search&db=gene&term=FHL2) | four and a half LIM domains 2 | 0.0002485 | 0.0324 | 161.36 | 239.47 | 35.45 | 35.18 | 97.03 | 55.25 | 10.44 | 45.38 | (3, 1), (4, 1), (7, 1), (3, 2), (4, 2), (6, 2), (7, 2), (8, 2), (7, 5) |
| [204042_at](https://www.affymetrix.com/LinkServlet?probeset=204042_at) | [WASF3](http://www.ncbi.nlm.nih.gov/entrez/query.fcgi?cmd=search&db=gene&term=WASF3) | WAS protein family, member 3 | 0.0002532 | 0.0328 | 529.7 | 263.18 | 848.07 | 1194.52 | 768.63 | 647.51 | 2510.62 | 835.25 | (1, 7), (2, 3), (2, 4), (2, 5), (2, 6), (2, 7), (2, 8), (6, 7) |
| [219497_s_at](https://www.affymetrix.com/LinkServlet?probeset=219497_s_at) | [BCL11A](http://www.ncbi.nlm.nih.gov/entrez/query.fcgi?cmd=search&db=gene&term=BCL11A) | B-cell CLL/lymphoma 11A (zinc finger protein) | 0.0002586 | 0.0333 | 27.31 | 30.75 | 5.9 | 7.45 | 8.22 | 5.47 | 7.4 | 7.19 | (3, 1), (4, 1), (5, 1), (6, 1), (8, 1), (3, 2), (4, 2), (5, 2), (6, 2), (8, 2) |
| [228010_at](https://www.affymetrix.com/LinkServlet?probeset=228010_at) | [PPP2R2C](http://www.ncbi.nlm.nih.gov/entrez/query.fcgi?cmd=search&db=gene&term=PPP2R2C) | protein phosphatase 2 (formerly 2A), regulatory subunit B, gamma isoform | 0.0002621 | 0.0336 | 114.98 | 6.74 | 20.33 | 10.05 | 16.04 | 6.86 | 480.89 | 23.3 | (2, 1), (4, 1), (5, 1), (6, 1), (2, 7), (3, 7), (4, 7), (5, 7), (6, 7), (8, 7) |
| [237094_at](https://www.affymetrix.com/LinkServlet?probeset=237094_at) | [FAM19A5](http://www.ncbi.nlm.nih.gov/entrez/query.fcgi?cmd=search&db=gene&term=FAM19A5) | family with sequence similarity 19 (chemokine (C-C motif)-like), member A5 | 0.000264 | 0.0336 | 286.42 | 97.86 | 456.73 | 466.87 | 265.7 | 321.93 | 538.04 | 222.58 | (2, 1), (2, 3), (2, 4), (2, 5), (2, 6), (2, 7) |
| [205858_at](https://www.affymetrix.com/LinkServlet?probeset=205858_at) | [NGFR](http://www.ncbi.nlm.nih.gov/entrez/query.fcgi?cmd=search&db=gene&term=NGFR) | nerve growth factor receptor (TNFR superfamily, member 16) | 0.0002653 | 0.0336 | 142.48 | 54.77 | 27.45 | 11.1 | 14.17 | 13.2 | 50.57 | 32.45 | (3, 1), (4, 1), (5, 1), (6, 1), (4, 2), (6, 2) |
| [203126_at](https://www.affymetrix.com/LinkServlet?probeset=203126_at) | [IMPA2](http://www.ncbi.nlm.nih.gov/entrez/query.fcgi?cmd=search&db=gene&term=IMPA2) | inositol(myo)-1(or 4)-monophosphatase 2 | 0.0002699 | 0.034 | 23.58 | 47.61 | 11.74 | 12.94 | 26.95 | 16.66 | 28.27 | 30.42 | (3, 2), (4, 2), (6, 2), (3, 5), (3, 8) |
| [209771_x_at](https://www.affymetrix.com/LinkServlet?probeset=209771_x_at) | [CD24](http://www.ncbi.nlm.nih.gov/entrez/query.fcgi?cmd=search&db=gene&term=CD24) | CD24 molecule | 0.0002719 | 0.034 | 410.34 | 19.94 | 36.79 | 41.74 | 27.54 | 13.98 | 84.75 | 316.64 | (2, 1), (3, 1), (4, 1), (5, 1), (6, 1), (2, 8), (5, 8), (6, 8) |
| [239340_at](https://www.affymetrix.com/LinkServlet?probeset=239340_at) | [NA](http://www.ncbi.nlm.nih.gov/entrez/query.fcgi?cmd=search&db=gene&term=NA) | NA | 0.0002859 | 0.0353 | 70.75 | 7.53 | 129.55 | 44.15 | 57.07 | 17.36 | 241.4 | 29.5 | (2, 1), (2, 3), (2, 4), (2, 5), (2, 7), (6, 3), (6, 7) |
| [222484_s_at](https://www.affymetrix.com/LinkServlet?probeset=222484_s_at) | [CXCL14](http://www.ncbi.nlm.nih.gov/entrez/query.fcgi?cmd=search&db=gene&term=CXCL14) | chemokine (C-X-C motif) ligand 14 | 0.0002869 | 0.0353 | 1809.84 | 297.03 | 50.36 | 43.01 | 63.12 | 30.95 | 88.68 | 281.04 | (3, 1), (4, 1), (5, 1), (6, 1), (6, 2) |
| [235321_at](https://www.affymetrix.com/LinkServlet?probeset=235321_at) | [NA](http://www.ncbi.nlm.nih.gov/entrez/query.fcgi?cmd=search&db=gene&term=NA) | NA | 0.0002869 | 0.0353 | 13.22 | 8.85 | 10.05 | 8.48 | 15.18 | 12.49 | 23.55 | 8.75 | (2, 5), (2, 7), (3, 7), (4, 5), (4, 7), (8, 5), (8, 7) |
| [212762_s_at](https://www.affymetrix.com/LinkServlet?probeset=212762_s_at) | [TCF7L2](http://www.ncbi.nlm.nih.gov/entrez/query.fcgi?cmd=search&db=gene&term=TCF7L2) | transcription factor 7-like 2 (T-cell specific, HMG-box) | 0.0002895 | 0.0354 | 63.46 | 42.63 | 73.68 | 117.33 | 87.63 | 179.39 | 102.64 | 41.17 | (1, 6), (2, 4), (2, 6), (3, 6), (8, 4), (8, 6) |
| [209368_at](https://www.affymetrix.com/LinkServlet?probeset=209368_at) | [EPHX2](http://www.ncbi.nlm.nih.gov/entrez/query.fcgi?cmd=search&db=gene&term=EPHX2) | epoxide hydrolase 2, cytoplasmic | 0.0002919 | 0.0355 | 10.38 | 10.74 | 9.71 | 9.34 | 10.46 | 8.73 | 61.26 | 10.93 | (1, 7), (2, 7), (3, 7), (4, 7), (5, 7), (6, 7), (8, 7) |
| [214761_at](https://www.affymetrix.com/LinkServlet?probeset=214761_at) | [ZNF423](http://www.ncbi.nlm.nih.gov/entrez/query.fcgi?cmd=search&db=gene&term=ZNF423) | zinc finger protein 423 | 0.0002981 | 0.036 | 266.12 | 596.23 | 1034.53 | 1229.48 | 1136.87 | 1003.76 | 1070.43 | 602.51 | (1, 2), (1, 3), (1, 4), (1, 5), (1, 6), (1, 7) |
| [204584_at](https://www.affymetrix.com/LinkServlet?probeset=204584_at) | [L1CAM](http://www.ncbi.nlm.nih.gov/entrez/query.fcgi?cmd=search&db=gene&term=L1CAM) | L1 cell adhesion molecule | 0.0003001 | 0.036 | 313.24 | 77.59 | 70.12 | 19.78 | 15.05 | 9.34 | 145.53 | 237.06 | (4, 1), (5, 1), (6, 1), (6, 2), (4, 8), (5, 8), (6, 8) |
| [234996_at](https://www.affymetrix.com/LinkServlet?probeset=234996_at) | [CALCRL](http://www.ncbi.nlm.nih.gov/entrez/query.fcgi?cmd=search&db=gene&term=CALCRL) | calcitonin receptor-like | 0.0003006 | 0.036 | 70.65 | 110.09 | 189.63 | 300.96 | 215.79 | 515.47 | 129.5 | 126.12 | (1, 4), (1, 5), (1, 6), (2, 4), (2, 6), (8, 6) |
| [232054_at](https://www.affymetrix.com/LinkServlet?probeset=232054_at) | [PCDH20](http://www.ncbi.nlm.nih.gov/entrez/query.fcgi?cmd=search&db=gene&term=PCDH20) | protocadherin 20 | 0.0003154 | 0.0376 | 36.43 | 39.67 | 178.24 | 273.79 | 274.11 | 496.41 | 66.38 | 33.03 | (1, 4), (1, 5), (1, 6), (2, 4), (2, 5), (2, 6), (8, 4), (8, 5), (8, 6) |
| [212445_s_at](https://www.affymetrix.com/LinkServlet?probeset=212445_s_at) | [NEDD4L](http://www.ncbi.nlm.nih.gov/entrez/query.fcgi?cmd=search&db=gene&term=NEDD4L) | neural precursor cell expressed, developmentally down-regulated 4-like | 0.0003209 | 0.0379 | 30.64 | 8.36 | 6.86 | 5.43 | 7.86 | 5.4 | 11.9 | 6.79 | (2, 1), (3, 1), (4, 1), (5, 1), (6, 1), (8, 1) |
| [226630_at](https://www.affymetrix.com/LinkServlet?probeset=226630_at) | [C14orf106](http://www.ncbi.nlm.nih.gov/entrez/query.fcgi?cmd=search&db=gene&term=C14orf106) | chromosome 14 open reading frame 106 | 0.0003235 | 0.0379 | 30.45 | 58.64 | 17.77 | 23.86 | 37.37 | 20.88 | 13.73 | 29.05 | (1, 2), (3, 2), (4, 2), (6, 2), (7, 2), (3, 5) |
| [235831_at](https://www.affymetrix.com/LinkServlet?probeset=235831_at) | [NA](http://www.ncbi.nlm.nih.gov/entrez/query.fcgi?cmd=search&db=gene&term=NA) | NA | 0.0003266 | 0.0379 | 14.03 | 52.97 | 120.69 | 126.9 | 52.41 | 114.61 | 115.73 | 43.86 | (1, 2), (1, 3), (1, 4), (1, 5), (1, 6), (1, 7) |
| [201572_x_at](https://www.affymetrix.com/LinkServlet?probeset=201572_x_at) | [DCTD](http://www.ncbi.nlm.nih.gov/entrez/query.fcgi?cmd=search&db=gene&term=DCTD) | dCMP deaminase | 0.0003269 | 0.0379 | 347.55 | 298.45 | 157.9 | 191.59 | 234.75 | 213.51 | 278.24 | 256.73 | (3, 1), (4, 1), (6, 1), (3, 2), (4, 2), (3, 8) |
| [219557_s_at](https://www.affymetrix.com/LinkServlet?probeset=219557_s_at) | [NRIP3](http://www.ncbi.nlm.nih.gov/entrez/query.fcgi?cmd=search&db=gene&term=NRIP3) | nuclear receptor interacting protein 3 | 0.0003271 | 0.0379 | 55.83 | 40.59 | 259.66 | 214 | 149.13 | 176.32 | 473.28 | 121.3 | (1, 3), (1, 4), (1, 7), (2, 3), (2, 4), (2, 5), (2, 6), (2, 7) |
| [1556212_x_at](https://www.affymetrix.com/LinkServlet?probeset=1556212_x_at) | [NA](http://www.ncbi.nlm.nih.gov/entrez/query.fcgi?cmd=search&db=gene&term=NA) | NA | 0.000331 | 0.0379 | 10.47 | 12.35 | 25.7 | 53.34 | 15.78 | 30.82 | 25.07 | 15.33 | (1, 3), (1, 4), (1, 6), (2, 4), (2, 6), (5, 4), (8, 4) |
| [242715_at](https://www.affymetrix.com/LinkServlet?probeset=242715_at) | [NA](http://www.ncbi.nlm.nih.gov/entrez/query.fcgi?cmd=search&db=gene&term=NA) | NA | 0.0003321 | 0.0379 | 172.43 | 10.31 | 459.49 | 169.26 | 123.98 | 32.22 | 535.05 | 62.29 | (2, 1), (2, 3), (2, 4), (2, 5), (2, 7), (6, 3) |
| [1552430_at](https://www.affymetrix.com/LinkServlet?probeset=1552430_at) | [WDR17](http://www.ncbi.nlm.nih.gov/entrez/query.fcgi?cmd=search&db=gene&term=WDR17) | WD repeat domain 17 | 0.0003322 | 0.0379 | 15.31 | 17 | 42.14 | 42.74 | 27.49 | 53.87 | 46.75 | 21.3 | (1, 3), (1, 4), (1, 6), (2, 3), (2, 4), (2, 6), (8, 6) |
| [231234_at](https://www.affymetrix.com/LinkServlet?probeset=231234_at) | [CTSC](http://www.ncbi.nlm.nih.gov/entrez/query.fcgi?cmd=search&db=gene&term=CTSC) | cathepsin C | 0.000346 | 0.0391 | 120.33 | 347.54 | 157.48 | 142.86 | 131.98 | 160.8 | 65.96 | 129.11 | (1, 2), (3, 2), (4, 2), (5, 2), (6, 2), (7, 2), (8, 2) |
| [1553720_a_at](https://www.affymetrix.com/LinkServlet?probeset=1553720_a_at) | [FAM123A](http://www.ncbi.nlm.nih.gov/entrez/query.fcgi?cmd=search&db=gene&term=FAM123A) | family with sequence similarity 123A | 0.0003463 | 0.0391 | 205.19 | 78.21 | 342.47 | 371.4 | 344.61 | 359 | 811.9 | 189.52 | (2, 3), (2, 4), (2, 5), (2, 6), (2, 7) |
| [232000_at](https://www.affymetrix.com/LinkServlet?probeset=232000_at) | [TTC39B](http://www.ncbi.nlm.nih.gov/entrez/query.fcgi?cmd=search&db=gene&term=TTC39B) | tetratricopeptide repeat domain 39B | 0.0003532 | 0.0397 | 77.58 | 226.33 | 226.87 | 192.4 | 127.09 | 248.05 | 93.33 | 143.1 | (1, 2), (1, 3), (1, 4), (1, 6) |
| [235657_at](https://www.affymetrix.com/LinkServlet?probeset=235657_at) | [NA](http://www.ncbi.nlm.nih.gov/entrez/query.fcgi?cmd=search&db=gene&term=NA) | NA | 0.0003641 | 0.0401 | 18.21 | 30.29 | 11.65 | 14.76 | 14.59 | 14.06 | 8.64 | 21.32 | (3, 2), (4, 2), (5, 2), (6, 2), (7, 2), (7, 8) |
| [218889_at](https://www.affymetrix.com/LinkServlet?probeset=218889_at) | [NOC3L](http://www.ncbi.nlm.nih.gov/entrez/query.fcgi?cmd=search&db=gene&term=NOC3L) | nucleolar complex associated 3 homolog (S. cerevisiae) | 0.0003642 | 0.0401 | 294.18 | 537.93 | 296.14 | 280.06 | 274.79 | 313.1 | 261.26 | 328.27 | (1, 2), (3, 2), (4, 2), (5, 2), (6, 2), (7, 2), (8, 2) |
| [239782_at](https://www.affymetrix.com/LinkServlet?probeset=239782_at) | [RBP1](http://www.ncbi.nlm.nih.gov/entrez/query.fcgi?cmd=search&db=gene&term=RBP1) | retinol binding protein 1, cellular | 0.0003651 | 0.0401 | 7.86 | 9.72 | 15.85 | 14.38 | 19.46 | 17.26 | 11.26 | 11.63 | (1, 3), (1, 4), (1, 5), (1, 6), (2, 5), (2, 6) |
| [214633_at](https://www.affymetrix.com/LinkServlet?probeset=214633_at) | [SOX3](http://www.ncbi.nlm.nih.gov/entrez/query.fcgi?cmd=search&db=gene&term=SOX3) | SRY (sex determining region Y)-box 3 | 0.0003658 | 0.0401 | 14.92 | 13.88 | 19.4 | 50.28 | 44.01 | 48.54 | 18.67 | 15.48 | (1, 4), (1, 5), (1, 6), (2, 4), (2, 5), (2, 6), (8, 4), (8, 5), (8, 6) |
| [202430_s_at](https://www.affymetrix.com/LinkServlet?probeset=202430_s_at) | [PLSCR1](http://www.ncbi.nlm.nih.gov/entrez/query.fcgi?cmd=search&db=gene&term=PLSCR1) | phospholipid scramblase 1 | 0.0003676 | 0.0401 | 562.26 | 1311.81 | 523.39 | 668.85 | 984.27 | 1060.56 | 296.11 | 838.4 | (1, 2), (3, 2), (4, 2), (7, 2), (3, 6), (7, 5), (7, 6), (7, 8) |
| [1569178_at](https://www.affymetrix.com/LinkServlet?probeset=1569178_at) | [GRIA4](http://www.ncbi.nlm.nih.gov/entrez/query.fcgi?cmd=search&db=gene&term=GRIA4) | glutamate receptor, ionotrophic, AMPA 4 | 0.0003678 | 0.0401 | 13.83 | 64.02 | 64.08 | 88.53 | 67.42 | 100.49 | 79.08 | 30.62 | (1, 2), (1, 3), (1, 4), (1, 5), (1, 6), (1, 7) |
| [225383_at](https://www.affymetrix.com/LinkServlet?probeset=225383_at) | [ZNF275](http://www.ncbi.nlm.nih.gov/entrez/query.fcgi?cmd=search&db=gene&term=ZNF275) | zinc finger protein 275 | 0.0003772 | 0.0408 | 352.25 | 234.38 | 363.08 | 528.07 | 530.11 | 569.11 | 761.78 | 264.34 | (2, 4), (2, 5), (2, 6), (2, 7), (8, 4), (8, 5), (8, 6), (8, 7) |
| [218541_s_at](https://www.affymetrix.com/LinkServlet?probeset=218541_s_at) | [C8orf4](http://www.ncbi.nlm.nih.gov/entrez/query.fcgi?cmd=search&db=gene&term=C8orf4) | chromosome 8 open reading frame 4 | 0.0003812 | 0.0408 | 400.18 | 310.98 | 67.06 | 45.94 | 235.9 | 76.21 | 26.32 | 223.71 | (3, 1), (4, 1), (6, 1), (7, 1), (3, 2), (4, 2), (7, 2), (4, 5), (7, 5) |
| [211484_s_at](https://www.affymetrix.com/LinkServlet?probeset=211484_s_at) | [DSCAM](http://www.ncbi.nlm.nih.gov/entrez/query.fcgi?cmd=search&db=gene&term=DSCAM) | Down syndrome cell adhesion molecule | 0.0003817 | 0.0408 | 67.86 | 60.7 | 141.18 | 336 | 243.68 | 408.54 | 288.48 | 59.78 | (1, 4), (1, 6), (2, 4), (2, 5), (2, 6), (8, 4), (8, 6) |
| [224970_at](https://www.affymetrix.com/LinkServlet?probeset=224970_at) | [NFIA](http://www.ncbi.nlm.nih.gov/entrez/query.fcgi?cmd=search&db=gene&term=NFIA) | nuclear factor I/A | 0.0003836 | 0.0408 | 1459.77 | 1223.78 | 2311.99 | 2934.21 | 2111.97 | 2922.15 | 2534.9 | 1887.53 | (1, 4), (1, 6), (2, 3), (2, 4), (2, 5), (2, 6) |
| [232235_at](https://www.affymetrix.com/LinkServlet?probeset=232235_at) | [DSEL](http://www.ncbi.nlm.nih.gov/entrez/query.fcgi?cmd=search&db=gene&term=DSEL) | dermatan sulfate epimerase-like | 0.0003848 | 0.0408 | 1119.41 | 1151.26 | 1600.87 | 2502.35 | 1781.47 | 2476.56 | 985.59 | 1295.17 | (1, 4), (1, 6), (2, 4), (2, 6), (7, 4), (8, 4), (7, 6), (8, 6) |
| [209617_s_at](https://www.affymetrix.com/LinkServlet?probeset=209617_s_at) | [CTNND2](http://www.ncbi.nlm.nih.gov/entrez/query.fcgi?cmd=search&db=gene&term=CTNND2) | catenin (cadherin-associated protein), delta 2 (neural plakophilin-related arm-repeat protein) | 0.0003851 | 0.0408 | 1321.24 | 535.4 | 1238.05 | 1794.07 | 1599.61 | 1698.53 | 1991.79 | 1171.82 | (2, 1), (2, 3), (2, 4), (2, 5), (2, 6), (2, 7), (2, 8) |
| [1553765_a_at](https://www.affymetrix.com/LinkServlet?probeset=1553765_a_at) | [KLHL32](http://www.ncbi.nlm.nih.gov/entrez/query.fcgi?cmd=search&db=gene&term=KLHL32) | kelch-like 32 (Drosophila) | 0.0003879 | 0.0409 | 46.86 | 34.83 | 257.12 | 278 | 144.94 | 249.67 | 252.52 | 35.99 | (1, 3), (1, 4), (1, 6), (2, 3), (2, 4), (2, 6), (8, 3), (8, 4), (8, 6) |
| [241833_at](https://www.affymetrix.com/LinkServlet?probeset=241833_at) | [NA](http://www.ncbi.nlm.nih.gov/entrez/query.fcgi?cmd=search&db=gene&term=NA) | NA | 0.0003936 | 0.0413 | 109.14 | 6 | 33.95 | 22.8 | 35.37 | 13.2 | 350.7 | 11.93 | (2, 1), (6, 1), (8, 1), (2, 5), (2, 7), (4, 7), (6, 7), (8, 7) |
| [216379_x_at](https://www.affymetrix.com/LinkServlet?probeset=216379_x_at) | [CD24](http://www.ncbi.nlm.nih.gov/entrez/query.fcgi?cmd=search&db=gene&term=CD24) | CD24 molecule | 0.0003982 | 0.0415 | 387.05 | 21.93 | 35.36 | 36.28 | 28.59 | 12.49 | 75.7 | 270.26 | (2, 1), (3, 1), (4, 1), (5, 1), (6, 1), (2, 8), (5, 8), (6, 8) |
| [1552767_a_at](https://www.affymetrix.com/LinkServlet?probeset=1552767_a_at) | [HS6ST2](http://www.ncbi.nlm.nih.gov/entrez/query.fcgi?cmd=search&db=gene&term=HS6ST2) | heparan sulfate 6-O-sulfotransferase 2 | 0.0004012 | 0.0417 | 116.36 | 653.27 | 549.77 | 933.75 | 899.86 | 1116.21 | 164.13 | 432 | (1, 2), (1, 3), (1, 4), (1, 5), (1, 6), (7, 6) |
| [1558387_at](https://www.affymetrix.com/LinkServlet?probeset=1558387_at) | [LOC643763](http://www.ncbi.nlm.nih.gov/entrez/query.fcgi?cmd=search&db=gene&term=LOC643763) | hypothetical LOC643763 | 0.000404 | 0.0417 | 98.44 | 8.68 | 92.06 | 151.32 | 104.77 | 124.81 | 422.98 | 48.55 | (2, 1), (2, 3), (2, 4), (2, 5), (2, 6), (2, 7) |
| [219090_at](https://www.affymetrix.com/LinkServlet?probeset=219090_at) | [SLC24A3](http://www.ncbi.nlm.nih.gov/entrez/query.fcgi?cmd=search&db=gene&term=SLC24A3) | solute carrier family 24 (sodium/potassium/calcium exchanger), member 3 | 0.0004056 | 0.0417 | 100.27 | 110.16 | 334.71 | 358.01 | 418.47 | 444.29 | 548.37 | 249.55 | (1, 3), (1, 4), (1, 5), (1, 6), (1, 7), (2, 3), (2, 4), (2, 5), (2, 6), (2, 7) |
| [219837_s_at](https://www.affymetrix.com/LinkServlet?probeset=219837_s_at) | [CYTL1](http://www.ncbi.nlm.nih.gov/entrez/query.fcgi?cmd=search&db=gene&term=CYTL1) | cytokine-like 1 | 0.0004074 | 0.0417 | 80.8 | 195.6 | 13.71 | 30.38 | 94.35 | 45.68 | 21.56 | 91.81 | (3, 1), (3, 2), (4, 2), (6, 2), (7, 2), (3, 5), (3, 8) |
| [209985_s_at](https://www.affymetrix.com/LinkServlet?probeset=209985_s_at) | [ASCL1](http://www.ncbi.nlm.nih.gov/entrez/query.fcgi?cmd=search&db=gene&term=ASCL1) | achaete-scute complex homolog 1 (Drosophila) | 0.0004122 | 0.0418 | 8.06 | 8.24 | 9.03 | 8.86 | 24.12 | 25.39 | 13.19 | 8.79 | (1, 5), (1, 6), (2, 5), (2, 6), (3, 5), (3, 6), (4, 5), (4, 6), (8, 5), (8, 6) |
| [206715_at](https://www.affymetrix.com/LinkServlet?probeset=206715_at) | [TFEC](http://www.ncbi.nlm.nih.gov/entrez/query.fcgi?cmd=search&db=gene&term=TFEC) | transcription factor EC | 0.0004125 | 0.0418 | 34.25 | 81.01 | 9.87 | 11.65 | 35.82 | 17.08 | 16.65 | 39.96 | (3, 1), (3, 2), (4, 2), (6, 2), (3, 5), (3, 8) |
| [221002_s_at](https://www.affymetrix.com/LinkServlet?probeset=221002_s_at) | [TSPAN14](http://www.ncbi.nlm.nih.gov/entrez/query.fcgi?cmd=search&db=gene&term=TSPAN14) | tetraspanin 14 | 0.0004207 | 0.0425 | 151.95 | 166 | 68.21 | 90.68 | 139.59 | 100.28 | 110.22 | 128 | (3, 1), (4, 1), (3, 2), (4, 2), (6, 2), (3, 5), (3, 8) |
| [229740_at](https://www.affymetrix.com/LinkServlet?probeset=229740_at) | [LOC643008](http://www.ncbi.nlm.nih.gov/entrez/query.fcgi?cmd=search&db=gene&term=LOC643008) | hypothetical protein LOC643008 | 0.0004224 | 0.0425 | 108.36 | 85.62 | 58.78 | 19.42 | 22.64 | 21.88 | 23.99 | 58.17 | (4, 1), (5, 1), (6, 1), (4, 2), (5, 2), (6, 2) |
| [1557745_at](https://www.affymetrix.com/LinkServlet?probeset=1557745_at) | [NA](http://www.ncbi.nlm.nih.gov/entrez/query.fcgi?cmd=search&db=gene&term=NA) | NA | 0.0004296 | 0.043 | 30.34 | 10.67 | 87.38 | 300.95 | 60.75 | 99.9 | 85.65 | 35.29 | (1, 4), (2, 3), (2, 4), (2, 5), (2, 6), (8, 4) |
| [225706_at](https://www.affymetrix.com/LinkServlet?probeset=225706_at) | [GLCCI1](http://www.ncbi.nlm.nih.gov/entrez/query.fcgi?cmd=search&db=gene&term=GLCCI1) | glucocorticoid induced transcript 1 | 0.0004322 | 0.043 | 277.77 | 302.9 | 620.16 | 745.52 | 603.58 | 646.11 | 495.39 | 388.1 | (1, 3), (1, 4), (1, 5), (1, 6), (2, 3), (2, 4), (2, 5), (2, 6) |
| [206634_at](https://www.affymetrix.com/LinkServlet?probeset=206634_at) | [SIX3](http://www.ncbi.nlm.nih.gov/entrez/query.fcgi?cmd=search&db=gene&term=SIX3) | SIX homeobox 3 | 0.0004336 | 0.043 | 4.9 | 27.06 | 4.75 | 4.75 | 4.75 | 4.76 | 4.75 | 50.89 | (1, 2), (1, 8), (3, 2), (4, 2), (5, 2), (6, 2), (3, 8), (4, 8), (5, 8), (6, 8), (7, 8) |
| [1556210_at](https://www.affymetrix.com/LinkServlet?probeset=1556210_at) | [NA](http://www.ncbi.nlm.nih.gov/entrez/query.fcgi?cmd=search&db=gene&term=NA) | NA | 0.0004412 | 0.0434 | 9.07 | 11.74 | 19.38 | 62.09 | 15.13 | 33.26 | 20.93 | 11.74 | (1, 4), (1, 6), (2, 4), (2, 6), (3, 4), (5, 4), (8, 4) |
| [224975_at](https://www.affymetrix.com/LinkServlet?probeset=224975_at) | [NFIA](http://www.ncbi.nlm.nih.gov/entrez/query.fcgi?cmd=search&db=gene&term=NFIA) | nuclear factor I/A | 0.0004418 | 0.0434 | 1481.4 | 1288.01 | 2107.16 | 2554.2 | 2172.62 | 2971.96 | 2504.6 | 2061.89 | (1, 4), (1, 6), (2, 3), (2, 4), (2, 5), (2, 6) |
| [226022_at](https://www.affymetrix.com/LinkServlet?probeset=226022_at) | [SASH1](http://www.ncbi.nlm.nih.gov/entrez/query.fcgi?cmd=search&db=gene&term=SASH1) | SAM and SH3 domain containing 1 | 0.0004455 | 0.0436 | 1776.69 | 1526.7 | 2714.24 | 3352.26 | 2486.62 | 2814.99 | 5294.57 | 1987.73 | (1, 4), (1, 7), (2, 3), (2, 4), (2, 6), (2, 7), (8, 7) |
| [229560_at](https://www.affymetrix.com/LinkServlet?probeset=229560_at) | [TLR8](http://www.ncbi.nlm.nih.gov/entrez/query.fcgi?cmd=search&db=gene&term=TLR8) | toll-like receptor 8 | 0.0004497 | 0.0436 | 54.98 | 125.32 | 16.32 | 31.35 | 90.43 | 47.59 | 25.24 | 60.66 | (3, 1), (3, 2), (4, 2), (7, 2), (3, 5), (3, 8) |
| [229034_at](https://www.affymetrix.com/LinkServlet?probeset=229034_at) | [SOBP](http://www.ncbi.nlm.nih.gov/entrez/query.fcgi?cmd=search&db=gene&term=SOBP) | sine oculis binding protein homolog (Drosophila) | 0.0004498 | 0.0436 | 164.77 | 114.56 | 282.21 | 351.4 | 235.18 | 317.91 | 268.2 | 145.03 | (1, 4), (2, 3), (2, 4), (2, 5), (2, 6), (8, 4), (8, 6) |
| [218002_s_at](https://www.affymetrix.com/LinkServlet?probeset=218002_s_at) | [CXCL14](http://www.ncbi.nlm.nih.gov/entrez/query.fcgi?cmd=search&db=gene&term=CXCL14) | chemokine (C-X-C motif) ligand 14 | 0.0004551 | 0.0439 | 2359.44 | 315.79 | 70.35 | 57.83 | 67.19 | 29.88 | 88.7 | 314.54 | (3, 1), (4, 1), (5, 1), (6, 1), (6, 2) |
| [210839_s_at](https://www.affymetrix.com/LinkServlet?probeset=210839_s_at) | [ENPP2](http://www.ncbi.nlm.nih.gov/entrez/query.fcgi?cmd=search&db=gene&term=ENPP2) | ectonucleotide pyrophosphatase/phosphodiesterase 2 | 0.0004576 | 0.0439 | 181.54 | 17.32 | 23.44 | 19.9 | 88.96 | 29.72 | 385.35 | 24.46 | (2, 1), (3, 1), (4, 1), (6, 1), (8, 1), (2, 5), (2, 7), (3, 7), (4, 7), (6, 7), (8, 7) |
| [205479_s_at](https://www.affymetrix.com/LinkServlet?probeset=205479_s_at) | [PLAU](http://www.ncbi.nlm.nih.gov/entrez/query.fcgi?cmd=search&db=gene&term=PLAU) | plasminogen activator, urokinase | 0.0004586 | 0.0439 | 189.69 | 950.79 | 102.01 | 184.2 | 394.34 | 405.26 | 20.61 | 362.52 | (1, 2), (7, 1), (3, 2), (4, 2), (7, 2), (7, 5), (7, 6), (7, 8) |
| [237365_at](https://www.affymetrix.com/LinkServlet?probeset=237365_at) | [NA](http://www.ncbi.nlm.nih.gov/entrez/query.fcgi?cmd=search&db=gene&term=NA) | NA | 0.0004647 | 0.0442 | 20.08 | 8.23 | 8.41 | 7.05 | 5.81 | 5.11 | 188.3 | 5.89 | (6, 1), (1, 7), (2, 7), (3, 7), (4, 7), (5, 7), (6, 7), (8, 7) |
| [210279_at](https://www.affymetrix.com/LinkServlet?probeset=210279_at) | [GPR18](http://www.ncbi.nlm.nih.gov/entrez/query.fcgi?cmd=search&db=gene&term=GPR18) | G protein-coupled receptor 18 | 0.0004665 | 0.0442 | 6.49 | 10.22 | 5.2 | 5.55 | 6.29 | 5.54 | 5.3 | 5.88 | (1, 2), (3, 2), (4, 2), (5, 2), (6, 2), (7, 2), (8, 2) |
| [220146_at](https://www.affymetrix.com/LinkServlet?probeset=220146_at) | [TLR7](http://www.ncbi.nlm.nih.gov/entrez/query.fcgi?cmd=search&db=gene&term=TLR7) | toll-like receptor 7 | 0.0004689 | 0.0443 | 132.47 | 325.33 | 35.27 | 73.25 | 164.03 | 139.46 | 107.06 | 249.83 | (3, 1), (3, 2), (4, 2), (3, 5), (3, 6), (3, 8) |
| [230644_at](https://www.affymetrix.com/LinkServlet?probeset=230644_at) | [LRFN5](http://www.ncbi.nlm.nih.gov/entrez/query.fcgi?cmd=search&db=gene&term=LRFN5) | leucine rich repeat and fibronectin type III domain containing 5 | 0.0004711 | 0.0443 | 16.23 | 147.9 | 61.66 | 143.31 | 106.9 | 133.49 | 155.16 | 25.61 | (1, 2), (1, 4), (1, 5), (1, 6), (1, 7), (8, 2), (8, 4), (8, 6) |
| [211026_s_at](https://www.affymetrix.com/LinkServlet?probeset=211026_s_at) | [MGLL](http://www.ncbi.nlm.nih.gov/entrez/query.fcgi?cmd=search&db=gene&term=MGLL) | monoglyceride lipase | 0.0004744 | 0.0444 | 587.68 | 467.51 | 1346 | 1816.18 | 1917.74 | 2224.66 | 1931.7 | 709.58 | (1, 4), (1, 5), (1, 6), (2, 3), (2, 4), (2, 5), (2, 6), (8, 6) |
| [222871_at](https://www.affymetrix.com/LinkServlet?probeset=222871_at) | [KLHDC8A](http://www.ncbi.nlm.nih.gov/entrez/query.fcgi?cmd=search&db=gene&term=KLHDC8A) | kelch domain containing 8A | 0.0004777 | 0.0444 | 324.09 | 329.89 | 169.05 | 104.32 | 147.37 | 108.98 | 997.25 | 347.77 | (4, 1), (6, 1), (4, 2), (6, 2), (3, 7), (4, 7), (4, 8), (5, 7), (6, 7), (6, 8) |
| [52837_at](https://www.affymetrix.com/LinkServlet?probeset=52837_at) | [KIAA1644](http://www.ncbi.nlm.nih.gov/entrez/query.fcgi?cmd=search&db=gene&term=KIAA1644) | KIAA1644 | 0.0004784 | 0.0444 | 13.27 | 4.9 | 9.24 | 8.43 | 7.29 | 6.08 | 46.47 | 7.46 | (2, 1), (1, 7), (2, 7), (3, 7), (4, 7), (5, 7), (6, 7), (8, 7) |
| [238133_at](https://www.affymetrix.com/LinkServlet?probeset=238133_at) | [NA](http://www.ncbi.nlm.nih.gov/entrez/query.fcgi?cmd=search&db=gene&term=NA) | NA | 0.0004804 | 0.0444 | 96.81 | 186.86 | 372.05 | 804.29 | 285.3 | 794.85 | 142.4 | 158.12 | (1, 3), (1, 4), (1, 6), (2, 4), (2, 6), (8, 4), (8, 6) |
| [243299_at](https://www.affymetrix.com/LinkServlet?probeset=243299_at) | [VRK2](http://www.ncbi.nlm.nih.gov/entrez/query.fcgi?cmd=search&db=gene&term=VRK2) | vaccinia related kinase 2 | 0.0004883 | 0.045 | 7.43 | 15.52 | 6.96 | 6.34 | 6.97 | 6.65 | 5.88 | 6.96 | (1, 2), (3, 2), (4, 2), (5, 2), (6, 2), (7, 2), (8, 2) |
| [207443_at](https://www.affymetrix.com/LinkServlet?probeset=207443_at) | [NR2E1](http://www.ncbi.nlm.nih.gov/entrez/query.fcgi?cmd=search&db=gene&term=NR2E1) | nuclear receptor subfamily 2, group E, member 1 | 0.0004929 | 0.0452 | 57.58 | 11.99 | 4.95 | 5.51 | 4.98 | 4.75 | 30.99 | 22.9 | (2, 1), (3, 1), (4, 1), (5, 1), (6, 1) |
| [234101_at](https://www.affymetrix.com/LinkServlet?probeset=234101_at) | [NA](http://www.ncbi.nlm.nih.gov/entrez/query.fcgi?cmd=search&db=gene&term=NA) | NA | 0.0004961 | 0.0453 | 9.71 | 19.27 | 47.82 | 71.57 | 19.5 | 51.98 | 18.27 | 23.77 | (1, 3), (1, 4), (1, 6), (2, 4), (5, 4) |
| [223566_s_at](https://www.affymetrix.com/LinkServlet?probeset=223566_s_at) | [BCOR](http://www.ncbi.nlm.nih.gov/entrez/query.fcgi?cmd=search&db=gene&term=BCOR) | BCL6 co-repressor | 0.0005007 | 0.0454 | 15.02 | 13.42 | 7.91 | 8.45 | 15.03 | 10.65 | 34.02 | 8.59 | (3, 1), (2, 7), (3, 5), (3, 7), (4, 7), (6, 7), (8, 7) |
| [209987_s_at](https://www.affymetrix.com/LinkServlet?probeset=209987_s_at) | [ASCL1](http://www.ncbi.nlm.nih.gov/entrez/query.fcgi?cmd=search&db=gene&term=ASCL1) | achaete-scute complex homolog 1 (Drosophila) | 0.0005011 | 0.0454 | 176.06 | 593.46 | 1095.77 | 1820.68 | 1300.36 | 2308.75 | 739.64 | 547.26 | (1, 3), (1, 4), (1, 5), (1, 6) |
| [228083_at](https://www.affymetrix.com/LinkServlet?probeset=228083_at) | [CACNA2D4](http://www.ncbi.nlm.nih.gov/entrez/query.fcgi?cmd=search&db=gene&term=CACNA2D4) | calcium channel, voltage-dependent, alpha 2/delta subunit 4 | 0.000503 | 0.0454 | 8.65 | 15.09 | 7.01 | 7.74 | 9.82 | 7.09 | 7.2 | 10.12 | (1, 2), (3, 2), (4, 2), (6, 2), (7, 2) |
| [207057_at](https://www.affymetrix.com/LinkServlet?probeset=207057_at) | [SLC16A7](http://www.ncbi.nlm.nih.gov/entrez/query.fcgi?cmd=search&db=gene&term=SLC16A7) | solute carrier family 16, member 7 (monocarboxylic acid transporter 2) | 0.0005079 | 0.0454 | 123.17 | 281.47 | 498.47 | 467.81 | 236.22 | 437.69 | 104.36 | 301.21 | (1, 2), (1, 3), (1, 4), (1, 6), (7, 3), (7, 4), (7, 6) |
| [240067_at](https://www.affymetrix.com/LinkServlet?probeset=240067_at) | [NA](http://www.ncbi.nlm.nih.gov/entrez/query.fcgi?cmd=search&db=gene&term=NA) | NA | 0.0005079 | 0.0454 | 25.01 | 25.49 | 194.45 | 248.39 | 49.59 | 184.01 | 96.98 | 37.47 | (1, 3), (1, 4), (1, 6), (2, 3), (2, 4), (2, 6), (8, 4) |
| [206068_s_at](https://www.affymetrix.com/LinkServlet?probeset=206068_s_at) | [ACADL](http://www.ncbi.nlm.nih.gov/entrez/query.fcgi?cmd=search&db=gene&term=ACADL) | acyl-Coenzyme A dehydrogenase, long chain | 0.0005099 | 0.0454 | 14.01 | 61.43 | 20.63 | 32.9 | 36.43 | 37.47 | 24.31 | 29.16 | (1, 2), (1, 4), (1, 5), (1, 6), (3, 2) |
| [213870_at](https://www.affymetrix.com/LinkServlet?probeset=213870_at) | [COL11A2](http://www.ncbi.nlm.nih.gov/entrez/query.fcgi?cmd=search&db=gene&term=COL11A2) | collagen, type XI, alpha 2 | 0.0005122 | 0.0454 | 9.51 | 10.9 | 11.19 | 10.97 | 8.11 | 8.94 | 31.66 | 7.91 | (1, 7), (2, 7), (3, 7), (4, 7), (5, 7), (6, 7), (8, 7) |
| [244007_at](https://www.affymetrix.com/LinkServlet?probeset=244007_at) | [ZNF462](http://www.ncbi.nlm.nih.gov/entrez/query.fcgi?cmd=search&db=gene&term=ZNF462) | zinc finger protein 462 | 0.0005227 | 0.0462 | 603.6 | 538.01 | 725.61 | 1116.78 | 709.3 | 1053.39 | 826.62 | 470.24 | (1, 4), (1, 6), (2, 4), (2, 6), (8, 4), (8, 6) |
| [219269_at](https://www.affymetrix.com/LinkServlet?probeset=219269_at) | [HMBOX1](http://www.ncbi.nlm.nih.gov/entrez/query.fcgi?cmd=search&db=gene&term=HMBOX1) | homeobox containing 1 | 0.0005308 | 0.0465 | 565.38 | 362.39 | 916.75 | 1450.22 | 1114.56 | 552.36 | 1039.03 | 522.83 | (1, 4), (2, 3), (2, 4), (2, 5), (6, 4), (8, 4) |
| [231240_at](https://www.affymetrix.com/LinkServlet?probeset=231240_at) | [DIO2](http://www.ncbi.nlm.nih.gov/entrez/query.fcgi?cmd=search&db=gene&term=DIO2) | deiodinase, iodothyronine, type II | 0.0005308 | 0.0465 | 11.39 | 9.41 | 4.93 | 5.78 | 5.09 | 5 | 30.41 | 5.12 | (3, 1), (5, 1), (6, 1), (2, 7), (3, 7), (4, 7), (5, 7), (6, 7), (8, 7) |
| [232562_at](https://www.affymetrix.com/LinkServlet?probeset=232562_at) | [NA](http://www.ncbi.nlm.nih.gov/entrez/query.fcgi?cmd=search&db=gene&term=NA) | NA | 0.0005345 | 0.0465 | 25.61 | 15.5 | 48.72 | 57.44 | 28.37 | 43.99 | 113.17 | 28.87 | (1, 7), (2, 3), (2, 4), (2, 6), (2, 7), (5, 7), (8, 7) |
| [214770_at](https://www.affymetrix.com/LinkServlet?probeset=214770_at) | [MSR1](http://www.ncbi.nlm.nih.gov/entrez/query.fcgi?cmd=search&db=gene&term=MSR1) | macrophage scavenger receptor 1 | 0.0005349 | 0.0465 | 340.54 | 567.61 | 108.2 | 176.58 | 391.7 | 316.1 | 78.22 | 365.01 | (3, 1), (7, 1), (3, 2), (4, 2), (7, 2), (3, 5), (3, 6), (3, 8), (7, 5), (7, 8) |
| [232874_at](https://www.affymetrix.com/LinkServlet?probeset=232874_at) | [DOCK9](http://www.ncbi.nlm.nih.gov/entrez/query.fcgi?cmd=search&db=gene&term=DOCK9) | dedicator of cytokinesis 9 | 0.0005426 | 0.047 | 30.22 | 28.62 | 120.14 | 289.33 | 57.23 | 137.93 | 39.81 | 34.32 | (1, 4), (1, 6), (2, 3), (2, 4), (2, 6), (5, 4), (8, 4) |
| [213478_at](https://www.affymetrix.com/LinkServlet?probeset=213478_at) | [RP1-21O18.1](http://www.ncbi.nlm.nih.gov/entrez/query.fcgi?cmd=search&db=gene&term=RP1-21O18.1) | kazrin | 0.0005455 | 0.047 | 232.35 | 181.6 | 540.15 | 520.4 | 426.21 | 452.67 | 428.97 | 320.26 | (1, 3), (1, 4), (2, 3), (2, 4), (2, 5), (2, 6) |
| [202295_s_at](https://www.affymetrix.com/LinkServlet?probeset=202295_s_at) | [CTSH](http://www.ncbi.nlm.nih.gov/entrez/query.fcgi?cmd=search&db=gene&term=CTSH) | cathepsin H | 0.0005495 | 0.047 | 729.96 | 1456.74 | 534.91 | 501.12 | 956.08 | 576.8 | 657.24 | 1729.38 | (1, 8), (3, 2), (4, 2), (6, 2), (3, 8), (4, 8), (6, 8) |
| [236664_at](https://www.affymetrix.com/LinkServlet?probeset=236664_at) | [AKT2](http://www.ncbi.nlm.nih.gov/entrez/query.fcgi?cmd=search&db=gene&term=AKT2) | v-akt murine thymoma viral oncogene homolog 2 | 0.000552 | 0.047 | 5.43 | 9.35 | 9.76 | 8.14 | 5.19 | 6.12 | 29.09 | 7.29 | (1, 7), (2, 7), (3, 7), (4, 7), (5, 7), (6, 7), (8, 7) |
| [213108_at](https://www.affymetrix.com/LinkServlet?probeset=213108_at) | [CAMK2A](http://www.ncbi.nlm.nih.gov/entrez/query.fcgi?cmd=search&db=gene&term=CAMK2A) | calcium/calmodulin-dependent protein kinase II alpha | 0.0005532 | 0.047 | 123.84 | 26.5 | 76.24 | 29.13 | 17.01 | 19.72 | 64.78 | 52.55 | (2, 1), (4, 1), (5, 1), (6, 1), (5, 3), (6, 3) |
| [224770_s_at](https://www.affymetrix.com/LinkServlet?probeset=224770_s_at) | [NAV1](http://www.ncbi.nlm.nih.gov/entrez/query.fcgi?cmd=search&db=gene&term=NAV1) | neuron navigator 1 | 0.0005535 | 0.047 | 29.36 | 16.25 | 40.53 | 73.06 | 40.81 | 67.92 | 40.35 | 22.6 | (1, 4), (2, 3), (2, 4), (2, 5), (2, 6), (8, 4), (8, 6) |
| [221082_s_at](https://www.affymetrix.com/LinkServlet?probeset=221082_s_at) | [NDRG3](http://www.ncbi.nlm.nih.gov/entrez/query.fcgi?cmd=search&db=gene&term=NDRG3) | NDRG family member 3 | 0.0005578 | 0.0472 | 38.69 | 36.5 | 80.92 | 48.53 | 57.29 | 62.09 | 81 | 40.25 | (1, 3), (1, 7), (2, 3), (2, 6), (2, 7), (8, 3) |
| [241928_at](https://www.affymetrix.com/LinkServlet?probeset=241928_at) | [NA](http://www.ncbi.nlm.nih.gov/entrez/query.fcgi?cmd=search&db=gene&term=NA) | NA | 0.0005678 | 0.0478 | 11.76 | 29.51 | 30.87 | 67.13 | 22.02 | 32.61 | 9.57 | 14.34 | (1, 2), (1, 4), (1, 6), (5, 4), (7, 4), (8, 4) |
| [226056_at](https://www.affymetrix.com/LinkServlet?probeset=226056_at) | [CDGAP](http://www.ncbi.nlm.nih.gov/entrez/query.fcgi?cmd=search&db=gene&term=CDGAP) | Cdc42 GTPase-activating protein | 0.0005798 | 0.0486 | 431.74 | 190.05 | 321.67 | 486.19 | 414.98 | 512.01 | 672.37 | 251.17 | (2, 1), (2, 4), (2, 5), (2, 6), (2, 7) |
| [213335_s_at](https://www.affymetrix.com/LinkServlet?probeset=213335_s_at) | [ST3GAL6](http://www.ncbi.nlm.nih.gov/entrez/query.fcgi?cmd=search&db=gene&term=ST3GAL6) | ST3 beta-galactoside alpha-2,3-sialyltransferase 6 | 0.0005816 | 0.0486 | 32.82 | 30.68 | 32.55 | 32.51 | 28.19 | 29.83 | 99.17 | 25.45 | (1, 7), (2, 7), (3, 7), (4, 7), (5, 7), (6, 7), (8, 7) |
| [202446_s_at](https://www.affymetrix.com/LinkServlet?probeset=202446_s_at) | [PLSCR1](http://www.ncbi.nlm.nih.gov/entrez/query.fcgi?cmd=search&db=gene&term=PLSCR1) | phospholipid scramblase 1 | 0.0006066 | 0.0504 | 604.86 | 1381.4 | 477.77 | 627.17 | 1405.68 | 1053.66 | 331.87 | 900.77 | (1, 2), (1, 5), (3, 2), (4, 2), (7, 2), (3, 5), (4, 5), (7, 5), (7, 6) |
| [203570_at](https://www.affymetrix.com/LinkServlet?probeset=203570_at) | [LOXL1](http://www.ncbi.nlm.nih.gov/entrez/query.fcgi?cmd=search&db=gene&term=LOXL1) | lysyl oxidase-like 1 | 0.0006068 | 0.0504 | 150.86 | 319.1 | 50.02 | 44.54 | 72.85 | 69.2 | 13.99 | 113.26 | (7, 1), (3, 2), (4, 2), (5, 2), (6, 2), (7, 2), (7, 8) |
| [219563_at](https://www.affymetrix.com/LinkServlet?probeset=219563_at) | [C14orf139](http://www.ncbi.nlm.nih.gov/entrez/query.fcgi?cmd=search&db=gene&term=C14orf139) | chromosome 14 open reading frame 139 | 0.0006131 | 0.0506 | 159.22 | 367.72 | 389.29 | 570.92 | 419.67 | 627.28 | 234.75 | 283.68 | (1, 2), (1, 3), (1, 4), (1, 5), (1, 6) |
| [225502_at](https://www.affymetrix.com/LinkServlet?probeset=225502_at) | [DOCK8](http://www.ncbi.nlm.nih.gov/entrez/query.fcgi?cmd=search&db=gene&term=DOCK8) | dedicator of cytokinesis 8 | 0.000614 | 0.0506 | 186.69 | 357.93 | 87.6 | 160.55 | 255.95 | 244.06 | 104.1 | 278.83 | (3, 2), (4, 2), (7, 2), (3, 5), (3, 6), (3, 8) |
| [207012_at](https://www.affymetrix.com/LinkServlet?probeset=207012_at) | [MMP16](http://www.ncbi.nlm.nih.gov/entrez/query.fcgi?cmd=search&db=gene&term=MMP16) | matrix metallopeptidase 16 (membrane-inserted) | 0.0006214 | 0.0509 | 43.31 | 132.3 | 128.55 | 186.18 | 199.59 | 298.33 | 66.48 | 53.01 | (1, 2), (1, 4), (1, 5), (1, 6), (8, 4), (8, 5), (8, 6) |
| [238009_at](https://www.affymetrix.com/LinkServlet?probeset=238009_at) | [NA](http://www.ncbi.nlm.nih.gov/entrez/query.fcgi?cmd=search&db=gene&term=NA) | NA | 0.000623 | 0.0509 | 508.09 | 485.09 | 766.77 | 1242.16 | 708.06 | 976.66 | 1064.27 | 399.15 | (1, 4), (2, 4), (2, 6), (8, 4), (8, 6) |
| [230030_at](https://www.affymetrix.com/LinkServlet?probeset=230030_at) | [HS6ST2](http://www.ncbi.nlm.nih.gov/entrez/query.fcgi?cmd=search&db=gene&term=HS6ST2) | heparan sulfate 6-O-sulfotransferase 2 | 0.000626 | 0.051 | 41.52 | 214.84 | 167.83 | 280.06 | 335.25 | 383.24 | 53.23 | 151.45 | (1, 2), (1, 3), (1, 4), (1, 5), (1, 6), (7, 6) |
| [1554821_a_at](https://www.affymetrix.com/LinkServlet?probeset=1554821_a_at) | [ZBED1](http://www.ncbi.nlm.nih.gov/entrez/query.fcgi?cmd=search&db=gene&term=ZBED1) | zinc finger, BED-type containing 1 | 0.0006311 | 0.0511 | 97.15 | 61.03 | 135.92 | 141.28 | 168.74 | 249.29 | 85.54 | 129.46 | (1, 6), (2, 3), (2, 4), (2, 5), (2, 6) |
| [1554281_at](https://www.affymetrix.com/LinkServlet?probeset=1554281_at) | [NA](http://www.ncbi.nlm.nih.gov/entrez/query.fcgi?cmd=search&db=gene&term=NA) | NA | 0.0006343 | 0.0511 | 8.04 | 6.31 | 7.4 | 20.51 | 11.93 | 24.79 | 22.56 | 6.47 | (1, 4), (1, 6), (2, 4), (2, 6), (3, 4), (3, 6), (8, 4), (8, 6) |
| [243049_at](https://www.affymetrix.com/LinkServlet?probeset=243049_at) | [NA](http://www.ncbi.nlm.nih.gov/entrez/query.fcgi?cmd=search&db=gene&term=NA) | NA | 0.0006375 | 0.0511 | 5.78 | 5.23 | 6.26 | 24.43 | 8.64 | 8.69 | 5.58 | 5.91 | (1, 4), (2, 4), (3, 4), (5, 4), (6, 4), (7, 4), (8, 4) |
| [231252_at](https://www.affymetrix.com/LinkServlet?probeset=231252_at) | [C2orf67](http://www.ncbi.nlm.nih.gov/entrez/query.fcgi?cmd=search&db=gene&term=C2orf67) | chromosome 2 open reading frame 67 | 0.0006387 | 0.0511 | 35.71 | 64.68 | 93.72 | 155.57 | 51.88 | 98.07 | 65.92 | 51.51 | (1, 3), (1, 4), (1, 6), (2, 4), (5, 4), (8, 4) |
| [232553_at](https://www.affymetrix.com/LinkServlet?probeset=232553_at) | [PCYT1B](http://www.ncbi.nlm.nih.gov/entrez/query.fcgi?cmd=search&db=gene&term=PCYT1B) | phosphate cytidylyltransferase 1, choline, beta | 0.0006395 | 0.0511 | 60.32 | 18.3 | 47.44 | 57.98 | 53.1 | 49.23 | 87.15 | 40.64 | (2, 1), (2, 3), (2, 4), (2, 5), (2, 6), (2, 7), (2, 8) |
| [228641_at](https://www.affymetrix.com/LinkServlet?probeset=228641_at) | [CARD8](http://www.ncbi.nlm.nih.gov/entrez/query.fcgi?cmd=search&db=gene&term=CARD8) | caspase recruitment domain family, member 8 | 0.0006412 | 0.0511 | 52.53 | 93.94 | 47.63 | 51.44 | 60.06 | 49.54 | 24.18 | 74.48 | (1, 2), (3, 2), (4, 2), (6, 2), (7, 2), (7, 5), (7, 8) |
| [227657_at](https://www.affymetrix.com/LinkServlet?probeset=227657_at) | [RNF150](http://www.ncbi.nlm.nih.gov/entrez/query.fcgi?cmd=search&db=gene&term=RNF150) | ring finger protein 150 | 0.0006496 | 0.0516 | 126.54 | 141.47 | 300.37 | 398.74 | 274.06 | 364.54 | 145.28 | 183.24 | (1, 3), (1, 4), (1, 5), (1, 6), (2, 3), (2, 4), (2, 6) |
| [241068_at](https://www.affymetrix.com/LinkServlet?probeset=241068_at) | [NA](http://www.ncbi.nlm.nih.gov/entrez/query.fcgi?cmd=search&db=gene&term=NA) | NA | 0.000657 | 0.0517 | 69.75 | 143.38 | 33.69 | 34.05 | 93.48 | 74.83 | 48.27 | 107.72 | (3, 2), (4, 2), (3, 5), (3, 8), (4, 5), (4, 8) |
| [204304_s_at](https://www.affymetrix.com/LinkServlet?probeset=204304_s_at) | [PROM1](http://www.ncbi.nlm.nih.gov/entrez/query.fcgi?cmd=search&db=gene&term=PROM1) | prominin 1 | 0.0006584 | 0.0517 | 100.08 | 31.9 | 205.75 | 405.08 | 465.95 | 411.28 | 171.45 | 165.86 | (2, 3), (2, 4), (2, 5), (2, 6) |
| [219806_s_at](https://www.affymetrix.com/LinkServlet?probeset=219806_s_at) | [C11orf75](http://www.ncbi.nlm.nih.gov/entrez/query.fcgi?cmd=search&db=gene&term=C11orf75) | chromosome 11 open reading frame 75 | 0.0006585 | 0.0517 | 133.9 | 284.67 | 81.94 | 123.48 | 233.23 | 218.39 | 181.97 | 239.87 | (1, 2), (3, 2), (4, 2), (3, 5), (3, 6), (3, 8) |
| [208002_s_at](https://www.affymetrix.com/LinkServlet?probeset=208002_s_at) | [ACOT7](http://www.ncbi.nlm.nih.gov/entrez/query.fcgi?cmd=search&db=gene&term=ACOT7) | acyl-CoA thioesterase 7 | 0.0006629 | 0.0519 | 418.12 | 253.08 | 380.64 | 178.35 | 376.93 | 257.34 | 283.94 | 348.77 | (2, 1), (4, 1), (4, 3), (4, 5), (4, 8) |
| [205721_at](https://www.affymetrix.com/LinkServlet?probeset=205721_at) | [GFRA2](http://www.ncbi.nlm.nih.gov/entrez/query.fcgi?cmd=search&db=gene&term=GFRA2) | GDNF family receptor alpha 2 | 0.0006677 | 0.0521 | 72.03 | 22.1 | 35.53 | 22.95 | 28.12 | 21.93 | 19.86 | 25.62 | (2, 1), (4, 1), (5, 1), (6, 1), (7, 1), (8, 1) |
| [213894_at](https://www.affymetrix.com/LinkServlet?probeset=213894_at) | [THSD7A](http://www.ncbi.nlm.nih.gov/entrez/query.fcgi?cmd=search&db=gene&term=THSD7A) | thrombospondin, type I, domain containing 7A | 0.0006745 | 0.0523 | 49.8 | 64.07 | 191.36 | 272.53 | 274.32 | 337.75 | 187.2 | 58.98 | (1, 4), (1, 5), (1, 6), (2, 4), (2, 5), (2, 6), (8, 4), (8, 5), (8, 6) |
| [237169_at](https://www.affymetrix.com/LinkServlet?probeset=237169_at) | [NA](http://www.ncbi.nlm.nih.gov/entrez/query.fcgi?cmd=search&db=gene&term=NA) | NA | 0.000675 | 0.0523 | 34.73 | 10.56 | 7.56 | 8.72 | 13.52 | 8.11 | 136.21 | 15.94 | (2, 1), (3, 1), (4, 1), (6, 1), (2, 7), (3, 7), (4, 7), (5, 7), (6, 7), (8, 7) |
| [219528_s_at](https://www.affymetrix.com/LinkServlet?probeset=219528_s_at) | [BCL11B](http://www.ncbi.nlm.nih.gov/entrez/query.fcgi?cmd=search&db=gene&term=BCL11B) | B-cell CLL/lymphoma 11B (zinc finger protein) | 0.00068 | 0.0523 | 13.57 | 15.22 | 24.56 | 47.57 | 38.2 | 49.65 | 11.75 | 12.83 | (1, 4), (1, 5), (1, 6), (2, 4), (2, 6), (8, 4), (8, 5), (8, 6) |
| [213506_at](https://www.affymetrix.com/LinkServlet?probeset=213506_at) | [F2RL1](http://www.ncbi.nlm.nih.gov/entrez/query.fcgi?cmd=search&db=gene&term=F2RL1) | coagulation factor II (thrombin) receptor-like 1 | 0.0006814 | 0.0523 | 38.53 | 173.16 | 29.32 | 94.82 | 126.34 | 127.18 | 21.85 | 60.97 | (1, 2), (3, 2), (7, 2), (3, 5), (3, 6), (7, 6) |
| [219797_at](https://www.affymetrix.com/LinkServlet?probeset=219797_at) | [MGAT4A](http://www.ncbi.nlm.nih.gov/entrez/query.fcgi?cmd=search&db=gene&term=MGAT4A) | mannosyl (alpha-1,3-)-glycoprotein beta-1,4-N-acetylglucosaminyltransferase, isozyme A | 0.0006831 | 0.0523 | 33.14 | 71.08 | 22.08 | 20.72 | 38.23 | 30.37 | 20.08 | 44.55 | (1, 2), (3, 2), (4, 2), (6, 2), (7, 2) |
| [228646_at](https://www.affymetrix.com/LinkServlet?probeset=228646_at) | [PPP1R1C](http://www.ncbi.nlm.nih.gov/entrez/query.fcgi?cmd=search&db=gene&term=PPP1R1C) | protein phosphatase 1, regulatory (inhibitor) subunit 1C | 0.0006893 | 0.0526 | 134.55 | 53.2 | 16.98 | 24.25 | 15.5 | 16.24 | 67.6 | 59.21 | (3, 1), (4, 1), (5, 1), (6, 1) |
| [240922_at](https://www.affymetrix.com/LinkServlet?probeset=240922_at) | [NA](http://www.ncbi.nlm.nih.gov/entrez/query.fcgi?cmd=search&db=gene&term=NA) | NA | 0.0006924 | 0.0527 | 9.83 | 12.6 | 29.92 | 27.15 | 10.75 | 9.4 | 12.49 | 15.35 | (1, 3), (1, 4), (2, 3), (2, 4), (5, 3), (6, 3), (5, 4), (6, 4) |
| [242521_at](https://www.affymetrix.com/LinkServlet?probeset=242521_at) | [NA](http://www.ncbi.nlm.nih.gov/entrez/query.fcgi?cmd=search&db=gene&term=NA) | NA | 0.0007058 | 0.0535 | 133.2 | 281.19 | 78.46 | 82.36 | 114.42 | 101.51 | 63.43 | 151.69 | (1, 2), (3, 2), (4, 2), (5, 2), (6, 2), (7, 2) |
| [238021_s_at](https://www.affymetrix.com/LinkServlet?probeset=238021_s_at) | [CRNDE](http://www.ncbi.nlm.nih.gov/entrez/query.fcgi?cmd=search&db=gene&term=CRNDE) | colorectal neoplasia differentially expressed (non-protein coding) | 0.00071 | 0.0536 | 57.53 | 382.83 | 1055.41 | 1410.74 | 1184.95 | 956.26 | 150.11 | 456.28 | (1, 2), (1, 3), (1, 4), (1, 5), (1, 6), (1, 8) |
| [225579_at](https://www.affymetrix.com/LinkServlet?probeset=225579_at) | [PQLC3](http://www.ncbi.nlm.nih.gov/entrez/query.fcgi?cmd=search&db=gene&term=PQLC3) | PQ loop repeat containing 3 | 0.0007232 | 0.0544 | 380.96 | 552.55 | 171.78 | 143.18 | 316.97 | 185.73 | 175.01 | 382.19 | (4, 1), (3, 2), (4, 2), (6, 2), (4, 8) |
| [214841_at](https://www.affymetrix.com/LinkServlet?probeset=214841_at) | [CNIH3](http://www.ncbi.nlm.nih.gov/entrez/query.fcgi?cmd=search&db=gene&term=CNIH3) | cornichon homolog 3 (Drosophila) | 0.0007284 | 0.0544 | 271.81 | 367.87 | 103.54 | 68.16 | 73.62 | 122.88 | 72.34 | 204.68 | (4, 1), (5, 1), (3, 2), (4, 2), (5, 2), (6, 2), (7, 2) |
| [244745_at](https://www.affymetrix.com/LinkServlet?probeset=244745_at) | [RERG](http://www.ncbi.nlm.nih.gov/entrez/query.fcgi?cmd=search&db=gene&term=RERG) | RAS-like, estrogen-regulated, growth inhibitor | 0.0007287 | 0.0544 | 6.63 | 8.23 | 5.77 | 4.76 | 4.78 | 4.75 | 27.5 | 5.04 | (1, 7), (2, 7), (3, 7), (4, 7), (5, 7), (6, 7), (8, 7) |
| [218775_s_at](https://www.affymetrix.com/LinkServlet?probeset=218775_s_at) | [WWC2](http://www.ncbi.nlm.nih.gov/entrez/query.fcgi?cmd=search&db=gene&term=WWC2) | WW and C2 domain containing 2 | 0.0007309 | 0.0544 | 49.99 | 28.24 | 35.98 | 59.76 | 36.41 | 78.63 | 46.09 | 25.45 | (2, 4), (2, 6), (3, 6), (8, 4), (5, 6), (8, 6) |
| [225174_at](https://www.affymetrix.com/LinkServlet?probeset=225174_at) | [DNAJC10](http://www.ncbi.nlm.nih.gov/entrez/query.fcgi?cmd=search&db=gene&term=DNAJC10) | DnaJ (Hsp40) homolog, subfamily C, member 10 | 0.0007327 | 0.0544 | 921.3 | 1397.14 | 755.45 | 837.75 | 1035.58 | 1032.02 | 637.41 | 1193.2 | (1, 2), (3, 2), (4, 2), (7, 2), (3, 8), (7, 8) |
| [1563494_at](https://www.affymetrix.com/LinkServlet?probeset=1563494_at) | [NA](http://www.ncbi.nlm.nih.gov/entrez/query.fcgi?cmd=search&db=gene&term=NA) | NA | 0.0007425 | 0.055 | 7.14 | 7.74 | 15.87 | 44.84 | 11.03 | 21.31 | 12.59 | 15.65 | (1, 4), (1, 6), (2, 4), (5, 4) |
| [205126_at](https://www.affymetrix.com/LinkServlet?probeset=205126_at) | [VRK2](http://www.ncbi.nlm.nih.gov/entrez/query.fcgi?cmd=search&db=gene&term=VRK2) | vaccinia related kinase 2 | 0.0007509 | 0.0552 | 111.48 | 300.08 | 158.56 | 156.31 | 149.82 | 174.33 | 102.5 | 144.83 | (1, 2), (3, 2), (4, 2), (5, 2), (7, 2), (8, 2) |
| [238846_at](https://www.affymetrix.com/LinkServlet?probeset=238846_at) | [TNFRSF11A](http://www.ncbi.nlm.nih.gov/entrez/query.fcgi?cmd=search&db=gene&term=TNFRSF11A) | tumor necrosis factor receptor superfamily, member 11a, NFKB activator | 0.0007543 | 0.0552 | 60.41 | 109.67 | 25.42 | 27.78 | 42.98 | 35.75 | 38.86 | 77.82 | (3, 2), (4, 2), (5, 2), (6, 2), (3, 8), (4, 8) |
| [232805_at](https://www.affymetrix.com/LinkServlet?probeset=232805_at) | [NA](http://www.ncbi.nlm.nih.gov/entrez/query.fcgi?cmd=search&db=gene&term=NA) | NA | 0.0007557 | 0.0552 | 41.56 | 173.62 | 237.27 | 470.08 | 174.2 | 317.19 | 76.49 | 86.37 | (1, 2), (1, 3), (1, 4), (1, 5), (1, 6), (8, 4) |
| [234317_s_at](https://www.affymetrix.com/LinkServlet?probeset=234317_s_at) | [STOX2](http://www.ncbi.nlm.nih.gov/entrez/query.fcgi?cmd=search&db=gene&term=STOX2) | storkhead box 2 | 0.0007559 | 0.0552 | 152.96 | 70.12 | 166.21 | 189.94 | 179.92 | 164.74 | 231.51 | 144.77 | (2, 1), (2, 3), (2, 4), (2, 5), (2, 6), (2, 7), (2, 8) |
| [238022_at](https://www.affymetrix.com/LinkServlet?probeset=238022_at) | [CRNDE](http://www.ncbi.nlm.nih.gov/entrez/query.fcgi?cmd=search&db=gene&term=CRNDE) | colorectal neoplasia differentially expressed (non-protein coding) | 0.0007593 | 0.0553 | 14.43 | 46.42 | 102.65 | 145.03 | 156.61 | 108.26 | 47.22 | 72.15 | (1, 3), (1, 4), (1, 5), (1, 6), (1, 8) |
| [221590_s_at](https://www.affymetrix.com/LinkServlet?probeset=221590_s_at) | [NA](http://www.ncbi.nlm.nih.gov/entrez/query.fcgi?cmd=search&db=gene&term=NA) | NA | 0.0007617 | 0.0553 | 121.27 | 68.65 | 138.67 | 119.31 | 135.88 | 183.22 | 267.44 | 108.76 | (2, 1), (2, 3), (2, 5), (2, 6), (2, 7), (8, 7) |
| [201571_s_at](https://www.affymetrix.com/LinkServlet?probeset=201571_s_at) | [DCTD](http://www.ncbi.nlm.nih.gov/entrez/query.fcgi?cmd=search&db=gene&term=DCTD) | dCMP deaminase | 0.0007651 | 0.0553 | 86.23 | 76.14 | 28.44 | 30.8 | 65.4 | 32.08 | 61.36 | 58.83 | (3, 1), (4, 1), (6, 1), (3, 2), (4, 2), (6, 2), (3, 5) |
| [1555216_a_at](https://www.affymetrix.com/LinkServlet?probeset=1555216_a_at) | [LOC645722](http://www.ncbi.nlm.nih.gov/entrez/query.fcgi?cmd=search&db=gene&term=LOC645722) | hypothetical LOC645722 | 0.0007721 | 0.0556 | 12.74 | 6.14 | 5.02 | 4.77 | 5.32 | 4.77 | 15.99 | 6.21 | (2, 1), (3, 1), (4, 1), (5, 1), (6, 1), (8, 1), (3, 7), (4, 7), (5, 7), (6, 7) |
| [215380_s_at](https://www.affymetrix.com/LinkServlet?probeset=215380_s_at) | [GGCT](http://www.ncbi.nlm.nih.gov/entrez/query.fcgi?cmd=search&db=gene&term=GGCT) | gamma-glutamyl cyclotransferase | 0.0007768 | 0.0557 | 1758.56 | 1724.52 | 1076.15 | 876.46 | 1586.34 | 1077.26 | 1906.36 | 1438.09 | (3, 1), (4, 1), (6, 1), (3, 2), (4, 2), (6, 2), (4, 5), (4, 7) |
| [212588_at](https://www.affymetrix.com/LinkServlet?probeset=212588_at) | [PTPRC](http://www.ncbi.nlm.nih.gov/entrez/query.fcgi?cmd=search&db=gene&term=PTPRC) | protein tyrosine phosphatase, receptor type, C | 0.0007805 | 0.0557 | 373.12 | 885.46 | 156.04 | 291.05 | 693.29 | 447.22 | 209.77 | 572.78 | (3, 2), (4, 2), (7, 2), (3, 5), (3, 6), (3, 8) |
| [203608_at](https://www.affymetrix.com/LinkServlet?probeset=203608_at) | [ALDH5A1](http://www.ncbi.nlm.nih.gov/entrez/query.fcgi?cmd=search&db=gene&term=ALDH5A1) | aldehyde dehydrogenase 5 family, member A1 | 0.0007873 | 0.0557 | 872.34 | 801.43 | 1600.39 | 1698.1 | 1453.36 | 1763.92 | 2600.74 | 1318.38 | (1, 4), (1, 6), (1, 7), (2, 3), (2, 4), (2, 5), (2, 6), (2, 7) |
| [213083_at](https://www.affymetrix.com/LinkServlet?probeset=213083_at) | [SLC35D2](http://www.ncbi.nlm.nih.gov/entrez/query.fcgi?cmd=search&db=gene&term=SLC35D2) | solute carrier family 35, member D2 | 0.0007883 | 0.0557 | 357.15 | 534.28 | 265.47 | 238.17 | 314.62 | 342.76 | 156.9 | 325.93 | (7, 1), (3, 2), (4, 2), (5, 2), (7, 2), (7, 6) |
| [203869_at](https://www.affymetrix.com/LinkServlet?probeset=203869_at) | [USP46](http://www.ncbi.nlm.nih.gov/entrez/query.fcgi?cmd=search&db=gene&term=USP46) | ubiquitin specific peptidase 46 | 0.0007894 | 0.0557 | 223.4 | 183.48 | 246.16 | 339.43 | 394.1 | 312.55 | 686.6 | 341.02 | (1, 7), (2, 4), (2, 5), (2, 7), (2, 8), (3, 7) |
| [242628_at](https://www.affymetrix.com/LinkServlet?probeset=242628_at) | [KLRB1](http://www.ncbi.nlm.nih.gov/entrez/query.fcgi?cmd=search&db=gene&term=KLRB1) | killer cell lectin-like receptor subfamily B, member 1 | 0.0007919 | 0.0557 | 10.68 | 11.67 | 28.69 | 45.08 | 24.52 | 53.42 | 9.76 | 8.8 | (1, 4), (1, 6), (2, 4), (2, 6), (8, 4), (8, 6) |
| [210461_s_at](https://www.affymetrix.com/LinkServlet?probeset=210461_s_at) | [ABLIM1](http://www.ncbi.nlm.nih.gov/entrez/query.fcgi?cmd=search&db=gene&term=ABLIM1) | actin binding LIM protein 1 | 0.0007927 | 0.0557 | 199.2 | 203.96 | 468.98 | 543.32 | 272.09 | 560.14 | 474.18 | 395.42 | (1, 3), (1, 4), (1, 6), (2, 3), (2, 4), (2, 6) |
| [230179_at](https://www.affymetrix.com/LinkServlet?probeset=230179_at) | [LOC285812](http://www.ncbi.nlm.nih.gov/entrez/query.fcgi?cmd=search&db=gene&term=LOC285812) | hypothetical protein LOC285812 | 0.0007936 | 0.0557 | 297.57 | 110.73 | 304.94 | 246.13 | 238.06 | 234.07 | 525.46 | 144.98 | (2, 1), (2, 3), (2, 4), (2, 5), (2, 6), (2, 7), (8, 7) |
| [204290_s_at](https://www.affymetrix.com/LinkServlet?probeset=204290_s_at) | [ALDH6A1](http://www.ncbi.nlm.nih.gov/entrez/query.fcgi?cmd=search&db=gene&term=ALDH6A1) | aldehyde dehydrogenase 6 family, member A1 | 0.0008015 | 0.0561 | 196.63 | 100.35 | 195.76 | 201.24 | 215.97 | 257.46 | 454.85 | 177.54 | (2, 1), (2, 3), (2, 4), (2, 5), (2, 6), (2, 7), (8, 7) |
| [207013_s_at](https://www.affymetrix.com/LinkServlet?probeset=207013_s_at) | [MMP16](http://www.ncbi.nlm.nih.gov/entrez/query.fcgi?cmd=search&db=gene&term=MMP16) | matrix metallopeptidase 16 (membrane-inserted) | 0.0008267 | 0.0576 | 16.77 | 27.68 | 26.17 | 31.35 | 33.63 | 40.17 | 15.1 | 20.82 | (1, 2), (1, 4), (1, 5), (1, 6), (7, 5), (7, 6), (8, 6) |
| [217820_s_at](https://www.affymetrix.com/LinkServlet?probeset=217820_s_at) | [ENAH](http://www.ncbi.nlm.nih.gov/entrez/query.fcgi?cmd=search&db=gene&term=ENAH) | enabled homolog (Drosophila) | 0.0008275 | 0.0576 | 3377.58 | 2220.53 | 3131.61 | 4975.43 | 3703.96 | 4319.23 | 3143.47 | 3562.19 | (2, 1), (2, 4), (2, 5), (2, 6), (2, 8), (3, 4) |
| [213351_s_at](https://www.affymetrix.com/LinkServlet?probeset=213351_s_at) | [TMCC1](http://www.ncbi.nlm.nih.gov/entrez/query.fcgi?cmd=search&db=gene&term=TMCC1) | transmembrane and coiled-coil domain family 1 | 0.0008337 | 0.0578 | 418.59 | 278.19 | 599.27 | 849.97 | 483.07 | 731.76 | 723.35 | 409.81 | (1, 4), (2, 3), (2, 4), (2, 6), (2, 7), (8, 4) |
| [240743_at](https://www.affymetrix.com/LinkServlet?probeset=240743_at) | [NA](http://www.ncbi.nlm.nih.gov/entrez/query.fcgi?cmd=search&db=gene&term=NA) | NA | 0.0008446 | 0.0584 | 8.15 | 7.43 | 15.18 | 19.11 | 8.51 | 8.66 | 11.63 | 8.33 | (1, 3), (1, 4), (2, 3), (2, 4), (5, 4), (6, 4), (8, 4) |
| [239767_at](https://www.affymetrix.com/LinkServlet?probeset=239767_at) | [NA](http://www.ncbi.nlm.nih.gov/entrez/query.fcgi?cmd=search&db=gene&term=NA) | NA | 0.0008493 | 0.0585 | 32.96 | 7.15 | 5.85 | 5.49 | 4.98 | 5.33 | 37.36 | 7.72 | (2, 1), (3, 1), (4, 1), (5, 1), (6, 1), (8, 1), (3, 7), (4, 7), (5, 7), (6, 7) |
| [201939_at](https://www.affymetrix.com/LinkServlet?probeset=201939_at) | [PLK2](http://www.ncbi.nlm.nih.gov/entrez/query.fcgi?cmd=search&db=gene&term=PLK2) | polo-like kinase 2 (Drosophila) | 0.0008528 | 0.0586 | 585.69 | 349.39 | 411.3 | 188.74 | 154.28 | 159.39 | 218.47 | 455.17 | (4, 1), (5, 1), (6, 1), (5, 3), (6, 3), (5, 8), (6, 8) |
| [206584_at](https://www.affymetrix.com/LinkServlet?probeset=206584_at) | [LY96](http://www.ncbi.nlm.nih.gov/entrez/query.fcgi?cmd=search&db=gene&term=LY96) | lymphocyte antigen 96 | 0.0008683 | 0.0593 | 729.46 | 1026.74 | 253 | 284.12 | 527.73 | 422.15 | 197.69 | 824.97 | (3, 1), (3, 2), (4, 2), (7, 2), (3, 8), (4, 8) |
| [204924_at](https://www.affymetrix.com/LinkServlet?probeset=204924_at) | [TLR2](http://www.ncbi.nlm.nih.gov/entrez/query.fcgi?cmd=search&db=gene&term=TLR2) | toll-like receptor 2 | 0.0008686 | 0.0593 | 107.56 | 192.42 | 29.37 | 46.51 | 172.48 | 78.8 | 57.49 | 118.01 | (3, 1), (3, 2), (4, 2), (3, 5), (3, 8), (4, 5) |
| [223843_at](https://www.affymetrix.com/LinkServlet?probeset=223843_at) | [SCARA3](http://www.ncbi.nlm.nih.gov/entrez/query.fcgi?cmd=search&db=gene&term=SCARA3) | scavenger receptor class A, member 3 | 0.0008797 | 0.0597 | 69.43 | 69.67 | 124.63 | 212.28 | 307.04 | 359.64 | 66.43 | 137.49 | (1, 5), (1, 6), (2, 5), (2, 6), (7, 6) |
| [204215_at](https://www.affymetrix.com/LinkServlet?probeset=204215_at) | [C7orf23](http://www.ncbi.nlm.nih.gov/entrez/query.fcgi?cmd=search&db=gene&term=C7orf23) | chromosome 7 open reading frame 23 | 0.0008805 | 0.0597 | 253.82 | 456.58 | 132.5 | 152.06 | 231.15 | 200.05 | 153.71 | 297.07 | (3, 2), (4, 2), (6, 2), (7, 2), (3, 8) |
| [215626_at](https://www.affymetrix.com/LinkServlet?probeset=215626_at) | [NA](http://www.ncbi.nlm.nih.gov/entrez/query.fcgi?cmd=search&db=gene&term=NA) | NA | 0.0008859 | 0.0599 | 8.91 | 9.45 | 11.06 | 28.87 | 12.17 | 21.91 | 11.1 | 9.7 | (1, 4), (1, 6), (2, 4), (2, 6), (3, 4), (5, 4), (8, 4) |
| [213429_at](https://www.affymetrix.com/LinkServlet?probeset=213429_at) | [NA](http://www.ncbi.nlm.nih.gov/entrez/query.fcgi?cmd=search&db=gene&term=NA) | NA | 0.0008938 | 0.06 | 42.83 | 175.53 | 19.06 | 30.13 | 37.83 | 30.22 | 17.13 | 33.38 | (1, 2), (3, 2), (4, 2), (5, 2), (6, 2), (7, 2), (8, 2) |
| [233823_at](https://www.affymetrix.com/LinkServlet?probeset=233823_at) | [FAM184B](http://www.ncbi.nlm.nih.gov/entrez/query.fcgi?cmd=search&db=gene&term=FAM184B) | family with sequence similarity 184, member B | 0.0008956 | 0.06 | 40.6 | 8.29 | 34.32 | 28.06 | 33.21 | 50.93 | 138.75 | 22.23 | (2, 1), (2, 3), (2, 4), (2, 5), (2, 6), (2, 7), (8, 7) |
| [219501_at](https://www.affymetrix.com/LinkServlet?probeset=219501_at) | [ENOX1](http://www.ncbi.nlm.nih.gov/entrez/query.fcgi?cmd=search&db=gene&term=ENOX1) | ecto-NOX disulfide-thiol exchanger 1 | 0.0008959 | 0.06 | 69.31 | 43.48 | 130.03 | 127.64 | 111.02 | 125.95 | 128.95 | 44.85 | (2, 3), (2, 4), (2, 5), (2, 6), (8, 3), (8, 4), (8, 5), (8, 6) |
| [217282_at](https://www.affymetrix.com/LinkServlet?probeset=217282_at) | [MAN1A2](http://www.ncbi.nlm.nih.gov/entrez/query.fcgi?cmd=search&db=gene&term=MAN1A2) | mannosidase, alpha, class 1A, member 2 | 0.0009019 | 0.0601 | 8.03 | 8.45 | 23.56 | 26.11 | 11.67 | 10.47 | 10.46 | 13.1 | (1, 3), (1, 4), (2, 3), (2, 4), (6, 3), (6, 4) |
| [226374_at](https://www.affymetrix.com/LinkServlet?probeset=226374_at) | [NA](http://www.ncbi.nlm.nih.gov/entrez/query.fcgi?cmd=search&db=gene&term=NA) | NA | 0.0009025 | 0.0601 | 2390.67 | 1007.33 | 924.27 | 497.97 | 860.47 | 985.36 | 1255.97 | 1256.65 | (2, 1), (3, 1), (4, 1), (5, 1), (6, 1), (4, 8) |
| [231969_at](https://www.affymetrix.com/LinkServlet?probeset=231969_at) | [STOX2](http://www.ncbi.nlm.nih.gov/entrez/query.fcgi?cmd=search&db=gene&term=STOX2) | storkhead box 2 | 0.0009143 | 0.0605 | 630.19 | 302.5 | 808.1 | 1047.03 | 598.22 | 672.89 | 763.08 | 578.03 | (2, 1), (2, 3), (2, 4), (2, 5), (2, 6) |
| [1556462_a_at](https://www.affymetrix.com/LinkServlet?probeset=1556462_a_at) | [NA](http://www.ncbi.nlm.nih.gov/entrez/query.fcgi?cmd=search&db=gene&term=NA) | NA | 0.0009147 | 0.0605 | 9.36 | 13.36 | 24.74 | 30.22 | 15.17 | 50.41 | 15.93 | 14.38 | (1, 3), (1, 4), (1, 6), (2, 6), (5, 6), (8, 6) |
| [219450_at](https://www.affymetrix.com/LinkServlet?probeset=219450_at) | [C4orf19](http://www.ncbi.nlm.nih.gov/entrez/query.fcgi?cmd=search&db=gene&term=C4orf19) | chromosome 4 open reading frame 19 | 0.0009165 | 0.0605 | 36.81 | 13.98 | 31.15 | 50.63 | 44.36 | 42.2 | 283.66 | 21.81 | (1, 7), (2, 4), (2, 5), (2, 6), (2, 7), (3, 7), (4, 7), (5, 7), (6, 7), (8, 7) |
| [208813_at](https://www.affymetrix.com/LinkServlet?probeset=208813_at) | [GOT1](http://www.ncbi.nlm.nih.gov/entrez/query.fcgi?cmd=search&db=gene&term=GOT1) | glutamic-oxaloacetic transaminase 1, soluble (aspartate aminotransferase 1) | 0.0009206 | 0.0605 | 242.12 | 122.74 | 183.37 | 88.37 | 168.38 | 127.92 | 223.21 | 145.94 | (2, 1), (4, 1), (6, 1), (4, 3), (4, 5), (4, 7) |
| [244413_at](https://www.affymetrix.com/LinkServlet?probeset=244413_at) | [CLECL1](http://www.ncbi.nlm.nih.gov/entrez/query.fcgi?cmd=search&db=gene&term=CLECL1) | C-type lectin-like 1 | 0.0009226 | 0.0605 | 55.49 | 147.09 | 21.84 | 30.92 | 63.21 | 62.56 | 14.87 | 56.08 | (3, 2), (4, 2), (7, 2) |
| [205823_at](https://www.affymetrix.com/LinkServlet?probeset=205823_at) | [RGS12](http://www.ncbi.nlm.nih.gov/entrez/query.fcgi?cmd=search&db=gene&term=RGS12) | regulator of G-protein signaling 12 | 0.0009255 | 0.0605 | 110.74 | 69.37 | 54.57 | 81.49 | 51.04 | 65.31 | 133.79 | 77.27 | (2, 1), (3, 1), (5, 1), (6, 1), (3, 7), (5, 7) |
| [207336_at](https://www.affymetrix.com/LinkServlet?probeset=207336_at) | [SOX5](http://www.ncbi.nlm.nih.gov/entrez/query.fcgi?cmd=search&db=gene&term=SOX5) | SRY (sex determining region Y)-box 5 | 0.0009376 | 0.0611 | 25.48 | 24 | 50.64 | 68.26 | 39.68 | 84.39 | 37.2 | 19.84 | (1, 4), (1, 6), (2, 4), (2, 6), (8, 4), (8, 6) |
| [236902_at](https://www.affymetrix.com/LinkServlet?probeset=236902_at) | [FLJ43390](http://www.ncbi.nlm.nih.gov/entrez/query.fcgi?cmd=search&db=gene&term=FLJ43390) | hypothetical LOC646113 | 0.0009394 | 0.0611 | 13.22 | 7.22 | 38.41 | 95.6 | 49.45 | 53.7 | 39.74 | 18.57 | (1, 4), (2, 3), (2, 4), (2, 5), (2, 6) |
| [1552798_a_at](https://www.affymetrix.com/LinkServlet?probeset=1552798_a_at) | [TLR4](http://www.ncbi.nlm.nih.gov/entrez/query.fcgi?cmd=search&db=gene&term=TLR4) | toll-like receptor 4 | 0.0009558 | 0.0618 | 9.7 | 17.24 | 6.7 | 6.99 | 8.81 | 8.27 | 7.65 | 9.97 | (1, 2), (3, 2), (4, 2), (5, 2), (6, 2), (7, 2) |
| [202341_s_at](https://www.affymetrix.com/LinkServlet?probeset=202341_s_at) | [TRIM2](http://www.ncbi.nlm.nih.gov/entrez/query.fcgi?cmd=search&db=gene&term=TRIM2) | tripartite motif-containing 2 | 0.000959 | 0.0618 | 3172.87 | 1374.66 | 3594.15 | 3678.89 | 2182.24 | 2937.49 | 3800.15 | 2227.54 | (2, 1), (2, 3), (2, 4), (2, 6), (2, 7) |
| [244011_at](https://www.affymetrix.com/LinkServlet?probeset=244011_at) | [PPM1K](http://www.ncbi.nlm.nih.gov/entrez/query.fcgi?cmd=search&db=gene&term=PPM1K) | protein phosphatase 1K (PP2C domain containing) | 0.0009596 | 0.0618 | 15.14 | 14.66 | 23.81 | 28.29 | 16.74 | 16.76 | 18.48 | 15.67 | (1, 3), (1, 4), (2, 3), (2, 4), (5, 4), (6, 4), (8, 4) |
| [1556205_at](https://www.affymetrix.com/LinkServlet?probeset=1556205_at) | [QDPR](http://www.ncbi.nlm.nih.gov/entrez/query.fcgi?cmd=search&db=gene&term=QDPR) | quinoid dihydropteridine reductase | 0.0009677 | 0.0622 | 68.22 | 25.81 | 46.16 | 43.18 | 23.79 | 36.77 | 94.4 | 41.78 | (2, 1), (5, 1), (2, 7), (5, 7) |
| [206132_at](https://www.affymetrix.com/LinkServlet?probeset=206132_at) | [MCC](http://www.ncbi.nlm.nih.gov/entrez/query.fcgi?cmd=search&db=gene&term=MCC) | mutated in colorectal cancers | 0.0009801 | 0.0628 | 7.27 | 5.04 | 5.44 | 4.96 | 6.25 | 6.59 | 16.57 | 5.53 | (1, 7), (2, 7), (3, 7), (4, 7), (5, 7), (6, 7), (8, 7) |
| [203618_at](https://www.affymetrix.com/LinkServlet?probeset=203618_at) | [FAIM2](http://www.ncbi.nlm.nih.gov/entrez/query.fcgi?cmd=search&db=gene&term=FAIM2) | Fas apoptotic inhibitory molecule 2 | 0.000986 | 0.0629 | 29.91 | 18 | 45.89 | 28.1 | 26.58 | 30.64 | 58.48 | 29.37 | (2, 3), (2, 6), (2, 7) |
| [219451_at](https://www.affymetrix.com/LinkServlet?probeset=219451_at) | [MSRB2](http://www.ncbi.nlm.nih.gov/entrez/query.fcgi?cmd=search&db=gene&term=MSRB2) | methionine sulfoxide reductase B2 | 0.0009902 | 0.0629 | 454.3 | 364.63 | 680.99 | 831.82 | 506.22 | 651.9 | 640.45 | 491.69 | (1, 4), (2, 3), (2, 4), (2, 6), (8, 4) |
| [219821_s_at](https://www.affymetrix.com/LinkServlet?probeset=219821_s_at) | [GFOD1](http://www.ncbi.nlm.nih.gov/entrez/query.fcgi?cmd=search&db=gene&term=GFOD1) | glucose-fructose oxidoreductase domain containing 1 | 0.0009907 | 0.0629 | 204.64 | 78.77 | 256.16 | 205.4 | 143.34 | 151.5 | 268.99 | 127.64 | (2, 1), (2, 3), (2, 4), (2, 7) |
