# Supplementary material for: Transcriptional profiles of pilocytic astrocytoma are related to their three different locations, but not to radiological tumor features
Source: BMC Cancer. 2015 Oct 24;15:778. doi: 10.1186/s12885-015-1810-z (PMC4619381; doi:10.1186/s12885-015-1810-z)
Supplement: Additional file 3: Table S3. — List of genes differentiating between pilocytic astrocytomas in the three most frequently observed locations. Comparison of three subgroups. FDR, false discovery rate; M1, cerebral hemispheric tumor; M2, optic tract and hypothalamic tumor; M3, cerebellar tumor (DOCX 169 kb) [file 12885_2015_1810_MOESM3_ESM.docx]

| **Probe set** | **Gene symbol** | **Description** | **Parametric *p*-value** | **FDR** | **Geom mean of intensities in class M1** | **Geom mean of intensities in class M2** | **Geom mean of intensities in class M3** | **Pairwise significant** |
| --- | --- | --- | --- | --- | --- | --- | --- | --- |
| [228462_at](https://www.affymetrix.com/LinkServlet?probeset=228462_at) | [IRX2](http://www.ncbi.nlm.nih.gov/entrez/query.fcgi?cmd=search&db=gene&term=IRX2) | iroquois homeobox 2 | < 1e-07 | < 1e-07 | 7.81 | 36.27 | 470.74 | (1, 2), (1, 3), (2, 3) |
| [231666_at](https://www.affymetrix.com/LinkServlet?probeset=231666_at) | [PAX3](http://www.ncbi.nlm.nih.gov/entrez/query.fcgi?cmd=search&db=gene&term=PAX3) | paired box 3 | < 1e-07 | < 1e-07 | 4.97 | 7.59 | 279.28 | (1, 3), (2, 3) |
| [207250_at](https://www.affymetrix.com/LinkServlet?probeset=207250_at) | [SIX6](http://www.ncbi.nlm.nih.gov/entrez/query.fcgi?cmd=search&db=gene&term=SIX6) | SIX homeobox 6 | < 1e-07 | < 1e-07 | 7.32 | 246.4 | 4.75 | (1, 2), (3, 2) |
| [206140_at](https://www.affymetrix.com/LinkServlet?probeset=206140_at) | [LHX2](http://www.ncbi.nlm.nih.gov/entrez/query.fcgi?cmd=search&db=gene&term=LHX2) | LIM homeobox 2 | < 1e-07 | < 1e-07 | 1680.77 | 2642.73 | 11.02 | (3, 1), (3, 2) |
| [211219_s_at](https://www.affymetrix.com/LinkServlet?probeset=211219_s_at) | [LHX2](http://www.ncbi.nlm.nih.gov/entrez/query.fcgi?cmd=search&db=gene&term=LHX2) | LIM homeobox 2 | < 1e-07 | < 1e-07 | 80.88 | 93.18 | 5.49 | (3, 1), (3, 2) |
| [223582_at](https://www.affymetrix.com/LinkServlet?probeset=223582_at) | [GPR98](http://www.ncbi.nlm.nih.gov/entrez/query.fcgi?cmd=search&db=gene&term=GPR98) | G protein-coupled receptor 98 | < 1e-07 | < 1e-07 | 50.94 | 146.32 | 15.08 | (1, 2), (3, 1), (3, 2) |
| [1561985_at](https://www.affymetrix.com/LinkServlet?probeset=1561985_at) | [C14orf39](http://www.ncbi.nlm.nih.gov/entrez/query.fcgi?cmd=search&db=gene&term=C14orf39) | chromosome 14 open reading frame 39 | < 1e-07 | < 1e-07 | 6.82 | 88.41 | 4.76 | (1, 2), (3, 2) |
| [1554784_at](https://www.affymetrix.com/LinkServlet?probeset=1554784_at) | [CNTN1](http://www.ncbi.nlm.nih.gov/entrez/query.fcgi?cmd=search&db=gene&term=CNTN1) | contactin 1 | < 1e-07 | < 1e-07 | 68.37 | 17.57 | 379.96 | (2, 1), (1, 3), (2, 3) |
| [230720_at](https://www.affymetrix.com/LinkServlet?probeset=230720_at) | [RNF182](http://www.ncbi.nlm.nih.gov/entrez/query.fcgi?cmd=search&db=gene&term=RNF182) | ring finger protein 182 | < 1e-07 | < 1e-07 | 171.48 | 1797.68 | 2061.29 | (1, 2), (1, 3) |
| [210239_at](https://www.affymetrix.com/LinkServlet?probeset=210239_at) | [IRX5](http://www.ncbi.nlm.nih.gov/entrez/query.fcgi?cmd=search&db=gene&term=IRX5) | iroquois homeobox 5 | < 1e-07 | < 1e-07 | 7.84 | 25.89 | 100.36 | (1, 2), (1, 3), (2, 3) |
| [213285_at](https://www.affymetrix.com/LinkServlet?probeset=213285_at) | [TMEM30B](http://www.ncbi.nlm.nih.gov/entrez/query.fcgi?cmd=search&db=gene&term=TMEM30B) | transmembrane protein 30B | < 1e-07 | < 1e-07 | 7.23 | 26.44 | 5.08 | (1, 2), (3, 2) |
| [238727_at](https://www.affymetrix.com/LinkServlet?probeset=238727_at) | [LOC440934](http://www.ncbi.nlm.nih.gov/entrez/query.fcgi?cmd=search&db=gene&term=LOC440934) | hypothetical LOC440934 | < 1e-07 | < 1e-07 | 26.05 | 13.66 | 258.93 | (1, 3), (2, 3) |
| [230472_at](https://www.affymetrix.com/LinkServlet?probeset=230472_at) | [IRX1](http://www.ncbi.nlm.nih.gov/entrez/query.fcgi?cmd=search&db=gene&term=IRX1) | iroquois homeobox 1 | < 1e-07 | < 1e-07 | 10.8 | 66.79 | 205.19 | (1, 2), (1, 3), (2, 3) |
| [242054_s_at](https://www.affymetrix.com/LinkServlet?probeset=242054_s_at) | [NA](http://www.ncbi.nlm.nih.gov/entrez/query.fcgi?cmd=search&db=gene&term=NA) | NA | < 1e-07 | < 1e-07 | 5 | 82.65 | 4.9 | (1, 2), (3, 2) |
| [227202_at](https://www.affymetrix.com/LinkServlet?probeset=227202_at) | [CNTN1](http://www.ncbi.nlm.nih.gov/entrez/query.fcgi?cmd=search&db=gene&term=CNTN1) | contactin 1 | < 1e-07 | < 1e-07 | 262.72 | 51.46 | 1717.72 | (2, 1), (1, 3), (2, 3) |
| [214954_at](https://www.affymetrix.com/LinkServlet?probeset=214954_at) | [SUSD5](http://www.ncbi.nlm.nih.gov/entrez/query.fcgi?cmd=search&db=gene&term=SUSD5) | sushi domain containing 5 | < 1e-07 | < 1e-07 | 125.14 | 1108.04 | 1603.52 | (1, 2), (1, 3) |
| [208464_at](https://www.affymetrix.com/LinkServlet?probeset=208464_at) | [GRIA4](http://www.ncbi.nlm.nih.gov/entrez/query.fcgi?cmd=search&db=gene&term=GRIA4) | glutamate receptor, ionotrophic, AMPA 4 | < 1e-07 | < 1e-07 | 5.9 | 16.53 | 41.53 | (1, 2), (1, 3), (2, 3) |
| [206018_at](https://www.affymetrix.com/LinkServlet?probeset=206018_at) | [FOXG1](http://www.ncbi.nlm.nih.gov/entrez/query.fcgi?cmd=search&db=gene&term=FOXG1) | forkhead box G1 | < 1e-07 | < 1e-07 | 1197.42 | 11.25 | 4.93 | (2, 1), (3, 1) |
| [208221_s_at](https://www.affymetrix.com/LinkServlet?probeset=208221_s_at) | [SLIT1](http://www.ncbi.nlm.nih.gov/entrez/query.fcgi?cmd=search&db=gene&term=SLIT1) | slit homolog 1 (Drosophila) | < 1e-07 | < 1e-07 | 8.42 | 17.8 | 6 | (1, 2), (3, 2) |
| [238021_s_at](https://www.affymetrix.com/LinkServlet?probeset=238021_s_at) | [CRNDE](http://www.ncbi.nlm.nih.gov/entrez/query.fcgi?cmd=search&db=gene&term=CRNDE) | colorectal neoplasia differentially expressed (non-protein coding) | < 1e-07 | < 1e-07 | 51.09 | 382.35 | 1216.52 | (1, 2), (1, 3), (2, 3) |
| [226448_at](https://www.affymetrix.com/LinkServlet?probeset=226448_at) | [FAM89A](http://www.ncbi.nlm.nih.gov/entrez/query.fcgi?cmd=search&db=gene&term=FAM89A) | family with sequence similarity 89, member A | < 1e-07 | < 1e-07 | 104 | 625.78 | 808.9 | (1, 2), (1, 3) |
| [229831_at](https://www.affymetrix.com/LinkServlet?probeset=229831_at) | [CNTN3](http://www.ncbi.nlm.nih.gov/entrez/query.fcgi?cmd=search&db=gene&term=CNTN3) | contactin 3 (plasmacytoma associated) | < 1e-07 | < 1e-07 | 21.57 | 9.97 | 176.8 | (1, 3), (2, 3) |
| [228347_at](https://www.affymetrix.com/LinkServlet?probeset=228347_at) | [SIX1](http://www.ncbi.nlm.nih.gov/entrez/query.fcgi?cmd=search&db=gene&term=SIX1) | SIX homeobox 1 | < 1e-07 | < 1e-07 | 13.8 | 421.71 | 9.17 | (1, 2), (3, 2) |
| [206634_at](https://www.affymetrix.com/LinkServlet?probeset=206634_at) | [SIX3](http://www.ncbi.nlm.nih.gov/entrez/query.fcgi?cmd=search&db=gene&term=SIX3) | SIX homeobox 3 | < 1e-07 | < 1e-07 | 4.86 | 49.47 | 4.75 | (1, 2), (3, 2) |
| [238022_at](https://www.affymetrix.com/LinkServlet?probeset=238022_at) | [CRNDE](http://www.ncbi.nlm.nih.gov/entrez/query.fcgi?cmd=search&db=gene&term=CRNDE) | colorectal neoplasia differentially expressed (non-protein coding) | < 1e-07 | < 1e-07 | 14 | 51.83 | 134.25 | (1, 2), (1, 3), (2, 3) |
| [1554507_at](https://www.affymetrix.com/LinkServlet?probeset=1554507_at) | [NAALAD2](http://www.ncbi.nlm.nih.gov/entrez/query.fcgi?cmd=search&db=gene&term=NAALAD2) | N-acetylated alpha-linked acidic dipeptidase 2 | < 1e-07 | < 1e-07 | 10.63 | 8.94 | 69.52 | (1, 3), (2, 3) |
| [238878_at](https://www.affymetrix.com/LinkServlet?probeset=238878_at) | [ARX](http://www.ncbi.nlm.nih.gov/entrez/query.fcgi?cmd=search&db=gene&term=ARX) | aristaless related homeobox | < 1e-07 | < 1e-07 | 102.99 | 6.88 | 4.85 | (2, 1), (3, 1) |
| [227614_at](https://www.affymetrix.com/LinkServlet?probeset=227614_at) | [HKDC1](http://www.ncbi.nlm.nih.gov/entrez/query.fcgi?cmd=search&db=gene&term=HKDC1) | hexokinase domain containing 1 | < 1e-07 | < 1e-07 | 30.54 | 9.56 | 8.08 | (2, 1), (3, 1) |
| [243932_at](https://www.affymetrix.com/LinkServlet?probeset=243932_at) | [NA](http://www.ncbi.nlm.nih.gov/entrez/query.fcgi?cmd=search&db=gene&term=NA) | NA | < 1e-07 | < 1e-07 | 8.86 | 12.57 | 60.51 | (1, 3), (2, 3) |
| [205858_at](https://www.affymetrix.com/LinkServlet?probeset=205858_at) | [NGFR](http://www.ncbi.nlm.nih.gov/entrez/query.fcgi?cmd=search&db=gene&term=NGFR) | nerve growth factor receptor (TNFR superfamily, member 16) | < 1e-07 | < 1e-07 | 135.44 | 53.07 | 14.67 | (3, 1), (3, 2) |
| [227209_at](https://www.affymetrix.com/LinkServlet?probeset=227209_at) | [CNTN1](http://www.ncbi.nlm.nih.gov/entrez/query.fcgi?cmd=search&db=gene&term=CNTN1) | contactin 1 | < 1e-07 | < 1e-07 | 197.22 | 38.14 | 933.25 | (2, 1), (1, 3), (2, 3) |
| [203917_at](https://www.affymetrix.com/LinkServlet?probeset=203917_at) | [CXADR](http://www.ncbi.nlm.nih.gov/entrez/query.fcgi?cmd=search&db=gene&term=CXADR) | coxsackie virus and adenovirus receptor | 1e-07 | 6.44e-05 | 4098.1 | 1363.02 | 1294.12 | (2, 1), (3, 1) |
| [205817_at](https://www.affymetrix.com/LinkServlet?probeset=205817_at) | [SIX1](http://www.ncbi.nlm.nih.gov/entrez/query.fcgi?cmd=search&db=gene&term=SIX1) | SIX homeobox 1 | 1e-07 | 6.44e-05 | 6.82 | 35.96 | 5.16 | (1, 2), (3, 2) |
| [220117_at](https://www.affymetrix.com/LinkServlet?probeset=220117_at) | [ZNF385D](http://www.ncbi.nlm.nih.gov/entrez/query.fcgi?cmd=search&db=gene&term=ZNF385D) | zinc finger protein 385D | 1e-07 | 6.44e-05 | 30.39 | 56.96 | 116.16 | (1, 3), (2, 3) |
| [218974_at](https://www.affymetrix.com/LinkServlet?probeset=218974_at) | [SOBP](http://www.ncbi.nlm.nih.gov/entrez/query.fcgi?cmd=search&db=gene&term=SOBP) | sine oculis binding protein homolog (Drosophila) | 2e-07 | 0.00011 | 1562.82 | 1130.96 | 2529.1 | (1, 3), (2, 3) |
| [236828_at](https://www.affymetrix.com/LinkServlet?probeset=236828_at) | [NA](http://www.ncbi.nlm.nih.gov/entrez/query.fcgi?cmd=search&db=gene&term=NA) | NA | 2e-07 | 0.00011 | 6.31 | 18.98 | 5.07 | (1, 2), (3, 2) |
| [214761_at](https://www.affymetrix.com/LinkServlet?probeset=214761_at) | [ZNF423](http://www.ncbi.nlm.nih.gov/entrez/query.fcgi?cmd=search&db=gene&term=ZNF423) | zinc finger protein 423 | 2e-07 | 0.00011 | 309.11 | 558.19 | 1140.35 | (1, 3), (2, 3) |
| [243440_at](https://www.affymetrix.com/LinkServlet?probeset=243440_at) | [NA](http://www.ncbi.nlm.nih.gov/entrez/query.fcgi?cmd=search&db=gene&term=NA) | NA | 2e-07 | 0.00011 | 5.17 | 31.3 | 4.76 | (1, 2), (3, 2) |
| [235236_at](https://www.affymetrix.com/LinkServlet?probeset=235236_at) | [LOC100131897](http://www.ncbi.nlm.nih.gov/entrez/query.fcgi?cmd=search&db=gene&term=LOC100131897) | Uncharacterized protein LOC100131897 | 2e-07 | 0.00011 | 54.45 | 14.25 | 158.79 | (2, 1), (1, 3), (2, 3) |
| [207443_at](https://www.affymetrix.com/LinkServlet?probeset=207443_at) | [NR2E1](http://www.ncbi.nlm.nih.gov/entrez/query.fcgi?cmd=search&db=gene&term=NR2E1) | nuclear receptor subfamily 2, group E, member 1 | 2e-07 | 0.00011 | 54.05 | 17.91 | 5.02 | (3, 1), (3, 2) |
| [232054_at](https://www.affymetrix.com/LinkServlet?probeset=232054_at) | [PCDH20](http://www.ncbi.nlm.nih.gov/entrez/query.fcgi?cmd=search&db=gene&term=PCDH20) | protocadherin 20 | 3e-07 | 0.000143 | 34.26 | 31.94 | 277.59 | (1, 3), (2, 3) |
| [230008_at](https://www.affymetrix.com/LinkServlet?probeset=230008_at) | [THSD7A](http://www.ncbi.nlm.nih.gov/entrez/query.fcgi?cmd=search&db=gene&term=THSD7A) | thrombospondin, type I, domain containing 7A | 3e-07 | 0.000143 | 62.41 | 70.15 | 294.74 | (1, 3), (2, 3) |
| [205593_s_at](https://www.affymetrix.com/LinkServlet?probeset=205593_s_at) | [PDE9A](http://www.ncbi.nlm.nih.gov/entrez/query.fcgi?cmd=search&db=gene&term=PDE9A) | phosphodiesterase 9A | 3e-07 | 0.000143 | 90.93 | 19.64 | 99.43 | (2, 1), (2, 3) |
| [1558508_a_at](https://www.affymetrix.com/LinkServlet?probeset=1558508_a_at) | [C1orf53](http://www.ncbi.nlm.nih.gov/entrez/query.fcgi?cmd=search&db=gene&term=C1orf53) | chromosome 1 open reading frame 53 | 3e-07 | 0.000143 | 48.7 | 40.48 | 10.9 | (3, 1), (3, 2) |
| [219501_at](https://www.affymetrix.com/LinkServlet?probeset=219501_at) | [ENOX1](http://www.ncbi.nlm.nih.gov/entrez/query.fcgi?cmd=search&db=gene&term=ENOX1) | ecto-NOX disulfide-thiol exchanger 1 | 3e-07 | 0.000143 | 78.22 | 37.92 | 122.13 | (2, 1), (2, 3) |
| [224215_s_at](https://www.affymetrix.com/LinkServlet?probeset=224215_s_at) | [DLL1](http://www.ncbi.nlm.nih.gov/entrez/query.fcgi?cmd=search&db=gene&term=DLL1) | delta-like 1 (Drosophila) | 3e-07 | 0.000143 | 36.59 | 61.67 | 227.9 | (1, 3), (2, 3) |
| [228646_at](https://www.affymetrix.com/LinkServlet?probeset=228646_at) | [PPP1R1C](http://www.ncbi.nlm.nih.gov/entrez/query.fcgi?cmd=search&db=gene&term=PPP1R1C) | protein phosphatase 1, regulatory (inhibitor) subunit 1C | 4e-07 | 0.000172 | 122.03 | 64.82 | 17.97 | (3, 1), (3, 2) |
| [229034_at](https://www.affymetrix.com/LinkServlet?probeset=229034_at) | [SOBP](http://www.ncbi.nlm.nih.gov/entrez/query.fcgi?cmd=search&db=gene&term=SOBP) | sine oculis binding protein homolog (Drosophila) | 4e-07 | 0.000172 | 167.72 | 113.58 | 295.79 | (1, 3), (2, 3) |
| [1553765_a_at](https://www.affymetrix.com/LinkServlet?probeset=1553765_a_at) | [KLHL32](http://www.ncbi.nlm.nih.gov/entrez/query.fcgi?cmd=search&db=gene&term=KLHL32) | kelch-like 32 (Drosophila) | 4e-07 | 0.000172 | 43.27 | 33.44 | 234.02 | (1, 3), (2, 3) |
| [214920_at](https://www.affymetrix.com/LinkServlet?probeset=214920_at) | [THSD7A](http://www.ncbi.nlm.nih.gov/entrez/query.fcgi?cmd=search&db=gene&term=THSD7A) | thrombospondin, type I, domain containing 7A | 4e-07 | 0.000172 | 90.78 | 101.71 | 404.64 | (1, 3), (2, 3) |
| [203300_x_at](https://www.affymetrix.com/LinkServlet?probeset=203300_x_at) | [AP1S2](http://www.ncbi.nlm.nih.gov/entrez/query.fcgi?cmd=search&db=gene&term=AP1S2) | adaptor-related protein complex 1, sigma 2 subunit | 4e-07 | 0.000172 | 1637.89 | 437.6 | 388.51 | (2, 1), (3, 1) |
| [1569178_at](https://www.affymetrix.com/LinkServlet?probeset=1569178_at) | [GRIA4](http://www.ncbi.nlm.nih.gov/entrez/query.fcgi?cmd=search&db=gene&term=GRIA4) | glutamate receptor, ionotrophic, AMPA 4 | 5e-07 | 0.000203 | 14.88 | 51.35 | 82.3 | (1, 2), (1, 3) |
| [244764_at](https://www.affymetrix.com/LinkServlet?probeset=244764_at) | [NA](http://www.ncbi.nlm.nih.gov/entrez/query.fcgi?cmd=search&db=gene&term=NA) | NA | 5e-07 | 0.000203 | 110.53 | 192.51 | 77.52 | (1, 2), (3, 2) |
| [225782_at](https://www.affymetrix.com/LinkServlet?probeset=225782_at) | [MSRB3](http://www.ncbi.nlm.nih.gov/entrez/query.fcgi?cmd=search&db=gene&term=MSRB3) | methionine sulfoxide reductase B3 | 5e-07 | 0.000203 | 490.39 | 1333.97 | 1269.16 | (1, 2), (1, 3) |
| [221030_s_at](https://www.affymetrix.com/LinkServlet?probeset=221030_s_at) | [ARHGAP24](http://www.ncbi.nlm.nih.gov/entrez/query.fcgi?cmd=search&db=gene&term=ARHGAP24) | Rho GTPase activating protein 24 | 6e-07 | 0.000235 | 32.57 | 45.11 | 15.59 | (3, 1), (3, 2) |
| [222484_s_at](https://www.affymetrix.com/LinkServlet?probeset=222484_s_at) | [CXCL14](http://www.ncbi.nlm.nih.gov/entrez/query.fcgi?cmd=search&db=gene&term=CXCL14) | chemokine (C-X-C motif) ligand 14 | 6e-07 | 0.000235 | 1306.56 | 302.17 | 45.69 | (3, 1), (3, 2) |
| [213894_at](https://www.affymetrix.com/LinkServlet?probeset=213894_at) | [THSD7A](http://www.ncbi.nlm.nih.gov/entrez/query.fcgi?cmd=search&db=gene&term=THSD7A) | thrombospondin, type I, domain containing 7A | 7e-07 | 0.00026 | 60.33 | 51.48 | 264.53 | (1, 3), (2, 3) |
| [244420_at](https://www.affymetrix.com/LinkServlet?probeset=244420_at) | [NA](http://www.ncbi.nlm.nih.gov/entrez/query.fcgi?cmd=search&db=gene&term=NA) | NA | 7e-07 | 0.00026 | 10.87 | 9.76 | 48.92 | (1, 3), (2, 3) |
| [230458_at](https://www.affymetrix.com/LinkServlet?probeset=230458_at) | [SLC45A1](http://www.ncbi.nlm.nih.gov/entrez/query.fcgi?cmd=search&db=gene&term=SLC45A1) | solute carrier family 45, member 1 | 7e-07 | 0.00026 | 28.77 | 31.56 | 58.66 | (1, 3), (2, 3) |
| [1558388_a_at](https://www.affymetrix.com/LinkServlet?probeset=1558388_a_at) | [LOC643763](http://www.ncbi.nlm.nih.gov/entrez/query.fcgi?cmd=search&db=gene&term=LOC643763) | hypothetical LOC643763 | 8e-07 | 0.000283 | 707.8 | 41.01 | 728.99 | (2, 1), (2, 3) |
| [235494_at](https://www.affymetrix.com/LinkServlet?probeset=235494_at) | [NA](http://www.ncbi.nlm.nih.gov/entrez/query.fcgi?cmd=search&db=gene&term=NA) | NA | 8e-07 | 0.000283 | 1241.98 | 1517.4 | 2650.31 | (1, 3), (2, 3) |
| [229638_at](https://www.affymetrix.com/LinkServlet?probeset=229638_at) | [IRX3](http://www.ncbi.nlm.nih.gov/entrez/query.fcgi?cmd=search&db=gene&term=IRX3) | iroquois homeobox 3 | 8e-07 | 0.000283 | 19.67 | 41.79 | 145.48 | (1, 3), (2, 3) |
| [230802_at](https://www.affymetrix.com/LinkServlet?probeset=230802_at) | [ARHGAP24](http://www.ncbi.nlm.nih.gov/entrez/query.fcgi?cmd=search&db=gene&term=ARHGAP24) | Rho GTPase activating protein 24 | 1e-06 | 0.000348 | 28.89 | 43.54 | 13.79 | (3, 1), (3, 2) |
| [244694_at](https://www.affymetrix.com/LinkServlet?probeset=244694_at) | [IGLON5](http://www.ncbi.nlm.nih.gov/entrez/query.fcgi?cmd=search&db=gene&term=IGLON5) | IgLON family member 5 | 1.2e-06 | 0.000392 | 14.27 | 6.38 | 34.76 | (1, 3), (2, 3) |
| [225504_at](https://www.affymetrix.com/LinkServlet?probeset=225504_at) | [NA](http://www.ncbi.nlm.nih.gov/entrez/query.fcgi?cmd=search&db=gene&term=NA) | NA | 1.2e-06 | 0.000392 | 303.56 | 171.03 | 402.17 | (2, 1), (2, 3) |
| [225242_s_at](https://www.affymetrix.com/LinkServlet?probeset=225242_s_at) | [CCDC80](http://www.ncbi.nlm.nih.gov/entrez/query.fcgi?cmd=search&db=gene&term=CCDC80) | coiled-coil domain containing 80 | 1.2e-06 | 0.000392 | 53.05 | 267.45 | 360.91 | (1, 2), (1, 3) |
| [221107_at](https://www.affymetrix.com/LinkServlet?probeset=221107_at) | [CHRNA9](http://www.ncbi.nlm.nih.gov/entrez/query.fcgi?cmd=search&db=gene&term=CHRNA9) | cholinergic receptor, nicotinic, alpha 9 | 1.2e-06 | 0.000392 | 24.66 | 7.33 | 4.89 | (2, 1), (3, 1) |
| [223422_s_at](https://www.affymetrix.com/LinkServlet?probeset=223422_s_at) | [ARHGAP24](http://www.ncbi.nlm.nih.gov/entrez/query.fcgi?cmd=search&db=gene&term=ARHGAP24) | Rho GTPase activating protein 24 | 1.4e-06 | 0.000451 | 195.81 | 212.58 | 63.56 | (3, 1), (3, 2) |
| [222482_at](https://www.affymetrix.com/LinkServlet?probeset=222482_at) | [NA](http://www.ncbi.nlm.nih.gov/entrez/query.fcgi?cmd=search&db=gene&term=NA) | NA | 1.5e-06 | 0.000476 | 231.54 | 79.98 | 275.34 | (2, 1), (2, 3) |
| [239767_at](https://www.affymetrix.com/LinkServlet?probeset=239767_at) | [NA](http://www.ncbi.nlm.nih.gov/entrez/query.fcgi?cmd=search&db=gene&term=NA) | NA | 1.6e-06 | 0.000487 | 33.89 | 7.91 | 5.37 | (2, 1), (3, 1) |
| [229163_at](https://www.affymetrix.com/LinkServlet?probeset=229163_at) | [CAMK2N1](http://www.ncbi.nlm.nih.gov/entrez/query.fcgi?cmd=search&db=gene&term=CAMK2N1) | calcium/calmodulin-dependent protein kinase II inhibitor 1 | 1.6e-06 | 0.000487 | 238.34 | 147.28 | 337.45 | (2, 1), (2, 3) |
| [203299_s_at](https://www.affymetrix.com/LinkServlet?probeset=203299_s_at) | [AP1S2](http://www.ncbi.nlm.nih.gov/entrez/query.fcgi?cmd=search&db=gene&term=AP1S2) | adaptor-related protein complex 1, sigma 2 subunit | 1.6e-06 | 0.000487 | 1205.88 | 553.12 | 400.77 | (2, 1), (3, 1) |
| [218002_s_at](https://www.affymetrix.com/LinkServlet?probeset=218002_s_at) | [CXCL14](http://www.ncbi.nlm.nih.gov/entrez/query.fcgi?cmd=search&db=gene&term=CXCL14) | chemokine (C-X-C motif) ligand 14 | 1.7e-06 | 0.00051 | 1646.22 | 325 | 53.24 | (3, 1), (3, 2) |
| [213601_at](https://www.affymetrix.com/LinkServlet?probeset=213601_at) | [SLIT1](http://www.ncbi.nlm.nih.gov/entrez/query.fcgi?cmd=search&db=gene&term=SLIT1) | slit homolog 1 (Drosophila) | 1.9e-06 | 0.000555 | 266.63 | 770.15 | 105.38 | (3, 2) |
| [205528_s_at](https://www.affymetrix.com/LinkServlet?probeset=205528_s_at) | [RUNX1T1](http://www.ncbi.nlm.nih.gov/entrez/query.fcgi?cmd=search&db=gene&term=RUNX1T1) | runt-related transcription factor 1; translocated to, 1 (cyclin D-related) | 1.9e-06 | 0.000555 | 84.15 | 85.67 | 228.34 | (1, 3), (2, 3) |
| [228307_at](https://www.affymetrix.com/LinkServlet?probeset=228307_at) | [EMILIN3](http://www.ncbi.nlm.nih.gov/entrez/query.fcgi?cmd=search&db=gene&term=EMILIN3) | elastin microfibril interfacer 3 | 2.1e-06 | 0.000603 | 28.91 | 56.31 | 168.05 | (1, 3), (2, 3) |
| [1552386_at](https://www.affymetrix.com/LinkServlet?probeset=1552386_at) | [GAPT](http://www.ncbi.nlm.nih.gov/entrez/query.fcgi?cmd=search&db=gene&term=GAPT) | GRB2-binding adaptor protein, transmembrane | 2.2e-06 | 0.000603 | 65.19 | 87.26 | 24.36 | (3, 1), (3, 2) |
| [224331_s_at](https://www.affymetrix.com/LinkServlet?probeset=224331_s_at) | [MRPL36](http://www.ncbi.nlm.nih.gov/entrez/query.fcgi?cmd=search&db=gene&term=MRPL36) | mitochondrial ribosomal protein L36 | 2.2e-06 | 0.000603 | 2230.02 | 2363.95 | 1467.88 | (3, 1), (3, 2) |
| [231223_at](https://www.affymetrix.com/LinkServlet?probeset=231223_at) | [CSMD1](http://www.ncbi.nlm.nih.gov/entrez/query.fcgi?cmd=search&db=gene&term=CSMD1) | CUB and Sushi multiple domains 1 | 2.2e-06 | 0.000603 | 94.19 | 133.14 | 409.94 | (1, 3), (2, 3) |
| [202834_at](https://www.affymetrix.com/LinkServlet?probeset=202834_at) | [AGT](http://www.ncbi.nlm.nih.gov/entrez/query.fcgi?cmd=search&db=gene&term=AGT) | angiotensinogen (serpin peptidase inhibitor, clade A, member 8) | 2.2e-06 | 0.000603 | 990.11 | 991.22 | 3882.66 | (1, 3), (2, 3) |
| [227297_at](https://www.affymetrix.com/LinkServlet?probeset=227297_at) | [ITGA9](http://www.ncbi.nlm.nih.gov/entrez/query.fcgi?cmd=search&db=gene&term=ITGA9) | integrin, alpha 9 | 2.3e-06 | 0.000622 | 48.87 | 134.98 | 194.98 | (1, 2), (1, 3) |
| [243061_at](https://www.affymetrix.com/LinkServlet?probeset=243061_at) | [C14orf23](http://www.ncbi.nlm.nih.gov/entrez/query.fcgi?cmd=search&db=gene&term=C14orf23) | chromosome 14 open reading frame 23 | 2.4e-06 | 0.000641 | 15.5 | 5.29 | 4.75 | (2, 1), (3, 1) |
| [205932_s_at](https://www.affymetrix.com/LinkServlet?probeset=205932_s_at) | [MSX1](http://www.ncbi.nlm.nih.gov/entrez/query.fcgi?cmd=search&db=gene&term=MSX1) | msh homeobox 1 | 2.5e-06 | 0.000652 | 113.26 | 257.14 | 60.55 | (3, 2) |
| [226743_at](https://www.affymetrix.com/LinkServlet?probeset=226743_at) | [SLFN11](http://www.ncbi.nlm.nih.gov/entrez/query.fcgi?cmd=search&db=gene&term=SLFN11) | schlafen family member 11 | 2.5e-06 | 0.000652 | 42 | 177.49 | 36.09 | (1, 2), (3, 2) |
| [219885_at](https://www.affymetrix.com/LinkServlet?probeset=219885_at) | [SLFN12](http://www.ncbi.nlm.nih.gov/entrez/query.fcgi?cmd=search&db=gene&term=SLFN12) | schlafen family member 12 | 2.6e-06 | 0.000662 | 18.33 | 45.81 | 16.15 | (1, 2), (3, 2) |
| [243879_at](https://www.affymetrix.com/LinkServlet?probeset=243879_at) | [NA](http://www.ncbi.nlm.nih.gov/entrez/query.fcgi?cmd=search&db=gene&term=NA) | NA | 2.6e-06 | 0.000662 | 47.87 | 83.79 | 225.57 | (1, 3), (2, 3) |
| [225250_at](https://www.affymetrix.com/LinkServlet?probeset=225250_at) | [STIM2](http://www.ncbi.nlm.nih.gov/entrez/query.fcgi?cmd=search&db=gene&term=STIM2) | stromal interaction molecule 2 | 3e-06 | 0.000739 | 57.6 | 29.78 | 52.33 | (2, 1), (2, 3) |
| [219197_s_at](https://www.affymetrix.com/LinkServlet?probeset=219197_s_at) | [SCUBE2](http://www.ncbi.nlm.nih.gov/entrez/query.fcgi?cmd=search&db=gene&term=SCUBE2) | signal peptide, CUB domain, EGF-like 2 | 3e-06 | 0.000739 | 45.04 | 43.06 | 180.8 | (1, 3), (2, 3) |
| [207231_at](https://www.affymetrix.com/LinkServlet?probeset=207231_at) | [DZIP3](http://www.ncbi.nlm.nih.gov/entrez/query.fcgi?cmd=search&db=gene&term=DZIP3) | DAZ interacting protein 3, zinc finger | 3e-06 | 0.000739 | 116.15 | 84 | 158.2 | (2, 3) |
| [226487_at](https://www.affymetrix.com/LinkServlet?probeset=226487_at) | [C12orf34](http://www.ncbi.nlm.nih.gov/entrez/query.fcgi?cmd=search&db=gene&term=C12orf34) | chromosome 12 open reading frame 34 | 3.1e-06 | 0.000755 | 61.54 | 56.79 | 141 | (1, 3), (2, 3) |
| [214841_at](https://www.affymetrix.com/LinkServlet?probeset=214841_at) | [CNIH3](http://www.ncbi.nlm.nih.gov/entrez/query.fcgi?cmd=search&db=gene&term=CNIH3) | cornichon homolog 3 (Drosophila) | 3.3e-06 | 0.000795 | 297.58 | 295.89 | 85.46 | (3, 1), (3, 2) |
| [220595_at](https://www.affymetrix.com/LinkServlet?probeset=220595_at) | [PDZRN4](http://www.ncbi.nlm.nih.gov/entrez/query.fcgi?cmd=search&db=gene&term=PDZRN4) | PDZ domain containing ring finger 4 | 3.4e-06 | 0.00081 | 15.81 | 24.9 | 115.23 | (1, 3), (2, 3) |
| [221704_s_at](https://www.affymetrix.com/LinkServlet?probeset=221704_s_at) | [VPS37B](http://www.ncbi.nlm.nih.gov/entrez/query.fcgi?cmd=search&db=gene&term=VPS37B) | vacuolar protein sorting 37 homolog B (S. cerevisiae) | 3.7e-06 | 0.000872 | 270.28 | 144.6 | 100.14 | (2, 1), (3, 1) |
| [208017_s_at](https://www.affymetrix.com/LinkServlet?probeset=208017_s_at) | [MCF2](http://www.ncbi.nlm.nih.gov/entrez/query.fcgi?cmd=search&db=gene&term=MCF2) | MCF.2 cell line derived transforming sequence | 3.9e-06 | 0.00089 | 21.94 | 35.53 | 147.94 | (1, 3), (2, 3) |
| [213478_at](https://www.affymetrix.com/LinkServlet?probeset=213478_at) | [RP1-21O18.1](http://www.ncbi.nlm.nih.gov/entrez/query.fcgi?cmd=search&db=gene&term=RP1-21O18.1) | kazrin | 3.9e-06 | 0.00089 | 264.21 | 198.38 | 482.21 | (1, 3), (2, 3) |
| [218901_at](https://www.affymetrix.com/LinkServlet?probeset=218901_at) | [PLSCR4](http://www.ncbi.nlm.nih.gov/entrez/query.fcgi?cmd=search&db=gene&term=PLSCR4) | phospholipid scramblase 4 | 3.9e-06 | 0.00089 | 748.82 | 1456.02 | 1803 | (1, 2), (1, 3) |
| [238846_at](https://www.affymetrix.com/LinkServlet?probeset=238846_at) | [TNFRSF11A](http://www.ncbi.nlm.nih.gov/entrez/query.fcgi?cmd=search&db=gene&term=TNFRSF11A) | tumor necrosis factor receptor superfamily, member 11a, NFKB activator | 4.5e-06 | 0.00102 | 68.41 | 99.49 | 32.03 | (3, 1), (3, 2) |
| [204304_s_at](https://www.affymetrix.com/LinkServlet?probeset=204304_s_at) | [PROM1](http://www.ncbi.nlm.nih.gov/entrez/query.fcgi?cmd=search&db=gene&term=PROM1) | prominin 1 | 4.6e-06 | 0.00103 | 129.99 | 40.1 | 350.45 | (2, 3) |
| [225706_at](https://www.affymetrix.com/LinkServlet?probeset=225706_at) | [GLCCI1](http://www.ncbi.nlm.nih.gov/entrez/query.fcgi?cmd=search&db=gene&term=GLCCI1) | glucocorticoid induced transcript 1 | 4.8e-06 | 0.00106 | 318.4 | 307.55 | 638.43 | (1, 3), (2, 3) |
| [238663_x_at](https://www.affymetrix.com/LinkServlet?probeset=238663_x_at) | [GRIA4](http://www.ncbi.nlm.nih.gov/entrez/query.fcgi?cmd=search&db=gene&term=GRIA4) | glutamate receptor, ionotrophic, AMPA 4 | 5e-06 | 0.0011 | 45.3 | 225.8 | 417.66 | (1, 2), (1, 3) |
| [234996_at](https://www.affymetrix.com/LinkServlet?probeset=234996_at) | [CALCRL](http://www.ncbi.nlm.nih.gov/entrez/query.fcgi?cmd=search&db=gene&term=CALCRL) | calcitonin receptor-like | 5.1e-06 | 0.00111 | 75.18 | 105.04 | 281.9 | (1, 3), (2, 3) |
| [204932_at](https://www.affymetrix.com/LinkServlet?probeset=204932_at) | [TNFRSF11B](http://www.ncbi.nlm.nih.gov/entrez/query.fcgi?cmd=search&db=gene&term=TNFRSF11B) | tumor necrosis factor receptor superfamily, member 11b | 5.3e-06 | 0.00114 | 26.65 | 92.15 | 147.14 | (1, 2), (1, 3) |
| [213108_at](https://www.affymetrix.com/LinkServlet?probeset=213108_at) | [CAMK2A](http://www.ncbi.nlm.nih.gov/entrez/query.fcgi?cmd=search&db=gene&term=CAMK2A) | calcium/calmodulin-dependent protein kinase II alpha | 5.6e-06 | 0.00118 | 149.14 | 27.6 | 29.15 | (2, 1), (3, 1) |
| [239782_at](https://www.affymetrix.com/LinkServlet?probeset=239782_at) | [RBP1](http://www.ncbi.nlm.nih.gov/entrez/query.fcgi?cmd=search&db=gene&term=RBP1) | retinol binding protein 1, cellular | 5.6e-06 | 0.00118 | 8.4 | 10.06 | 16.36 | (1, 3), (2, 3) |
| [1558387_at](https://www.affymetrix.com/LinkServlet?probeset=1558387_at) | [LOC643763](http://www.ncbi.nlm.nih.gov/entrez/query.fcgi?cmd=search&db=gene&term=LOC643763) | hypothetical LOC643763 | 6.4e-06 | 0.00133 | 110 | 12.13 | 123.79 | (2, 1), (2, 3) |
| [213353_at](https://www.affymetrix.com/LinkServlet?probeset=213353_at) | [ABCA5](http://www.ncbi.nlm.nih.gov/entrez/query.fcgi?cmd=search&db=gene&term=ABCA5) | ATP-binding cassette, sub-family A (ABC1), member 5 | 6.5e-06 | 0.00133 | 25.78 | 112.59 | 123.41 | (1, 2), (1, 3) |
| [210347_s_at](https://www.affymetrix.com/LinkServlet?probeset=210347_s_at) | [BCL11A](http://www.ncbi.nlm.nih.gov/entrez/query.fcgi?cmd=search&db=gene&term=BCL11A) | B-cell CLL/lymphoma 11A (zinc finger protein) | 6.5e-06 | 0.00133 | 33.11 | 20.74 | 6.5 | (3, 1), (3, 2) |
| [1553972_a_at](https://www.affymetrix.com/LinkServlet?probeset=1553972_a_at) | [CBS](http://www.ncbi.nlm.nih.gov/entrez/query.fcgi?cmd=search&db=gene&term=CBS) | cystathionine-beta-synthase | 6.9e-06 | 0.0014 | 49.46 | 35.86 | 82.76 | (1, 3), (2, 3) |
| [226197_at](https://www.affymetrix.com/LinkServlet?probeset=226197_at) | [NA](http://www.ncbi.nlm.nih.gov/entrez/query.fcgi?cmd=search&db=gene&term=NA) | NA | 7.3e-06 | 0.00147 | 44.45 | 208.45 | 127.47 | (1, 2), (1, 3) |
| [229065_at](https://www.affymetrix.com/LinkServlet?probeset=229065_at) | [SLC35F3](http://www.ncbi.nlm.nih.gov/entrez/query.fcgi?cmd=search&db=gene&term=SLC35F3) | solute carrier family 35, member F3 | 7.4e-06 | 0.00147 | 15.71 | 46.11 | 91.66 | (1, 2), (1, 3) |
| [1552798_a_at](https://www.affymetrix.com/LinkServlet?probeset=1552798_a_at) | [TLR4](http://www.ncbi.nlm.nih.gov/entrez/query.fcgi?cmd=search&db=gene&term=TLR4) | toll-like receptor 4 | 7.9e-06 | 0.00156 | 9.81 | 15.46 | 7.5 | (1, 2), (3, 2) |
| [201188_s_at](https://www.affymetrix.com/LinkServlet?probeset=201188_s_at) | [ITPR3](http://www.ncbi.nlm.nih.gov/entrez/query.fcgi?cmd=search&db=gene&term=ITPR3) | inositol 1,4,5-triphosphate receptor, type 3 | 8e-06 | 0.00156 | 36.85 | 15.27 | 11.65 | (2, 1), (3, 1) |
| [226374_at](https://www.affymetrix.com/LinkServlet?probeset=226374_at) | [NA](http://www.ncbi.nlm.nih.gov/entrez/query.fcgi?cmd=search&db=gene&term=NA) | NA | 8.4e-06 | 0.00163 | 2320.39 | 1094.42 | 780.1 | (2, 1), (3, 1) |
| [236536_at](https://www.affymetrix.com/LinkServlet?probeset=236536_at) | [GALNT13](http://www.ncbi.nlm.nih.gov/entrez/query.fcgi?cmd=search&db=gene&term=GALNT13) | UDP-N-acetyl-alpha-D-galactosamine:polypeptide N-acetylgalactosaminyltransferase 13 (GalNAc-T13) | 8.8e-06 | 0.00169 | 31.05 | 82.11 | 176.73 | (1, 3) |
| [1552634_a_at](https://www.affymetrix.com/LinkServlet?probeset=1552634_a_at) | [ZNF101](http://www.ncbi.nlm.nih.gov/entrez/query.fcgi?cmd=search&db=gene&term=ZNF101) | zinc finger protein 101 | 9.3e-06 | 0.00177 | 10.07 | 14.27 | 8.81 | (1, 2), (3, 2) |
| [230179_at](https://www.affymetrix.com/LinkServlet?probeset=230179_at) | [LOC285812](http://www.ncbi.nlm.nih.gov/entrez/query.fcgi?cmd=search&db=gene&term=LOC285812) | hypothetical protein LOC285812 | 9.7e-06 | 0.00182 | 280.56 | 110.13 | 268.49 | (2, 1), (2, 3) |
| [1557745_at](https://www.affymetrix.com/LinkServlet?probeset=1557745_at) | [NA](http://www.ncbi.nlm.nih.gov/entrez/query.fcgi?cmd=search&db=gene&term=NA) | NA | 9.7e-06 | 0.00182 | 27.49 | 14.03 | 117.81 | (1, 3), (2, 3) |
| [235831_at](https://www.affymetrix.com/LinkServlet?probeset=235831_at) | [NA](http://www.ncbi.nlm.nih.gov/entrez/query.fcgi?cmd=search&db=gene&term=NA) | NA | 1.01e-05 | 0.00188 | 18.76 | 43.18 | 104.47 | (1, 3), (2, 3) |
| [209035_at](https://www.affymetrix.com/LinkServlet?probeset=209035_at) | [MDK](http://www.ncbi.nlm.nih.gov/entrez/query.fcgi?cmd=search&db=gene&term=MDK) | midkine (neurite growth-promoting factor 2) | 1.12e-05 | 0.00205 | 162.72 | 101.66 | 46.6 | (3, 1), (3, 2) |
| [230482_at](https://www.affymetrix.com/LinkServlet?probeset=230482_at) | [ST6GALNAC5](http://www.ncbi.nlm.nih.gov/entrez/query.fcgi?cmd=search&db=gene&term=ST6GALNAC5) | ST6 (alpha-N-acetyl-neuraminyl-2,3-beta-galactosyl-1,3)-N-acetylgalactosaminide alpha-2,6-sialyltransferase 5 | 1.13e-05 | 0.00205 | 9.14 | 4.83 | 4.81 | (2, 1), (3, 1) |
| [227481_at](https://www.affymetrix.com/LinkServlet?probeset=227481_at) | [CNKSR3](http://www.ncbi.nlm.nih.gov/entrez/query.fcgi?cmd=search&db=gene&term=CNKSR3) | CNKSR family member 3 | 1.13e-05 | 0.00205 | 207.53 | 208.17 | 394.33 | (1, 3), (2, 3) |
| [233499_at](https://www.affymetrix.com/LinkServlet?probeset=233499_at) | [LRRC7](http://www.ncbi.nlm.nih.gov/entrez/query.fcgi?cmd=search&db=gene&term=LRRC7) | leucine rich repeat containing 7 | 1.15e-05 | 0.00207 | 20.82 | 6.67 | 4.75 | (2, 1), (3, 1) |
| [229606_at](https://www.affymetrix.com/LinkServlet?probeset=229606_at) | [NA](http://www.ncbi.nlm.nih.gov/entrez/query.fcgi?cmd=search&db=gene&term=NA) | NA | 1.16e-05 | 0.00207 | 910.22 | 495.29 | 942.26 | (2, 1), (2, 3) |
| [229159_at](https://www.affymetrix.com/LinkServlet?probeset=229159_at) | [THSD7A](http://www.ncbi.nlm.nih.gov/entrez/query.fcgi?cmd=search&db=gene&term=THSD7A) | thrombospondin, type I, domain containing 7A | 1.17e-05 | 0.00207 | 17.02 | 22.1 | 71.42 | (1, 3), (2, 3) |
| [230895_at](https://www.affymetrix.com/LinkServlet?probeset=230895_at) | [HAPLN1](http://www.ncbi.nlm.nih.gov/entrez/query.fcgi?cmd=search&db=gene&term=HAPLN1) | hyaluronan and proteoglycan link protein 1 | 1.23e-05 | 0.00216 | 20.82 | 6.86 | 5.86 | (2, 1), (3, 1) |
| [220979_s_at](https://www.affymetrix.com/LinkServlet?probeset=220979_s_at) | [ST6GALNAC5](http://www.ncbi.nlm.nih.gov/entrez/query.fcgi?cmd=search&db=gene&term=ST6GALNAC5) | ST6 (alpha-N-acetyl-neuraminyl-2,3-beta-galactosyl-1,3)-N-acetylgalactosaminide alpha-2,6-sialyltransferase 5 | 1.24e-05 | 0.00216 | 12.29 | 4.76 | 4.83 | (2, 1), (3, 1) |
| [228104_at](https://www.affymetrix.com/LinkServlet?probeset=228104_at) | [PLXNA4](http://www.ncbi.nlm.nih.gov/entrez/query.fcgi?cmd=search&db=gene&term=PLXNA4) | plexin A4 | 1.26e-05 | 0.00217 | 37.22 | 11.34 | 25.91 | (2, 1), (2, 3) |
| [243139_at](https://www.affymetrix.com/LinkServlet?probeset=243139_at) | [NA](http://www.ncbi.nlm.nih.gov/entrez/query.fcgi?cmd=search&db=gene&term=NA) | NA | 1.27e-05 | 0.00217 | 9.72 | 12.84 | 40.36 | (1, 3), (2, 3) |
| [213188_s_at](https://www.affymetrix.com/LinkServlet?probeset=213188_s_at) | [MINA](http://www.ncbi.nlm.nih.gov/entrez/query.fcgi?cmd=search&db=gene&term=MINA) | MYC induced nuclear antigen | 1.28e-05 | 0.00217 | 613.86 | 1305.76 | 918.22 | (1, 2), (1, 3), (3, 2) |
| [226589_at](https://www.affymetrix.com/LinkServlet?probeset=226589_at) | [TMEM192](http://www.ncbi.nlm.nih.gov/entrez/query.fcgi?cmd=search&db=gene&term=TMEM192) | transmembrane protein 192 | 1.31e-05 | 0.00221 | 103.74 | 67.38 | 124.93 | (2, 1), (2, 3) |
| [218796_at](https://www.affymetrix.com/LinkServlet?probeset=218796_at) | [FERMT1](http://www.ncbi.nlm.nih.gov/entrez/query.fcgi?cmd=search&db=gene&term=FERMT1) | fermitin family homolog 1 (Drosophila) | 1.37e-05 | 0.00227 | 37.49 | 61.46 | 241.78 | (1, 3), (2, 3) |
| [213752_at](https://www.affymetrix.com/LinkServlet?probeset=213752_at) | [RP1-21O18.1](http://www.ncbi.nlm.nih.gov/entrez/query.fcgi?cmd=search&db=gene&term=RP1-21O18.1) | kazrin | 1.37e-05 | 0.00227 | 80.87 | 100.02 | 209.82 | (1, 3), (2, 3) |
| [214053_at](https://www.affymetrix.com/LinkServlet?probeset=214053_at) | [ERBB4](http://www.ncbi.nlm.nih.gov/entrez/query.fcgi?cmd=search&db=gene&term=ERBB4) | v-erb-a erythroblastic leukemia viral oncogene homolog 4 (avian) | 1.38e-05 | 0.00227 | 117.82 | 466.16 | 654.31 | (1, 2), (1, 3) |
| [209815_at](https://www.affymetrix.com/LinkServlet?probeset=209815_at) | [PTCH1](http://www.ncbi.nlm.nih.gov/entrez/query.fcgi?cmd=search&db=gene&term=PTCH1) | patched homolog 1 (Drosophila) | 1.41e-05 | 0.0023 | 426.31 | 628.32 | 1153.95 | (1, 3), (2, 3) |
| [201189_s_at](https://www.affymetrix.com/LinkServlet?probeset=201189_s_at) | [ITPR3](http://www.ncbi.nlm.nih.gov/entrez/query.fcgi?cmd=search&db=gene&term=ITPR3) | inositol 1,4,5-triphosphate receptor, type 3 | 1.42e-05 | 0.0023 | 278.03 | 72.15 | 49.33 | (2, 1), (3, 1) |
| [224970_at](https://www.affymetrix.com/LinkServlet?probeset=224970_at) | [NFIA](http://www.ncbi.nlm.nih.gov/entrez/query.fcgi?cmd=search&db=gene&term=NFIA) | nuclear factor I/A | 1.44e-05 | 0.00232 | 1566.97 | 1340.57 | 2560.05 | (1, 3), (2, 3) |
| [205498_at](https://www.affymetrix.com/LinkServlet?probeset=205498_at) | [GHR](http://www.ncbi.nlm.nih.gov/entrez/query.fcgi?cmd=search&db=gene&term=GHR) | growth hormone receptor | 1.48e-05 | 0.00237 | 46.62 | 175.01 | 155.23 | (1, 2), (1, 3) |
| [238426_at](https://www.affymetrix.com/LinkServlet?probeset=238426_at) | [TMEM130](http://www.ncbi.nlm.nih.gov/entrez/query.fcgi?cmd=search&db=gene&term=TMEM130) | transmembrane protein 130 | 1.54e-05 | 0.00245 | 21.29 | 6.47 | 33.72 | (2, 1), (2, 3) |
| [204214_s_at](https://www.affymetrix.com/LinkServlet?probeset=204214_s_at) | [RAB32](http://www.ncbi.nlm.nih.gov/entrez/query.fcgi?cmd=search&db=gene&term=RAB32) | RAB32, member RAS oncogene family | 1.57e-05 | 0.00247 | 154.86 | 244.07 | 81.2 | (3, 1), (3, 2) |
| [228083_at](https://www.affymetrix.com/LinkServlet?probeset=228083_at) | [CACNA2D4](http://www.ncbi.nlm.nih.gov/entrez/query.fcgi?cmd=search&db=gene&term=CACNA2D4) | calcium channel, voltage-dependent, alpha 2/delta subunit 4 | 1.58e-05 | 0.00247 | 8.82 | 13.95 | 7.73 | (1, 2), (3, 2) |
| [229714_at](https://www.affymetrix.com/LinkServlet?probeset=229714_at) | [HS6ST3](http://www.ncbi.nlm.nih.gov/entrez/query.fcgi?cmd=search&db=gene&term=HS6ST3) | heparan sulfate 6-O-sulfotransferase 3 | 1.59e-05 | 0.00247 | 22.13 | 18.55 | 75.06 | (1, 3), (2, 3) |
| [218309_at](https://www.affymetrix.com/LinkServlet?probeset=218309_at) | [CAMK2N1](http://www.ncbi.nlm.nih.gov/entrez/query.fcgi?cmd=search&db=gene&term=CAMK2N1) | calcium/calmodulin-dependent protein kinase II inhibitor 1 | 1.63e-05 | 0.00251 | 1947.14 | 1180.51 | 2373.32 | (2, 1), (2, 3) |
| [219563_at](https://www.affymetrix.com/LinkServlet?probeset=219563_at) | [C14orf139](http://www.ncbi.nlm.nih.gov/entrez/query.fcgi?cmd=search&db=gene&term=C14orf139) | chromosome 14 open reading frame 139 | 1.64e-05 | 0.00251 | 167.49 | 342.4 | 477.4 | (1, 2), (1, 3) |
| [225790_at](https://www.affymetrix.com/LinkServlet?probeset=225790_at) | [MSRB3](http://www.ncbi.nlm.nih.gov/entrez/query.fcgi?cmd=search&db=gene&term=MSRB3) | methionine sulfoxide reductase B3 | 1.66e-05 | 0.00251 | 34.06 | 93.2 | 102.47 | (1, 2), (1, 3) |
| [1555216_a_at](https://www.affymetrix.com/LinkServlet?probeset=1555216_a_at) | [LOC645722](http://www.ncbi.nlm.nih.gov/entrez/query.fcgi?cmd=search&db=gene&term=LOC645722) | hypothetical LOC645722 | 1.66e-05 | 0.00251 | 12.77 | 6.38 | 5.06 | (2, 1), (3, 1) |
| [230496_at](https://www.affymetrix.com/LinkServlet?probeset=230496_at) | [FAM123A](http://www.ncbi.nlm.nih.gov/entrez/query.fcgi?cmd=search&db=gene&term=FAM123A) | family with sequence similarity 123A | 1.68e-05 | 0.00252 | 716.63 | 335.97 | 1820.6 | (2, 3) |
| [219528_s_at](https://www.affymetrix.com/LinkServlet?probeset=219528_s_at) | [BCL11B](http://www.ncbi.nlm.nih.gov/entrez/query.fcgi?cmd=search&db=gene&term=BCL11B) | B-cell CLL/lymphoma 11B (zinc finger protein) | 1.7e-05 | 0.00253 | 11.73 | 14.52 | 36.89 | (1, 3), (2, 3) |
| [205110_s_at](https://www.affymetrix.com/LinkServlet?probeset=205110_s_at) | [FGF13](http://www.ncbi.nlm.nih.gov/entrez/query.fcgi?cmd=search&db=gene&term=FGF13) | fibroblast growth factor 13 | 1.72e-05 | 0.00254 | 85.07 | 23.87 | 206.23 | (2, 3) |
| [232275_s_at](https://www.affymetrix.com/LinkServlet?probeset=232275_s_at) | [HS6ST3](http://www.ncbi.nlm.nih.gov/entrez/query.fcgi?cmd=search&db=gene&term=HS6ST3) | heparan sulfate 6-O-sulfotransferase 3 | 1.73e-05 | 0.00254 | 11.02 | 8.84 | 39.85 | (1, 3), (2, 3) |
| [203126_at](https://www.affymetrix.com/LinkServlet?probeset=203126_at) | [IMPA2](http://www.ncbi.nlm.nih.gov/entrez/query.fcgi?cmd=search&db=gene&term=IMPA2) | inositol(myo)-1(or 4)-monophosphatase 2 | 1.79e-05 | 0.00261 | 25.8 | 44.94 | 16.23 | (3, 2) |
| [213015_at](https://www.affymetrix.com/LinkServlet?probeset=213015_at) | [BBX](http://www.ncbi.nlm.nih.gov/entrez/query.fcgi?cmd=search&db=gene&term=BBX) | bobby sox homolog (Drosophila) | 1.87e-05 | 0.00271 | 429.05 | 281.32 | 517.04 | (2, 1), (2, 3) |
| [212641_at](https://www.affymetrix.com/LinkServlet?probeset=212641_at) | [HIVEP2](http://www.ncbi.nlm.nih.gov/entrez/query.fcgi?cmd=search&db=gene&term=HIVEP2) | human immunodeficiency virus type I enhancer binding protein 2 | 1.91e-05 | 0.00275 | 85.81 | 40.86 | 96.93 | (2, 1), (2, 3) |
| [1555867_at](https://www.affymetrix.com/LinkServlet?probeset=1555867_at) | [GNG4](http://www.ncbi.nlm.nih.gov/entrez/query.fcgi?cmd=search&db=gene&term=GNG4) | guanine nucleotide binding protein (G protein), gamma 4 | 1.94e-05 | 0.00277 | 49.74 | 61.83 | 250.53 | (1, 3), (2, 3) |
| [205330_at](https://www.affymetrix.com/LinkServlet?probeset=205330_at) | [MN1](http://www.ncbi.nlm.nih.gov/entrez/query.fcgi?cmd=search&db=gene&term=MN1) | meningioma (disrupted in balanced translocation) 1 | 1.95e-05 | 0.00277 | 172.64 | 89.32 | 405.26 | (1, 3), (2, 3) |
| [219195_at](https://www.affymetrix.com/LinkServlet?probeset=219195_at) | [PPARGC1A](http://www.ncbi.nlm.nih.gov/entrez/query.fcgi?cmd=search&db=gene&term=PPARGC1A) | peroxisome proliferator-activated receptor gamma, coactivator 1 alpha | 1.96e-05 | 0.00277 | 169.45 | 48.95 | 156.65 | (2, 1), (2, 3) |
| [228728_at](https://www.affymetrix.com/LinkServlet?probeset=228728_at) | [C7orf58](http://www.ncbi.nlm.nih.gov/entrez/query.fcgi?cmd=search&db=gene&term=C7orf58) | chromosome 7 open reading frame 58 | 2.02e-05 | 0.00281 | 357.99 | 522.8 | 178.44 | (3, 1), (3, 2) |
| [232037_at](https://www.affymetrix.com/LinkServlet?probeset=232037_at) | [IGDCC3](http://www.ncbi.nlm.nih.gov/entrez/query.fcgi?cmd=search&db=gene&term=IGDCC3) | immunoglobulin superfamily, DCC subclass, member 3 | 2.02e-05 | 0.00281 | 76.56 | 16.58 | 32.5 | (2, 1), (3, 1), (2, 3) |
| [205240_at](https://www.affymetrix.com/LinkServlet?probeset=205240_at) | [GPSM2](http://www.ncbi.nlm.nih.gov/entrez/query.fcgi?cmd=search&db=gene&term=GPSM2) | G-protein signaling modulator 2 (AGS3-like, C. elegans) | 2.04e-05 | 0.00281 | 78.03 | 120.6 | 196.22 | (1, 3), (2, 3) |
| [240067_at](https://www.affymetrix.com/LinkServlet?probeset=240067_at) | [NA](http://www.ncbi.nlm.nih.gov/entrez/query.fcgi?cmd=search&db=gene&term=NA) | NA | 2.05e-05 | 0.00281 | 29.3 | 24.35 | 146.23 | (1, 3), (2, 3) |
| [226548_at](https://www.affymetrix.com/LinkServlet?probeset=226548_at) | [SBK1](http://www.ncbi.nlm.nih.gov/entrez/query.fcgi?cmd=search&db=gene&term=SBK1) | SH3-binding domain kinase 1 | 2.05e-05 | 0.00281 | 62.91 | 29.34 | 108.53 | (2, 3) |
| [212207_at](https://www.affymetrix.com/LinkServlet?probeset=212207_at) | [MED13L](http://www.ncbi.nlm.nih.gov/entrez/query.fcgi?cmd=search&db=gene&term=MED13L) | mediator complex subunit 13-like | 2.08e-05 | 0.00283 | 206.57 | 160.28 | 285.48 | (2, 3) |
| [215425_at](https://www.affymetrix.com/LinkServlet?probeset=215425_at) | [BTG3](http://www.ncbi.nlm.nih.gov/entrez/query.fcgi?cmd=search&db=gene&term=BTG3) | BTG family, member 3 | 2.13e-05 | 0.00288 | 48.89 | 32.28 | 20.55 | (3, 1), (3, 2) |
| [207658_s_at](https://www.affymetrix.com/LinkServlet?probeset=207658_s_at) | [FOXG1](http://www.ncbi.nlm.nih.gov/entrez/query.fcgi?cmd=search&db=gene&term=FOXG1) | forkhead box G1 | 2.19e-05 | 0.00294 | 13.29 | 5.84 | 5.13 | (2, 1), (3, 1) |
| [206068_s_at](https://www.affymetrix.com/LinkServlet?probeset=206068_s_at) | [ACADL](http://www.ncbi.nlm.nih.gov/entrez/query.fcgi?cmd=search&db=gene&term=ACADL) | acyl-Coenzyme A dehydrogenase, long chain | 2.22e-05 | 0.00296 | 14.48 | 51.26 | 31.55 | (1, 2), (1, 3) |
| [223691_at](https://www.affymetrix.com/LinkServlet?probeset=223691_at) | [RGS22](http://www.ncbi.nlm.nih.gov/entrez/query.fcgi?cmd=search&db=gene&term=RGS22) | regulator of G-protein signaling 22 | 2.24e-05 | 0.00296 | 14.39 | 35.94 | 20.13 | (1, 2), (3, 2) |
| [221044_s_at](https://www.affymetrix.com/LinkServlet?probeset=221044_s_at) | [NA](http://www.ncbi.nlm.nih.gov/entrez/query.fcgi?cmd=search&db=gene&term=NA) | NA | 2.24e-05 | 0.00296 | 71.54 | 106.84 | 40.8 | (3, 1), (3, 2) |
| [223395_at](https://www.affymetrix.com/LinkServlet?probeset=223395_at) | [ABI3BP](http://www.ncbi.nlm.nih.gov/entrez/query.fcgi?cmd=search&db=gene&term=ABI3BP) | ABI family, member 3 (NESH) binding protein | 2.26e-05 | 0.00297 | 49.12 | 1108.34 | 289.62 | (1, 2), (1, 3), (3, 2) |
| [230561_s_at](https://www.affymetrix.com/LinkServlet?probeset=230561_s_at) | [NA](http://www.ncbi.nlm.nih.gov/entrez/query.fcgi?cmd=search&db=gene&term=NA) | NA | 2.28e-05 | 0.00297 | 145.6 | 326.47 | 367.65 | (1, 2), (1, 3) |
| [221922_at](https://www.affymetrix.com/LinkServlet?probeset=221922_at) | [GPSM2](http://www.ncbi.nlm.nih.gov/entrez/query.fcgi?cmd=search&db=gene&term=GPSM2) | G-protein signaling modulator 2 (AGS3-like, C. elegans) | 2.33e-05 | 0.00302 | 611.27 | 856.76 | 1371.89 | (1, 3), (2, 3) |
| [226158_at](https://www.affymetrix.com/LinkServlet?probeset=226158_at) | [KLHL24](http://www.ncbi.nlm.nih.gov/entrez/query.fcgi?cmd=search&db=gene&term=KLHL24) | kelch-like 24 (Drosophila) | 2.35e-05 | 0.00303 | 866.95 | 606 | 1029 | (2, 1), (2, 3) |
| [228737_at](https://www.affymetrix.com/LinkServlet?probeset=228737_at) | [TOX2](http://www.ncbi.nlm.nih.gov/entrez/query.fcgi?cmd=search&db=gene&term=TOX2) | TOX high mobility group box family member 2 | 2.37e-05 | 0.00304 | 20.03 | 65.7 | 77.21 | (1, 2), (1, 3) |
| [202425_x_at](https://www.affymetrix.com/LinkServlet?probeset=202425_x_at) | [PPP3CA](http://www.ncbi.nlm.nih.gov/entrez/query.fcgi?cmd=search&db=gene&term=PPP3CA) | protein phosphatase 3 (formerly 2B), catalytic subunit, alpha isoform | 2.39e-05 | 0.00304 | 1039.81 | 679.05 | 1319.28 | (2, 3) |
| [1553720_a_at](https://www.affymetrix.com/LinkServlet?probeset=1553720_a_at) | [FAM123A](http://www.ncbi.nlm.nih.gov/entrez/query.fcgi?cmd=search&db=gene&term=FAM123A) | family with sequence similarity 123A | 2.48e-05 | 0.00314 | 200.71 | 101.17 | 375.54 | (2, 3) |
| [205738_s_at](https://www.affymetrix.com/LinkServlet?probeset=205738_s_at) | [FABP3](http://www.ncbi.nlm.nih.gov/entrez/query.fcgi?cmd=search&db=gene&term=FABP3) | fatty acid binding protein 3, muscle and heart (mammary-derived growth inhibitor) | 2.52e-05 | 0.00317 | 51.5 | 113.99 | 28.5 | (3, 2) |
| [212445_s_at](https://www.affymetrix.com/LinkServlet?probeset=212445_s_at) | [NEDD4L](http://www.ncbi.nlm.nih.gov/entrez/query.fcgi?cmd=search&db=gene&term=NEDD4L) | neural precursor cell expressed, developmentally down-regulated 4-like | 2.56e-05 | 0.00321 | 23.88 | 7.94 | 6.42 | (2, 1), (3, 1) |
| [60474_at](https://www.affymetrix.com/LinkServlet?probeset=60474_at) | [FERMT1](http://www.ncbi.nlm.nih.gov/entrez/query.fcgi?cmd=search&db=gene&term=FERMT1) | fermitin family homolog 1 (Drosophila) | 2.64e-05 | 0.00327 | 48.4 | 71.21 | 281.77 | (1, 3), (2, 3) |
| [207705_s_at](https://www.affymetrix.com/LinkServlet?probeset=207705_s_at) | [NINL](http://www.ncbi.nlm.nih.gov/entrez/query.fcgi?cmd=search&db=gene&term=NINL) | ninein-like | 2.66e-05 | 0.00327 | 102.81 | 96.35 | 167.77 | (1, 3), (2, 3) |
| [212239_at](https://www.affymetrix.com/LinkServlet?probeset=212239_at) | [PIK3R1](http://www.ncbi.nlm.nih.gov/entrez/query.fcgi?cmd=search&db=gene&term=PIK3R1) | phosphoinositide-3-kinase, regulatory subunit 1 (alpha) | 2.66e-05 | 0.00327 | 2534.33 | 2949.37 | 4694.65 | (1, 3), (2, 3) |
| [57588_at](https://www.affymetrix.com/LinkServlet?probeset=57588_at) | [SLC24A3](http://www.ncbi.nlm.nih.gov/entrez/query.fcgi?cmd=search&db=gene&term=SLC24A3) | solute carrier family 24 (sodium/potassium/calcium exchanger), member 3 | 2.74e-05 | 0.00335 | 99.26 | 133.6 | 369.33 | (1, 3), (2, 3) |
| [216086_at](https://www.affymetrix.com/LinkServlet?probeset=216086_at) | [SV2C](http://www.ncbi.nlm.nih.gov/entrez/query.fcgi?cmd=search&db=gene&term=SV2C) | synaptic vesicle glycoprotein 2C | 2.8e-05 | 0.00341 | 9.75 | 13.07 | 48.72 | (1, 3), (2, 3) |
| [222895_s_at](https://www.affymetrix.com/LinkServlet?probeset=222895_s_at) | [BCL11B](http://www.ncbi.nlm.nih.gov/entrez/query.fcgi?cmd=search&db=gene&term=BCL11B) | B-cell CLL/lymphoma 11B (zinc finger protein) | 2.84e-05 | 0.00344 | 11.28 | 11.59 | 40.03 | (1, 3), (2, 3) |
| [235916_at](https://www.affymetrix.com/LinkServlet?probeset=235916_at) | [YPEL4](http://www.ncbi.nlm.nih.gov/entrez/query.fcgi?cmd=search&db=gene&term=YPEL4) | yippee-like 4 (Drosophila) | 2.86e-05 | 0.00344 | 72.89 | 34.74 | 106.91 | (2, 1), (2, 3) |
| [201572_x_at](https://www.affymetrix.com/LinkServlet?probeset=201572_x_at) | [DCTD](http://www.ncbi.nlm.nih.gov/entrez/query.fcgi?cmd=search&db=gene&term=DCTD) | dCMP deaminase | 2.88e-05 | 0.00345 | 328.65 | 294.63 | 199.42 | (3, 1), (3, 2) |
| [242319_at](https://www.affymetrix.com/LinkServlet?probeset=242319_at) | [DGKG](http://www.ncbi.nlm.nih.gov/entrez/query.fcgi?cmd=search&db=gene&term=DGKG) | diacylglycerol kinase, gamma 90kDa | 2.92e-05 | 0.00346 | 8.09 | 6.24 | 24.9 | (1, 3), (2, 3) |
| [212897_at](https://www.affymetrix.com/LinkServlet?probeset=212897_at) | [CDC2L6](http://www.ncbi.nlm.nih.gov/entrez/query.fcgi?cmd=search&db=gene&term=CDC2L6) | cell division cycle 2-like 6 (CDK8-like) | 2.92e-05 | 0.00346 | 295.49 | 157.02 | 172.42 | (2, 1), (3, 1) |
| [228302_x_at](https://www.affymetrix.com/LinkServlet?probeset=228302_x_at) | [CAMK2N1](http://www.ncbi.nlm.nih.gov/entrez/query.fcgi?cmd=search&db=gene&term=CAMK2N1) | calcium/calmodulin-dependent protein kinase II inhibitor 1 | 2.96e-05 | 0.00347 | 209.09 | 90.08 | 277.83 | (2, 1), (2, 3) |
| [230264_s_at](https://www.affymetrix.com/LinkServlet?probeset=230264_s_at) | [AP1S2](http://www.ncbi.nlm.nih.gov/entrez/query.fcgi?cmd=search&db=gene&term=AP1S2) | adaptor-related protein complex 1, sigma 2 subunit | 2.97e-05 | 0.00347 | 3079.38 | 1396.2 | 1040.89 | (2, 1), (3, 1) |
| [225666_at](https://www.affymetrix.com/LinkServlet?probeset=225666_at) | [TMTC4](http://www.ncbi.nlm.nih.gov/entrez/query.fcgi?cmd=search&db=gene&term=TMTC4) | transmembrane and tetratricopeptide repeat containing 4 | 2.98e-05 | 0.00347 | 691.04 | 275.07 | 166.8 | (2, 1), (3, 1) |
| [206869_at](https://www.affymetrix.com/LinkServlet?probeset=206869_at) | [CHAD](http://www.ncbi.nlm.nih.gov/entrez/query.fcgi?cmd=search&db=gene&term=CHAD) | chondroadherin | 3.04e-05 | 0.00352 | 16.53 | 38.99 | 125.92 | (1, 3), (2, 3) |
| [233823_at](https://www.affymetrix.com/LinkServlet?probeset=233823_at) | [FAM184B](http://www.ncbi.nlm.nih.gov/entrez/query.fcgi?cmd=search&db=gene&term=FAM184B) | family with sequence similarity 184, member B | 3.07e-05 | 0.00353 | 50.78 | 9.71 | 36.73 | (2, 1), (2, 3) |
| [218889_at](https://www.affymetrix.com/LinkServlet?probeset=218889_at) | [NOC3L](http://www.ncbi.nlm.nih.gov/entrez/query.fcgi?cmd=search&db=gene&term=NOC3L) | nucleolar complex associated 3 homolog (S. cerevisiae) | 3.08e-05 | 0.00353 | 278.14 | 473.91 | 295.41 | (1, 2), (3, 2) |
| [229160_at](https://www.affymetrix.com/LinkServlet?probeset=229160_at) | [MUM1L1](http://www.ncbi.nlm.nih.gov/entrez/query.fcgi?cmd=search&db=gene&term=MUM1L1) | melanoma associated antigen (mutated) 1-like 1 | 3.22e-05 | 0.00367 | 9.9 | 16.86 | 43.05 | (1, 3), (2, 3) |
| [230773_at](https://www.affymetrix.com/LinkServlet?probeset=230773_at) | [NA](http://www.ncbi.nlm.nih.gov/entrez/query.fcgi?cmd=search&db=gene&term=NA) | NA | 3.32e-05 | 0.00377 | 21.63 | 40.33 | 72.84 | (1, 3) |
| [229989_at](https://www.affymetrix.com/LinkServlet?probeset=229989_at) | [FDXACB1](http://www.ncbi.nlm.nih.gov/entrez/query.fcgi?cmd=search&db=gene&term=FDXACB1) | ferredoxin-fold anticodon binding domain containing 1 | 3.4e-05 | 0.00384 | 10.03 | 15.42 | 9.34 | (1, 2), (3, 2) |
| [205529_s_at](https://www.affymetrix.com/LinkServlet?probeset=205529_s_at) | [RUNX1T1](http://www.ncbi.nlm.nih.gov/entrez/query.fcgi?cmd=search&db=gene&term=RUNX1T1) | runt-related transcription factor 1; translocated to, 1 (cyclin D-related) | 3.42e-05 | 0.00384 | 160.39 | 158.61 | 469.32 | (1, 3), (2, 3) |
| [235591_at](https://www.affymetrix.com/LinkServlet?probeset=235591_at) | [SSTR1](http://www.ncbi.nlm.nih.gov/entrez/query.fcgi?cmd=search&db=gene&term=SSTR1) | somatostatin receptor 1 | 3.46e-05 | 0.00386 | 31.35 | 31.37 | 203.32 | (1, 3), (2, 3) |
| [235465_at](https://www.affymetrix.com/LinkServlet?probeset=235465_at) | [FAM123A](http://www.ncbi.nlm.nih.gov/entrez/query.fcgi?cmd=search&db=gene&term=FAM123A) | family with sequence similarity 123A | 3.47e-05 | 0.00386 | 398.16 | 187.44 | 947.1 | (2, 3) |
| [208469_s_at](https://www.affymetrix.com/LinkServlet?probeset=208469_s_at) | [NA](http://www.ncbi.nlm.nih.gov/entrez/query.fcgi?cmd=search&db=gene&term=NA) | NA | 3.5e-05 | 0.00387 | 40.86 | 20.44 | 17.81 | (2, 1), (3, 1) |
| [227282_at](https://www.affymetrix.com/LinkServlet?probeset=227282_at) | [PCDH19](http://www.ncbi.nlm.nih.gov/entrez/query.fcgi?cmd=search&db=gene&term=PCDH19) | protocadherin 19 | 3.54e-05 | 0.00389 | 134.89 | 138.05 | 487.79 | (1, 3), (2, 3) |
| [242715_at](https://www.affymetrix.com/LinkServlet?probeset=242715_at) | [NA](http://www.ncbi.nlm.nih.gov/entrez/query.fcgi?cmd=search&db=gene&term=NA) | NA | 3.55e-05 | 0.00389 | 191.3 | 12.08 | 151.71 | (2, 1), (2, 3) |
| [230137_at](https://www.affymetrix.com/LinkServlet?probeset=230137_at) | [TMEM155](http://www.ncbi.nlm.nih.gov/entrez/query.fcgi?cmd=search&db=gene&term=TMEM155) | transmembrane protein 155 | 3.74e-05 | 0.00407 | 16.84 | 4.91 | 4.91 | (2, 1), (3, 1) |
| [221577_x_at](https://www.affymetrix.com/LinkServlet?probeset=221577_x_at) | [GDF15](http://www.ncbi.nlm.nih.gov/entrez/query.fcgi?cmd=search&db=gene&term=GDF15) | growth differentiation factor 15 | 3.75e-05 | 0.00407 | 170.26 | 45.01 | 20.84 | (2, 1), (3, 1) |
| [229441_at](https://www.affymetrix.com/LinkServlet?probeset=229441_at) | [PRSS23](http://www.ncbi.nlm.nih.gov/entrez/query.fcgi?cmd=search&db=gene&term=PRSS23) | protease, serine, 23 | 3.81e-05 | 0.00411 | 31.56 | 106.32 | 29.77 | (1, 2), (3, 2) |
| [201571_s_at](https://www.affymetrix.com/LinkServlet?probeset=201571_s_at) | [DCTD](http://www.ncbi.nlm.nih.gov/entrez/query.fcgi?cmd=search&db=gene&term=DCTD) | dCMP deaminase | 3.88e-05 | 0.00415 | 89.13 | 72.12 | 36.51 | (3, 1), (3, 2) |
| [212208_at](https://www.affymetrix.com/LinkServlet?probeset=212208_at) | [MED13L](http://www.ncbi.nlm.nih.gov/entrez/query.fcgi?cmd=search&db=gene&term=MED13L) | mediator complex subunit 13-like | 3.92e-05 | 0.00415 | 131.03 | 113.06 | 200.65 | (1, 3), (2, 3) |
| [228608_at](https://www.affymetrix.com/LinkServlet?probeset=228608_at) | [NALCN](http://www.ncbi.nlm.nih.gov/entrez/query.fcgi?cmd=search&db=gene&term=NALCN) | sodium leak channel, non-selective | 3.92e-05 | 0.00415 | 263.05 | 671.48 | 862.24 | (1, 2), (1, 3) |
| [220010_at](https://www.affymetrix.com/LinkServlet?probeset=220010_at) | [KCNE1L](http://www.ncbi.nlm.nih.gov/entrez/query.fcgi?cmd=search&db=gene&term=KCNE1L) | KCNE1-like | 3.92e-05 | 0.00415 | 13.49 | 19.87 | 64.57 | (1, 3), (2, 3) |
| [1555462_at](https://www.affymetrix.com/LinkServlet?probeset=1555462_at) | [PPP1R1C](http://www.ncbi.nlm.nih.gov/entrez/query.fcgi?cmd=search&db=gene&term=PPP1R1C) | protein phosphatase 1, regulatory (inhibitor) subunit 1C | 3.96e-05 | 0.00417 | 21.08 | 13.1 | 5.07 | (3, 1), (3, 2) |
| [219537_x_at](https://www.affymetrix.com/LinkServlet?probeset=219537_x_at) | [DLL3](http://www.ncbi.nlm.nih.gov/entrez/query.fcgi?cmd=search&db=gene&term=DLL3) | delta-like 3 (Drosophila) | 4.02e-05 | 0.00421 | 19.22 | 20.43 | 77.64 | (1, 3), (2, 3) |
| [209839_at](https://www.affymetrix.com/LinkServlet?probeset=209839_at) | [DNM3](http://www.ncbi.nlm.nih.gov/entrez/query.fcgi?cmd=search&db=gene&term=DNM3) | dynamin 3 | 4.06e-05 | 0.00422 | 457.9 | 271.61 | 801.16 | (2, 3) |
| [205862_at](https://www.affymetrix.com/LinkServlet?probeset=205862_at) | [GREB1](http://www.ncbi.nlm.nih.gov/entrez/query.fcgi?cmd=search&db=gene&term=GREB1) | GREB1 protein | 4.08e-05 | 0.00422 | 30.32 | 9.14 | 9.79 | (2, 1), (3, 1) |
| [230418_s_at](https://www.affymetrix.com/LinkServlet?probeset=230418_s_at) | [GALNTL1](http://www.ncbi.nlm.nih.gov/entrez/query.fcgi?cmd=search&db=gene&term=GALNTL1) | UDP-N-acetyl-alpha-D-galactosamine:polypeptide N-acetylgalactosaminyltransferase-like 1 | 4.09e-05 | 0.00422 | 102.02 | 131.27 | 383.07 | (1, 3), (2, 3) |
| [218284_at](https://www.affymetrix.com/LinkServlet?probeset=218284_at) | [SMAD3](http://www.ncbi.nlm.nih.gov/entrez/query.fcgi?cmd=search&db=gene&term=SMAD3) | SMAD family member 3 | 4.11e-05 | 0.00422 | 157.69 | 371.68 | 549.83 | (1, 2), (1, 3) |
| [235024_at](https://www.affymetrix.com/LinkServlet?probeset=235024_at) | [PHF17](http://www.ncbi.nlm.nih.gov/entrez/query.fcgi?cmd=search&db=gene&term=PHF17) | PHD finger protein 17 | 4.12e-05 | 0.00422 | 30.69 | 25.8 | 49.34 | (1, 3), (2, 3) |
| [241396_at](https://www.affymetrix.com/LinkServlet?probeset=241396_at) | [NEDD4L](http://www.ncbi.nlm.nih.gov/entrez/query.fcgi?cmd=search&db=gene&term=NEDD4L) | neural precursor cell expressed, developmentally down-regulated 4-like | 4.18e-05 | 0.00424 | 35 | 12.85 | 12.37 | (2, 1), (3, 1) |
| [210906_x_at](https://www.affymetrix.com/LinkServlet?probeset=210906_x_at) | [AQP4](http://www.ncbi.nlm.nih.gov/entrez/query.fcgi?cmd=search&db=gene&term=AQP4) | aquaporin 4 | 4.18e-05 | 0.00424 | 146.66 | 644.06 | 1098.3 | (1, 2), (1, 3) |
| [223278_at](https://www.affymetrix.com/LinkServlet?probeset=223278_at) | [GJB2](http://www.ncbi.nlm.nih.gov/entrez/query.fcgi?cmd=search&db=gene&term=GJB2) | gap junction protein, beta 2, 26kDa | 4.23e-05 | 0.00427 | 35.38 | 11.55 | 5.48 | (3, 1) |
| [1552767_a_at](https://www.affymetrix.com/LinkServlet?probeset=1552767_a_at) | [HS6ST2](http://www.ncbi.nlm.nih.gov/entrez/query.fcgi?cmd=search&db=gene&term=HS6ST2) | heparan sulfate 6-O-sulfotransferase 2 | 4.29e-05 | 0.00431 | 143.34 | 499.61 | 820.84 | (1, 2), (1, 3) |
| [206898_at](https://www.affymetrix.com/LinkServlet?probeset=206898_at) | [CDH19](http://www.ncbi.nlm.nih.gov/entrez/query.fcgi?cmd=search&db=gene&term=CDH19) | cadherin 19, type 2 | 4.32e-05 | 0.00432 | 1850.25 | 81.15 | 2329.29 | (2, 1), (2, 3) |
| [218870_at](https://www.affymetrix.com/LinkServlet?probeset=218870_at) | [ARHGAP15](http://www.ncbi.nlm.nih.gov/entrez/query.fcgi?cmd=search&db=gene&term=ARHGAP15) | Rho GTPase activating protein 15 | 4.42e-05 | 0.0044 | 174.35 | 206.95 | 88.64 | (3, 1), (3, 2) |
| [226690_at](https://www.affymetrix.com/LinkServlet?probeset=226690_at) | [ADCYAP1R1](http://www.ncbi.nlm.nih.gov/entrez/query.fcgi?cmd=search&db=gene&term=ADCYAP1R1) | adenylate cyclase activating polypeptide 1 (pituitary) receptor type I | 4.54e-05 | 0.0045 | 141.72 | 334.78 | 838.03 | (1, 3) |
| [226806_s_at](https://www.affymetrix.com/LinkServlet?probeset=226806_s_at) | [NFIA](http://www.ncbi.nlm.nih.gov/entrez/query.fcgi?cmd=search&db=gene&term=NFIA) | nuclear factor I/A | 4.57e-05 | 0.00451 | 2603.17 | 2515.4 | 4254.38 | (1, 3), (2, 3) |
| [244461_at](https://www.affymetrix.com/LinkServlet?probeset=244461_at) | [CYTSB](http://www.ncbi.nlm.nih.gov/entrez/query.fcgi?cmd=search&db=gene&term=CYTSB) | cytospin B | 4.66e-05 | 0.00458 | 41.1 | 50.64 | 24.29 | (3, 1), (3, 2) |
| [219557_s_at](https://www.affymetrix.com/LinkServlet?probeset=219557_s_at) | [NRIP3](http://www.ncbi.nlm.nih.gov/entrez/query.fcgi?cmd=search&db=gene&term=NRIP3) | nuclear receptor interacting protein 3 | 4.76e-05 | 0.00466 | 74.37 | 50.45 | 208.18 | (1, 3), (2, 3) |
| [209291_at](https://www.affymetrix.com/LinkServlet?probeset=209291_at) | [ID4](http://www.ncbi.nlm.nih.gov/entrez/query.fcgi?cmd=search&db=gene&term=ID4) | inhibitor of DNA binding 4, dominant negative helix-loop-helix protein | 4.95e-05 | 0.00481 | 5136.79 | 9993.02 | 11072.37 | (1, 2), (1, 3) |
| [240312_at](https://www.affymetrix.com/LinkServlet?probeset=240312_at) | [LOC389895](http://www.ncbi.nlm.nih.gov/entrez/query.fcgi?cmd=search&db=gene&term=LOC389895) | hypothetical LOC389895 | 4.98e-05 | 0.00481 | 10.7 | 9.34 | 28.42 | (1, 3), (2, 3) |
| [212240_s_at](https://www.affymetrix.com/LinkServlet?probeset=212240_s_at) | [PIK3R1](http://www.ncbi.nlm.nih.gov/entrez/query.fcgi?cmd=search&db=gene&term=PIK3R1) | phosphoinositide-3-kinase, regulatory subunit 1 (alpha) | 5e-05 | 0.00481 | 660.78 | 599.61 | 1245.74 | (1, 3), (2, 3) |
| [205081_at](https://www.affymetrix.com/LinkServlet?probeset=205081_at) | [CRIP1](http://www.ncbi.nlm.nih.gov/entrez/query.fcgi?cmd=search&db=gene&term=CRIP1) | cysteine-rich protein 1 (intestinal) | 5.01e-05 | 0.00481 | 114.87 | 80.99 | 32.64 | (3, 1), (3, 2) |
| [228827_at](https://www.affymetrix.com/LinkServlet?probeset=228827_at) | [NA](http://www.ncbi.nlm.nih.gov/entrez/query.fcgi?cmd=search&db=gene&term=NA) | NA | 5.15e-05 | 0.00491 | 121.45 | 167.89 | 347.41 | (1, 3), (2, 3) |
| [228444_at](https://www.affymetrix.com/LinkServlet?probeset=228444_at) | [NA](http://www.ncbi.nlm.nih.gov/entrez/query.fcgi?cmd=search&db=gene&term=NA) | NA | 5.16e-05 | 0.00491 | 28.36 | 20.46 | 15.4 | (3, 1) |
| [203732_at](https://www.affymetrix.com/LinkServlet?probeset=203732_at) | [TRIP4](http://www.ncbi.nlm.nih.gov/entrez/query.fcgi?cmd=search&db=gene&term=TRIP4) | thyroid hormone receptor interactor 4 | 5.18e-05 | 0.00491 | 196.48 | 245.66 | 148.84 | (3, 2) |
| [226137_at](https://www.affymetrix.com/LinkServlet?probeset=226137_at) | [ZFHX3](http://www.ncbi.nlm.nih.gov/entrez/query.fcgi?cmd=search&db=gene&term=ZFHX3) | zinc finger homeobox 3 | 5.23e-05 | 0.00494 | 605.8 | 431.86 | 305.29 | (3, 1) |
| [224995_at](https://www.affymetrix.com/LinkServlet?probeset=224995_at) | [SPIRE1](http://www.ncbi.nlm.nih.gov/entrez/query.fcgi?cmd=search&db=gene&term=SPIRE1) | spire homolog 1 (Drosophila) | 5.29e-05 | 0.00497 | 1847.47 | 1139.64 | 1980.02 | (2, 1), (2, 3) |
| [225368_at](https://www.affymetrix.com/LinkServlet?probeset=225368_at) | [HIPK2](http://www.ncbi.nlm.nih.gov/entrez/query.fcgi?cmd=search&db=gene&term=HIPK2) | homeodomain interacting protein kinase 2 | 5.32e-05 | 0.00498 | 5688.99 | 3190.59 | 6266.78 | (2, 1), (2, 3) |
| [235567_at](https://www.affymetrix.com/LinkServlet?probeset=235567_at) | [RORA](http://www.ncbi.nlm.nih.gov/entrez/query.fcgi?cmd=search&db=gene&term=RORA) | RAR-related orphan receptor A | 5.35e-05 | 0.00499 | 37.21 | 31.98 | 117.05 | (1, 3), (2, 3) |
| [206307_s_at](https://www.affymetrix.com/LinkServlet?probeset=206307_s_at) | [FOXD1](http://www.ncbi.nlm.nih.gov/entrez/query.fcgi?cmd=search&db=gene&term=FOXD1) | forkhead box D1 | 5.53e-05 | 0.00513 | 25.38 | 82.33 | 15.34 | (1, 2), (3, 2) |
| [204513_s_at](https://www.affymetrix.com/LinkServlet?probeset=204513_s_at) | [ELMO1](http://www.ncbi.nlm.nih.gov/entrez/query.fcgi?cmd=search&db=gene&term=ELMO1) | engulfment and cell motility 1 | 5.56e-05 | 0.00514 | 264.83 | 117.99 | 271.12 | (2, 1), (2, 3) |
| [206309_at](https://www.affymetrix.com/LinkServlet?probeset=206309_at) | [LECT1](http://www.ncbi.nlm.nih.gov/entrez/query.fcgi?cmd=search&db=gene&term=LECT1) | leukocyte cell derived chemotaxin 1 | 5.58e-05 | 0.00514 | 9.84 | 18.9 | 35.62 | (1, 3) |
| [204689_at](https://www.affymetrix.com/LinkServlet?probeset=204689_at) | [HHEX](http://www.ncbi.nlm.nih.gov/entrez/query.fcgi?cmd=search&db=gene&term=HHEX) | hematopoietically expressed homeobox | 5.68e-05 | 0.00517 | 41.07 | 61.78 | 27.94 | (3, 2) |
| [210137_s_at](https://www.affymetrix.com/LinkServlet?probeset=210137_s_at) | [DCTD](http://www.ncbi.nlm.nih.gov/entrez/query.fcgi?cmd=search&db=gene&term=DCTD) | dCMP deaminase | 5.68e-05 | 0.00517 | 251.85 | 240.89 | 154.64 | (3, 1), (3, 2) |
| [226228_at](https://www.affymetrix.com/LinkServlet?probeset=226228_at) | [AQP4](http://www.ncbi.nlm.nih.gov/entrez/query.fcgi?cmd=search&db=gene&term=AQP4) | aquaporin 4 | 5.69e-05 | 0.00517 | 3810.13 | 11246.98 | 15395.31 | (1, 2), (1, 3) |
| [218403_at](https://www.affymetrix.com/LinkServlet?probeset=218403_at) | [TRIAP1](http://www.ncbi.nlm.nih.gov/entrez/query.fcgi?cmd=search&db=gene&term=TRIAP1) | TP53 regulated inhibitor of apoptosis 1 | 5.72e-05 | 0.00517 | 773.84 | 891.11 | 555.84 | (3, 1), (3, 2) |
| [225020_at](https://www.affymetrix.com/LinkServlet?probeset=225020_at) | [DAB2IP](http://www.ncbi.nlm.nih.gov/entrez/query.fcgi?cmd=search&db=gene&term=DAB2IP) | DAB2 interacting protein | 5.75e-05 | 0.00517 | 114.66 | 69.27 | 139.98 | (2, 1), (2, 3) |
| [207232_s_at](https://www.affymetrix.com/LinkServlet?probeset=207232_s_at) | [DZIP3](http://www.ncbi.nlm.nih.gov/entrez/query.fcgi?cmd=search&db=gene&term=DZIP3) | DAZ interacting protein 3, zinc finger | 5.76e-05 | 0.00517 | 52.95 | 36.17 | 64.95 | (2, 3) |
| [230030_at](https://www.affymetrix.com/LinkServlet?probeset=230030_at) | [HS6ST2](http://www.ncbi.nlm.nih.gov/entrez/query.fcgi?cmd=search&db=gene&term=HS6ST2) | heparan sulfate 6-O-sulfotransferase 2 | 5.8e-05 | 0.00517 | 48.91 | 169 | 272.33 | (1, 2), (1, 3) |
| [228425_at](https://www.affymetrix.com/LinkServlet?probeset=228425_at) | [LOC654433](http://www.ncbi.nlm.nih.gov/entrez/query.fcgi?cmd=search&db=gene&term=LOC654433) | hypothetical LOC654433 | 5.8e-05 | 0.00517 | 21.1 | 14.11 | 5.04 | (3, 1), (3, 2) |
| [239155_at](https://www.affymetrix.com/LinkServlet?probeset=239155_at) | [NA](http://www.ncbi.nlm.nih.gov/entrez/query.fcgi?cmd=search&db=gene&term=NA) | NA | 5.87e-05 | 0.0052 | 61.55 | 32.71 | 15.75 | (3, 1), (3, 2) |
| [236692_at](https://www.affymetrix.com/LinkServlet?probeset=236692_at) | [LOC729839](http://www.ncbi.nlm.nih.gov/entrez/query.fcgi?cmd=search&db=gene&term=LOC729839) | similar to DTW domain containing 2 | 5.89e-05 | 0.0052 | 8.82 | 18.23 | 8.38 | (1, 2), (3, 2) |
| [229740_at](https://www.affymetrix.com/LinkServlet?probeset=229740_at) | [LOC643008](http://www.ncbi.nlm.nih.gov/entrez/query.fcgi?cmd=search&db=gene&term=LOC643008) | hypothetical protein LOC643008 | 5.94e-05 | 0.00523 | 87.05 | 85.37 | 26.72 | (3, 1), (3, 2) |
| [239340_at](https://www.affymetrix.com/LinkServlet?probeset=239340_at) | [NA](http://www.ncbi.nlm.nih.gov/entrez/query.fcgi?cmd=search&db=gene&term=NA) | NA | 6.13e-05 | 0.00532 | 77.57 | 8.52 | 55.51 | (2, 1), (2, 3) |
| [232874_at](https://www.affymetrix.com/LinkServlet?probeset=232874_at) | [DOCK9](http://www.ncbi.nlm.nih.gov/entrez/query.fcgi?cmd=search&db=gene&term=DOCK9) | dedicator of cytokinesis 9 | 6.15e-05 | 0.00532 | 31.42 | 26.85 | 122.92 | (1, 3), (2, 3) |
| [208296_x_at](https://www.affymetrix.com/LinkServlet?probeset=208296_x_at) | [TNFAIP8](http://www.ncbi.nlm.nih.gov/entrez/query.fcgi?cmd=search&db=gene&term=TNFAIP8) | tumor necrosis factor, alpha-induced protein 8 | 6.16e-05 | 0.00532 | 100.01 | 146.3 | 54.65 | (3, 1), (3, 2) |
| [1563561_at](https://www.affymetrix.com/LinkServlet?probeset=1563561_at) | [NA](http://www.ncbi.nlm.nih.gov/entrez/query.fcgi?cmd=search&db=gene&term=NA) | NA | 6.17e-05 | 0.00532 | 5.35 | 5.35 | 14.47 | (1, 3), (2, 3) |
| [200950_at](https://www.affymetrix.com/LinkServlet?probeset=200950_at) | [ARPC1A](http://www.ncbi.nlm.nih.gov/entrez/query.fcgi?cmd=search&db=gene&term=ARPC1A) | actin related protein 2/3 complex, subunit 1A, 41kDa | 6.19e-05 | 0.00532 | 1219.52 | 622.68 | 543.5 | (2, 1), (3, 1) |
| [243013_at](https://www.affymetrix.com/LinkServlet?probeset=243013_at) | [NA](http://www.ncbi.nlm.nih.gov/entrez/query.fcgi?cmd=search&db=gene&term=NA) | NA | 6.19e-05 | 0.00532 | 199.16 | 127.23 | 261.97 | (2, 3) |
| [206811_at](https://www.affymetrix.com/LinkServlet?probeset=206811_at) | [ADCY8](http://www.ncbi.nlm.nih.gov/entrez/query.fcgi?cmd=search&db=gene&term=ADCY8) | adenylate cyclase 8 (brain) | 6.25e-05 | 0.00535 | 15.69 | 9.96 | 49.58 | (1, 3), (2, 3) |
| [228415_at](https://www.affymetrix.com/LinkServlet?probeset=228415_at) | [AP1S2](http://www.ncbi.nlm.nih.gov/entrez/query.fcgi?cmd=search&db=gene&term=AP1S2) | adaptor-related protein complex 1, sigma 2 subunit | 6.37e-05 | 0.00543 | 511.75 | 145.92 | 165.9 | (2, 1), (3, 1) |
| [224975_at](https://www.affymetrix.com/LinkServlet?probeset=224975_at) | [NFIA](http://www.ncbi.nlm.nih.gov/entrez/query.fcgi?cmd=search&db=gene&term=NFIA) | nuclear factor I/A | 6.4e-05 | 0.00544 | 1557.08 | 1460.47 | 2456.56 | (1, 3), (2, 3) |
| [226525_at](https://www.affymetrix.com/LinkServlet?probeset=226525_at) | [STK17B](http://www.ncbi.nlm.nih.gov/entrez/query.fcgi?cmd=search&db=gene&term=STK17B) | serine/threonine kinase 17b | 6.44e-05 | 0.00545 | 164.7 | 277.87 | 383.76 | (1, 3) |
| [40560_at](https://www.affymetrix.com/LinkServlet?probeset=40560_at) | [TBX2](http://www.ncbi.nlm.nih.gov/entrez/query.fcgi?cmd=search&db=gene&term=TBX2) | T-box 2 | 6.55e-05 | 0.00552 | 54.53 | 17.46 | 13.22 | (2, 1), (3, 1) |
| [204959_at](https://www.affymetrix.com/LinkServlet?probeset=204959_at) | [MNDA](http://www.ncbi.nlm.nih.gov/entrez/query.fcgi?cmd=search&db=gene&term=MNDA) | myeloid cell nuclear differentiation antigen | 6.62e-05 | 0.00556 | 414.51 | 654.03 | 215.78 | (3, 2) |
| [228708_at](https://www.affymetrix.com/LinkServlet?probeset=228708_at) | [RAB27B](http://www.ncbi.nlm.nih.gov/entrez/query.fcgi?cmd=search&db=gene&term=RAB27B) | RAB27B, member RAS oncogene family | 6.65e-05 | 0.00556 | 156.36 | 29.62 | 210.95 | (2, 1), (2, 3) |
| [228598_at](https://www.affymetrix.com/LinkServlet?probeset=228598_at) | [DPP10](http://www.ncbi.nlm.nih.gov/entrez/query.fcgi?cmd=search&db=gene&term=DPP10) | dipeptidyl-peptidase 10 | 6.72e-05 | 0.0056 | 52.79 | 164.53 | 348.88 | (1, 3) |
| [1552430_at](https://www.affymetrix.com/LinkServlet?probeset=1552430_at) | [WDR17](http://www.ncbi.nlm.nih.gov/entrez/query.fcgi?cmd=search&db=gene&term=WDR17) | WD repeat domain 17 | 6.76e-05 | 0.00561 | 18.05 | 17.59 | 40.06 | (1, 3), (2, 3) |
| [230463_at](https://www.affymetrix.com/LinkServlet?probeset=230463_at) | [NA](http://www.ncbi.nlm.nih.gov/entrez/query.fcgi?cmd=search&db=gene&term=NA) | NA | 6.85e-05 | 0.00566 | 165.48 | 239.7 | 489.19 | (1, 3), (2, 3) |
| [227692_at](https://www.affymetrix.com/LinkServlet?probeset=227692_at) | [GNAI1](http://www.ncbi.nlm.nih.gov/entrez/query.fcgi?cmd=search&db=gene&term=GNAI1) | guanine nucleotide binding protein (G protein), alpha inhibiting activity polypeptide 1 | 6.87e-05 | 0.00566 | 379.48 | 966.6 | 1112.84 | (1, 2), (1, 3) |
| [206960_at](https://www.affymetrix.com/LinkServlet?probeset=206960_at) | [LPAR4](http://www.ncbi.nlm.nih.gov/entrez/query.fcgi?cmd=search&db=gene&term=LPAR4) | lysophosphatidic acid receptor 4 | 6.91e-05 | 0.00567 | 53.62 | 98.33 | 226.76 | (1, 3), (2, 3) |
| [204485_s_at](https://www.affymetrix.com/LinkServlet?probeset=204485_s_at) | [TOM1L1](http://www.ncbi.nlm.nih.gov/entrez/query.fcgi?cmd=search&db=gene&term=TOM1L1) | target of myb1 (chicken)-like 1 | 7.25e-05 | 0.00592 | 44.28 | 120.99 | 83.74 | (1, 2), (1, 3) |
| [232282_at](https://www.affymetrix.com/LinkServlet?probeset=232282_at) | [WNK3](http://www.ncbi.nlm.nih.gov/entrez/query.fcgi?cmd=search&db=gene&term=WNK3) | WNK lysine deficient protein kinase 3 | 7.27e-05 | 0.00592 | 252.25 | 162.42 | 392.07 | (2, 3) |
| [230563_at](https://www.affymetrix.com/LinkServlet?probeset=230563_at) | [RASGEF1A](http://www.ncbi.nlm.nih.gov/entrez/query.fcgi?cmd=search&db=gene&term=RASGEF1A) | RasGEF domain family, member 1A | 7.56e-05 | 0.00613 | 226.32 | 676.31 | 1568.8 | (1, 3) |
| [244231_at](https://www.affymetrix.com/LinkServlet?probeset=244231_at) | [LOC149684](http://www.ncbi.nlm.nih.gov/entrez/query.fcgi?cmd=search&db=gene&term=LOC149684) | hypothetical protein LOC149684 | 7.62e-05 | 0.00616 | 21.03 | 42.69 | 18.19 | (1, 2), (3, 2) |
| [230960_at](https://www.affymetrix.com/LinkServlet?probeset=230960_at) | [IGDCC3](http://www.ncbi.nlm.nih.gov/entrez/query.fcgi?cmd=search&db=gene&term=IGDCC3) | immunoglobulin superfamily, DCC subclass, member 3 | 7.65e-05 | 0.00616 | 42.48 | 11.61 | 21.68 | (2, 1), (3, 1), (2, 3) |
| [239146_at](https://www.affymetrix.com/LinkServlet?probeset=239146_at) | [CLDND1](http://www.ncbi.nlm.nih.gov/entrez/query.fcgi?cmd=search&db=gene&term=CLDND1) | claudin domain containing 1 | 7.72e-05 | 0.00619 | 17 | 14.78 | 7.85 | (3, 1), (3, 2) |
| [219090_at](https://www.affymetrix.com/LinkServlet?probeset=219090_at) | [SLC24A3](http://www.ncbi.nlm.nih.gov/entrez/query.fcgi?cmd=search&db=gene&term=SLC24A3) | solute carrier family 24 (sodium/potassium/calcium exchanger), member 3 | 7.74e-05 | 0.00619 | 115.21 | 155.17 | 385.73 | (1, 3), (2, 3) |
| [214460_at](https://www.affymetrix.com/LinkServlet?probeset=214460_at) | [LSAMP](http://www.ncbi.nlm.nih.gov/entrez/query.fcgi?cmd=search&db=gene&term=LSAMP) | limbic system-associated membrane protein | 7.78e-05 | 0.0062 | 230.94 | 207.3 | 431.13 | (1, 3), (2, 3) |
| [209987_s_at](https://www.affymetrix.com/LinkServlet?probeset=209987_s_at) | [ASCL1](http://www.ncbi.nlm.nih.gov/entrez/query.fcgi?cmd=search&db=gene&term=ASCL1) | achaete-scute complex homolog 1 (Drosophila) | 7.82e-05 | 0.00621 | 231.59 | 638.89 | 1407.29 | (1, 3) |
| [242628_at](https://www.affymetrix.com/LinkServlet?probeset=242628_at) | [KLRB1](http://www.ncbi.nlm.nih.gov/entrez/query.fcgi?cmd=search&db=gene&term=KLRB1) | killer cell lectin-like receptor subfamily B, member 1 | 7.87e-05 | 0.00622 | 9.79 | 10.69 | 33.27 | (1, 3), (2, 3) |
| [222787_s_at](https://www.affymetrix.com/LinkServlet?probeset=222787_s_at) | [TMEM106B](http://www.ncbi.nlm.nih.gov/entrez/query.fcgi?cmd=search&db=gene&term=TMEM106B) | transmembrane protein 106B | 8.06e-05 | 0.00635 | 755.72 | 744.21 | 1282.15 | (1, 3), (2, 3) |
| [229463_at](https://www.affymetrix.com/LinkServlet?probeset=229463_at) | [NTRK2](http://www.ncbi.nlm.nih.gov/entrez/query.fcgi?cmd=search&db=gene&term=NTRK2) | neurotrophic tyrosine kinase, receptor, type 2 | 8.13e-05 | 0.00635 | 61.68 | 35.1 | 118.47 | (2, 3) |
| [226261_at](https://www.affymetrix.com/LinkServlet?probeset=226261_at) | [ZNRF2](http://www.ncbi.nlm.nih.gov/entrez/query.fcgi?cmd=search&db=gene&term=ZNRF2) | zinc and ring finger 2 | 8.14e-05 | 0.00635 | 64.48 | 102.57 | 51.7 | (3, 2) |
| [203570_at](https://www.affymetrix.com/LinkServlet?probeset=203570_at) | [LOXL1](http://www.ncbi.nlm.nih.gov/entrez/query.fcgi?cmd=search&db=gene&term=LOXL1) | lysyl oxidase-like 1 | 8.14e-05 | 0.00635 | 124.4 | 259.93 | 52.52 | (3, 2) |
| [205126_at](https://www.affymetrix.com/LinkServlet?probeset=205126_at) | [VRK2](http://www.ncbi.nlm.nih.gov/entrez/query.fcgi?cmd=search&db=gene&term=VRK2) | vaccinia related kinase 2 | 8.39e-05 | 0.00652 | 108.67 | 249.49 | 157.77 | (1, 2), (3, 2) |
| [225241_at](https://www.affymetrix.com/LinkServlet?probeset=225241_at) | [CCDC80](http://www.ncbi.nlm.nih.gov/entrez/query.fcgi?cmd=search&db=gene&term=CCDC80) | coiled-coil domain containing 80 | 8.43e-05 | 0.00653 | 26.75 | 90.19 | 141.69 | (1, 2), (1, 3) |
| [236902_at](https://www.affymetrix.com/LinkServlet?probeset=236902_at) | [FLJ43390](http://www.ncbi.nlm.nih.gov/entrez/query.fcgi?cmd=search&db=gene&term=FLJ43390) | hypothetical LOC646113 | 8.47e-05 | 0.00653 | 15.9 | 9.35 | 52.39 | (1, 3), (2, 3) |
| [207857_at](https://www.affymetrix.com/LinkServlet?probeset=207857_at) | [LILRA2](http://www.ncbi.nlm.nih.gov/entrez/query.fcgi?cmd=search&db=gene&term=LILRA2) | leukocyte immunoglobulin-like receptor, subfamily A (with TM domain), member 2 | 8.52e-05 | 0.00655 | 151.27 | 178.72 | 80.35 | (3, 1), (3, 2) |
| [201556_s_at](https://www.affymetrix.com/LinkServlet?probeset=201556_s_at) | [VAMP2](http://www.ncbi.nlm.nih.gov/entrez/query.fcgi?cmd=search&db=gene&term=VAMP2) | vesicle-associated membrane protein 2 (synaptobrevin 2) | 8.55e-05 | 0.00655 | 58.65 | 29.74 | 50.82 | (2, 1), (2, 3) |
| [229132_at](https://www.affymetrix.com/LinkServlet?probeset=229132_at) | [MINA](http://www.ncbi.nlm.nih.gov/entrez/query.fcgi?cmd=search&db=gene&term=MINA) | MYC induced nuclear antigen | 8.86e-05 | 0.00676 | 31.02 | 81.3 | 54.46 | (1, 2), (1, 3) |
| [222891_s_at](https://www.affymetrix.com/LinkServlet?probeset=222891_s_at) | [BCL11A](http://www.ncbi.nlm.nih.gov/entrez/query.fcgi?cmd=search&db=gene&term=BCL11A) | B-cell CLL/lymphoma 11A (zinc finger protein) | 8.88e-05 | 0.00676 | 33.15 | 28.85 | 8.97 | (3, 1), (3, 2) |
| [204811_s_at](https://www.affymetrix.com/LinkServlet?probeset=204811_s_at) | [CACNA2D2](http://www.ncbi.nlm.nih.gov/entrez/query.fcgi?cmd=search&db=gene&term=CACNA2D2) | calcium channel, voltage-dependent, alpha 2/delta subunit 2 | 8.92e-05 | 0.00676 | 18.32 | 29.63 | 60.75 | (1, 3), (2, 3) |
| [225299_at](https://www.affymetrix.com/LinkServlet?probeset=225299_at) | [MYO5B](http://www.ncbi.nlm.nih.gov/entrez/query.fcgi?cmd=search&db=gene&term=MYO5B) | myosin VB | 8.97e-05 | 0.00678 | 23.3 | 5.68 | 6.13 | (2, 1), (3, 1) |
| [219686_at](https://www.affymetrix.com/LinkServlet?probeset=219686_at) | [STK32B](http://www.ncbi.nlm.nih.gov/entrez/query.fcgi?cmd=search&db=gene&term=STK32B) | serine/threonine kinase 32B | 9.2e-05 | 0.00693 | 71.57 | 219.57 | 391.86 | (1, 2), (1, 3) |
| [243299_at](https://www.affymetrix.com/LinkServlet?probeset=243299_at) | [VRK2](http://www.ncbi.nlm.nih.gov/entrez/query.fcgi?cmd=search&db=gene&term=VRK2) | vaccinia related kinase 2 | 9.28e-05 | 0.00695 | 7.13 | 12.75 | 6.66 | (1, 2), (3, 2) |
| [225102_at](https://www.affymetrix.com/LinkServlet?probeset=225102_at) | [MGLL](http://www.ncbi.nlm.nih.gov/entrez/query.fcgi?cmd=search&db=gene&term=MGLL) | monoglyceride lipase | 9.3e-05 | 0.00695 | 619.57 | 694.44 | 1701.41 | (1, 3), (2, 3) |
| [242172_at](https://www.affymetrix.com/LinkServlet?probeset=242172_at) | [MEIS1](http://www.ncbi.nlm.nih.gov/entrez/query.fcgi?cmd=search&db=gene&term=MEIS1) | Meis homeobox 1 | 9.46e-05 | 0.00705 | 12.27 | 7.72 | 32.44 | (1, 3), (2, 3) |
| [224690_at](https://www.affymetrix.com/LinkServlet?probeset=224690_at) | [C20orf108](http://www.ncbi.nlm.nih.gov/entrez/query.fcgi?cmd=search&db=gene&term=C20orf108) | chromosome 20 open reading frame 108 | 9.54e-05 | 0.00707 | 1469.58 | 1002.48 | 842.54 | (2, 1), (3, 1) |
| [230417_at](https://www.affymetrix.com/LinkServlet?probeset=230417_at) | [GALNTL1](http://www.ncbi.nlm.nih.gov/entrez/query.fcgi?cmd=search&db=gene&term=GALNTL1) | UDP-N-acetyl-alpha-D-galactosamine:polypeptide N-acetylgalactosaminyltransferase-like 1 | 9.56e-05 | 0.00707 | 26.18 | 29.4 | 81.51 | (1, 3), (2, 3) |
| [206715_at](https://www.affymetrix.com/LinkServlet?probeset=206715_at) | [TFEC](http://www.ncbi.nlm.nih.gov/entrez/query.fcgi?cmd=search&db=gene&term=TFEC) | transcription factor EC | 9.58e-05 | 0.00707 | 33.36 | 70.01 | 16.33 | (3, 2) |
| [211484_s_at](https://www.affymetrix.com/LinkServlet?probeset=211484_s_at) | [DSCAM](http://www.ncbi.nlm.nih.gov/entrez/query.fcgi?cmd=search&db=gene&term=DSCAM) | Down syndrome cell adhesion molecule | 9.69e-05 | 0.00712 | 89.25 | 55.33 | 246.92 | (1, 3), (2, 3) |
| [204602_at](https://www.affymetrix.com/LinkServlet?probeset=204602_at) | [DKK1](http://www.ncbi.nlm.nih.gov/entrez/query.fcgi?cmd=search&db=gene&term=DKK1) | dickkopf homolog 1 (Xenopus laevis) | 9.71e-05 | 0.00712 | 60.5 | 26.58 | 5.71 | (3, 1), (3, 2) |
| [204831_at](https://www.affymetrix.com/LinkServlet?probeset=204831_at) | [CDK8](http://www.ncbi.nlm.nih.gov/entrez/query.fcgi?cmd=search&db=gene&term=CDK8) | cyclin-dependent kinase 8 | 9.78e-05 | 0.00714 | 152.16 | 248.34 | 399.47 | (1, 3) |
| [206243_at](https://www.affymetrix.com/LinkServlet?probeset=206243_at) | [TIMP4](http://www.ncbi.nlm.nih.gov/entrez/query.fcgi?cmd=search&db=gene&term=TIMP4) | TIMP metallopeptidase inhibitor 4 | 9.82e-05 | 0.00715 | 539.39 | 1047 | 1988.13 | (1, 3) |
| [205442_at](https://www.affymetrix.com/LinkServlet?probeset=205442_at) | [MFAP3L](http://www.ncbi.nlm.nih.gov/entrez/query.fcgi?cmd=search&db=gene&term=MFAP3L) | microfibrillar-associated protein 3-like | 9.85e-05 | 0.00715 | 1496.5 | 917.78 | 2224.08 | (2, 3) |
| [235639_at](https://www.affymetrix.com/LinkServlet?probeset=235639_at) | [NA](http://www.ncbi.nlm.nih.gov/entrez/query.fcgi?cmd=search&db=gene&term=NA) | NA | 0.0001003 | 0.00724 | 359.83 | 21.98 | 431.43 | (2, 1), (2, 3) |
| [209826_at](https://www.affymetrix.com/LinkServlet?probeset=209826_at) | [PPT2](http://www.ncbi.nlm.nih.gov/entrez/query.fcgi?cmd=search&db=gene&term=PPT2) | palmitoyl-protein thioesterase 2 | 0.0001005 | 0.00724 | 130.12 | 62.51 | 42.02 | (3, 1) |
| [219331_s_at](https://www.affymetrix.com/LinkServlet?probeset=219331_s_at) | [KLHDC8A](http://www.ncbi.nlm.nih.gov/entrez/query.fcgi?cmd=search&db=gene&term=KLHDC8A) | kelch domain containing 8A | 0.000101 | 0.00726 | 65.47 | 62.72 | 23.85 | (3, 1), (3, 2) |
| [207012_at](https://www.affymetrix.com/LinkServlet?probeset=207012_at) | [MMP16](http://www.ncbi.nlm.nih.gov/entrez/query.fcgi?cmd=search&db=gene&term=MMP16) | matrix metallopeptidase 16 (membrane-inserted) | 0.0001021 | 0.00731 | 42.18 | 108.36 | 179.56 | (1, 3) |
| [212816_s_at](https://www.affymetrix.com/LinkServlet?probeset=212816_s_at) | [CBS](http://www.ncbi.nlm.nih.gov/entrez/query.fcgi?cmd=search&db=gene&term=CBS) | cystathionine-beta-synthase | 0.000103 | 0.00735 | 57.33 | 45.2 | 95.14 | (1, 3), (2, 3) |
| [202295_s_at](https://www.affymetrix.com/LinkServlet?probeset=202295_s_at) | [CTSH](http://www.ncbi.nlm.nih.gov/entrez/query.fcgi?cmd=search&db=gene&term=CTSH) | cathepsin H | 0.0001044 | 0.00741 | 770.8 | 1608.79 | 638.8 | (1, 2), (3, 2) |
| [232805_at](https://www.affymetrix.com/LinkServlet?probeset=232805_at) | [NA](http://www.ncbi.nlm.nih.gov/entrez/query.fcgi?cmd=search&db=gene&term=NA) | NA | 0.0001046 | 0.00741 | 48.86 | 128.29 | 265.94 | (1, 3) |
| [219821_s_at](https://www.affymetrix.com/LinkServlet?probeset=219821_s_at) | [GFOD1](http://www.ncbi.nlm.nih.gov/entrez/query.fcgi?cmd=search&db=gene&term=GFOD1) | glucose-fructose oxidoreductase domain containing 1 | 0.0001049 | 0.00741 | 200.35 | 85.78 | 187.55 | (2, 1), (2, 3) |
| [224397_s_at](https://www.affymetrix.com/LinkServlet?probeset=224397_s_at) | [TMTC1](http://www.ncbi.nlm.nih.gov/entrez/query.fcgi?cmd=search&db=gene&term=TMTC1) | transmembrane and tetratricopeptide repeat containing 1 | 0.0001068 | 0.00748 | 16.12 | 40.67 | 13.73 | (1, 2), (3, 2) |
| [227657_at](https://www.affymetrix.com/LinkServlet?probeset=227657_at) | [RNF150](http://www.ncbi.nlm.nih.gov/entrez/query.fcgi?cmd=search&db=gene&term=RNF150) | ring finger protein 150 | 0.0001069 | 0.00748 | 128.41 | 163.98 | 307.61 | (1, 3), (2, 3) |
| [232553_at](https://www.affymetrix.com/LinkServlet?probeset=232553_at) | [PCYT1B](http://www.ncbi.nlm.nih.gov/entrez/query.fcgi?cmd=search&db=gene&term=PCYT1B) | phosphate cytidylyltransferase 1, choline, beta | 0.0001069 | 0.00748 | 62.66 | 23.01 | 51.35 | (2, 1), (2, 3) |
| [244849_at](https://www.affymetrix.com/LinkServlet?probeset=244849_at) | [SEMA3A](http://www.ncbi.nlm.nih.gov/entrez/query.fcgi?cmd=search&db=gene&term=SEMA3A) | sema domain, immunoglobulin domain (Ig), short basic domain, secreted, (semaphorin) 3A | 0.000108 | 0.00753 | 5.87 | 15.59 | 5.2 | (1, 2), (3, 2) |
| [52940_at](https://www.affymetrix.com/LinkServlet?probeset=52940_at) | [SIGIRR](http://www.ncbi.nlm.nih.gov/entrez/query.fcgi?cmd=search&db=gene&term=SIGIRR) | single immunoglobulin and toll-interleukin 1 receptor (TIR) domain | 0.0001083 | 0.00753 | 102.3 | 167.5 | 64.11 | (3, 2) |
| [212642_s_at](https://www.affymetrix.com/LinkServlet?probeset=212642_s_at) | [HIVEP2](http://www.ncbi.nlm.nih.gov/entrez/query.fcgi?cmd=search&db=gene&term=HIVEP2) | human immunodeficiency virus type I enhancer binding protein 2 | 0.0001091 | 0.00754 | 380.5 | 249.87 | 488.47 | (2, 3) |
| [221587_s_at](https://www.affymetrix.com/LinkServlet?probeset=221587_s_at) | [C19orf24](http://www.ncbi.nlm.nih.gov/entrez/query.fcgi?cmd=search&db=gene&term=C19orf24) | chromosome 19 open reading frame 24 | 0.0001094 | 0.00754 | 45.88 | 47.02 | 28.99 | (3, 1), (3, 2) |
| [204081_at](https://www.affymetrix.com/LinkServlet?probeset=204081_at) | [NRGN](http://www.ncbi.nlm.nih.gov/entrez/query.fcgi?cmd=search&db=gene&term=NRGN) | neurogranin (protein kinase C substrate, RC3) | 0.0001095 | 0.00754 | 64 | 18.11 | 11.41 | (2, 1), (3, 1) |
| [205363_at](https://www.affymetrix.com/LinkServlet?probeset=205363_at) | [BBOX1](http://www.ncbi.nlm.nih.gov/entrez/query.fcgi?cmd=search&db=gene&term=BBOX1) | butyrobetaine (gamma), 2-oxoglutarate dioxygenase (gamma-butyrobetaine hydroxylase) 1 | 0.0001106 | 0.0076 | 167.76 | 92.53 | 555.36 | (1, 3), (2, 3) |
| [206984_s_at](https://www.affymetrix.com/LinkServlet?probeset=206984_s_at) | [RIT2](http://www.ncbi.nlm.nih.gov/entrez/query.fcgi?cmd=search&db=gene&term=RIT2) | Ras-like without CAAX 2 | 0.0001118 | 0.00765 | 160.58 | 164.39 | 831.82 | (1, 3), (2, 3) |
| [209047_at](https://www.affymetrix.com/LinkServlet?probeset=209047_at) | [AQP1](http://www.ncbi.nlm.nih.gov/entrez/query.fcgi?cmd=search&db=gene&term=AQP1) | aquaporin 1 (Colton blood group) | 0.0001141 | 0.00779 | 125.08 | 138.19 | 1022.89 | (1, 3), (2, 3) |
| [227474_at](https://www.affymetrix.com/LinkServlet?probeset=227474_at) | [LOC654433](http://www.ncbi.nlm.nih.gov/entrez/query.fcgi?cmd=search&db=gene&term=LOC654433) | hypothetical LOC654433 | 0.0001165 | 0.00793 | 30.78 | 28.08 | 10.52 | (3, 1), (3, 2) |
| [204215_at](https://www.affymetrix.com/LinkServlet?probeset=204215_at) | [C7orf23](http://www.ncbi.nlm.nih.gov/entrez/query.fcgi?cmd=search&db=gene&term=C7orf23) | chromosome 7 open reading frame 23 | 0.0001173 | 0.00796 | 247.03 | 410.43 | 175.96 | (3, 2) |
| [204570_at](https://www.affymetrix.com/LinkServlet?probeset=204570_at) | [COX7A1](http://www.ncbi.nlm.nih.gov/entrez/query.fcgi?cmd=search&db=gene&term=COX7A1) | cytochrome c oxidase subunit VIIa polypeptide 1 (muscle) | 0.0001194 | 0.00806 | 582.63 | 480.93 | 134.65 | (3, 1), (3, 2) |
| [204082_at](https://www.affymetrix.com/LinkServlet?probeset=204082_at) | [PBX3](http://www.ncbi.nlm.nih.gov/entrez/query.fcgi?cmd=search&db=gene&term=PBX3) | pre-B-cell leukemia homeobox 3 | 0.0001196 | 0.00806 | 270.05 | 361.99 | 559.81 | (1, 3), (2, 3) |
| [209310_s_at](https://www.affymetrix.com/LinkServlet?probeset=209310_s_at) | [CASP4](http://www.ncbi.nlm.nih.gov/entrez/query.fcgi?cmd=search&db=gene&term=CASP4) | caspase 4, apoptosis-related cysteine peptidase | 0.000121 | 0.00813 | 68.06 | 135.51 | 40.5 | (3, 2) |
| [215014_at](https://www.affymetrix.com/LinkServlet?probeset=215014_at) | [KCND3](http://www.ncbi.nlm.nih.gov/entrez/query.fcgi?cmd=search&db=gene&term=KCND3) | potassium voltage-gated channel, Shal-related subfamily, member 3 | 0.0001224 | 0.00818 | 144.74 | 180.79 | 367.16 | (1, 3), (2, 3) |
| [228481_at](https://www.affymetrix.com/LinkServlet?probeset=228481_at) | [NA](http://www.ncbi.nlm.nih.gov/entrez/query.fcgi?cmd=search&db=gene&term=NA) | NA | 0.0001225 | 0.00818 | 12.61 | 90.11 | 15.44 | (1, 2), (3, 2) |
| [230413_s_at](https://www.affymetrix.com/LinkServlet?probeset=230413_s_at) | [NA](http://www.ncbi.nlm.nih.gov/entrez/query.fcgi?cmd=search&db=gene&term=NA) | NA | 0.0001251 | 0.00833 | 515.42 | 207.92 | 152.14 | (2, 1), (3, 1) |
| [218844_at](https://www.affymetrix.com/LinkServlet?probeset=218844_at) | [ACSF2](http://www.ncbi.nlm.nih.gov/entrez/query.fcgi?cmd=search&db=gene&term=ACSF2) | acyl-CoA synthetase family member 2 | 0.0001259 | 0.00836 | 67.46 | 119.11 | 247.87 | (1, 3), (2, 3) |
| [201242_s_at](https://www.affymetrix.com/LinkServlet?probeset=201242_s_at) | [ATP1B1](http://www.ncbi.nlm.nih.gov/entrez/query.fcgi?cmd=search&db=gene&term=ATP1B1) | ATPase, Na+/K+ transporting, beta 1 polypeptide | 0.0001274 | 0.00843 | 3098.74 | 1333.85 | 3128.74 | (2, 1), (2, 3) |
| [239132_at](https://www.affymetrix.com/LinkServlet?probeset=239132_at) | [NOS1](http://www.ncbi.nlm.nih.gov/entrez/query.fcgi?cmd=search&db=gene&term=NOS1) | nitric oxide synthase 1 (neuronal) | 0.0001288 | 0.00847 | 80.14 | 10.27 | 175.27 | (2, 1), (2, 3) |
| [230644_at](https://www.affymetrix.com/LinkServlet?probeset=230644_at) | [LRFN5](http://www.ncbi.nlm.nih.gov/entrez/query.fcgi?cmd=search&db=gene&term=LRFN5) | leucine rich repeat and fibronectin type III domain containing 5 | 0.0001288 | 0.00847 | 17.69 | 88.46 | 111.37 | (1, 2), (1, 3) |
| [227314_at](https://www.affymetrix.com/LinkServlet?probeset=227314_at) | [ITGA2](http://www.ncbi.nlm.nih.gov/entrez/query.fcgi?cmd=search&db=gene&term=ITGA2) | integrin, alpha 2 (CD49B, alpha 2 subunit of VLA-2 receptor) | 0.0001297 | 0.00851 | 32.86 | 12.66 | 7.27 | (3, 1) |
| [218589_at](https://www.affymetrix.com/LinkServlet?probeset=218589_at) | [LPAR6](http://www.ncbi.nlm.nih.gov/entrez/query.fcgi?cmd=search&db=gene&term=LPAR6) | lysophosphatidic acid receptor 6 | 0.000131 | 0.00851 | 551.78 | 876.17 | 281.01 | (3, 2) |
| [210260_s_at](https://www.affymetrix.com/LinkServlet?probeset=210260_s_at) | [TNFAIP8](http://www.ncbi.nlm.nih.gov/entrez/query.fcgi?cmd=search&db=gene&term=TNFAIP8) | tumor necrosis factor, alpha-induced protein 8 | 0.0001311 | 0.00851 | 134.52 | 221.86 | 75.85 | (3, 2) |
| [227632_at](https://www.affymetrix.com/LinkServlet?probeset=227632_at) | [TBC1D24](http://www.ncbi.nlm.nih.gov/entrez/query.fcgi?cmd=search&db=gene&term=TBC1D24) | TBC1 domain family, member 24 | 0.0001314 | 0.00851 | 53.32 | 44.18 | 96.35 | (1, 3), (2, 3) |
| [232000_at](https://www.affymetrix.com/LinkServlet?probeset=232000_at) | [TTC39B](http://www.ncbi.nlm.nih.gov/entrez/query.fcgi?cmd=search&db=gene&term=TTC39B) | tetratricopeptide repeat domain 39B | 0.0001317 | 0.00851 | 81.09 | 184.06 | 194.66 | (1, 2), (1, 3) |
| [242521_at](https://www.affymetrix.com/LinkServlet?probeset=242521_at) | [NA](http://www.ncbi.nlm.nih.gov/entrez/query.fcgi?cmd=search&db=gene&term=NA) | NA | 0.0001317 | 0.00851 | 141.35 | 226.83 | 91.2 | (3, 2) |
| [231015_at](https://www.affymetrix.com/LinkServlet?probeset=231015_at) | [KLF15](http://www.ncbi.nlm.nih.gov/entrez/query.fcgi?cmd=search&db=gene&term=KLF15) | Kruppel-like factor 15 | 0.0001325 | 0.00854 | 219.58 | 153.27 | 387.21 | (2, 3) |
| [236038_at](https://www.affymetrix.com/LinkServlet?probeset=236038_at) | [NA](http://www.ncbi.nlm.nih.gov/entrez/query.fcgi?cmd=search&db=gene&term=NA) | NA | 0.0001333 | 0.00855 | 206.62 | 324.66 | 518.75 | (1, 3) |
| [204897_at](https://www.affymetrix.com/LinkServlet?probeset=204897_at) | [PTGER4](http://www.ncbi.nlm.nih.gov/entrez/query.fcgi?cmd=search&db=gene&term=PTGER4) | prostaglandin E receptor 4 (subtype EP4) | 0.0001334 | 0.00855 | 504.91 | 728.12 | 201.86 | (3, 1), (3, 2) |
| [222942_s_at](https://www.affymetrix.com/LinkServlet?probeset=222942_s_at) | [TIAM2](http://www.ncbi.nlm.nih.gov/entrez/query.fcgi?cmd=search&db=gene&term=TIAM2) | T-cell lymphoma invasion and metastasis 2 | 0.0001346 | 0.0086 | 72.94 | 53.61 | 204.18 | (1, 3), (2, 3) |
| [237939_at](https://www.affymetrix.com/LinkServlet?probeset=237939_at) | [EPHA5](http://www.ncbi.nlm.nih.gov/entrez/query.fcgi?cmd=search&db=gene&term=EPHA5) | EPH receptor A5 | 0.0001355 | 0.00862 | 37.4 | 16.47 | 110.24 | (2, 3) |
| [215311_at](https://www.affymetrix.com/LinkServlet?probeset=215311_at) | [NTRK3](http://www.ncbi.nlm.nih.gov/entrez/query.fcgi?cmd=search&db=gene&term=NTRK3) | neurotrophic tyrosine kinase, receptor, type 3 | 0.0001359 | 0.00862 | 390.93 | 440.03 | 1333.53 | (1, 3), (2, 3) |
| [224916_at](https://www.affymetrix.com/LinkServlet?probeset=224916_at) | [TMEM173](http://www.ncbi.nlm.nih.gov/entrez/query.fcgi?cmd=search&db=gene&term=TMEM173) | transmembrane protein 173 | 0.0001362 | 0.00862 | 15.78 | 20.54 | 11.54 | (3, 2) |
| [218921_at](https://www.affymetrix.com/LinkServlet?probeset=218921_at) | [SIGIRR](http://www.ncbi.nlm.nih.gov/entrez/query.fcgi?cmd=search&db=gene&term=SIGIRR) | single immunoglobulin and toll-interleukin 1 receptor (TIR) domain | 0.0001367 | 0.00863 | 46.33 | 75.74 | 28.56 | (3, 2) |
| [237268_at](https://www.affymetrix.com/LinkServlet?probeset=237268_at) | [DSCAM](http://www.ncbi.nlm.nih.gov/entrez/query.fcgi?cmd=search&db=gene&term=DSCAM) | Down syndrome cell adhesion molecule | 0.000138 | 0.00868 | 107.44 | 107.91 | 379.96 | (1, 3), (2, 3) |
| [212538_at](https://www.affymetrix.com/LinkServlet?probeset=212538_at) | [DOCK9](http://www.ncbi.nlm.nih.gov/entrez/query.fcgi?cmd=search&db=gene&term=DOCK9) | dedicator of cytokinesis 9 | 0.0001386 | 0.00868 | 478.01 | 377.63 | 829.8 | (1, 3), (2, 3) |
| [220134_x_at](https://www.affymetrix.com/LinkServlet?probeset=220134_x_at) | [FAM176B](http://www.ncbi.nlm.nih.gov/entrez/query.fcgi?cmd=search&db=gene&term=FAM176B) | family with sequence similarity 176, member B | 0.0001392 | 0.00868 | 89.35 | 84.6 | 52.52 | (3, 1), (3, 2) |
| [231489_x_at](https://www.affymetrix.com/LinkServlet?probeset=231489_x_at) | [NA](http://www.ncbi.nlm.nih.gov/entrez/query.fcgi?cmd=search&db=gene&term=NA) | NA | 0.0001394 | 0.00868 | 16.77 | 4.83 | 4.92 | (2, 1), (3, 1) |
| [204072_s_at](https://www.affymetrix.com/LinkServlet?probeset=204072_s_at) | [FRY](http://www.ncbi.nlm.nih.gov/entrez/query.fcgi?cmd=search&db=gene&term=FRY) | furry homolog (Drosophila) | 0.0001394 | 0.00868 | 555.33 | 635.29 | 1028.38 | (1, 3), (2, 3) |
| [228799_at](https://www.affymetrix.com/LinkServlet?probeset=228799_at) | [NA](http://www.ncbi.nlm.nih.gov/entrez/query.fcgi?cmd=search&db=gene&term=NA) | NA | 0.0001408 | 0.00874 | 56.79 | 91.37 | 34.42 | (3, 2) |
| [240218_at](https://www.affymetrix.com/LinkServlet?probeset=240218_at) | [DSCAM](http://www.ncbi.nlm.nih.gov/entrez/query.fcgi?cmd=search&db=gene&term=DSCAM) | Down syndrome cell adhesion molecule | 0.0001417 | 0.00876 | 97.91 | 90.72 | 353.24 | (1, 3), (2, 3) |
| [232752_at](https://www.affymetrix.com/LinkServlet?probeset=232752_at) | [NA](http://www.ncbi.nlm.nih.gov/entrez/query.fcgi?cmd=search&db=gene&term=NA) | NA | 0.000142 | 0.00876 | 7.57 | 12.28 | 4.99 | (3, 2) |
| [225928_at](https://www.affymetrix.com/LinkServlet?probeset=225928_at) | [NA](http://www.ncbi.nlm.nih.gov/entrez/query.fcgi?cmd=search&db=gene&term=NA) | NA | 0.0001427 | 0.00877 | 64.83 | 60.57 | 39.73 | (3, 1), (3, 2) |
| [228218_at](https://www.affymetrix.com/LinkServlet?probeset=228218_at) | [NA](http://www.ncbi.nlm.nih.gov/entrez/query.fcgi?cmd=search&db=gene&term=NA) | NA | 0.0001435 | 0.00877 | 767.02 | 809.51 | 1334.04 | (1, 3), (2, 3) |
| [239671_at](https://www.affymetrix.com/LinkServlet?probeset=239671_at) | [NA](http://www.ncbi.nlm.nih.gov/entrez/query.fcgi?cmd=search&db=gene&term=NA) | NA | 0.0001438 | 0.00877 | 182.6 | 101.46 | 446.14 | (2, 3) |
| [228066_at](https://www.affymetrix.com/LinkServlet?probeset=228066_at) | [C17orf96](http://www.ncbi.nlm.nih.gov/entrez/query.fcgi?cmd=search&db=gene&term=C17orf96) | chromosome 17 open reading frame 96 | 0.000144 | 0.00877 | 44.5 | 18.64 | 21.82 | (2, 1), (3, 1) |
| [209472_at](https://www.affymetrix.com/LinkServlet?probeset=209472_at) | [CCBL2](http://www.ncbi.nlm.nih.gov/entrez/query.fcgi?cmd=search&db=gene&term=CCBL2) | cysteine conjugate-beta lyase 2 | 0.0001442 | 0.00877 | 522.04 | 469.99 | 328.03 | (3, 1), (3, 2) |
| [213373_s_at](https://www.affymetrix.com/LinkServlet?probeset=213373_s_at) | [CASP8](http://www.ncbi.nlm.nih.gov/entrez/query.fcgi?cmd=search&db=gene&term=CASP8) | caspase 8, apoptosis-related cysteine peptidase | 0.0001445 | 0.00877 | 122.78 | 174.39 | 74.12 | (3, 2) |
| [225097_at](https://www.affymetrix.com/LinkServlet?probeset=225097_at) | [HIPK2](http://www.ncbi.nlm.nih.gov/entrez/query.fcgi?cmd=search&db=gene&term=HIPK2) | homeodomain interacting protein kinase 2 | 0.0001469 | 0.00889 | 1363.17 | 519.03 | 1425.28 | (2, 1), (2, 3) |
| [211026_s_at](https://www.affymetrix.com/LinkServlet?probeset=211026_s_at) | [MGLL](http://www.ncbi.nlm.nih.gov/entrez/query.fcgi?cmd=search&db=gene&term=MGLL) | monoglyceride lipase | 0.0001478 | 0.00891 | 688.86 | 557.75 | 1689.96 | (1, 3), (2, 3) |
| [226415_at](https://www.affymetrix.com/LinkServlet?probeset=226415_at) | [VAT1L](http://www.ncbi.nlm.nih.gov/entrez/query.fcgi?cmd=search&db=gene&term=VAT1L) | vesicle amine transport protein 1 homolog (T. californica)-like | 0.000148 | 0.00891 | 529.9 | 607.48 | 1444.34 | (1, 3), (2, 3) |
| [207103_at](https://www.affymetrix.com/LinkServlet?probeset=207103_at) | [KCND2](http://www.ncbi.nlm.nih.gov/entrez/query.fcgi?cmd=search&db=gene&term=KCND2) | potassium voltage-gated channel, Shal-related subfamily, member 2 | 0.0001506 | 0.00904 | 104.44 | 235.47 | 773.52 | (1, 3), (2, 3) |
| [225918_at](https://www.affymetrix.com/LinkServlet?probeset=225918_at) | [GLG1](http://www.ncbi.nlm.nih.gov/entrez/query.fcgi?cmd=search&db=gene&term=GLG1) | golgi apparatus protein 1 | 0.0001515 | 0.00906 | 529.69 | 327.48 | 643.65 | (2, 3) |
| [1557433_at](https://www.affymetrix.com/LinkServlet?probeset=1557433_at) | [NA](http://www.ncbi.nlm.nih.gov/entrez/query.fcgi?cmd=search&db=gene&term=NA) | NA | 0.0001517 | 0.00906 | 63.17 | 89.44 | 119.36 | (1, 3) |
| [225018_at](https://www.affymetrix.com/LinkServlet?probeset=225018_at) | [SPIRE1](http://www.ncbi.nlm.nih.gov/entrez/query.fcgi?cmd=search&db=gene&term=SPIRE1) | spire homolog 1 (Drosophila) | 0.0001532 | 0.00911 | 276.19 | 175.97 | 294.2 | (2, 1), (2, 3) |
| [210839_s_at](https://www.affymetrix.com/LinkServlet?probeset=210839_s_at) | [ENPP2](http://www.ncbi.nlm.nih.gov/entrez/query.fcgi?cmd=search&db=gene&term=ENPP2) | ectonucleotide pyrophosphatase/phosphodiesterase 2 | 0.0001535 | 0.00911 | 174.5 | 15.46 | 38.17 | (2, 1), (3, 1) |
| [220112_at](https://www.affymetrix.com/LinkServlet?probeset=220112_at) | [ANKRD55](http://www.ncbi.nlm.nih.gov/entrez/query.fcgi?cmd=search&db=gene&term=ANKRD55) | ankyrin repeat domain 55 | 0.0001542 | 0.00911 | 18.63 | 127.87 | 76.17 | (1, 2), (1, 3) |
| [221710_x_at](https://www.affymetrix.com/LinkServlet?probeset=221710_x_at) | [FAM176B](http://www.ncbi.nlm.nih.gov/entrez/query.fcgi?cmd=search&db=gene&term=FAM176B) | family with sequence similarity 176, member B | 0.0001542 | 0.00911 | 93.41 | 90.23 | 57.22 | (3, 1), (3, 2) |
| [227949_at](https://www.affymetrix.com/LinkServlet?probeset=227949_at) | [PHACTR3](http://www.ncbi.nlm.nih.gov/entrez/query.fcgi?cmd=search&db=gene&term=PHACTR3) | phosphatase and actin regulator 3 | 0.000155 | 0.00913 | 210.91 | 451.69 | 1004.67 | (1, 3) |
| [238212_at](https://www.affymetrix.com/LinkServlet?probeset=238212_at) | [NA](http://www.ncbi.nlm.nih.gov/entrez/query.fcgi?cmd=search&db=gene&term=NA) | NA | 0.0001567 | 0.00915 | 33.27 | 5.63 | 6.1 | (2, 1), (3, 1) |
| [214981_at](https://www.affymetrix.com/LinkServlet?probeset=214981_at) | [POSTN](http://www.ncbi.nlm.nih.gov/entrez/query.fcgi?cmd=search&db=gene&term=POSTN) | periostin, osteoblast specific factor | 0.0001568 | 0.00915 | 17.49 | 138.57 | 27.57 | (1, 2), (3, 2) |
| [235657_at](https://www.affymetrix.com/LinkServlet?probeset=235657_at) | [NA](http://www.ncbi.nlm.nih.gov/entrez/query.fcgi?cmd=search&db=gene&term=NA) | NA | 0.0001572 | 0.00915 | 19.08 | 26.18 | 13.46 | (3, 2) |
| [214633_at](https://www.affymetrix.com/LinkServlet?probeset=214633_at) | [SOX3](http://www.ncbi.nlm.nih.gov/entrez/query.fcgi?cmd=search&db=gene&term=SOX3) | SRY (sex determining region Y)-box 3 | 0.0001572 | 0.00915 | 14.46 | 14.5 | 36.25 | (1, 3), (2, 3) |
| [219355_at](https://www.affymetrix.com/LinkServlet?probeset=219355_at) | [CXorf57](http://www.ncbi.nlm.nih.gov/entrez/query.fcgi?cmd=search&db=gene&term=CXorf57) | chromosome X open reading frame 57 | 0.0001575 | 0.00915 | 61.03 | 65.19 | 209.52 | (1, 3), (2, 3) |
| [1557345_at](https://www.affymetrix.com/LinkServlet?probeset=1557345_at) | [LOC283516](http://www.ncbi.nlm.nih.gov/entrez/query.fcgi?cmd=search&db=gene&term=LOC283516) | hypothetical protein LOC283516 | 0.0001584 | 0.00918 | 6.32 | 6.84 | 10.51 | (1, 3), (2, 3) |
| [209789_at](https://www.affymetrix.com/LinkServlet?probeset=209789_at) | [CORO2B](http://www.ncbi.nlm.nih.gov/entrez/query.fcgi?cmd=search&db=gene&term=CORO2B) | coronin, actin binding protein, 2B | 0.0001601 | 0.00926 | 1116.71 | 315.49 | 259.18 | (2, 1), (3, 1) |
| [207242_s_at](https://www.affymetrix.com/LinkServlet?probeset=207242_s_at) | [GRIK1](http://www.ncbi.nlm.nih.gov/entrez/query.fcgi?cmd=search&db=gene&term=GRIK1) | glutamate receptor, ionotropic, kainate 1 | 0.0001608 | 0.00927 | 52.32 | 85.33 | 191.31 | (1, 3), (2, 3) |
| [234314_at](https://www.affymetrix.com/LinkServlet?probeset=234314_at) | [C20orf74](http://www.ncbi.nlm.nih.gov/entrez/query.fcgi?cmd=search&db=gene&term=C20orf74) | chromosome 20 open reading frame 74 | 0.0001614 | 0.00928 | 6.66 | 5.15 | 63.96 | (1, 3), (2, 3) |
| [221900_at](https://www.affymetrix.com/LinkServlet?probeset=221900_at) | [COL8A2](http://www.ncbi.nlm.nih.gov/entrez/query.fcgi?cmd=search&db=gene&term=COL8A2) | collagen, type VIII, alpha 2 | 0.0001626 | 0.00933 | 26.54 | 61.02 | 132.65 | (1, 3) |
| [241833_at](https://www.affymetrix.com/LinkServlet?probeset=241833_at) | [NA](http://www.ncbi.nlm.nih.gov/entrez/query.fcgi?cmd=search&db=gene&term=NA) | NA | 0.0001636 | 0.00935 | 92.74 | 6.48 | 28.13 | (2, 1), (2, 3) |
| [218829_s_at](https://www.affymetrix.com/LinkServlet?probeset=218829_s_at) | [CHD7](http://www.ncbi.nlm.nih.gov/entrez/query.fcgi?cmd=search&db=gene&term=CHD7) | chromodomain helicase DNA binding protein 7 | 0.0001639 | 0.00935 | 443.9 | 496.37 | 807.19 | (1, 3), (2, 3) |
| [218409_s_at](https://www.affymetrix.com/LinkServlet?probeset=218409_s_at) | [DNAJC1](http://www.ncbi.nlm.nih.gov/entrez/query.fcgi?cmd=search&db=gene&term=DNAJC1) | DnaJ (Hsp40) homolog, subfamily C, member 1 | 0.0001666 | 0.00947 | 304.56 | 405.2 | 212.37 | (3, 2) |
| [215025_at](https://www.affymetrix.com/LinkServlet?probeset=215025_at) | [NTRK3](http://www.ncbi.nlm.nih.gov/entrez/query.fcgi?cmd=search&db=gene&term=NTRK3) | neurotrophic tyrosine kinase, receptor, type 3 | 0.0001668 | 0.00947 | 26.15 | 50.8 | 78.65 | (1, 3) |
| [242524_at](https://www.affymetrix.com/LinkServlet?probeset=242524_at) | [CBLN4](http://www.ncbi.nlm.nih.gov/entrez/query.fcgi?cmd=search&db=gene&term=CBLN4) | cerebellin 4 precursor | 0.0001676 | 0.00949 | 20.86 | 11.58 | 5.91 | (3, 1) |
| [219054_at](https://www.affymetrix.com/LinkServlet?probeset=219054_at) | [C5orf23](http://www.ncbi.nlm.nih.gov/entrez/query.fcgi?cmd=search&db=gene&term=C5orf23) | chromosome 5 open reading frame 23 | 0.0001681 | 0.00949 | 18.28 | 7.07 | 6.65 | (2, 1), (3, 1) |
| [223842_s_at](https://www.affymetrix.com/LinkServlet?probeset=223842_s_at) | [SCARA3](http://www.ncbi.nlm.nih.gov/entrez/query.fcgi?cmd=search&db=gene&term=SCARA3) | scavenger receptor class A, member 3 | 0.0001686 | 0.0095 | 29.62 | 30.4 | 79.97 | (1, 3), (2, 3) |
| [228602_at](https://www.affymetrix.com/LinkServlet?probeset=228602_at) | [SGCD](http://www.ncbi.nlm.nih.gov/entrez/query.fcgi?cmd=search&db=gene&term=SGCD) | sarcoglycan, delta (35kDa dystrophin-associated glycoprotein) | 0.0001702 | 0.00955 | 98.34 | 113.98 | 241.44 | (1, 3), (2, 3) |
| [236373_at](https://www.affymetrix.com/LinkServlet?probeset=236373_at) | [NA](http://www.ncbi.nlm.nih.gov/entrez/query.fcgi?cmd=search&db=gene&term=NA) | NA | 0.0001706 | 0.00955 | 30.09 | 52.52 | 185.58 | (1, 3), (2, 3) |
| [239726_at](https://www.affymetrix.com/LinkServlet?probeset=239726_at) | [ANK3](http://www.ncbi.nlm.nih.gov/entrez/query.fcgi?cmd=search&db=gene&term=ANK3) | ankyrin 3, node of Ranvier (ankyrin G) | 0.0001708 | 0.00955 | 34.74 | 66.19 | 154.25 | (1, 3) |
| [206456_at](https://www.affymetrix.com/LinkServlet?probeset=206456_at) | [GABRA5](http://www.ncbi.nlm.nih.gov/entrez/query.fcgi?cmd=search&db=gene&term=GABRA5) | gamma-aminobutyric acid (GABA) A receptor, alpha 5 | 0.0001728 | 0.00963 | 18.16 | 7.19 | 4.78 | (2, 1), (3, 1) |
| [209617_s_at](https://www.affymetrix.com/LinkServlet?probeset=209617_s_at) | [CTNND2](http://www.ncbi.nlm.nih.gov/entrez/query.fcgi?cmd=search&db=gene&term=CTNND2) | catenin (cadherin-associated protein), delta 2 (neural plakophilin-related arm-repeat protein) | 0.0001738 | 0.00964 | 1411.58 | 673.45 | 1535.76 | (2, 1), (2, 3) |
| [204341_at](https://www.affymetrix.com/LinkServlet?probeset=204341_at) | [TRIM16](http://www.ncbi.nlm.nih.gov/entrez/query.fcgi?cmd=search&db=gene&term=TRIM16) | tripartite motif-containing 16 | 0.0001745 | 0.00964 | 67.75 | 82.6 | 43.03 | (3, 1), (3, 2) |
| [203911_at](https://www.affymetrix.com/LinkServlet?probeset=203911_at) | [RAP1GAP](http://www.ncbi.nlm.nih.gov/entrez/query.fcgi?cmd=search&db=gene&term=RAP1GAP) | RAP1 GTPase activating protein | 0.0001748 | 0.00964 | 32.04 | 28.39 | 90.37 | (1, 3), (2, 3) |
| [227607_at](https://www.affymetrix.com/LinkServlet?probeset=227607_at) | [STAMBPL1](http://www.ncbi.nlm.nih.gov/entrez/query.fcgi?cmd=search&db=gene&term=STAMBPL1) | STAM binding protein-like 1 | 0.0001754 | 0.00964 | 359.18 | 1138.07 | 677.34 | (1, 2), (1, 3) |
| [229890_at](https://www.affymetrix.com/LinkServlet?probeset=229890_at) | [PRRT1](http://www.ncbi.nlm.nih.gov/entrez/query.fcgi?cmd=search&db=gene&term=PRRT1) | proline-rich transmembrane protein 1 | 0.0001757 | 0.00964 | 220.59 | 87.13 | 226.56 | (2, 1), (2, 3) |
| [225384_at](https://www.affymetrix.com/LinkServlet?probeset=225384_at) | [DOCK7](http://www.ncbi.nlm.nih.gov/entrez/query.fcgi?cmd=search&db=gene&term=DOCK7) | dedicator of cytokinesis 7 | 0.0001758 | 0.00964 | 856.72 | 1064.31 | 470.82 | (3, 1), (3, 2) |
| [213888_s_at](https://www.affymetrix.com/LinkServlet?probeset=213888_s_at) | [TRAF3IP3](http://www.ncbi.nlm.nih.gov/entrez/query.fcgi?cmd=search&db=gene&term=TRAF3IP3) | TRAF3 interacting protein 3 | 0.000176 | 0.00964 | 41.6 | 80.98 | 33.8 | (1, 2), (3, 2) |
| [226056_at](https://www.affymetrix.com/LinkServlet?probeset=226056_at) | [CDGAP](http://www.ncbi.nlm.nih.gov/entrez/query.fcgi?cmd=search&db=gene&term=CDGAP) | Cdc42 GTPase-activating protein | 0.0001791 | 0.00978 | 428.69 | 204.78 | 425.38 | (2, 1), (2, 3) |
| [219497_s_at](https://www.affymetrix.com/LinkServlet?probeset=219497_s_at) | [BCL11A](http://www.ncbi.nlm.nih.gov/entrez/query.fcgi?cmd=search&db=gene&term=BCL11A) | B-cell CLL/lymphoma 11A (zinc finger protein) | 0.0001794 | 0.00978 | 20.71 | 20.92 | 6.64 | (3, 1), (3, 2) |
| [213600_at](https://www.affymetrix.com/LinkServlet?probeset=213600_at) | [SIPA1L3](http://www.ncbi.nlm.nih.gov/entrez/query.fcgi?cmd=search&db=gene&term=SIPA1L3) | signal-induced proliferation-associated 1 like 3 | 0.0001808 | 0.00983 | 61.97 | 50.95 | 80.46 | (2, 3) |
| [232276_at](https://www.affymetrix.com/LinkServlet?probeset=232276_at) | [HS6ST3](http://www.ncbi.nlm.nih.gov/entrez/query.fcgi?cmd=search&db=gene&term=HS6ST3) | heparan sulfate 6-O-sulfotransferase 3 | 0.0001813 | 0.00983 | 57.42 | 49.08 | 189.65 | (1, 3), (2, 3) |
| [243756_at](https://www.affymetrix.com/LinkServlet?probeset=243756_at) | [NA](http://www.ncbi.nlm.nih.gov/entrez/query.fcgi?cmd=search&db=gene&term=NA) | NA | 0.0001818 | 0.00984 | 16.32 | 16.85 | 70.87 | (1, 3), (2, 3) |
| [201341_at](https://www.affymetrix.com/LinkServlet?probeset=201341_at) | [ENC1](http://www.ncbi.nlm.nih.gov/entrez/query.fcgi?cmd=search&db=gene&term=ENC1) | ectodermal-neural cortex (with BTB-like domain) | 0.0001823 | 0.00984 | 430.53 | 219.13 | 150.74 | (3, 1) |
| [208650_s_at](https://www.affymetrix.com/LinkServlet?probeset=208650_s_at) | [CD24](http://www.ncbi.nlm.nih.gov/entrez/query.fcgi?cmd=search&db=gene&term=CD24) | CD24 molecule | 0.0001828 | 0.00984 | 29 | 7.5 | 5.55 | (2, 1), (3, 1) |
| [204203_at](https://www.affymetrix.com/LinkServlet?probeset=204203_at) | [CEBPG](http://www.ncbi.nlm.nih.gov/entrez/query.fcgi?cmd=search&db=gene&term=CEBPG) | CCAAT/enhancer binding protein (C/EBP), gamma | 0.0001845 | 0.00991 | 151.92 | 207.06 | 106.05 | (3, 2) |
| [1558692_at](https://www.affymetrix.com/LinkServlet?probeset=1558692_at) | [C1orf85](http://www.ncbi.nlm.nih.gov/entrez/query.fcgi?cmd=search&db=gene&term=C1orf85) | chromosome 1 open reading frame 85 | 0.0001856 | 0.00992 | 189.4 | 243.94 | 130.21 | (3, 2) |
| [206144_at](https://www.affymetrix.com/LinkServlet?probeset=206144_at) | [MAGI1](http://www.ncbi.nlm.nih.gov/entrez/query.fcgi?cmd=search&db=gene&term=MAGI1) | membrane associated guanylate kinase, WW and PDZ domain containing 1 | 0.0001857 | 0.00992 | 39.41 | 18.34 | 70.29 | (2, 3) |
| [215983_s_at](https://www.affymetrix.com/LinkServlet?probeset=215983_s_at) | [UBXN8](http://www.ncbi.nlm.nih.gov/entrez/query.fcgi?cmd=search&db=gene&term=UBXN8) | UBX domain protein 8 | 0.000187 | 0.00997 | 222.74 | 292.92 | 134.64 | (3, 2) |
| [230924_at](https://www.affymetrix.com/LinkServlet?probeset=230924_at) | [TTLL6](http://www.ncbi.nlm.nih.gov/entrez/query.fcgi?cmd=search&db=gene&term=TTLL6) | tubulin tyrosine ligase-like family, member 6 | 0.0001877 | 0.00998 | 15.55 | 4.83 | 5.09 | (2, 1), (3, 1) |
| [212848_s_at](https://www.affymetrix.com/LinkServlet?probeset=212848_s_at) | [C9orf3](http://www.ncbi.nlm.nih.gov/entrez/query.fcgi?cmd=search&db=gene&term=C9orf3) | chromosome 9 open reading frame 3 | 0.0001895 | 0.0101 | 146.97 | 362.86 | 167.55 | (1, 2), (3, 2) |
| [203820_s_at](https://www.affymetrix.com/LinkServlet?probeset=203820_s_at) | [IGF2BP3](http://www.ncbi.nlm.nih.gov/entrez/query.fcgi?cmd=search&db=gene&term=IGF2BP3) | insulin-like growth factor 2 mRNA binding protein 3 | 0.0001902 | 0.0101 | 24.94 | 166.23 | 19.44 | (1, 2), (3, 2) |
| [227061_at](https://www.affymetrix.com/LinkServlet?probeset=227061_at) | [NA](http://www.ncbi.nlm.nih.gov/entrez/query.fcgi?cmd=search&db=gene&term=NA) | NA | 0.0001911 | 0.0101 | 24.67 | 113.84 | 156.91 | (1, 2), (1, 3) |
| [220231_at](https://www.affymetrix.com/LinkServlet?probeset=220231_at) | [C7orf16](http://www.ncbi.nlm.nih.gov/entrez/query.fcgi?cmd=search&db=gene&term=C7orf16) | chromosome 7 open reading frame 16 | 0.0001917 | 0.0101 | 6.78 | 35.91 | 41.49 | (1, 2), (1, 3) |
| [228742_at](https://www.affymetrix.com/LinkServlet?probeset=228742_at) | [NA](http://www.ncbi.nlm.nih.gov/entrez/query.fcgi?cmd=search&db=gene&term=NA) | NA | 0.0001962 | 0.0103 | 12.16 | 29.38 | 84.16 | (1, 3) |
| [225445_at](https://www.affymetrix.com/LinkServlet?probeset=225445_at) | [UBN2](http://www.ncbi.nlm.nih.gov/entrez/query.fcgi?cmd=search&db=gene&term=UBN2) | ubinuclein 2 | 0.0001965 | 0.0103 | 271.21 | 254.41 | 458.35 | (1, 3), (2, 3) |
| [212098_at](https://www.affymetrix.com/LinkServlet?probeset=212098_at) | [NA](http://www.ncbi.nlm.nih.gov/entrez/query.fcgi?cmd=search&db=gene&term=NA) | NA | 0.0001976 | 0.0103 | 756.22 | 1416.88 | 1436.54 | (1, 2), (1, 3) |
| [232833_at](https://www.affymetrix.com/LinkServlet?probeset=232833_at) | [NA](http://www.ncbi.nlm.nih.gov/entrez/query.fcgi?cmd=search&db=gene&term=NA) | NA | 0.000198 | 0.0103 | 34.47 | 119.55 | 130.61 | (1, 2), (1, 3) |
| [213793_s_at](https://www.affymetrix.com/LinkServlet?probeset=213793_s_at) | [HOMER1](http://www.ncbi.nlm.nih.gov/entrez/query.fcgi?cmd=search&db=gene&term=HOMER1) | homer homolog 1 (Drosophila) | 0.0001995 | 0.0104 | 984.81 | 454.41 | 1002.95 | (2, 1), (2, 3) |
| [223614_at](https://www.affymetrix.com/LinkServlet?probeset=223614_at) | [MMP16](http://www.ncbi.nlm.nih.gov/entrez/query.fcgi?cmd=search&db=gene&term=MMP16) | matrix metallopeptidase 16 (membrane-inserted) | 0.0002034 | 0.0106 | 602.14 | 2091.59 | 2881.51 | (1, 2), (1, 3) |
| [210279_at](https://www.affymetrix.com/LinkServlet?probeset=210279_at) | [GPR18](http://www.ncbi.nlm.nih.gov/entrez/query.fcgi?cmd=search&db=gene&term=GPR18) | G protein-coupled receptor 18 | 0.0002045 | 0.0106 | 6.31 | 8.85 | 5.6 | (1, 2), (3, 2) |
| [228240_at](https://www.affymetrix.com/LinkServlet?probeset=228240_at) | [NA](http://www.ncbi.nlm.nih.gov/entrez/query.fcgi?cmd=search&db=gene&term=NA) | NA | 0.0002055 | 0.0106 | 311.52 | 156.47 | 292.67 | (2, 1), (2, 3) |
| [229233_at](https://www.affymetrix.com/LinkServlet?probeset=229233_at) | [NRG3](http://www.ncbi.nlm.nih.gov/entrez/query.fcgi?cmd=search&db=gene&term=NRG3) | neuregulin 3 | 0.0002057 | 0.0106 | 30.64 | 66.02 | 117.75 | (1, 3) |
| [211070_x_at](https://www.affymetrix.com/LinkServlet?probeset=211070_x_at) | [DBI](http://www.ncbi.nlm.nih.gov/entrez/query.fcgi?cmd=search&db=gene&term=DBI) | diazepam binding inhibitor (GABA receptor modulator, acyl-Coenzyme A binding protein) | 0.0002061 | 0.0106 | 8445.03 | 5282.87 | 8670.1 | (2, 1), (2, 3) |
| [224770_s_at](https://www.affymetrix.com/LinkServlet?probeset=224770_s_at) | [NAV1](http://www.ncbi.nlm.nih.gov/entrez/query.fcgi?cmd=search&db=gene&term=NAV1) | neuron navigator 1 | 0.0002102 | 0.0108 | 31.93 | 17.81 | 49.81 | (2, 3) |
| [239907_at](https://www.affymetrix.com/LinkServlet?probeset=239907_at) | [NA](http://www.ncbi.nlm.nih.gov/entrez/query.fcgi?cmd=search&db=gene&term=NA) | NA | 0.0002106 | 0.0108 | 43.32 | 55.45 | 204.24 | (1, 3), (2, 3) |
| [234103_at](https://www.affymetrix.com/LinkServlet?probeset=234103_at) | [KCNT2](http://www.ncbi.nlm.nih.gov/entrez/query.fcgi?cmd=search&db=gene&term=KCNT2) | potassium channel, subfamily T, member 2 | 0.0002128 | 0.0109 | 37.73 | 115.33 | 226.98 | (1, 3) |
| [240735_at](https://www.affymetrix.com/LinkServlet?probeset=240735_at) | [CDC42BPA](http://www.ncbi.nlm.nih.gov/entrez/query.fcgi?cmd=search&db=gene&term=CDC42BPA) | CDC42 binding protein kinase alpha (DMPK-like) | 0.0002137 | 0.0109 | 7.47 | 8.04 | 18.48 | (1, 3), (2, 3) |
| [205594_at](https://www.affymetrix.com/LinkServlet?probeset=205594_at) | [ZNF652](http://www.ncbi.nlm.nih.gov/entrez/query.fcgi?cmd=search&db=gene&term=ZNF652) | zinc finger protein 652 | 0.000214 | 0.0109 | 210.43 | 100.34 | 204.4 | (2, 1), (2, 3) |
| [205173_x_at](https://www.affymetrix.com/LinkServlet?probeset=205173_x_at) | [CD58](http://www.ncbi.nlm.nih.gov/entrez/query.fcgi?cmd=search&db=gene&term=CD58) | CD58 molecule | 0.0002148 | 0.0109 | 629.73 | 655.3 | 261.28 | (3, 1), (3, 2) |
| [213030_s_at](https://www.affymetrix.com/LinkServlet?probeset=213030_s_at) | [PLXNA2](http://www.ncbi.nlm.nih.gov/entrez/query.fcgi?cmd=search&db=gene&term=PLXNA2) | plexin A2 | 0.0002178 | 0.011 | 75.79 | 33.87 | 94.71 | (2, 1), (2, 3) |
| [238009_at](https://www.affymetrix.com/LinkServlet?probeset=238009_at) | [NA](http://www.ncbi.nlm.nih.gov/entrez/query.fcgi?cmd=search&db=gene&term=NA) | NA | 0.0002189 | 0.011 | 545.69 | 439.64 | 890.72 | (1, 3), (2, 3) |
| [214844_s_at](https://www.affymetrix.com/LinkServlet?probeset=214844_s_at) | [DOK5](http://www.ncbi.nlm.nih.gov/entrez/query.fcgi?cmd=search&db=gene&term=DOK5) | docking protein 5 | 0.0002193 | 0.011 | 466.13 | 981.69 | 344.31 | (3, 2) |
| [227835_at](https://www.affymetrix.com/LinkServlet?probeset=227835_at) | [NA](http://www.ncbi.nlm.nih.gov/entrez/query.fcgi?cmd=search&db=gene&term=NA) | NA | 0.0002202 | 0.011 | 23.85 | 9.32 | 6.78 | (2, 1), (3, 1) |
| [206690_at](https://www.affymetrix.com/LinkServlet?probeset=206690_at) | [ACCN1](http://www.ncbi.nlm.nih.gov/entrez/query.fcgi?cmd=search&db=gene&term=ACCN1) | amiloride-sensitive cation channel 1, neuronal | 0.0002205 | 0.011 | 36.23 | 44.77 | 92.63 | (1, 3), (2, 3) |
| [219406_at](https://www.affymetrix.com/LinkServlet?probeset=219406_at) | [C1orf50](http://www.ncbi.nlm.nih.gov/entrez/query.fcgi?cmd=search&db=gene&term=C1orf50) | chromosome 1 open reading frame 50 | 0.0002208 | 0.011 | 152.66 | 165.28 | 108.44 | (3, 1), (3, 2) |
| [229323_at](https://www.affymetrix.com/LinkServlet?probeset=229323_at) | [LOC387723](http://www.ncbi.nlm.nih.gov/entrez/query.fcgi?cmd=search&db=gene&term=LOC387723) | similar to hCG1648656 | 0.000221 | 0.011 | 23.5 | 34.25 | 17.62 | (3, 2) |
| [203851_at](https://www.affymetrix.com/LinkServlet?probeset=203851_at) | [IGFBP6](http://www.ncbi.nlm.nih.gov/entrez/query.fcgi?cmd=search&db=gene&term=IGFBP6) | insulin-like growth factor binding protein 6 | 0.0002211 | 0.011 | 227.13 | 539.77 | 104.87 | (3, 2) |
| [203608_at](https://www.affymetrix.com/LinkServlet?probeset=203608_at) | [ALDH5A1](http://www.ncbi.nlm.nih.gov/entrez/query.fcgi?cmd=search&db=gene&term=ALDH5A1) | aldehyde dehydrogenase 5 family, member A1 | 0.0002221 | 0.011 | 1014.28 | 895.12 | 1668.17 | (1, 3), (2, 3) |
| [220032_at](https://www.affymetrix.com/LinkServlet?probeset=220032_at) | [C7orf58](http://www.ncbi.nlm.nih.gov/entrez/query.fcgi?cmd=search&db=gene&term=C7orf58) | chromosome 7 open reading frame 58 | 0.0002238 | 0.0111 | 11.01 | 16.49 | 9.9 | (1, 2), (3, 2) |
| [1563217_at](https://www.affymetrix.com/LinkServlet?probeset=1563217_at) | [NA](http://www.ncbi.nlm.nih.gov/entrez/query.fcgi?cmd=search&db=gene&term=NA) | NA | 0.0002257 | 0.0112 | 19.34 | 25.58 | 70.11 | (1, 3), (2, 3) |
| [214217_at](https://www.affymetrix.com/LinkServlet?probeset=214217_at) | [GRM5](http://www.ncbi.nlm.nih.gov/entrez/query.fcgi?cmd=search&db=gene&term=GRM5) | glutamate receptor, metabotropic 5 | 0.0002269 | 0.0112 | 96.37 | 543.62 | 939.23 | (1, 2), (1, 3) |
| [214285_at](https://www.affymetrix.com/LinkServlet?probeset=214285_at) | [FABP3](http://www.ncbi.nlm.nih.gov/entrez/query.fcgi?cmd=search&db=gene&term=FABP3) | fatty acid binding protein 3, muscle and heart (mammary-derived growth inhibitor) | 0.000229 | 0.0113 | 13.93 | 38.12 | 11.95 | (1, 2), (3, 2) |
| [239272_at](https://www.affymetrix.com/LinkServlet?probeset=239272_at) | [MMP28](http://www.ncbi.nlm.nih.gov/entrez/query.fcgi?cmd=search&db=gene&term=MMP28) | matrix metallopeptidase 28 | 0.0002293 | 0.0113 | 19.54 | 19.93 | 8.52 | (3, 1), (3, 2) |
| [235288_at](https://www.affymetrix.com/LinkServlet?probeset=235288_at) | [NA](http://www.ncbi.nlm.nih.gov/entrez/query.fcgi?cmd=search&db=gene&term=NA) | NA | 0.0002306 | 0.0113 | 57.3 | 31.03 | 58.82 | (2, 1), (2, 3) |
| [223611_s_at](https://www.affymetrix.com/LinkServlet?probeset=223611_s_at) | [LNX1](http://www.ncbi.nlm.nih.gov/entrez/query.fcgi?cmd=search&db=gene&term=LNX1) | ligand of numb-protein X 1 | 0.0002337 | 0.0114 | 205.33 | 172.42 | 899.21 | (1, 3), (2, 3) |
| [229459_at](https://www.affymetrix.com/LinkServlet?probeset=229459_at) | [FAM19A5](http://www.ncbi.nlm.nih.gov/entrez/query.fcgi?cmd=search&db=gene&term=FAM19A5) | family with sequence similarity 19 (chemokine (C-C motif)-like), member A5 | 0.0002351 | 0.0114 | 871.78 | 389.85 | 1016.45 | (2, 1), (2, 3) |
| [242532_at](https://www.affymetrix.com/LinkServlet?probeset=242532_at) | [NA](http://www.ncbi.nlm.nih.gov/entrez/query.fcgi?cmd=search&db=gene&term=NA) | NA | 0.0002352 | 0.0114 | 25.35 | 16.47 | 85.73 | (1, 3), (2, 3) |
| [205337_at](https://www.affymetrix.com/LinkServlet?probeset=205337_at) | [DCT](http://www.ncbi.nlm.nih.gov/entrez/query.fcgi?cmd=search&db=gene&term=DCT) | dopachrome tautomerase (dopachrome delta-isomerase, tyrosine-related protein 2) | 0.0002355 | 0.0114 | 61.41 | 12.31 | 12.04 | (2, 1), (3, 1) |
| [230698_at](https://www.affymetrix.com/LinkServlet?probeset=230698_at) | [CALN1](http://www.ncbi.nlm.nih.gov/entrez/query.fcgi?cmd=search&db=gene&term=CALN1) | calneuron 1 | 0.0002394 | 0.0116 | 59.81 | 13.18 | 133.34 | (2, 3) |
| [241392_at](https://www.affymetrix.com/LinkServlet?probeset=241392_at) | [TMEM39A](http://www.ncbi.nlm.nih.gov/entrez/query.fcgi?cmd=search&db=gene&term=TMEM39A) | transmembrane protein 39A | 0.0002397 | 0.0116 | 49.91 | 20.18 | 63.21 | (2, 1), (2, 3) |
| [237839_at](https://www.affymetrix.com/LinkServlet?probeset=237839_at) | [NA](http://www.ncbi.nlm.nih.gov/entrez/query.fcgi?cmd=search&db=gene&term=NA) | NA | 0.0002402 | 0.0116 | 16.97 | 42.22 | 92.32 | (1, 3) |
| [217004_s_at](https://www.affymetrix.com/LinkServlet?probeset=217004_s_at) | [MCF2](http://www.ncbi.nlm.nih.gov/entrez/query.fcgi?cmd=search&db=gene&term=MCF2) | MCF.2 cell line derived transforming sequence | 0.0002405 | 0.0116 | 8.36 | 8.97 | 18.72 | (1, 3), (2, 3) |
| [219191_s_at](https://www.affymetrix.com/LinkServlet?probeset=219191_s_at) | [BIN2](http://www.ncbi.nlm.nih.gov/entrez/query.fcgi?cmd=search&db=gene&term=BIN2) | bridging integrator 2 | 0.0002436 | 0.0117 | 25.43 | 50.98 | 21.82 | (1, 2), (3, 2) |
| [232235_at](https://www.affymetrix.com/LinkServlet?probeset=232235_at) | [DSEL](http://www.ncbi.nlm.nih.gov/entrez/query.fcgi?cmd=search&db=gene&term=DSEL) | dermatan sulfate epimerase-like | 0.0002465 | 0.0118 | 1250.19 | 1088.88 | 1957.76 | (1, 3), (2, 3) |
| [225292_at](https://www.affymetrix.com/LinkServlet?probeset=225292_at) | [COL27A1](http://www.ncbi.nlm.nih.gov/entrez/query.fcgi?cmd=search&db=gene&term=COL27A1) | collagen, type XXVII, alpha 1 | 0.0002469 | 0.0118 | 32.08 | 27.08 | 61.92 | (1, 3), (2, 3) |
| [1556627_at](https://www.affymetrix.com/LinkServlet?probeset=1556627_at) | [DRP2](http://www.ncbi.nlm.nih.gov/entrez/query.fcgi?cmd=search&db=gene&term=DRP2) | dystrophin related protein 2 | 0.0002477 | 0.0118 | 17.64 | 29.65 | 62.36 | (1, 3) |
| [214611_at](https://www.affymetrix.com/LinkServlet?probeset=214611_at) | [GRIK1](http://www.ncbi.nlm.nih.gov/entrez/query.fcgi?cmd=search&db=gene&term=GRIK1) | glutamate receptor, ionotropic, kainate 1 | 0.0002488 | 0.0119 | 19.98 | 36.82 | 59.51 | (1, 3) |
| [204068_at](https://www.affymetrix.com/LinkServlet?probeset=204068_at) | [STK3](http://www.ncbi.nlm.nih.gov/entrez/query.fcgi?cmd=search&db=gene&term=STK3) | serine/threonine kinase 3 (STE20 homolog, yeast) | 0.0002505 | 0.0119 | 313.58 | 429.91 | 257.42 | (3, 2) |
| [236835_at](https://www.affymetrix.com/LinkServlet?probeset=236835_at) | [LOC645431](http://www.ncbi.nlm.nih.gov/entrez/query.fcgi?cmd=search&db=gene&term=LOC645431) | hypothetical LOC645431 | 0.0002506 | 0.0119 | 16.24 | 7.43 | 7.82 | (2, 1), (3, 1) |
| [235182_at](https://www.affymetrix.com/LinkServlet?probeset=235182_at) | [ISM1](http://www.ncbi.nlm.nih.gov/entrez/query.fcgi?cmd=search&db=gene&term=ISM1) | isthmin 1 homolog (zebrafish) | 0.0002522 | 0.0119 | 13.95 | 6.55 | 6.41 | (2, 1), (3, 1) |
| [243163_at](https://www.affymetrix.com/LinkServlet?probeset=243163_at) | [NA](http://www.ncbi.nlm.nih.gov/entrez/query.fcgi?cmd=search&db=gene&term=NA) | NA | 0.0002534 | 0.012 | 39.41 | 72.95 | 177.23 | (1, 3) |
| [213997_at](https://www.affymetrix.com/LinkServlet?probeset=213997_at) | [FAM189A1](http://www.ncbi.nlm.nih.gov/entrez/query.fcgi?cmd=search&db=gene&term=FAM189A1) | family with sequence similarity 189, member A1 | 0.0002547 | 0.012 | 24.38 | 32.24 | 14.85 | (3, 2) |
| [225579_at](https://www.affymetrix.com/LinkServlet?probeset=225579_at) | [PQLC3](http://www.ncbi.nlm.nih.gov/entrez/query.fcgi?cmd=search&db=gene&term=PQLC3) | PQ loop repeat containing 3 | 0.0002582 | 0.0121 | 362.61 | 492.84 | 197.98 | (3, 2) |
| [215134_at](https://www.affymetrix.com/LinkServlet?probeset=215134_at) | [PI4K2A](http://www.ncbi.nlm.nih.gov/entrez/query.fcgi?cmd=search&db=gene&term=PI4K2A) | phosphatidylinositol 4-kinase type 2 alpha | 0.0002583 | 0.0121 | 14.08 | 15.49 | 9.5 | (3, 1), (3, 2) |
| [209389_x_at](https://www.affymetrix.com/LinkServlet?probeset=209389_x_at) | [DBI](http://www.ncbi.nlm.nih.gov/entrez/query.fcgi?cmd=search&db=gene&term=DBI) | diazepam binding inhibitor (GABA receptor modulator, acyl-Coenzyme A binding protein) | 0.0002602 | 0.0121 | 5459.33 | 2964.7 | 5300.89 | (2, 1), (2, 3) |
| [223843_at](https://www.affymetrix.com/LinkServlet?probeset=223843_at) | [SCARA3](http://www.ncbi.nlm.nih.gov/entrez/query.fcgi?cmd=search&db=gene&term=SCARA3) | scavenger receptor class A, member 3 | 0.0002604 | 0.0121 | 65.67 | 88.45 | 221.79 | (1, 3), (2, 3) |
| [209496_at](https://www.affymetrix.com/LinkServlet?probeset=209496_at) | [RARRES2](http://www.ncbi.nlm.nih.gov/entrez/query.fcgi?cmd=search&db=gene&term=RARRES2) | retinoic acid receptor responder (tazarotene induced) 2 | 0.0002605 | 0.0121 | 312.62 | 1632.44 | 1101.75 | (1, 2), (1, 3) |
| [229437_at](https://www.affymetrix.com/LinkServlet?probeset=229437_at) | [MIR155HG](http://www.ncbi.nlm.nih.gov/entrez/query.fcgi?cmd=search&db=gene&term=MIR155HG) | MIR155 host gene (non-protein coding) | 0.0002621 | 0.0122 | 11.46 | 31.25 | 7.33 | (3, 2) |
| [205524_s_at](https://www.affymetrix.com/LinkServlet?probeset=205524_s_at) | [HAPLN1](http://www.ncbi.nlm.nih.gov/entrez/query.fcgi?cmd=search&db=gene&term=HAPLN1) | hyaluronan and proteoglycan link protein 1 | 0.0002633 | 0.0122 | 45.47 | 19.8 | 12.07 | (3, 1) |
| [1554863_s_at](https://www.affymetrix.com/LinkServlet?probeset=1554863_s_at) | [DOK5](http://www.ncbi.nlm.nih.gov/entrez/query.fcgi?cmd=search&db=gene&term=DOK5) | docking protein 5 | 0.0002633 | 0.0122 | 70.62 | 176.38 | 52.31 | (1, 2), (3, 2) |
| [224325_at](https://www.affymetrix.com/LinkServlet?probeset=224325_at) | [FZD8](http://www.ncbi.nlm.nih.gov/entrez/query.fcgi?cmd=search&db=gene&term=FZD8) | frizzled homolog 8 (Drosophila) | 0.0002637 | 0.0122 | 156.09 | 158.48 | 48.75 | (3, 1), (3, 2) |
| [205051_s_at](https://www.affymetrix.com/LinkServlet?probeset=205051_s_at) | [KIT](http://www.ncbi.nlm.nih.gov/entrez/query.fcgi?cmd=search&db=gene&term=KIT) | v-kit Hardy-Zuckerman 4 feline sarcoma viral oncogene homolog | 0.0002656 | 0.0122 | 71.25 | 52.82 | 217.11 | (1, 3), (2, 3) |
| [203408_s_at](https://www.affymetrix.com/LinkServlet?probeset=203408_s_at) | [SATB1](http://www.ncbi.nlm.nih.gov/entrez/query.fcgi?cmd=search&db=gene&term=SATB1) | SATB homeobox 1 | 0.0002658 | 0.0122 | 1143.69 | 991.12 | 2247.3 | (1, 3), (2, 3) |
| [216095_x_at](https://www.affymetrix.com/LinkServlet?probeset=216095_x_at) | [MTMR1](http://www.ncbi.nlm.nih.gov/entrez/query.fcgi?cmd=search&db=gene&term=MTMR1) | myotubularin related protein 1 | 0.000266 | 0.0122 | 448.42 | 550.65 | 729 | (1, 3) |
| [230923_at](https://www.affymetrix.com/LinkServlet?probeset=230923_at) | [FAM19A1](http://www.ncbi.nlm.nih.gov/entrez/query.fcgi?cmd=search&db=gene&term=FAM19A1) | family with sequence similarity 19 (chemokine (C-C motif)-like), member A1 | 0.0002675 | 0.0122 | 22.43 | 5.77 | 31.59 | (2, 1), (2, 3) |
| [239682_at](https://www.affymetrix.com/LinkServlet?probeset=239682_at) | [NA](http://www.ncbi.nlm.nih.gov/entrez/query.fcgi?cmd=search&db=gene&term=NA) | NA | 0.0002676 | 0.0122 | 97.93 | 188.54 | 209.17 | (1, 2), (1, 3) |
| [234317_s_at](https://www.affymetrix.com/LinkServlet?probeset=234317_s_at) | [STOX2](http://www.ncbi.nlm.nih.gov/entrez/query.fcgi?cmd=search&db=gene&term=STOX2) | storkhead box 2 | 0.0002677 | 0.0122 | 157.52 | 86.91 | 174.89 | (2, 1), (2, 3) |
| [213429_at](https://www.affymetrix.com/LinkServlet?probeset=213429_at) | [NA](http://www.ncbi.nlm.nih.gov/entrez/query.fcgi?cmd=search&db=gene&term=NA) | NA | 0.0002687 | 0.0122 | 41.42 | 114.55 | 26.87 | (3, 2) |
| [213417_at](https://www.affymetrix.com/LinkServlet?probeset=213417_at) | [TBX2](http://www.ncbi.nlm.nih.gov/entrez/query.fcgi?cmd=search&db=gene&term=TBX2) | T-box 2 | 0.0002725 | 0.0123 | 14.84 | 6.25 | 5.82 | (2, 1), (3, 1) |
| [222008_at](https://www.affymetrix.com/LinkServlet?probeset=222008_at) | [COL9A1](http://www.ncbi.nlm.nih.gov/entrez/query.fcgi?cmd=search&db=gene&term=COL9A1) | collagen, type IX, alpha 1 | 0.0002731 | 0.0123 | 52.42 | 180.9 | 309.2 | (1, 3) |
| [204057_at](https://www.affymetrix.com/LinkServlet?probeset=204057_at) | [IRF8](http://www.ncbi.nlm.nih.gov/entrez/query.fcgi?cmd=search&db=gene&term=IRF8) | interferon regulatory factor 8 | 0.0002733 | 0.0123 | 239.02 | 420.71 | 146.31 | (3, 2) |
| [208651_x_at](https://www.affymetrix.com/LinkServlet?probeset=208651_x_at) | [CD24](http://www.ncbi.nlm.nih.gov/entrez/query.fcgi?cmd=search&db=gene&term=CD24) | CD24 molecule | 0.0002739 | 0.0123 | 50.53 | 11.59 | 9 | (2, 1), (3, 1) |
| [238344_at](https://www.affymetrix.com/LinkServlet?probeset=238344_at) | [NA](http://www.ncbi.nlm.nih.gov/entrez/query.fcgi?cmd=search&db=gene&term=NA) | NA | 0.0002744 | 0.0123 | 53.02 | 51.19 | 175.34 | (1, 3), (2, 3) |
| [204933_s_at](https://www.affymetrix.com/LinkServlet?probeset=204933_s_at) | [TNFRSF11B](http://www.ncbi.nlm.nih.gov/entrez/query.fcgi?cmd=search&db=gene&term=TNFRSF11B) | tumor necrosis factor receptor superfamily, member 11b | 0.0002745 | 0.0123 | 14.93 | 53.23 | 66.01 | (1, 2), (1, 3) |
| [231856_at](https://www.affymetrix.com/LinkServlet?probeset=231856_at) | [KIAA1244](http://www.ncbi.nlm.nih.gov/entrez/query.fcgi?cmd=search&db=gene&term=KIAA1244) | KIAA1244 | 0.0002766 | 0.0124 | 101.23 | 71.19 | 174.65 | (2, 3) |
| [212448_at](https://www.affymetrix.com/LinkServlet?probeset=212448_at) | [NEDD4L](http://www.ncbi.nlm.nih.gov/entrez/query.fcgi?cmd=search&db=gene&term=NEDD4L) | neural precursor cell expressed, developmentally down-regulated 4-like | 0.0002779 | 0.0125 | 147.3 | 23.03 | 30.45 | (2, 1), (3, 1) |
| [242228_at](https://www.affymetrix.com/LinkServlet?probeset=242228_at) | [NA](http://www.ncbi.nlm.nih.gov/entrez/query.fcgi?cmd=search&db=gene&term=NA) | NA | 0.0002815 | 0.0126 | 98.64 | 78.69 | 48.66 | (3, 1), (3, 2) |
| [230362_at](https://www.affymetrix.com/LinkServlet?probeset=230362_at) | [INPP5F](http://www.ncbi.nlm.nih.gov/entrez/query.fcgi?cmd=search&db=gene&term=INPP5F) | inositol polyphosphate-5-phosphatase F | 0.0002833 | 0.0126 | 37.9 | 55.9 | 22.29 | (3, 2) |
| [1554474_a_at](https://www.affymetrix.com/LinkServlet?probeset=1554474_a_at) | [MOXD1](http://www.ncbi.nlm.nih.gov/entrez/query.fcgi?cmd=search&db=gene&term=MOXD1) | monooxygenase, DBH-like 1 | 0.0002862 | 0.0127 | 138.94 | 24.99 | 18.7 | (2, 1), (3, 1) |
| [221636_s_at](https://www.affymetrix.com/LinkServlet?probeset=221636_s_at) | [MOSC2](http://www.ncbi.nlm.nih.gov/entrez/query.fcgi?cmd=search&db=gene&term=MOSC2) | MOCO sulphurase C-terminal domain containing 2 | 0.0002893 | 0.0129 | 52.5 | 28.55 | 50.78 | (2, 1), (2, 3) |
| [206342_x_at](https://www.affymetrix.com/LinkServlet?probeset=206342_x_at) | [IDS](http://www.ncbi.nlm.nih.gov/entrez/query.fcgi?cmd=search&db=gene&term=IDS) | iduronate 2-sulfatase | 0.0002912 | 0.0129 | 122.82 | 117.75 | 69.8 | (3, 1), (3, 2) |
| [222609_s_at](https://www.affymetrix.com/LinkServlet?probeset=222609_s_at) | [EXOSC1](http://www.ncbi.nlm.nih.gov/entrez/query.fcgi?cmd=search&db=gene&term=EXOSC1) | exosome component 1 | 0.0002913 | 0.0129 | 301.72 | 338.24 | 215.07 | (3, 1), (3, 2) |
| [219926_at](https://www.affymetrix.com/LinkServlet?probeset=219926_at) | [POPDC3](http://www.ncbi.nlm.nih.gov/entrez/query.fcgi?cmd=search&db=gene&term=POPDC3) | popeye domain containing 3 | 0.0002949 | 0.013 | 55.36 | 39.51 | 9.91 | (3, 1), (3, 2) |
| [223179_at](https://www.affymetrix.com/LinkServlet?probeset=223179_at) | [YPEL3](http://www.ncbi.nlm.nih.gov/entrez/query.fcgi?cmd=search&db=gene&term=YPEL3) | yippee-like 3 (Drosophila) | 0.0002952 | 0.013 | 449.59 | 364.65 | 637.84 | (2, 3) |
| [232412_at](https://www.affymetrix.com/LinkServlet?probeset=232412_at) | [FBXL20](http://www.ncbi.nlm.nih.gov/entrez/query.fcgi?cmd=search&db=gene&term=FBXL20) | F-box and leucine-rich repeat protein 20 | 0.0002998 | 0.0132 | 42.11 | 21.2 | 32.51 | (2, 1), (2, 3) |
| [205466_s_at](https://www.affymetrix.com/LinkServlet?probeset=205466_s_at) | [HS3ST1](http://www.ncbi.nlm.nih.gov/entrez/query.fcgi?cmd=search&db=gene&term=HS3ST1) | heparan sulfate (glucosamine) 3-O-sulfotransferase 1 | 0.0003036 | 0.0133 | 363.49 | 793.06 | 962.57 | (1, 2), (1, 3) |
| [206089_at](https://www.affymetrix.com/LinkServlet?probeset=206089_at) | [NELL1](http://www.ncbi.nlm.nih.gov/entrez/query.fcgi?cmd=search&db=gene&term=NELL1) | NEL-like 1 (chicken) | 0.0003045 | 0.0133 | 35.62 | 6.33 | 7.09 | (2, 1), (3, 1) |
| [227892_at](https://www.affymetrix.com/LinkServlet?probeset=227892_at) | [PRKAA2](http://www.ncbi.nlm.nih.gov/entrez/query.fcgi?cmd=search&db=gene&term=PRKAA2) | protein kinase, AMP-activated, alpha 2 catalytic subunit | 0.0003046 | 0.0133 | 61.5 | 117.62 | 189.36 | (1, 3) |
| [227812_at](https://www.affymetrix.com/LinkServlet?probeset=227812_at) | [TNFRSF19](http://www.ncbi.nlm.nih.gov/entrez/query.fcgi?cmd=search&db=gene&term=TNFRSF19) | tumor necrosis factor receptor superfamily, member 19 | 0.0003048 | 0.0133 | 743.27 | 192.05 | 332.8 | (2, 1), (3, 1) |
| [213351_s_at](https://www.affymetrix.com/LinkServlet?probeset=213351_s_at) | [TMCC1](http://www.ncbi.nlm.nih.gov/entrez/query.fcgi?cmd=search&db=gene&term=TMCC1) | transmembrane and coiled-coil domain family 1 | 0.0003134 | 0.0136 | 456.57 | 307.14 | 637.98 | (2, 3) |
| [230363_s_at](https://www.affymetrix.com/LinkServlet?probeset=230363_s_at) | [INPP5F](http://www.ncbi.nlm.nih.gov/entrez/query.fcgi?cmd=search&db=gene&term=INPP5F) | inositol polyphosphate-5-phosphatase F | 0.0003139 | 0.0136 | 120.95 | 154.88 | 71.36 | (3, 2) |
| [228821_at](https://www.affymetrix.com/LinkServlet?probeset=228821_at) | [ST6GAL2](http://www.ncbi.nlm.nih.gov/entrez/query.fcgi?cmd=search&db=gene&term=ST6GAL2) | ST6 beta-galactosamide alpha-2,6-sialyltranferase 2 | 0.0003151 | 0.0137 | 22.33 | 19.92 | 115.09 | (1, 3), (2, 3) |
| [202781_s_at](https://www.affymetrix.com/LinkServlet?probeset=202781_s_at) | [INPP5K](http://www.ncbi.nlm.nih.gov/entrez/query.fcgi?cmd=search&db=gene&term=INPP5K) | inositol polyphosphate-5-phosphatase K | 0.0003161 | 0.0137 | 28.6 | 29.53 | 19.92 | (3, 1), (3, 2) |
| [238583_at](https://www.affymetrix.com/LinkServlet?probeset=238583_at) | [MSRB3](http://www.ncbi.nlm.nih.gov/entrez/query.fcgi?cmd=search&db=gene&term=MSRB3) | methionine sulfoxide reductase B3 | 0.0003163 | 0.0137 | 15.59 | 28.48 | 25.69 | (1, 2), (1, 3) |
| [64942_at](https://www.affymetrix.com/LinkServlet?probeset=64942_at) | [GPR153](http://www.ncbi.nlm.nih.gov/entrez/query.fcgi?cmd=search&db=gene&term=GPR153) | G protein-coupled receptor 153 | 0.0003178 | 0.0137 | 117.27 | 37.96 | 95.65 | (2, 1), (2, 3) |
| [230068_s_at](https://www.affymetrix.com/LinkServlet?probeset=230068_s_at) | [PEG3](http://www.ncbi.nlm.nih.gov/entrez/query.fcgi?cmd=search&db=gene&term=PEG3) | paternally expressed 3 | 0.0003182 | 0.0137 | 59.33 | 53.36 | 118.56 | (1, 3), (2, 3) |
| [210068_s_at](https://www.affymetrix.com/LinkServlet?probeset=210068_s_at) | [AQP4](http://www.ncbi.nlm.nih.gov/entrez/query.fcgi?cmd=search&db=gene&term=AQP4) | aquaporin 4 | 0.0003191 | 0.0137 | 79.05 | 328.34 | 559.28 | (1, 2), (1, 3) |
| [241404_at](https://www.affymetrix.com/LinkServlet?probeset=241404_at) | [NA](http://www.ncbi.nlm.nih.gov/entrez/query.fcgi?cmd=search&db=gene&term=NA) | NA | 0.0003204 | 0.0137 | 12.02 | 8.79 | 26.67 | (1, 3), (2, 3) |
| [209392_at](https://www.affymetrix.com/LinkServlet?probeset=209392_at) | [ENPP2](http://www.ncbi.nlm.nih.gov/entrez/query.fcgi?cmd=search&db=gene&term=ENPP2) | ectonucleotide pyrophosphatase/phosphodiesterase 2 | 0.0003236 | 0.0138 | 1827.41 | 178.31 | 501.11 | (2, 1), (3, 1) |
| [219440_at](https://www.affymetrix.com/LinkServlet?probeset=219440_at) | [RAI2](http://www.ncbi.nlm.nih.gov/entrez/query.fcgi?cmd=search&db=gene&term=RAI2) | retinoic acid induced 2 | 0.0003249 | 0.0139 | 88.75 | 101.21 | 199.26 | (1, 3), (2, 3) |
| [226682_at](https://www.affymetrix.com/LinkServlet?probeset=226682_at) | [RORA](http://www.ncbi.nlm.nih.gov/entrez/query.fcgi?cmd=search&db=gene&term=RORA) | RAR-related orphan receptor A | 0.0003262 | 0.0139 | 506.89 | 556.05 | 1112.55 | (1, 3), (2, 3) |
| [224831_at](https://www.affymetrix.com/LinkServlet?probeset=224831_at) | [CPEB4](http://www.ncbi.nlm.nih.gov/entrez/query.fcgi?cmd=search&db=gene&term=CPEB4) | cytoplasmic polyadenylation element binding protein 4 | 0.0003287 | 0.014 | 1065.71 | 1076.76 | 1729.4 | (1, 3), (2, 3) |
| [203736_s_at](https://www.affymetrix.com/LinkServlet?probeset=203736_s_at) | [PPFIBP1](http://www.ncbi.nlm.nih.gov/entrez/query.fcgi?cmd=search&db=gene&term=PPFIBP1) | PTPRF interacting protein, binding protein 1 (liprin beta 1) | 0.0003303 | 0.014 | 14.93 | 20.48 | 11.23 | (3, 2) |
| [225016_at](https://www.affymetrix.com/LinkServlet?probeset=225016_at) | [APCDD1](http://www.ncbi.nlm.nih.gov/entrez/query.fcgi?cmd=search&db=gene&term=APCDD1) | adenomatosis polyposis coli down-regulated 1 | 0.0003318 | 0.0141 | 571.68 | 277.47 | 833.54 | (2, 3) |
| [228151_at](https://www.affymetrix.com/LinkServlet?probeset=228151_at) | [NA](http://www.ncbi.nlm.nih.gov/entrez/query.fcgi?cmd=search&db=gene&term=NA) | NA | 0.0003339 | 0.0141 | 677.67 | 530.39 | 411.41 | (3, 1) |
| [219947_at](https://www.affymetrix.com/LinkServlet?probeset=219947_at) | [CLEC4A](http://www.ncbi.nlm.nih.gov/entrez/query.fcgi?cmd=search&db=gene&term=CLEC4A) | C-type lectin domain family 4, member A | 0.0003365 | 0.0142 | 84.04 | 150.38 | 49.36 | (3, 2) |
| [238133_at](https://www.affymetrix.com/LinkServlet?probeset=238133_at) | [NA](http://www.ncbi.nlm.nih.gov/entrez/query.fcgi?cmd=search&db=gene&term=NA) | NA | 0.0003395 | 0.0143 | 109.5 | 178.72 | 460.18 | (1, 3), (2, 3) |
| [235419_at](https://www.affymetrix.com/LinkServlet?probeset=235419_at) | [NA](http://www.ncbi.nlm.nih.gov/entrez/query.fcgi?cmd=search&db=gene&term=NA) | NA | 0.0003428 | 0.0144 | 17.32 | 13.66 | 41.31 | (1, 3), (2, 3) |
| [204368_at](https://www.affymetrix.com/LinkServlet?probeset=204368_at) | [SLCO2A1](http://www.ncbi.nlm.nih.gov/entrez/query.fcgi?cmd=search&db=gene&term=SLCO2A1) | solute carrier organic anion transporter family, member 2A1 | 0.0003429 | 0.0144 | 48.59 | 32.27 | 19.43 | (3, 1) |
| [225293_at](https://www.affymetrix.com/LinkServlet?probeset=225293_at) | [COL27A1](http://www.ncbi.nlm.nih.gov/entrez/query.fcgi?cmd=search&db=gene&term=COL27A1) | collagen, type XXVII, alpha 1 | 0.0003461 | 0.0145 | 344.94 | 254.41 | 725.52 | (2, 3) |
| [235800_at](https://www.affymetrix.com/LinkServlet?probeset=235800_at) | [NA](http://www.ncbi.nlm.nih.gov/entrez/query.fcgi?cmd=search&db=gene&term=NA) | NA | 0.0003472 | 0.0145 | 37.71 | 51.2 | 23.76 | (3, 2) |
| [227534_at](https://www.affymetrix.com/LinkServlet?probeset=227534_at) | [C9orf21](http://www.ncbi.nlm.nih.gov/entrez/query.fcgi?cmd=search&db=gene&term=C9orf21) | chromosome 9 open reading frame 21 | 0.0003475 | 0.0145 | 189.52 | 321.88 | 161.39 | (3, 2) |
| [1566638_at](https://www.affymetrix.com/LinkServlet?probeset=1566638_at) | [NA](http://www.ncbi.nlm.nih.gov/entrez/query.fcgi?cmd=search&db=gene&term=NA) | NA | 0.000348 | 0.0145 | 14.79 | 15.66 | 70.55 | (1, 3), (2, 3) |
| [220311_at](https://www.affymetrix.com/LinkServlet?probeset=220311_at) | [N6AMT1](http://www.ncbi.nlm.nih.gov/entrez/query.fcgi?cmd=search&db=gene&term=N6AMT1) | N-6 adenine-specific DNA methyltransferase 1 (putative) | 0.0003481 | 0.0145 | 37.83 | 34.31 | 18.63 | (3, 1), (3, 2) |
| [225116_at](https://www.affymetrix.com/LinkServlet?probeset=225116_at) | [HIPK2](http://www.ncbi.nlm.nih.gov/entrez/query.fcgi?cmd=search&db=gene&term=HIPK2) | homeodomain interacting protein kinase 2 | 0.0003486 | 0.0145 | 1392.67 | 568.44 | 1453.5 | (2, 1), (2, 3) |
| [216942_s_at](https://www.affymetrix.com/LinkServlet?probeset=216942_s_at) | [CD58](http://www.ncbi.nlm.nih.gov/entrez/query.fcgi?cmd=search&db=gene&term=CD58) | CD58 molecule | 0.0003491 | 0.0145 | 258.17 | 242.67 | 86.02 | (3, 1), (3, 2) |
| [238669_at](https://www.affymetrix.com/LinkServlet?probeset=238669_at) | [PTGS1](http://www.ncbi.nlm.nih.gov/entrez/query.fcgi?cmd=search&db=gene&term=PTGS1) | prostaglandin-endoperoxide synthase 1 (prostaglandin G/H synthase and cyclooxygenase) | 0.0003507 | 0.0145 | 106.13 | 105.67 | 60.18 | (3, 1), (3, 2) |
| [211744_s_at](https://www.affymetrix.com/LinkServlet?probeset=211744_s_at) | [CD58](http://www.ncbi.nlm.nih.gov/entrez/query.fcgi?cmd=search&db=gene&term=CD58) | CD58 molecule | 0.000353 | 0.0146 | 308.28 | 279.26 | 95.25 | (3, 1), (3, 2) |
| [206622_at](https://www.affymetrix.com/LinkServlet?probeset=206622_at) | [TRH](http://www.ncbi.nlm.nih.gov/entrez/query.fcgi?cmd=search&db=gene&term=TRH) | thyrotropin-releasing hormone | 0.0003538 | 0.0146 | 7.71 | 12.47 | 56.65 | (1, 3), (2, 3) |
| [227417_at](https://www.affymetrix.com/LinkServlet?probeset=227417_at) | [MOSC2](http://www.ncbi.nlm.nih.gov/entrez/query.fcgi?cmd=search&db=gene&term=MOSC2) | MOCO sulphurase C-terminal domain containing 2 | 0.0003557 | 0.0146 | 54.59 | 25.31 | 69.49 | (2, 3) |
| [243929_at](https://www.affymetrix.com/LinkServlet?probeset=243929_at) | [NA](http://www.ncbi.nlm.nih.gov/entrez/query.fcgi?cmd=search&db=gene&term=NA) | NA | 0.0003565 | 0.0146 | 5.43 | 4.87 | 25.64 | (1, 3), (2, 3) |
| [223398_at](https://www.affymetrix.com/LinkServlet?probeset=223398_at) | [C9orf89](http://www.ncbi.nlm.nih.gov/entrez/query.fcgi?cmd=search&db=gene&term=C9orf89) | chromosome 9 open reading frame 89 | 0.0003571 | 0.0146 | 170.69 | 215.2 | 128.81 | (3, 2) |
| [35974_at](https://www.affymetrix.com/LinkServlet?probeset=35974_at) | [LRMP](http://www.ncbi.nlm.nih.gov/entrez/query.fcgi?cmd=search&db=gene&term=LRMP) | lymphoid-restricted membrane protein | 0.0003585 | 0.0147 | 34.83 | 60.8 | 23.99 | (3, 2) |
| [205338_s_at](https://www.affymetrix.com/LinkServlet?probeset=205338_s_at) | [DCT](http://www.ncbi.nlm.nih.gov/entrez/query.fcgi?cmd=search&db=gene&term=DCT) | dopachrome tautomerase (dopachrome delta-isomerase, tyrosine-related protein 2) | 0.0003651 | 0.0149 | 26.43 | 6.34 | 7.25 | (2, 1), (3, 1) |
| [210066_s_at](https://www.affymetrix.com/LinkServlet?probeset=210066_s_at) | [AQP4](http://www.ncbi.nlm.nih.gov/entrez/query.fcgi?cmd=search&db=gene&term=AQP4) | aquaporin 4 | 0.0003676 | 0.015 | 33.73 | 139.07 | 229.94 | (1, 2), (1, 3) |
| [206302_s_at](https://www.affymetrix.com/LinkServlet?probeset=206302_s_at) | [NA](http://www.ncbi.nlm.nih.gov/entrez/query.fcgi?cmd=search&db=gene&term=NA) | NA | 0.0003686 | 0.015 | 571.83 | 1203.61 | 499.67 | (1, 2), (3, 2) |
| [240138_at](https://www.affymetrix.com/LinkServlet?probeset=240138_at) | [NA](http://www.ncbi.nlm.nih.gov/entrez/query.fcgi?cmd=search&db=gene&term=NA) | NA | 0.0003697 | 0.015 | 57.98 | 98.86 | 317.34 | (1, 3), (2, 3) |
| [206584_at](https://www.affymetrix.com/LinkServlet?probeset=206584_at) | [LY96](http://www.ncbi.nlm.nih.gov/entrez/query.fcgi?cmd=search&db=gene&term=LY96) | lymphocyte antigen 96 | 0.0003706 | 0.015 | 707.09 | 941.6 | 350.94 | (3, 2) |
| [209665_at](https://www.affymetrix.com/LinkServlet?probeset=209665_at) | [CYB561D2](http://www.ncbi.nlm.nih.gov/entrez/query.fcgi?cmd=search&db=gene&term=CYB561D2) | cytochrome b-561 domain containing 2 | 0.0003708 | 0.015 | 115.1 | 114.1 | 73.21 | (3, 1), (3, 2) |
| [238076_at](https://www.affymetrix.com/LinkServlet?probeset=238076_at) | [GATAD2B](http://www.ncbi.nlm.nih.gov/entrez/query.fcgi?cmd=search&db=gene&term=GATAD2B) | GATA zinc finger domain containing 2B | 0.0003708 | 0.015 | 523.86 | 375.77 | 638.36 | (2, 3) |
| [222871_at](https://www.affymetrix.com/LinkServlet?probeset=222871_at) | [KLHDC8A](http://www.ncbi.nlm.nih.gov/entrez/query.fcgi?cmd=search&db=gene&term=KLHDC8A) | kelch domain containing 8A | 0.000372 | 0.015 | 396.65 | 336.59 | 141.94 | (3, 1), (3, 2) |
| [207057_at](https://www.affymetrix.com/LinkServlet?probeset=207057_at) | [SLC16A7](http://www.ncbi.nlm.nih.gov/entrez/query.fcgi?cmd=search&db=gene&term=SLC16A7) | solute carrier family 16, member 7 (monocarboxylic acid transporter 2) | 0.0003784 | 0.0152 | 131.19 | 277.12 | 378.27 | (1, 3) |
| [205905_s_at](https://www.affymetrix.com/LinkServlet?probeset=205905_s_at) | [NA](http://www.ncbi.nlm.nih.gov/entrez/query.fcgi?cmd=search&db=gene&term=NA) | NA | 0.0003801 | 0.0152 | 22.06 | 19.15 | 12.05 | (3, 1), (3, 2) |
| [1555573_at](https://www.affymetrix.com/LinkServlet?probeset=1555573_at) | [C10orf93](http://www.ncbi.nlm.nih.gov/entrez/query.fcgi?cmd=search&db=gene&term=C10orf93) | chromosome 10 open reading frame 93 | 0.0003801 | 0.0152 | 19.53 | 36.84 | 16.44 | (1, 2), (3, 2) |
| [215789_s_at](https://www.affymetrix.com/LinkServlet?probeset=215789_s_at) | [AJAP1](http://www.ncbi.nlm.nih.gov/entrez/query.fcgi?cmd=search&db=gene&term=AJAP1) | adherens junctions associated protein 1 | 0.0003852 | 0.0154 | 11.89 | 41.78 | 54.87 | (1, 2), (1, 3) |
| [226695_at](https://www.affymetrix.com/LinkServlet?probeset=226695_at) | [PRRX1](http://www.ncbi.nlm.nih.gov/entrez/query.fcgi?cmd=search&db=gene&term=PRRX1) | paired related homeobox 1 | 0.000386 | 0.0154 | 947.81 | 2290.01 | 1683.16 | (1, 2), (1, 3) |
| [223087_at](https://www.affymetrix.com/LinkServlet?probeset=223087_at) | [ECHDC1](http://www.ncbi.nlm.nih.gov/entrez/query.fcgi?cmd=search&db=gene&term=ECHDC1) | enoyl Coenzyme A hydratase domain containing 1 | 0.0003869 | 0.0154 | 2372.37 | 2823.25 | 1820.16 | (3, 2) |
| [224997_x_at](https://www.affymetrix.com/LinkServlet?probeset=224997_x_at) | [H19](http://www.ncbi.nlm.nih.gov/entrez/query.fcgi?cmd=search&db=gene&term=H19) | H19, imprinted maternally expressed transcript (non-protein coding) | 0.0003874 | 0.0154 | 6.53 | 6.41 | 19.92 | (1, 3), (2, 3) |
| [231252_at](https://www.affymetrix.com/LinkServlet?probeset=231252_at) | [C2orf67](http://www.ncbi.nlm.nih.gov/entrez/query.fcgi?cmd=search&db=gene&term=C2orf67) | chromosome 2 open reading frame 67 | 0.0003899 | 0.0155 | 38.26 | 56.16 | 93.87 | (1, 3) |
| [223612_s_at](https://www.affymetrix.com/LinkServlet?probeset=223612_s_at) | [LNX1](http://www.ncbi.nlm.nih.gov/entrez/query.fcgi?cmd=search&db=gene&term=LNX1) | ligand of numb-protein X 1 | 0.0003909 | 0.0155 | 30.61 | 24.75 | 73.53 | (1, 3), (2, 3) |
| [236875_at](https://www.affymetrix.com/LinkServlet?probeset=236875_at) | [NA](http://www.ncbi.nlm.nih.gov/entrez/query.fcgi?cmd=search&db=gene&term=NA) | NA | 0.0003921 | 0.0155 | 21.39 | 12.66 | 11.9 | (2, 1), (3, 1) |
| [1558322_a_at](https://www.affymetrix.com/LinkServlet?probeset=1558322_a_at) | [PAQR9](http://www.ncbi.nlm.nih.gov/entrez/query.fcgi?cmd=search&db=gene&term=PAQR9) | progestin and adipoQ receptor family member IX | 0.0003923 | 0.0155 | 10.06 | 5.74 | 5.78 | (2, 1), (3, 1) |
| [212655_at](https://www.affymetrix.com/LinkServlet?probeset=212655_at) | [ZCCHC14](http://www.ncbi.nlm.nih.gov/entrez/query.fcgi?cmd=search&db=gene&term=ZCCHC14) | zinc finger, CCHC domain containing 14 | 0.0003935 | 0.0155 | 444.15 | 306.06 | 514.41 | (2, 3) |
| [203819_s_at](https://www.affymetrix.com/LinkServlet?probeset=203819_s_at) | [NA](http://www.ncbi.nlm.nih.gov/entrez/query.fcgi?cmd=search&db=gene&term=NA) | NA | 0.0003938 | 0.0155 | 34.04 | 220.1 | 28.69 | (1, 2), (3, 2) |
| [204352_at](https://www.affymetrix.com/LinkServlet?probeset=204352_at) | [TRAF5](http://www.ncbi.nlm.nih.gov/entrez/query.fcgi?cmd=search&db=gene&term=TRAF5) | TNF receptor-associated factor 5 | 0.0004001 | 0.0157 | 51.44 | 121.18 | 62.18 | (1, 2), (3, 2) |
| [226728_at](https://www.affymetrix.com/LinkServlet?probeset=226728_at) | [SLC27A1](http://www.ncbi.nlm.nih.gov/entrez/query.fcgi?cmd=search&db=gene&term=SLC27A1) | solute carrier family 27 (fatty acid transporter), member 1 | 0.000402 | 0.0157 | 324.84 | 150.57 | 336.57 | (2, 1), (2, 3) |
| [209049_s_at](https://www.affymetrix.com/LinkServlet?probeset=209049_s_at) | [ZMYND8](http://www.ncbi.nlm.nih.gov/entrez/query.fcgi?cmd=search&db=gene&term=ZMYND8) | zinc finger, MYND-type containing 8 | 0.0004023 | 0.0157 | 489.5 | 375.13 | 604.01 | (2, 3) |
| [205206_at](https://www.affymetrix.com/LinkServlet?probeset=205206_at) | [KAL1](http://www.ncbi.nlm.nih.gov/entrez/query.fcgi?cmd=search&db=gene&term=KAL1) | Kallmann syndrome 1 sequence | 0.0004029 | 0.0157 | 337.73 | 1353.9 | 1593.26 | (1, 2), (1, 3) |
| [201234_at](https://www.affymetrix.com/LinkServlet?probeset=201234_at) | [ILK](http://www.ncbi.nlm.nih.gov/entrez/query.fcgi?cmd=search&db=gene&term=ILK) | integrin-linked kinase | 0.0004057 | 0.0158 | 217.76 | 192.14 | 129.29 | (3, 1), (3, 2) |
| [231862_at](https://www.affymetrix.com/LinkServlet?probeset=231862_at) | [CBX5](http://www.ncbi.nlm.nih.gov/entrez/query.fcgi?cmd=search&db=gene&term=CBX5) | chromobox homolog 5 (HP1 alpha homolog, Drosophila) | 0.0004077 | 0.0158 | 126.49 | 83.27 | 139.15 | (2, 1), (2, 3) |
| [226192_at](https://www.affymetrix.com/LinkServlet?probeset=226192_at) | [NA](http://www.ncbi.nlm.nih.gov/entrez/query.fcgi?cmd=search&db=gene&term=NA) | NA | 0.0004078 | 0.0158 | 67.51 | 277.28 | 196.29 | (1, 2), (1, 3) |
| [222957_at](https://www.affymetrix.com/LinkServlet?probeset=222957_at) | [NEU4](http://www.ncbi.nlm.nih.gov/entrez/query.fcgi?cmd=search&db=gene&term=NEU4) | sialidase 4 | 0.0004099 | 0.0159 | 67.41 | 76.39 | 218.21 | (1, 3), (2, 3) |
| [231969_at](https://www.affymetrix.com/LinkServlet?probeset=231969_at) | [STOX2](http://www.ncbi.nlm.nih.gov/entrez/query.fcgi?cmd=search&db=gene&term=STOX2) | storkhead box 2 | 0.0004119 | 0.0159 | 630.66 | 361.9 | 757.76 | (2, 3) |
| [204303_s_at](https://www.affymetrix.com/LinkServlet?probeset=204303_s_at) | [KIAA0427](http://www.ncbi.nlm.nih.gov/entrez/query.fcgi?cmd=search&db=gene&term=KIAA0427) | KIAA0427 | 0.0004158 | 0.0161 | 58.38 | 48.64 | 90.72 | (2, 3) |
| [229973_at](https://www.affymetrix.com/LinkServlet?probeset=229973_at) | [C1orf173](http://www.ncbi.nlm.nih.gov/entrez/query.fcgi?cmd=search&db=gene&term=C1orf173) | chromosome 1 open reading frame 173 | 0.0004184 | 0.0161 | 29.59 | 117.1 | 15.99 | (3, 2) |
| [1552263_at](https://www.affymetrix.com/LinkServlet?probeset=1552263_at) | [MAPK1](http://www.ncbi.nlm.nih.gov/entrez/query.fcgi?cmd=search&db=gene&term=MAPK1) | mitogen-activated protein kinase 1 | 0.0004206 | 0.0161 | 38.39 | 51.96 | 25.77 | (3, 2) |
| [236045_x_at](https://www.affymetrix.com/LinkServlet?probeset=236045_x_at) | [NA](http://www.ncbi.nlm.nih.gov/entrez/query.fcgi?cmd=search&db=gene&term=NA) | NA | 0.0004214 | 0.0161 | 24.48 | 30.46 | 103.05 | (1, 3), (2, 3) |
| [219689_at](https://www.affymetrix.com/LinkServlet?probeset=219689_at) | [SEMA3G](http://www.ncbi.nlm.nih.gov/entrez/query.fcgi?cmd=search&db=gene&term=SEMA3G) | sema domain, immunoglobulin domain (Ig), short basic domain, secreted, (semaphorin) 3G | 0.0004215 | 0.0161 | 33.46 | 9.09 | 9.06 | (2, 1), (3, 1) |
| [218935_at](https://www.affymetrix.com/LinkServlet?probeset=218935_at) | [EHD3](http://www.ncbi.nlm.nih.gov/entrez/query.fcgi?cmd=search&db=gene&term=EHD3) | EH-domain containing 3 | 0.0004216 | 0.0161 | 400.15 | 493.27 | 1087.07 | (1, 3), (2, 3) |
| [239657_x_at](https://www.affymetrix.com/LinkServlet?probeset=239657_x_at) | [FOXO6](http://www.ncbi.nlm.nih.gov/entrez/query.fcgi?cmd=search&db=gene&term=FOXO6) | forkhead box O6 | 0.0004248 | 0.0162 | 29.44 | 18.89 | 15.62 | (3, 1) |
| [201939_at](https://www.affymetrix.com/LinkServlet?probeset=201939_at) | [PLK2](http://www.ncbi.nlm.nih.gov/entrez/query.fcgi?cmd=search&db=gene&term=PLK2) | polo-like kinase 2 (Drosophila) | 0.0004254 | 0.0162 | 543.37 | 391.79 | 207.37 | (3, 1), (3, 2) |
| [213921_at](https://www.affymetrix.com/LinkServlet?probeset=213921_at) | [SST](http://www.ncbi.nlm.nih.gov/entrez/query.fcgi?cmd=search&db=gene&term=SST) | somatostatin | 0.0004254 | 0.0162 | 34.31 | 4.79 | 5.33 | (2, 1), (3, 1) |
| [226322_at](https://www.affymetrix.com/LinkServlet?probeset=226322_at) | [TMTC1](http://www.ncbi.nlm.nih.gov/entrez/query.fcgi?cmd=search&db=gene&term=TMTC1) | transmembrane and tetratricopeptide repeat containing 1 | 0.0004259 | 0.0162 | 132.53 | 509.4 | 116.45 | (1, 2), (3, 2) |
| [220108_at](https://www.affymetrix.com/LinkServlet?probeset=220108_at) | [GNA14](http://www.ncbi.nlm.nih.gov/entrez/query.fcgi?cmd=search&db=gene&term=GNA14) | guanine nucleotide binding protein (G protein), alpha 14 | 0.0004298 | 0.0163 | 41.64 | 68.6 | 16.98 | (3, 2) |
| [233257_at](https://www.affymetrix.com/LinkServlet?probeset=233257_at) | [NA](http://www.ncbi.nlm.nih.gov/entrez/query.fcgi?cmd=search&db=gene&term=NA) | NA | 0.0004322 | 0.0164 | 35.15 | 12.19 | 8.45 | (2, 1), (3, 1) |
| [204712_at](https://www.affymetrix.com/LinkServlet?probeset=204712_at) | [WIF1](http://www.ncbi.nlm.nih.gov/entrez/query.fcgi?cmd=search&db=gene&term=WIF1) | WNT inhibitory factor 1 | 0.0004336 | 0.0164 | 9.61 | 120.59 | 136.92 | (1, 2), (1, 3) |
| [37831_at](https://www.affymetrix.com/LinkServlet?probeset=37831_at) | [SIPA1L3](http://www.ncbi.nlm.nih.gov/entrez/query.fcgi?cmd=search&db=gene&term=SIPA1L3) | signal-induced proliferation-associated 1 like 3 | 0.0004339 | 0.0164 | 27.27 | 24.09 | 37.04 | (2, 3) |
| [207551_s_at](https://www.affymetrix.com/LinkServlet?probeset=207551_s_at) | [MSL3](http://www.ncbi.nlm.nih.gov/entrez/query.fcgi?cmd=search&db=gene&term=MSL3) | male-specific lethal 3 homolog (Drosophila) | 0.0004354 | 0.0164 | 226.09 | 292.85 | 192.43 | (3, 2) |
| [201486_at](https://www.affymetrix.com/LinkServlet?probeset=201486_at) | [RCN2](http://www.ncbi.nlm.nih.gov/entrez/query.fcgi?cmd=search&db=gene&term=RCN2) | reticulocalbin 2, EF-hand calcium binding domain | 0.0004375 | 0.0165 | 2911.19 | 2638.03 | 1637.99 | (3, 1), (3, 2) |
| [210479_s_at](https://www.affymetrix.com/LinkServlet?probeset=210479_s_at) | [RORA](http://www.ncbi.nlm.nih.gov/entrez/query.fcgi?cmd=search&db=gene&term=RORA) | RAR-related orphan receptor A | 0.0004426 | 0.0166 | 39.13 | 39.56 | 123.29 | (1, 3), (2, 3) |
| [221773_at](https://www.affymetrix.com/LinkServlet?probeset=221773_at) | [ELK3](http://www.ncbi.nlm.nih.gov/entrez/query.fcgi?cmd=search&db=gene&term=ELK3) | ELK3, ETS-domain protein (SRF accessory protein 2) | 0.0004427 | 0.0166 | 1282.7 | 2135.58 | 1186.32 | (1, 2), (3, 2) |
| [204839_at](https://www.affymetrix.com/LinkServlet?probeset=204839_at) | [POP5](http://www.ncbi.nlm.nih.gov/entrez/query.fcgi?cmd=search&db=gene&term=POP5) | processing of precursor 5, ribonuclease P/MRP subunit (S. cerevisiae) | 0.0004433 | 0.0166 | 896.83 | 1109.27 | 708.57 | (3, 2) |
| [213763_at](https://www.affymetrix.com/LinkServlet?probeset=213763_at) | [HIPK2](http://www.ncbi.nlm.nih.gov/entrez/query.fcgi?cmd=search&db=gene&term=HIPK2) | homeodomain interacting protein kinase 2 | 0.0004434 | 0.0166 | 241.48 | 114.22 | 393.21 | (2, 3) |
| [266_s_at](https://www.affymetrix.com/LinkServlet?probeset=266_s_at) | [CD24](http://www.ncbi.nlm.nih.gov/entrez/query.fcgi?cmd=search&db=gene&term=CD24) | CD24 molecule | 0.0004442 | 0.0166 | 74.12 | 13.77 | 10.74 | (2, 1), (3, 1) |
| [242826_at](https://www.affymetrix.com/LinkServlet?probeset=242826_at) | [NA](http://www.ncbi.nlm.nih.gov/entrez/query.fcgi?cmd=search&db=gene&term=NA) | NA | 0.0004443 | 0.0166 | 127.95 | 127.77 | 212.85 | (1, 3), (2, 3) |
| [203759_at](https://www.affymetrix.com/LinkServlet?probeset=203759_at) | [ST3GAL4](http://www.ncbi.nlm.nih.gov/entrez/query.fcgi?cmd=search&db=gene&term=ST3GAL4) | ST3 beta-galactoside alpha-2,3-sialyltransferase 4 | 0.0004451 | 0.0166 | 36.05 | 19.82 | 14.69 | (3, 1) |
| [240228_at](https://www.affymetrix.com/LinkServlet?probeset=240228_at) | [CSMD3](http://www.ncbi.nlm.nih.gov/entrez/query.fcgi?cmd=search&db=gene&term=CSMD3) | CUB and Sushi multiple domains 3 | 0.000453 | 0.0168 | 84.42 | 241.08 | 353.15 | (1, 2), (1, 3) |
| [226022_at](https://www.affymetrix.com/LinkServlet?probeset=226022_at) | [SASH1](http://www.ncbi.nlm.nih.gov/entrez/query.fcgi?cmd=search&db=gene&term=SASH1) | SAM and SH3 domain containing 1 | 0.0004545 | 0.0168 | 2015.59 | 1609.7 | 2885.92 | (2, 3) |
| [213040_s_at](https://www.affymetrix.com/LinkServlet?probeset=213040_s_at) | [NPTXR](http://www.ncbi.nlm.nih.gov/entrez/query.fcgi?cmd=search&db=gene&term=NPTXR) | neuronal pentraxin receptor | 0.0004569 | 0.0169 | 203.99 | 60.25 | 81.21 | (2, 1), (3, 1) |
| [227792_at](https://www.affymetrix.com/LinkServlet?probeset=227792_at) | [ITPRIPL2](http://www.ncbi.nlm.nih.gov/entrez/query.fcgi?cmd=search&db=gene&term=ITPRIPL2) | inositol 1,4,5-triphosphate receptor interacting protein-like 2 | 0.0004576 | 0.0169 | 1748.24 | 1966.16 | 1160.37 | (3, 1), (3, 2) |
| [219779_at](https://www.affymetrix.com/LinkServlet?probeset=219779_at) | [ZFHX4](http://www.ncbi.nlm.nih.gov/entrez/query.fcgi?cmd=search&db=gene&term=ZFHX4) | zinc finger homeobox 4 | 0.0004617 | 0.017 | 192.95 | 62.77 | 25.61 | (3, 1) |
| [212651_at](https://www.affymetrix.com/LinkServlet?probeset=212651_at) | [RHOBTB1](http://www.ncbi.nlm.nih.gov/entrez/query.fcgi?cmd=search&db=gene&term=RHOBTB1) | Rho-related BTB domain containing 1 | 0.0004649 | 0.0171 | 280.95 | 359.85 | 191.03 | (3, 2) |
| [219119_at](https://www.affymetrix.com/LinkServlet?probeset=219119_at) | [LSM8](http://www.ncbi.nlm.nih.gov/entrez/query.fcgi?cmd=search&db=gene&term=LSM8) | LSM8 homolog, U6 small nuclear RNA associated (S. cerevisiae) | 0.0004651 | 0.0171 | 527.02 | 594.65 | 338.67 | (3, 1), (3, 2) |
| [209485_s_at](https://www.affymetrix.com/LinkServlet?probeset=209485_s_at) | [OSBPL1A](http://www.ncbi.nlm.nih.gov/entrez/query.fcgi?cmd=search&db=gene&term=OSBPL1A) | oxysterol binding protein-like 1A | 0.0004657 | 0.0171 | 1339.72 | 872.13 | 1760.67 | (2, 3) |
| [219263_at](https://www.affymetrix.com/LinkServlet?probeset=219263_at) | [RNF128](http://www.ncbi.nlm.nih.gov/entrez/query.fcgi?cmd=search&db=gene&term=RNF128) | ring finger protein 128 | 0.000467 | 0.0171 | 131.94 | 123.3 | 622.73 | (1, 3), (2, 3) |
| [223855_s_at](https://www.affymetrix.com/LinkServlet?probeset=223855_s_at) | [MCHR1](http://www.ncbi.nlm.nih.gov/entrez/query.fcgi?cmd=search&db=gene&term=MCHR1) | melanin-concentrating hormone receptor 1 | 0.0004684 | 0.0171 | 131.48 | 65.99 | 33.13 | (3, 1) |
| [218454_at](https://www.affymetrix.com/LinkServlet?probeset=218454_at) | [PLBD1](http://www.ncbi.nlm.nih.gov/entrez/query.fcgi?cmd=search&db=gene&term=PLBD1) | phospholipase B domain containing 1 | 0.0004689 | 0.0171 | 117.18 | 168.72 | 57.53 | (3, 2) |
| [223134_at](https://www.affymetrix.com/LinkServlet?probeset=223134_at) | [BBX](http://www.ncbi.nlm.nih.gov/entrez/query.fcgi?cmd=search&db=gene&term=BBX) | bobby sox homolog (Drosophila) | 0.0004739 | 0.0172 | 1351.42 | 989.75 | 1689.66 | (2, 3) |
| [1554298_a_at](https://www.affymetrix.com/LinkServlet?probeset=1554298_a_at) | [WDR49](http://www.ncbi.nlm.nih.gov/entrez/query.fcgi?cmd=search&db=gene&term=WDR49) | WD repeat domain 49 | 0.0004739 | 0.0172 | 10.43 | 37.8 | 55.23 | (1, 2), (1, 3) |
| [202909_at](https://www.affymetrix.com/LinkServlet?probeset=202909_at) | [EPM2AIP1](http://www.ncbi.nlm.nih.gov/entrez/query.fcgi?cmd=search&db=gene&term=EPM2AIP1) | EPM2A (laforin) interacting protein 1 | 0.000474 | 0.0172 | 2025.35 | 1802.44 | 2732.52 | (2, 3) |
| [232027_at](https://www.affymetrix.com/LinkServlet?probeset=232027_at) | [SYNE1](http://www.ncbi.nlm.nih.gov/entrez/query.fcgi?cmd=search&db=gene&term=SYNE1) | spectrin repeat containing, nuclear envelope 1 | 0.0004838 | 0.0175 | 18.2 | 26.49 | 80.55 | (1, 3), (2, 3) |
| [227145_at](https://www.affymetrix.com/LinkServlet?probeset=227145_at) | [LOXL4](http://www.ncbi.nlm.nih.gov/entrez/query.fcgi?cmd=search&db=gene&term=LOXL4) | lysyl oxidase-like 4 | 0.0004843 | 0.0175 | 17.66 | 22.66 | 8.64 | (3, 1), (3, 2) |
| [202421_at](https://www.affymetrix.com/LinkServlet?probeset=202421_at) | [IGSF3](http://www.ncbi.nlm.nih.gov/entrez/query.fcgi?cmd=search&db=gene&term=IGSF3) | immunoglobulin superfamily, member 3 | 0.000486 | 0.0176 | 436.35 | 173.15 | 99.62 | (3, 1) |
| [219905_at](https://www.affymetrix.com/LinkServlet?probeset=219905_at) | [ERMAP](http://www.ncbi.nlm.nih.gov/entrez/query.fcgi?cmd=search&db=gene&term=ERMAP) | erythroblast membrane-associated protein (Scianna blood group) | 0.0004866 | 0.0176 | 87.03 | 86.6 | 41.45 | (3, 1), (3, 2) |
| [223396_at](https://www.affymetrix.com/LinkServlet?probeset=223396_at) | [TMEM60](http://www.ncbi.nlm.nih.gov/entrez/query.fcgi?cmd=search&db=gene&term=TMEM60) | transmembrane protein 60 | 0.0004887 | 0.0176 | 515.47 | 538.32 | 333.84 | (3, 1), (3, 2) |
| [213479_at](https://www.affymetrix.com/LinkServlet?probeset=213479_at) | [NPTX2](http://www.ncbi.nlm.nih.gov/entrez/query.fcgi?cmd=search&db=gene&term=NPTX2) | neuronal pentraxin II | 0.0004888 | 0.0176 | 973.04 | 1266.51 | 128.98 | (3, 1), (3, 2) |
| [213362_at](https://www.affymetrix.com/LinkServlet?probeset=213362_at) | [PTPRD](http://www.ncbi.nlm.nih.gov/entrez/query.fcgi?cmd=search&db=gene&term=PTPRD) | protein tyrosine phosphatase, receptor type, D | 0.0004904 | 0.0176 | 174.14 | 170.68 | 442.37 | (1, 3), (2, 3) |
| [212752_at](https://www.affymetrix.com/LinkServlet?probeset=212752_at) | [CLASP1](http://www.ncbi.nlm.nih.gov/entrez/query.fcgi?cmd=search&db=gene&term=CLASP1) | cytoplasmic linker associated protein 1 | 0.0004933 | 0.0177 | 880.65 | 559.26 | 865.45 | (2, 1), (2, 3) |
| [204741_at](https://www.affymetrix.com/LinkServlet?probeset=204741_at) | [BICD1](http://www.ncbi.nlm.nih.gov/entrez/query.fcgi?cmd=search&db=gene&term=BICD1) | bicaudal D homolog 1 (Drosophila) | 0.0004964 | 0.0178 | 201.71 | 163.5 | 111.56 | (3, 1), (3, 2) |
| [235560_at](https://www.affymetrix.com/LinkServlet?probeset=235560_at) | [NA](http://www.ncbi.nlm.nih.gov/entrez/query.fcgi?cmd=search&db=gene&term=NA) | NA | 0.0004978 | 0.0178 | 64.65 | 28.48 | 62.62 | (2, 1), (2, 3) |
| [238468_at](https://www.affymetrix.com/LinkServlet?probeset=238468_at) | [TNRC6B](http://www.ncbi.nlm.nih.gov/entrez/query.fcgi?cmd=search&db=gene&term=TNRC6B) | trinucleotide repeat containing 6B | 0.0004994 | 0.0178 | 218.9 | 119.67 | 238.66 | (2, 1), (2, 3) |
| [37566_at](https://www.affymetrix.com/LinkServlet?probeset=37566_at) | [KIAA1045](http://www.ncbi.nlm.nih.gov/entrez/query.fcgi?cmd=search&db=gene&term=KIAA1045) | KIAA1045 | 0.0005004 | 0.0178 | 14.43 | 6.75 | 6.56 | (2, 1), (3, 1) |
| [219436_s_at](https://www.affymetrix.com/LinkServlet?probeset=219436_s_at) | [EMCN](http://www.ncbi.nlm.nih.gov/entrez/query.fcgi?cmd=search&db=gene&term=EMCN) | endomucin | 0.0005016 | 0.0178 | 42.43 | 28.99 | 13.53 | (3, 1), (3, 2) |
| [1570255_s_at](https://www.affymetrix.com/LinkServlet?probeset=1570255_s_at) | [NA](http://www.ncbi.nlm.nih.gov/entrez/query.fcgi?cmd=search&db=gene&term=NA) | NA | 0.0005017 | 0.0178 | 14.52 | 48.08 | 52.35 | (1, 2), (1, 3) |
| [216379_x_at](https://www.affymetrix.com/LinkServlet?probeset=216379_x_at) | [CD24](http://www.ncbi.nlm.nih.gov/entrez/query.fcgi?cmd=search&db=gene&term=CD24) | CD24 molecule | 0.000502 | 0.0178 | 328.41 | 42.51 | 29.37 | (2, 1), (3, 1) |
| [204069_at](https://www.affymetrix.com/LinkServlet?probeset=204069_at) | [MEIS1](http://www.ncbi.nlm.nih.gov/entrez/query.fcgi?cmd=search&db=gene&term=MEIS1) | Meis homeobox 1 | 0.0005046 | 0.0179 | 142.5 | 145.08 | 330.35 | (1, 3), (2, 3) |
| [228051_at](https://www.affymetrix.com/LinkServlet?probeset=228051_at) | [LOC202451](http://www.ncbi.nlm.nih.gov/entrez/query.fcgi?cmd=search&db=gene&term=LOC202451) | hypothetical protein LOC202451 | 0.0005064 | 0.0179 | 231.16 | 193.84 | 441.67 | (1, 3), (2, 3) |
| [229936_at](https://www.affymetrix.com/LinkServlet?probeset=229936_at) | [GFRA3](http://www.ncbi.nlm.nih.gov/entrez/query.fcgi?cmd=search&db=gene&term=GFRA3) | GDNF family receptor alpha 3 | 0.0005094 | 0.018 | 21.57 | 5.99 | 5.4 | (2, 1), (3, 1) |
| [239481_at](https://www.affymetrix.com/LinkServlet?probeset=239481_at) | [FAM133A](http://www.ncbi.nlm.nih.gov/entrez/query.fcgi?cmd=search&db=gene&term=FAM133A) | family with sequence similarity 133, member A | 0.0005098 | 0.018 | 157.88 | 277.5 | 475.57 | (1, 3) |
| [206638_at](https://www.affymetrix.com/LinkServlet?probeset=206638_at) | [HTR2B](http://www.ncbi.nlm.nih.gov/entrez/query.fcgi?cmd=search&db=gene&term=HTR2B) | 5-hydroxytryptamine (serotonin) receptor 2B | 0.0005109 | 0.018 | 11.26 | 18.97 | 6.36 | (3, 2) |
| [202193_at](https://www.affymetrix.com/LinkServlet?probeset=202193_at) | [LIMK2](http://www.ncbi.nlm.nih.gov/entrez/query.fcgi?cmd=search&db=gene&term=LIMK2) | LIM domain kinase 2 | 0.0005153 | 0.0181 | 141.76 | 373.54 | 351.15 | (1, 2), (1, 3) |
| [205902_at](https://www.affymetrix.com/LinkServlet?probeset=205902_at) | [KCNN3](http://www.ncbi.nlm.nih.gov/entrez/query.fcgi?cmd=search&db=gene&term=KCNN3) | potassium intermediate/small conductance calcium-activated channel, subfamily N, member 3 | 0.0005157 | 0.0181 | 203.98 | 152.54 | 699.93 | (1, 3), (2, 3) |
| [225307_at](https://www.affymetrix.com/LinkServlet?probeset=225307_at) | [ZNF511](http://www.ncbi.nlm.nih.gov/entrez/query.fcgi?cmd=search&db=gene&term=ZNF511) | zinc finger protein 511 | 0.0005159 | 0.0181 | 205.42 | 255.25 | 159.8 | (3, 2) |
| [227733_at](https://www.affymetrix.com/LinkServlet?probeset=227733_at) | [TMEM63C](http://www.ncbi.nlm.nih.gov/entrez/query.fcgi?cmd=search&db=gene&term=TMEM63C) | transmembrane protein 63C | 0.000518 | 0.0181 | 15.7 | 11.28 | 19.67 | (2, 3) |
| [206462_s_at](https://www.affymetrix.com/LinkServlet?probeset=206462_s_at) | [NTRK3](http://www.ncbi.nlm.nih.gov/entrez/query.fcgi?cmd=search&db=gene&term=NTRK3) | neurotrophic tyrosine kinase, receptor, type 3 | 0.0005182 | 0.0181 | 123.22 | 151.42 | 339.07 | (1, 3), (2, 3) |
| [205104_at](https://www.affymetrix.com/LinkServlet?probeset=205104_at) | [SNPH](http://www.ncbi.nlm.nih.gov/entrez/query.fcgi?cmd=search&db=gene&term=SNPH) | syntaphilin | 0.0005186 | 0.0181 | 132.92 | 114.26 | 220.88 | (1, 3), (2, 3) |
| [210067_at](https://www.affymetrix.com/LinkServlet?probeset=210067_at) | [AQP4](http://www.ncbi.nlm.nih.gov/entrez/query.fcgi?cmd=search&db=gene&term=AQP4) | aquaporin 4 | 0.0005199 | 0.0181 | 19.46 | 65.21 | 116.25 | (1, 3) |
| [231001_at](https://www.affymetrix.com/LinkServlet?probeset=231001_at) | [FIBIN](http://www.ncbi.nlm.nih.gov/entrez/query.fcgi?cmd=search&db=gene&term=FIBIN) | fin bud initiation factor homolog (zebrafish) | 0.0005216 | 0.0181 | 144.73 | 120.01 | 314.84 | (1, 3), (2, 3) |
| [215407_s_at](https://www.affymetrix.com/LinkServlet?probeset=215407_s_at) | [ASTN2](http://www.ncbi.nlm.nih.gov/entrez/query.fcgi?cmd=search&db=gene&term=ASTN2) | astrotactin 2 | 0.0005219 | 0.0181 | 515.46 | 752.13 | 1158.19 | (1, 3) |
| [209243_s_at](https://www.affymetrix.com/LinkServlet?probeset=209243_s_at) | [NA](http://www.ncbi.nlm.nih.gov/entrez/query.fcgi?cmd=search&db=gene&term=NA) | NA | 0.0005246 | 0.0181 | 728.8 | 563.9 | 1561.08 | (1, 3), (2, 3) |
| [220146_at](https://www.affymetrix.com/LinkServlet?probeset=220146_at) | [TLR7](http://www.ncbi.nlm.nih.gov/entrez/query.fcgi?cmd=search&db=gene&term=TLR7) | toll-like receptor 7 | 0.0005251 | 0.0181 | 160.6 | 313.62 | 86.16 | (3, 2) |
| [210319_x_at](https://www.affymetrix.com/LinkServlet?probeset=210319_x_at) | [MSX2](http://www.ncbi.nlm.nih.gov/entrez/query.fcgi?cmd=search&db=gene&term=MSX2) | msh homeobox 2 | 0.0005253 | 0.0181 | 7.58 | 6.66 | 19.02 | (1, 3), (2, 3) |
| [226073_at](https://www.affymetrix.com/LinkServlet?probeset=226073_at) | [TMEM218](http://www.ncbi.nlm.nih.gov/entrez/query.fcgi?cmd=search&db=gene&term=TMEM218) | transmembrane protein 218 | 0.0005257 | 0.0181 | 288.94 | 447.56 | 273.61 | (1, 2), (3, 2) |
| [219837_s_at](https://www.affymetrix.com/LinkServlet?probeset=219837_s_at) | [CYTL1](http://www.ncbi.nlm.nih.gov/entrez/query.fcgi?cmd=search&db=gene&term=CYTL1) | cytokine-like 1 | 0.000526 | 0.0181 | 94.55 | 153.57 | 34.12 | (3, 2) |
| [228268_at](https://www.affymetrix.com/LinkServlet?probeset=228268_at) | [FMO2](http://www.ncbi.nlm.nih.gov/entrez/query.fcgi?cmd=search&db=gene&term=FMO2) | flavin containing monooxygenase 2 (non-functional) | 0.0005265 | 0.0181 | 7.78 | 85.09 | 34.82 | (1, 2), (1, 3) |
| [201562_s_at](https://www.affymetrix.com/LinkServlet?probeset=201562_s_at) | [SORD](http://www.ncbi.nlm.nih.gov/entrez/query.fcgi?cmd=search&db=gene&term=SORD) | sorbitol dehydrogenase | 0.0005294 | 0.0182 | 31.21 | 50.7 | 27.35 | (3, 2) |
| [229909_at](https://www.affymetrix.com/LinkServlet?probeset=229909_at) | [B4GALNT3](http://www.ncbi.nlm.nih.gov/entrez/query.fcgi?cmd=search&db=gene&term=B4GALNT3) | beta-1,4-N-acetyl-galactosaminyl transferase 3 | 0.0005327 | 0.0182 | 14.07 | 7.4 | 7.83 | (2, 1), (3, 1) |
| [223282_at](https://www.affymetrix.com/LinkServlet?probeset=223282_at) | [TSHZ1](http://www.ncbi.nlm.nih.gov/entrez/query.fcgi?cmd=search&db=gene&term=TSHZ1) | teashirt zinc finger homeobox 1 | 0.0005339 | 0.0182 | 863.97 | 970.04 | 1479.27 | (1, 3), (2, 3) |
| [218634_at](https://www.affymetrix.com/LinkServlet?probeset=218634_at) | [PHLDA3](http://www.ncbi.nlm.nih.gov/entrez/query.fcgi?cmd=search&db=gene&term=PHLDA3) | pleckstrin homology-like domain, family A, member 3 | 0.0005373 | 0.0183 | 117.01 | 68.54 | 60.18 | (2, 1), (3, 1) |
| [206051_at](https://www.affymetrix.com/LinkServlet?probeset=206051_at) | [ELAVL4](http://www.ncbi.nlm.nih.gov/entrez/query.fcgi?cmd=search&db=gene&term=ELAVL4) | ELAV (embryonic lethal, abnormal vision, Drosophila)-like 4 (Hu antigen D) | 0.0005387 | 0.0184 | 197.85 | 80.34 | 421.11 | (2, 3) |
| [229144_at](https://www.affymetrix.com/LinkServlet?probeset=229144_at) | [RP1-21O18.1](http://www.ncbi.nlm.nih.gov/entrez/query.fcgi?cmd=search&db=gene&term=RP1-21O18.1) | kazrin | 0.0005402 | 0.0184 | 112.21 | 88.21 | 162.02 | (2, 3) |
| [1554332_a_at](https://www.affymetrix.com/LinkServlet?probeset=1554332_a_at) | [hCG_2018279](http://www.ncbi.nlm.nih.gov/entrez/query.fcgi?cmd=search&db=gene&term=hCG_2018279) | hypothetical protein LOC100127888 | 0.0005498 | 0.0186 | 14.12 | 22.08 | 13.47 | (1, 2), (3, 2) |
| [209685_s_at](https://www.affymetrix.com/LinkServlet?probeset=209685_s_at) | [PRKCB](http://www.ncbi.nlm.nih.gov/entrez/query.fcgi?cmd=search&db=gene&term=PRKCB) | protein kinase C, beta | 0.0005499 | 0.0186 | 635.54 | 213.59 | 541.15 | (2, 1), (2, 3) |
| [1556629_a_at](https://www.affymetrix.com/LinkServlet?probeset=1556629_a_at) | [SNAP25](http://www.ncbi.nlm.nih.gov/entrez/query.fcgi?cmd=search&db=gene&term=SNAP25) | synaptosomal-associated protein, 25kDa | 0.0005503 | 0.0186 | 41.99 | 34.11 | 125.82 | (1, 3), (2, 3) |
| [209708_at](https://www.affymetrix.com/LinkServlet?probeset=209708_at) | [MOXD1](http://www.ncbi.nlm.nih.gov/entrez/query.fcgi?cmd=search&db=gene&term=MOXD1) | monooxygenase, DBH-like 1 | 0.0005507 | 0.0186 | 190 | 26.1 | 22.24 | (2, 1), (3, 1) |
| [208405_s_at](https://www.affymetrix.com/LinkServlet?probeset=208405_s_at) | [CD164](http://www.ncbi.nlm.nih.gov/entrez/query.fcgi?cmd=search&db=gene&term=CD164) | CD164 molecule, sialomucin | 0.0005562 | 0.0188 | 2162.19 | 2630.65 | 1711.8 | (3, 2) |
| [228531_at](https://www.affymetrix.com/LinkServlet?probeset=228531_at) | [SAMD9](http://www.ncbi.nlm.nih.gov/entrez/query.fcgi?cmd=search&db=gene&term=SAMD9) | sterile alpha motif domain containing 9 | 0.0005646 | 0.019 | 222.73 | 230.6 | 125.18 | (3, 1), (3, 2) |
| [213035_at](https://www.affymetrix.com/LinkServlet?probeset=213035_at) | [ANKRD28](http://www.ncbi.nlm.nih.gov/entrez/query.fcgi?cmd=search&db=gene&term=ANKRD28) | ankyrin repeat domain 28 | 0.0005671 | 0.0191 | 260.65 | 274.47 | 430.77 | (1, 3), (2, 3) |
| [224872_at](https://www.affymetrix.com/LinkServlet?probeset=224872_at) | [DIP2B](http://www.ncbi.nlm.nih.gov/entrez/query.fcgi?cmd=search&db=gene&term=DIP2B) | DIP2 disco-interacting protein 2 homolog B (Drosophila) | 0.0005693 | 0.0191 | 1003.25 | 593.11 | 1038.29 | (2, 1), (2, 3) |
| [235918_x_at](https://www.affymetrix.com/LinkServlet?probeset=235918_x_at) | [CEP97](http://www.ncbi.nlm.nih.gov/entrez/query.fcgi?cmd=search&db=gene&term=CEP97) | centrosomal protein 97kDa | 0.0005751 | 0.0193 | 221.86 | 176.95 | 300.09 | (2, 3) |
| [229585_at](https://www.affymetrix.com/LinkServlet?probeset=229585_at) | [ADAMTSL1](http://www.ncbi.nlm.nih.gov/entrez/query.fcgi?cmd=search&db=gene&term=ADAMTSL1) | ADAMTS-like 1 | 0.0005764 | 0.0193 | 38.09 | 71.27 | 19.08 | (3, 2) |
| [217967_s_at](https://www.affymetrix.com/LinkServlet?probeset=217967_s_at) | [FAM129A](http://www.ncbi.nlm.nih.gov/entrez/query.fcgi?cmd=search&db=gene&term=FAM129A) | family with sequence similarity 129, member A | 0.0005794 | 0.0194 | 460.84 | 847.67 | 358.09 | (3, 2) |
| [231234_at](https://www.affymetrix.com/LinkServlet?probeset=231234_at) | [CTSC](http://www.ncbi.nlm.nih.gov/entrez/query.fcgi?cmd=search&db=gene&term=CTSC) | cathepsin C | 0.0005807 | 0.0194 | 107.55 | 265.82 | 145.09 | (1, 2), (3, 2) |
| [204249_s_at](https://www.affymetrix.com/LinkServlet?probeset=204249_s_at) | [LMO2](http://www.ncbi.nlm.nih.gov/entrez/query.fcgi?cmd=search&db=gene&term=LMO2) | LIM domain only 2 (rhombotin-like 1) | 0.000583 | 0.0194 | 2330.17 | 3943.14 | 1705.91 | (3, 2) |
| [226362_at](https://www.affymetrix.com/LinkServlet?probeset=226362_at) | [NA](http://www.ncbi.nlm.nih.gov/entrez/query.fcgi?cmd=search&db=gene&term=NA) | NA | 0.0005846 | 0.0195 | 40.6 | 96.76 | 97.73 | (1, 2), (1, 3) |
| [207542_s_at](https://www.affymetrix.com/LinkServlet?probeset=207542_s_at) | [AQP1](http://www.ncbi.nlm.nih.gov/entrez/query.fcgi?cmd=search&db=gene&term=AQP1) | aquaporin 1 (Colton blood group) | 0.0005869 | 0.0195 | 34.94 | 31.28 | 188.59 | (1, 3), (2, 3) |
| [1556212_x_at](https://www.affymetrix.com/LinkServlet?probeset=1556212_x_at) | [NA](http://www.ncbi.nlm.nih.gov/entrez/query.fcgi?cmd=search&db=gene&term=NA) | NA | 0.0005876 | 0.0195 | 11.64 | 13.02 | 28.16 | (1, 3), (2, 3) |
| [213418_at](https://www.affymetrix.com/LinkServlet?probeset=213418_at) | [HSPA6](http://www.ncbi.nlm.nih.gov/entrez/query.fcgi?cmd=search&db=gene&term=HSPA6) | heat shock 70kDa protein 6 (HSP70B') | 0.0005884 | 0.0195 | 135.31 | 144 | 44.64 | (3, 1), (3, 2) |
| [228088_at](https://www.affymetrix.com/LinkServlet?probeset=228088_at) | [SESTD1](http://www.ncbi.nlm.nih.gov/entrez/query.fcgi?cmd=search&db=gene&term=SESTD1) | SEC14 and spectrin domains 1 | 0.0005893 | 0.0195 | 172.25 | 129.52 | 255.33 | (2, 3) |
| [212492_s_at](https://www.affymetrix.com/LinkServlet?probeset=212492_s_at) | [KDM4B](http://www.ncbi.nlm.nih.gov/entrez/query.fcgi?cmd=search&db=gene&term=KDM4B) | lysine (K)-specific demethylase 4B | 0.00059 | 0.0195 | 174.89 | 84.06 | 190.89 | (2, 1), (2, 3) |
| [214983_at](https://www.affymetrix.com/LinkServlet?probeset=214983_at) | [TTTY15](http://www.ncbi.nlm.nih.gov/entrez/query.fcgi?cmd=search&db=gene&term=TTTY15) | testis-specific transcript, Y-linked 15 (non-protein coding) | 0.0005933 | 0.0196 | 37.06 | 12.3 | 52.19 | (2, 3) |
| [205241_at](https://www.affymetrix.com/LinkServlet?probeset=205241_at) | [SCO2](http://www.ncbi.nlm.nih.gov/entrez/query.fcgi?cmd=search&db=gene&term=SCO2) | SCO cytochrome oxidase deficient homolog 2 (yeast) | 0.0005964 | 0.0196 | 206.27 | 250.97 | 133.11 | (3, 2) |
| [213060_s_at](https://www.affymetrix.com/LinkServlet?probeset=213060_s_at) | [CHI3L2](http://www.ncbi.nlm.nih.gov/entrez/query.fcgi?cmd=search&db=gene&term=CHI3L2) | chitinase 3-like 2 | 0.0006004 | 0.0198 | 84.71 | 825.47 | 1065.82 | (1, 2), (1, 3) |
| [227995_at](https://www.affymetrix.com/LinkServlet?probeset=227995_at) | [NA](http://www.ncbi.nlm.nih.gov/entrez/query.fcgi?cmd=search&db=gene&term=NA) | NA | 0.0006014 | 0.0198 | 225.06 | 254.84 | 131.25 | (3, 1), (3, 2) |
| [228749_at](https://www.affymetrix.com/LinkServlet?probeset=228749_at) | [ZDBF2](http://www.ncbi.nlm.nih.gov/entrez/query.fcgi?cmd=search&db=gene&term=ZDBF2) | zinc finger, DBF-type containing 2 | 0.0006028 | 0.0198 | 130.55 | 194.55 | 391.27 | (1, 3) |
| [226085_at](https://www.affymetrix.com/LinkServlet?probeset=226085_at) | [CBX5](http://www.ncbi.nlm.nih.gov/entrez/query.fcgi?cmd=search&db=gene&term=CBX5) | chromobox homolog 5 (HP1 alpha homolog, Drosophila) | 0.0006058 | 0.0198 | 1422.25 | 918.37 | 1771.77 | (2, 3) |
| [234101_at](https://www.affymetrix.com/LinkServlet?probeset=234101_at) | [NA](http://www.ncbi.nlm.nih.gov/entrez/query.fcgi?cmd=search&db=gene&term=NA) | NA | 0.0006098 | 0.0199 | 11.71 | 19.77 | 41.09 | (1, 3) |
| [204040_at](https://www.affymetrix.com/LinkServlet?probeset=204040_at) | [RNF144A](http://www.ncbi.nlm.nih.gov/entrez/query.fcgi?cmd=search&db=gene&term=RNF144A) | ring finger protein 144A | 0.00061 | 0.0199 | 173.73 | 187.72 | 407.46 | (1, 3), (2, 3) |
| [212817_at](https://www.affymetrix.com/LinkServlet?probeset=212817_at) | [DNAJB5](http://www.ncbi.nlm.nih.gov/entrez/query.fcgi?cmd=search&db=gene&term=DNAJB5) | DnaJ (Hsp40) homolog, subfamily B, member 5 | 0.0006108 | 0.0199 | 190.29 | 105.6 | 111.93 | (2, 1), (3, 1) |
| [1552714_at](https://www.affymetrix.com/LinkServlet?probeset=1552714_at) | [CREG2](http://www.ncbi.nlm.nih.gov/entrez/query.fcgi?cmd=search&db=gene&term=CREG2) | cellular repressor of E1A-stimulated genes 2 | 0.0006129 | 0.0199 | 11.55 | 4.77 | 4.8 | (2, 1), (3, 1) |
| [213189_at](https://www.affymetrix.com/LinkServlet?probeset=213189_at) | [NA](http://www.ncbi.nlm.nih.gov/entrez/query.fcgi?cmd=search&db=gene&term=NA) | NA | 0.000614 | 0.0199 | 156.43 | 382.87 | 223.11 | (1, 2), (3, 2) |
| [202438_x_at](https://www.affymetrix.com/LinkServlet?probeset=202438_x_at) | [IDS](http://www.ncbi.nlm.nih.gov/entrez/query.fcgi?cmd=search&db=gene&term=IDS) | iduronate 2-sulfatase | 0.0006155 | 0.0199 | 30.07 | 21.59 | 15.9 | (3, 1) |
| [230550_at](https://www.affymetrix.com/LinkServlet?probeset=230550_at) | [MS4A6A](http://www.ncbi.nlm.nih.gov/entrez/query.fcgi?cmd=search&db=gene&term=MS4A6A) | membrane-spanning 4-domains, subfamily A, member 6A | 0.0006171 | 0.0199 | 164.06 | 328.51 | 108.7 | (3, 2) |
| [206249_at](https://www.affymetrix.com/LinkServlet?probeset=206249_at) | [MAP3K13](http://www.ncbi.nlm.nih.gov/entrez/query.fcgi?cmd=search&db=gene&term=MAP3K13) | mitogen-activated protein kinase kinase kinase 13 | 0.0006174 | 0.0199 | 20.08 | 11.56 | 20.24 | (2, 1), (2, 3) |
| [204419_x_at](https://www.affymetrix.com/LinkServlet?probeset=204419_x_at) | [NA](http://www.ncbi.nlm.nih.gov/entrez/query.fcgi?cmd=search&db=gene&term=NA) | NA | 0.0006175 | 0.0199 | 30.9 | 79.99 | 16.66 | (3, 2) |
| [205890_s_at](https://www.affymetrix.com/LinkServlet?probeset=205890_s_at) | [NA](http://www.ncbi.nlm.nih.gov/entrez/query.fcgi?cmd=search&db=gene&term=NA) | NA | 0.0006181 | 0.0199 | 8.97 | 33.2 | 8.2 | (1, 2), (3, 2) |
| [1554547_at](https://www.affymetrix.com/LinkServlet?probeset=1554547_at) | [FAM13C](http://www.ncbi.nlm.nih.gov/entrez/query.fcgi?cmd=search&db=gene&term=FAM13C) | family with sequence similarity 13, member C | 0.0006195 | 0.02 | 502.6 | 902.04 | 619.07 | (1, 2), (3, 2) |
| [225943_at](https://www.affymetrix.com/LinkServlet?probeset=225943_at) | [NLN](http://www.ncbi.nlm.nih.gov/entrez/query.fcgi?cmd=search&db=gene&term=NLN) | neurolysin (metallopeptidase M3 family) | 0.0006211 | 0.02 | 67.18 | 85.25 | 49.05 | (3, 2) |
| [229881_at](https://www.affymetrix.com/LinkServlet?probeset=229881_at) | [KLF12](http://www.ncbi.nlm.nih.gov/entrez/query.fcgi?cmd=search&db=gene&term=KLF12) | Kruppel-like factor 12 | 0.0006215 | 0.02 | 20.94 | 33.64 | 54 | (1, 3) |
| [1554101_a_at](https://www.affymetrix.com/LinkServlet?probeset=1554101_a_at) | [TMTC4](http://www.ncbi.nlm.nih.gov/entrez/query.fcgi?cmd=search&db=gene&term=TMTC4) | transmembrane and tetratricopeptide repeat containing 4 | 0.000623 | 0.02 | 13.17 | 7.12 | 5.22 | (3, 1) |
| [232270_at](https://www.affymetrix.com/LinkServlet?probeset=232270_at) | [C9orf3](http://www.ncbi.nlm.nih.gov/entrez/query.fcgi?cmd=search&db=gene&term=C9orf3) | chromosome 9 open reading frame 3 | 0.0006256 | 0.02 | 8.73 | 14.57 | 9.51 | (1, 2), (3, 2) |
| [206718_at](https://www.affymetrix.com/LinkServlet?probeset=206718_at) | [LMO1](http://www.ncbi.nlm.nih.gov/entrez/query.fcgi?cmd=search&db=gene&term=LMO1) | LIM domain only 1 (rhombotin 1) | 0.0006259 | 0.02 | 15.4 | 8.93 | 6.1 | (3, 1) |
| [1552455_at](https://www.affymetrix.com/LinkServlet?probeset=1552455_at) | [PRUNE2](http://www.ncbi.nlm.nih.gov/entrez/query.fcgi?cmd=search&db=gene&term=PRUNE2) | prune homolog 2 (Drosophila) | 0.0006278 | 0.0201 | 41.73 | 14.7 | 72.81 | (2, 3) |
| [232562_at](https://www.affymetrix.com/LinkServlet?probeset=232562_at) | [NA](http://www.ncbi.nlm.nih.gov/entrez/query.fcgi?cmd=search&db=gene&term=NA) | NA | 0.0006291 | 0.0201 | 29.48 | 17.68 | 45.47 | (2, 3) |
| [208025_s_at](https://www.affymetrix.com/LinkServlet?probeset=208025_s_at) | [HMGA2](http://www.ncbi.nlm.nih.gov/entrez/query.fcgi?cmd=search&db=gene&term=HMGA2) | high mobility group AT-hook 2 | 0.0006317 | 0.0201 | 8.86 | 30.98 | 7.02 | (1, 2), (3, 2) |
| [232856_at](https://www.affymetrix.com/LinkServlet?probeset=232856_at) | [LRRC55](http://www.ncbi.nlm.nih.gov/entrez/query.fcgi?cmd=search&db=gene&term=LRRC55) | leucine rich repeat containing 55 | 0.000634 | 0.0202 | 38.22 | 135.3 | 302.89 | (1, 3) |
| [227889_at](https://www.affymetrix.com/LinkServlet?probeset=227889_at) | [LPCAT2](http://www.ncbi.nlm.nih.gov/entrez/query.fcgi?cmd=search&db=gene&term=LPCAT2) | lysophosphatidylcholine acyltransferase 2 | 0.0006389 | 0.0203 | 340.89 | 450.76 | 178.43 | (3, 2) |
| [210651_s_at](https://www.affymetrix.com/LinkServlet?probeset=210651_s_at) | [EPHB2](http://www.ncbi.nlm.nih.gov/entrez/query.fcgi?cmd=search&db=gene&term=EPHB2) | EPH receptor B2 | 0.000639 | 0.0203 | 26.04 | 37.52 | 18.16 | (3, 2) |
| [206762_at](https://www.affymetrix.com/LinkServlet?probeset=206762_at) | [KCNA5](http://www.ncbi.nlm.nih.gov/entrez/query.fcgi?cmd=search&db=gene&term=KCNA5) | potassium voltage-gated channel, shaker-related subfamily, member 5 | 0.0006416 | 0.0203 | 53.9 | 20.84 | 54.79 | (2, 1), (2, 3) |
| [224275_at](https://www.affymetrix.com/LinkServlet?probeset=224275_at) | [GPR98](http://www.ncbi.nlm.nih.gov/entrez/query.fcgi?cmd=search&db=gene&term=GPR98) | G protein-coupled receptor 98 | 0.0006421 | 0.0203 | 5.74 | 10.79 | 4.78 | (3, 2) |
| [233498_at](https://www.affymetrix.com/LinkServlet?probeset=233498_at) | [ERBB4](http://www.ncbi.nlm.nih.gov/entrez/query.fcgi?cmd=search&db=gene&term=ERBB4) | v-erb-a erythroblastic leukemia viral oncogene homolog 4 (avian) | 0.0006505 | 0.0205 | 56.04 | 152.02 | 202.03 | (1, 2), (1, 3) |
| [214994_at](https://www.affymetrix.com/LinkServlet?probeset=214994_at) | [APOBEC3F](http://www.ncbi.nlm.nih.gov/entrez/query.fcgi?cmd=search&db=gene&term=APOBEC3F) | apolipoprotein B mRNA editing enzyme, catalytic polypeptide-like 3F | 0.0006518 | 0.0205 | 38.78 | 45.65 | 29.05 | (3, 2) |
| [213186_at](https://www.affymetrix.com/LinkServlet?probeset=213186_at) | [DZIP3](http://www.ncbi.nlm.nih.gov/entrez/query.fcgi?cmd=search&db=gene&term=DZIP3) | DAZ interacting protein 3, zinc finger | 0.0006532 | 0.0206 | 446.77 | 438.76 | 673.12 | (1, 3), (2, 3) |
| [210139_s_at](https://www.affymetrix.com/LinkServlet?probeset=210139_s_at) | [PMP22](http://www.ncbi.nlm.nih.gov/entrez/query.fcgi?cmd=search&db=gene&term=PMP22) | peripheral myelin protein 22 | 0.000657 | 0.0207 | 4766.72 | 4807.87 | 3033.09 | (3, 1), (3, 2) |
| [203072_at](https://www.affymetrix.com/LinkServlet?probeset=203072_at) | [MYO1E](http://www.ncbi.nlm.nih.gov/entrez/query.fcgi?cmd=search&db=gene&term=MYO1E) | myosin IE | 0.0006608 | 0.0207 | 44.32 | 23.93 | 18.89 | (3, 1) |
| [229723_at](https://www.affymetrix.com/LinkServlet?probeset=229723_at) | [TAGAP](http://www.ncbi.nlm.nih.gov/entrez/query.fcgi?cmd=search&db=gene&term=TAGAP) | T-cell activation RhoGTPase activating protein | 0.0006613 | 0.0207 | 161.7 | 263.1 | 84.43 | (3, 2) |
| [206693_at](https://www.affymetrix.com/LinkServlet?probeset=206693_at) | [IL7](http://www.ncbi.nlm.nih.gov/entrez/query.fcgi?cmd=search&db=gene&term=IL7) | interleukin 7 | 0.0006648 | 0.0208 | 8.53 | 28.34 | 12.42 | (1, 2), (3, 2) |
| [230641_at](https://www.affymetrix.com/LinkServlet?probeset=230641_at) | [NA](http://www.ncbi.nlm.nih.gov/entrez/query.fcgi?cmd=search&db=gene&term=NA) | NA | 0.000669 | 0.0209 | 15.4 | 30.92 | 18.73 | (1, 2), (3, 2) |
| [208309_s_at](https://www.affymetrix.com/LinkServlet?probeset=208309_s_at) | [MALT1](http://www.ncbi.nlm.nih.gov/entrez/query.fcgi?cmd=search&db=gene&term=MALT1) | mucosa associated lymphoid tissue lymphoma translocation gene 1 | 0.0006812 | 0.0212 | 29.08 | 33.95 | 16.89 | (3, 1), (3, 2) |
| [213832_at](https://www.affymetrix.com/LinkServlet?probeset=213832_at) | [KCND3](http://www.ncbi.nlm.nih.gov/entrez/query.fcgi?cmd=search&db=gene&term=KCND3) | potassium voltage-gated channel, Shal-related subfamily, member 3 | 0.0006816 | 0.0212 | 339.91 | 539.37 | 910.15 | (1, 3) |
| [219797_at](https://www.affymetrix.com/LinkServlet?probeset=219797_at) | [MGAT4A](http://www.ncbi.nlm.nih.gov/entrez/query.fcgi?cmd=search&db=gene&term=MGAT4A) | mannosyl (alpha-1,3-)-glycoprotein beta-1,4-N-acetylglucosaminyltransferase, isozyme A | 0.000683 | 0.0212 | 36.62 | 59.22 | 26.69 | (3, 2) |
| [231929_at](https://www.affymetrix.com/LinkServlet?probeset=231929_at) | [IKZF2](http://www.ncbi.nlm.nih.gov/entrez/query.fcgi?cmd=search&db=gene&term=IKZF2) | IKAROS family zinc finger 2 (Helios) | 0.0006834 | 0.0212 | 112.03 | 210.89 | 246.88 | (1, 2), (1, 3) |
| [1558170_at](https://www.affymetrix.com/LinkServlet?probeset=1558170_at) | [NA](http://www.ncbi.nlm.nih.gov/entrez/query.fcgi?cmd=search&db=gene&term=NA) | NA | 0.0006846 | 0.0212 | 74.35 | 68.94 | 372.75 | (1, 3), (2, 3) |
| [204358_s_at](https://www.affymetrix.com/LinkServlet?probeset=204358_s_at) | [FLRT2](http://www.ncbi.nlm.nih.gov/entrez/query.fcgi?cmd=search&db=gene&term=FLRT2) | fibronectin leucine rich transmembrane protein 2 | 0.0006852 | 0.0212 | 19.48 | 24.6 | 13.86 | (3, 2) |
| [219100_at](https://www.affymetrix.com/LinkServlet?probeset=219100_at) | [OBFC1](http://www.ncbi.nlm.nih.gov/entrez/query.fcgi?cmd=search&db=gene&term=OBFC1) | oligonucleotide/oligosaccharide-binding fold containing 1 | 0.0006878 | 0.0213 | 145.7 | 210.78 | 107.52 | (3, 2) |
| [229506_at](https://www.affymetrix.com/LinkServlet?probeset=229506_at) | [NA](http://www.ncbi.nlm.nih.gov/entrez/query.fcgi?cmd=search&db=gene&term=NA) | NA | 0.0006891 | 0.0213 | 60.94 | 109.82 | 130.45 | (1, 2), (1, 3) |
| [209858_x_at](https://www.affymetrix.com/LinkServlet?probeset=209858_x_at) | [MPPE1](http://www.ncbi.nlm.nih.gov/entrez/query.fcgi?cmd=search&db=gene&term=MPPE1) | metallophosphoesterase 1 | 0.0006927 | 0.0213 | 139.96 | 218.11 | 138 | (1, 2), (3, 2) |
| [224829_at](https://www.affymetrix.com/LinkServlet?probeset=224829_at) | [CPEB4](http://www.ncbi.nlm.nih.gov/entrez/query.fcgi?cmd=search&db=gene&term=CPEB4) | cytoplasmic polyadenylation element binding protein 4 | 0.0006927 | 0.0213 | 632.19 | 512.73 | 916.57 | (2, 3) |
| [209771_x_at](https://www.affymetrix.com/LinkServlet?probeset=209771_x_at) | [CD24](http://www.ncbi.nlm.nih.gov/entrez/query.fcgi?cmd=search&db=gene&term=CD24) | CD24 molecule | 0.0006947 | 0.0214 | 346.88 | 41.68 | 31.62 | (2, 1), (3, 1) |
| [223386_at](https://www.affymetrix.com/LinkServlet?probeset=223386_at) | [FAM118B](http://www.ncbi.nlm.nih.gov/entrez/query.fcgi?cmd=search&db=gene&term=FAM118B) | family with sequence similarity 118, member B | 0.0006975 | 0.0214 | 212.85 | 246.79 | 142.49 | (3, 2) |
| [204316_at](https://www.affymetrix.com/LinkServlet?probeset=204316_at) | [RGS10](http://www.ncbi.nlm.nih.gov/entrez/query.fcgi?cmd=search&db=gene&term=RGS10) | regulator of G-protein signaling 10 | 0.0006999 | 0.0215 | 8.13 | 11.22 | 6.84 | (3, 2) |
| [213960_at](https://www.affymetrix.com/LinkServlet?probeset=213960_at) | [NTRK3](http://www.ncbi.nlm.nih.gov/entrez/query.fcgi?cmd=search&db=gene&term=NTRK3) | neurotrophic tyrosine kinase, receptor, type 3 | 0.000701 | 0.0215 | 284.87 | 462.81 | 761.4 | (1, 3) |
| [1554702_at](https://www.affymetrix.com/LinkServlet?probeset=1554702_at) | [NALCN](http://www.ncbi.nlm.nih.gov/entrez/query.fcgi?cmd=search&db=gene&term=NALCN) | sodium leak channel, non-selective | 0.0007018 | 0.0215 | 6.99 | 8.98 | 12.63 | (1, 3) |
| [207013_s_at](https://www.affymetrix.com/LinkServlet?probeset=207013_s_at) | [MMP16](http://www.ncbi.nlm.nih.gov/entrez/query.fcgi?cmd=search&db=gene&term=MMP16) | matrix metallopeptidase 16 (membrane-inserted) | 0.0007043 | 0.0215 | 16.61 | 27.21 | 30.42 | (1, 2), (1, 3) |
| [237665_at](https://www.affymetrix.com/LinkServlet?probeset=237665_at) | [NA](http://www.ncbi.nlm.nih.gov/entrez/query.fcgi?cmd=search&db=gene&term=NA) | NA | 0.0007061 | 0.0215 | 9.22 | 9.54 | 20.59 | (1, 3), (2, 3) |
| [207336_at](https://www.affymetrix.com/LinkServlet?probeset=207336_at) | [SOX5](http://www.ncbi.nlm.nih.gov/entrez/query.fcgi?cmd=search&db=gene&term=SOX5) | SRY (sex determining region Y)-box 5 | 0.0007085 | 0.0216 | 27.47 | 22.63 | 54.01 | (1, 3), (2, 3) |
| [226074_at](https://www.affymetrix.com/LinkServlet?probeset=226074_at) | [PPM1M](http://www.ncbi.nlm.nih.gov/entrez/query.fcgi?cmd=search&db=gene&term=PPM1M) | protein phosphatase 1M (PP2C domain containing) | 0.0007091 | 0.0216 | 42.1 | 63.14 | 39.6 | (1, 2), (3, 2) |
| [239483_at](https://www.affymetrix.com/LinkServlet?probeset=239483_at) | [FLJ37035](http://www.ncbi.nlm.nih.gov/entrez/query.fcgi?cmd=search&db=gene&term=FLJ37035) | FLJ37035 protein | 0.0007095 | 0.0216 | 20.92 | 29.28 | 17.45 | (3, 2) |
| [231914_at](https://www.affymetrix.com/LinkServlet?probeset=231914_at) | [NUDT14](http://www.ncbi.nlm.nih.gov/entrez/query.fcgi?cmd=search&db=gene&term=NUDT14) | nudix (nucleoside diphosphate linked moiety X)-type motif 14 | 0.0007108 | 0.0216 | 29.27 | 34.29 | 20.32 | (3, 2) |
| [224984_at](https://www.affymetrix.com/LinkServlet?probeset=224984_at) | [NFAT5](http://www.ncbi.nlm.nih.gov/entrez/query.fcgi?cmd=search&db=gene&term=NFAT5) | nuclear factor of activated T-cells 5, tonicity-responsive | 0.0007141 | 0.0216 | 729.07 | 856.76 | 1160.29 | (1, 3) |
| [213486_at](https://www.affymetrix.com/LinkServlet?probeset=213486_at) | [NA](http://www.ncbi.nlm.nih.gov/entrez/query.fcgi?cmd=search&db=gene&term=NA) | NA | 0.0007162 | 0.0217 | 107.3 | 155.39 | 606.31 | (1, 3), (2, 3) |
| [219825_at](https://www.affymetrix.com/LinkServlet?probeset=219825_at) | [CYP26B1](http://www.ncbi.nlm.nih.gov/entrez/query.fcgi?cmd=search&db=gene&term=CYP26B1) | cytochrome P450, family 26, subfamily B, polypeptide 1 | 0.0007182 | 0.0217 | 29.35 | 17.48 | 63.11 | (2, 3) |
| [204447_at](https://www.affymetrix.com/LinkServlet?probeset=204447_at) | [ProSAPiP1](http://www.ncbi.nlm.nih.gov/entrez/query.fcgi?cmd=search&db=gene&term=ProSAPiP1) | ProSAPiP1 protein | 0.0007182 | 0.0217 | 251.3 | 248.17 | 527.53 | (1, 3), (2, 3) |
| [242873_at](https://www.affymetrix.com/LinkServlet?probeset=242873_at) | [NA](http://www.ncbi.nlm.nih.gov/entrez/query.fcgi?cmd=search&db=gene&term=NA) | NA | 0.0007238 | 0.0218 | 18.83 | 17.19 | 10.65 | (3, 1), (3, 2) |
| [213636_at](https://www.affymetrix.com/LinkServlet?probeset=213636_at) | [KIAA1045](http://www.ncbi.nlm.nih.gov/entrez/query.fcgi?cmd=search&db=gene&term=KIAA1045) | KIAA1045 | 0.0007274 | 0.0219 | 10.78 | 4.99 | 5.17 | (2, 1), (3, 1) |
| [1553764_a_at](https://www.affymetrix.com/LinkServlet?probeset=1553764_a_at) | [JUB](http://www.ncbi.nlm.nih.gov/entrez/query.fcgi?cmd=search&db=gene&term=JUB) | jub, ajuba homolog (Xenopus laevis) | 0.0007283 | 0.0219 | 16.13 | 20.97 | 10.39 | (3, 2) |
| [229796_at](https://www.affymetrix.com/LinkServlet?probeset=229796_at) | [SIX4](http://www.ncbi.nlm.nih.gov/entrez/query.fcgi?cmd=search&db=gene&term=SIX4) | SIX homeobox 4 | 0.0007307 | 0.0219 | 21.72 | 89.57 | 46.56 | (1, 2) |
| [218223_s_at](https://www.affymetrix.com/LinkServlet?probeset=218223_s_at) | [PLEKHO1](http://www.ncbi.nlm.nih.gov/entrez/query.fcgi?cmd=search&db=gene&term=PLEKHO1) | pleckstrin homology domain containing, family O member 1 | 0.0007322 | 0.0219 | 348.89 | 390.07 | 259.04 | (3, 2) |
| [225465_at](https://www.affymetrix.com/LinkServlet?probeset=225465_at) | [MAGI1](http://www.ncbi.nlm.nih.gov/entrez/query.fcgi?cmd=search&db=gene&term=MAGI1) | membrane associated guanylate kinase, WW and PDZ domain containing 1 | 0.0007323 | 0.0219 | 266.43 | 141.61 | 360.39 | (2, 3) |
| [237094_at](https://www.affymetrix.com/LinkServlet?probeset=237094_at) | [FAM19A5](http://www.ncbi.nlm.nih.gov/entrez/query.fcgi?cmd=search&db=gene&term=FAM19A5) | family with sequence similarity 19 (chemokine (C-C motif)-like), member A5 | 0.0007332 | 0.0219 | 292.98 | 133.21 | 355.36 | (2, 3) |
| [226786_at](https://www.affymetrix.com/LinkServlet?probeset=226786_at) | [RFX1](http://www.ncbi.nlm.nih.gov/entrez/query.fcgi?cmd=search&db=gene&term=RFX1) | regulatory factor X, 1 (influences HLA class II expression) | 0.0007336 | 0.0219 | 54.01 | 103.84 | 53.27 | (1, 2), (3, 2) |
| [218541_s_at](https://www.affymetrix.com/LinkServlet?probeset=218541_s_at) | [C8orf4](http://www.ncbi.nlm.nih.gov/entrez/query.fcgi?cmd=search&db=gene&term=C8orf4) | chromosome 8 open reading frame 4 | 0.0007352 | 0.0219 | 333.09 | 281.53 | 80.56 | (3, 1), (3, 2) |
| [1559965_at](https://www.affymetrix.com/LinkServlet?probeset=1559965_at) | [LOC100192378](http://www.ncbi.nlm.nih.gov/entrez/query.fcgi?cmd=search&db=gene&term=LOC100192378) | hypothetical LOC100192378 | 0.0007355 | 0.0219 | 118.58 | 37.32 | 17.44 | (3, 1) |
| [201280_s_at](https://www.affymetrix.com/LinkServlet?probeset=201280_s_at) | [DAB2](http://www.ncbi.nlm.nih.gov/entrez/query.fcgi?cmd=search&db=gene&term=DAB2) | disabled homolog 2, mitogen-responsive phosphoprotein (Drosophila) | 0.0007398 | 0.022 | 296.1 | 429.36 | 657.21 | (1, 3) |
| [212666_at](https://www.affymetrix.com/LinkServlet?probeset=212666_at) | [SMURF1](http://www.ncbi.nlm.nih.gov/entrez/query.fcgi?cmd=search&db=gene&term=SMURF1) | SMAD specific E3 ubiquitin protein ligase 1 | 0.0007444 | 0.0221 | 101.84 | 62.88 | 57.93 | (2, 1), (3, 1) |
| [204584_at](https://www.affymetrix.com/LinkServlet?probeset=204584_at) | [L1CAM](http://www.ncbi.nlm.nih.gov/entrez/query.fcgi?cmd=search&db=gene&term=L1CAM) | L1 cell adhesion molecule | 0.000748 | 0.0222 | 251.13 | 106.46 | 25.64 | (3, 1) |
| [217377_x_at](https://www.affymetrix.com/LinkServlet?probeset=217377_x_at) | [NTRK3](http://www.ncbi.nlm.nih.gov/entrez/query.fcgi?cmd=search&db=gene&term=NTRK3) | neurotrophic tyrosine kinase, receptor, type 3 | 0.0007523 | 0.0223 | 119.2 | 149.24 | 312.5 | (1, 3), (2, 3) |
| [205150_s_at](https://www.affymetrix.com/LinkServlet?probeset=205150_s_at) | [KIAA0644](http://www.ncbi.nlm.nih.gov/entrez/query.fcgi?cmd=search&db=gene&term=KIAA0644) | KIAA0644 gene product | 0.0007529 | 0.0223 | 139.12 | 432.07 | 758.29 | (1, 3) |
| [226972_s_at](https://www.affymetrix.com/LinkServlet?probeset=226972_s_at) | [CCDC136](http://www.ncbi.nlm.nih.gov/entrez/query.fcgi?cmd=search&db=gene&term=CCDC136) | coiled-coil domain containing 136 | 0.0007555 | 0.0223 | 28.9 | 43.34 | 72.77 | (1, 3) |
| [206167_s_at](https://www.affymetrix.com/LinkServlet?probeset=206167_s_at) | [ARHGAP6](http://www.ncbi.nlm.nih.gov/entrez/query.fcgi?cmd=search&db=gene&term=ARHGAP6) | Rho GTPase activating protein 6 | 0.0007564 | 0.0223 | 214.54 | 439.58 | 524.29 | (1, 2), (1, 3) |
| [222885_at](https://www.affymetrix.com/LinkServlet?probeset=222885_at) | [EMCN](http://www.ncbi.nlm.nih.gov/entrez/query.fcgi?cmd=search&db=gene&term=EMCN) | endomucin | 0.0007652 | 0.0225 | 132.94 | 118.93 | 59.3 | (3, 1), (3, 2) |
| [229178_at](https://www.affymetrix.com/LinkServlet?probeset=229178_at) | [PRTG](http://www.ncbi.nlm.nih.gov/entrez/query.fcgi?cmd=search&db=gene&term=PRTG) | protogenin homolog (Gallus gallus) | 0.0007687 | 0.0226 | 46.94 | 41.3 | 99.57 | (1, 3), (2, 3) |
| [221082_s_at](https://www.affymetrix.com/LinkServlet?probeset=221082_s_at) | [NDRG3](http://www.ncbi.nlm.nih.gov/entrez/query.fcgi?cmd=search&db=gene&term=NDRG3) | NDRG family member 3 | 0.0007688 | 0.0226 | 40.79 | 38.03 | 61.15 | (1, 3), (2, 3) |
| [210249_s_at](https://www.affymetrix.com/LinkServlet?probeset=210249_s_at) | [NCOA1](http://www.ncbi.nlm.nih.gov/entrez/query.fcgi?cmd=search&db=gene&term=NCOA1) | nuclear receptor coactivator 1 | 0.0007728 | 0.0226 | 693.51 | 661 | 1032.93 | (1, 3), (2, 3) |
| [228910_at](https://www.affymetrix.com/LinkServlet?probeset=228910_at) | [NA](http://www.ncbi.nlm.nih.gov/entrez/query.fcgi?cmd=search&db=gene&term=NA) | NA | 0.0007729 | 0.0226 | 94.7 | 91.27 | 251.47 | (1, 3), (2, 3) |
| [228298_at](https://www.affymetrix.com/LinkServlet?probeset=228298_at) | [FAM113B](http://www.ncbi.nlm.nih.gov/entrez/query.fcgi?cmd=search&db=gene&term=FAM113B) | family with sequence similarity 113, member B | 0.0007744 | 0.0226 | 7.91 | 10.27 | 6.34 | (3, 2) |
| [225807_at](https://www.affymetrix.com/LinkServlet?probeset=225807_at) | [JUB](http://www.ncbi.nlm.nih.gov/entrez/query.fcgi?cmd=search&db=gene&term=JUB) | jub, ajuba homolog (Xenopus laevis) | 0.0007751 | 0.0226 | 10.82 | 13.78 | 8.52 | (3, 2) |
| [231484_at](https://www.affymetrix.com/LinkServlet?probeset=231484_at) | [NA](http://www.ncbi.nlm.nih.gov/entrez/query.fcgi?cmd=search&db=gene&term=NA) | NA | 0.0007794 | 0.0227 | 12.96 | 10.06 | 31.73 | (1, 3), (2, 3) |
| [201922_at](https://www.affymetrix.com/LinkServlet?probeset=201922_at) | [TINP1](http://www.ncbi.nlm.nih.gov/entrez/query.fcgi?cmd=search&db=gene&term=TINP1) | TGF beta-inducible nuclear protein 1 | 0.0007815 | 0.0227 | 2928.22 | 2808.8 | 1940.06 | (3, 1), (3, 2) |
| [206990_at](https://www.affymetrix.com/LinkServlet?probeset=206990_at) | [TNR](http://www.ncbi.nlm.nih.gov/entrez/query.fcgi?cmd=search&db=gene&term=TNR) | tenascin R (restrictin, janusin) | 0.0007821 | 0.0227 | 18.34 | 16.24 | 29.26 | (1, 3), (2, 3) |
| [226933_s_at](https://www.affymetrix.com/LinkServlet?probeset=226933_s_at) | [ID4](http://www.ncbi.nlm.nih.gov/entrez/query.fcgi?cmd=search&db=gene&term=ID4) | inhibitor of DNA binding 4, dominant negative helix-loop-helix protein | 0.0007829 | 0.0227 | 224.81 | 471.24 | 554.65 | (1, 2), (1, 3) |
| [228900_at](https://www.affymetrix.com/LinkServlet?probeset=228900_at) | [CYTSB](http://www.ncbi.nlm.nih.gov/entrez/query.fcgi?cmd=search&db=gene&term=CYTSB) | cytospin B | 0.0007852 | 0.0228 | 372.84 | 212.33 | 170.55 | (3, 1) |
| [231919_at](https://www.affymetrix.com/LinkServlet?probeset=231919_at) | [DBT](http://www.ncbi.nlm.nih.gov/entrez/query.fcgi?cmd=search&db=gene&term=DBT) | dihydrolipoamide branched chain transacylase E2 | 0.0007854 | 0.0228 | 85.64 | 93.6 | 128.81 | (1, 3), (2, 3) |
| [204834_at](https://www.affymetrix.com/LinkServlet?probeset=204834_at) | [FGL2](http://www.ncbi.nlm.nih.gov/entrez/query.fcgi?cmd=search&db=gene&term=FGL2) | fibrinogen-like 2 | 0.0007946 | 0.023 | 224.35 | 312.2 | 127.72 | (3, 2) |
| [218574_s_at](https://www.affymetrix.com/LinkServlet?probeset=218574_s_at) | [LMCD1](http://www.ncbi.nlm.nih.gov/entrez/query.fcgi?cmd=search&db=gene&term=LMCD1) | LIM and cysteine-rich domains 1 | 0.0007948 | 0.023 | 86.82 | 181.81 | 183.65 | (1, 2), (1, 3) |
| [222797_at](https://www.affymetrix.com/LinkServlet?probeset=222797_at) | [DPYSL5](http://www.ncbi.nlm.nih.gov/entrez/query.fcgi?cmd=search&db=gene&term=DPYSL5) | dihydropyrimidinase-like 5 | 0.0007951 | 0.023 | 53.76 | 21.84 | 31.52 | (2, 1), (3, 1) |
| [207966_s_at](https://www.affymetrix.com/LinkServlet?probeset=207966_s_at) | [GLG1](http://www.ncbi.nlm.nih.gov/entrez/query.fcgi?cmd=search&db=gene&term=GLG1) | golgi apparatus protein 1 | 0.0007989 | 0.023 | 961.79 | 477.94 | 1092.32 | (2, 1), (2, 3) |
| [244859_at](https://www.affymetrix.com/LinkServlet?probeset=244859_at) | [NA](http://www.ncbi.nlm.nih.gov/entrez/query.fcgi?cmd=search&db=gene&term=NA) | NA | 0.0008019 | 0.0231 | 54.58 | 44.78 | 114.99 | (1, 3), (2, 3) |
| [227265_at](https://www.affymetrix.com/LinkServlet?probeset=227265_at) | [FGL2](http://www.ncbi.nlm.nih.gov/entrez/query.fcgi?cmd=search&db=gene&term=FGL2) | fibrinogen-like 2 | 0.0008032 | 0.0231 | 942.96 | 1609.28 | 611.81 | (3, 2) |
| [225808_at](https://www.affymetrix.com/LinkServlet?probeset=225808_at) | [C17orf95](http://www.ncbi.nlm.nih.gov/entrez/query.fcgi?cmd=search&db=gene&term=C17orf95) | chromosome 17 open reading frame 95 | 0.0008035 | 0.0231 | 791.15 | 891.35 | 596.98 | (3, 2) |
| [1552439_s_at](https://www.affymetrix.com/LinkServlet?probeset=1552439_s_at) | [MEGF11](http://www.ncbi.nlm.nih.gov/entrez/query.fcgi?cmd=search&db=gene&term=MEGF11) | multiple EGF-like-domains 11 | 0.0008043 | 0.0231 | 591.57 | 915.44 | 1887.82 | (1, 3) |
| [233536_at](https://www.affymetrix.com/LinkServlet?probeset=233536_at) | [ASXL3](http://www.ncbi.nlm.nih.gov/entrez/query.fcgi?cmd=search&db=gene&term=ASXL3) | additional sex combs like 3 (Drosophila) | 0.0008103 | 0.0232 | 32 | 56.16 | 89.59 | (1, 3) |
| [221698_s_at](https://www.affymetrix.com/LinkServlet?probeset=221698_s_at) | [CLEC7A](http://www.ncbi.nlm.nih.gov/entrez/query.fcgi?cmd=search&db=gene&term=CLEC7A) | C-type lectin domain family 7, member A | 0.0008116 | 0.0232 | 215.56 | 379.42 | 134.6 | (3, 2) |
| [235501_at](https://www.affymetrix.com/LinkServlet?probeset=235501_at) | [NA](http://www.ncbi.nlm.nih.gov/entrez/query.fcgi?cmd=search&db=gene&term=NA) | NA | 0.0008124 | 0.0232 | 38.94 | 14.21 | 52.88 | (2, 3) |
| [216348_at](https://www.affymetrix.com/LinkServlet?probeset=216348_at) | [RPS17P5](http://www.ncbi.nlm.nih.gov/entrez/query.fcgi?cmd=search&db=gene&term=RPS17P5) | ribosomal protein S17 pseudogene 5 | 0.0008146 | 0.0232 | 416.72 | 524.87 | 338.54 | (3, 2) |
| [213592_at](https://www.affymetrix.com/LinkServlet?probeset=213592_at) | [APLNR](http://www.ncbi.nlm.nih.gov/entrez/query.fcgi?cmd=search&db=gene&term=APLNR) | apelin receptor | 0.0008147 | 0.0232 | 507.69 | 763.27 | 2679.96 | (1, 3), (2, 3) |
| [213174_at](https://www.affymetrix.com/LinkServlet?probeset=213174_at) | [TTC9](http://www.ncbi.nlm.nih.gov/entrez/query.fcgi?cmd=search&db=gene&term=TTC9) | tetratricopeptide repeat domain 9 | 0.0008156 | 0.0232 | 82.09 | 155.72 | 61.98 | (3, 2) |
| [207554_x_at](https://www.affymetrix.com/LinkServlet?probeset=207554_x_at) | [TBXA2R](http://www.ncbi.nlm.nih.gov/entrez/query.fcgi?cmd=search&db=gene&term=TBXA2R) | thromboxane A2 receptor | 0.0008162 | 0.0232 | 51.71 | 42.58 | 29.79 | (3, 1) |
| [1554251_at](https://www.affymetrix.com/LinkServlet?probeset=1554251_at) | [HP1BP3](http://www.ncbi.nlm.nih.gov/entrez/query.fcgi?cmd=search&db=gene&term=HP1BP3) | heterochromatin protein 1, binding protein 3 | 0.0008213 | 0.0233 | 107.8 | 163.15 | 218.55 | (1, 3) |
| [1569661_at](https://www.affymetrix.com/LinkServlet?probeset=1569661_at) | [NA](http://www.ncbi.nlm.nih.gov/entrez/query.fcgi?cmd=search&db=gene&term=NA) | NA | 0.0008229 | 0.0233 | 46.49 | 76.49 | 155.14 | (1, 3) |
| [207655_s_at](https://www.affymetrix.com/LinkServlet?probeset=207655_s_at) | [BLNK](http://www.ncbi.nlm.nih.gov/entrez/query.fcgi?cmd=search&db=gene&term=BLNK) | B-cell linker | 0.0008237 | 0.0233 | 307.46 | 522.8 | 227.21 | (3, 2) |
| [201944_at](https://www.affymetrix.com/LinkServlet?probeset=201944_at) | [HEXB](http://www.ncbi.nlm.nih.gov/entrez/query.fcgi?cmd=search&db=gene&term=HEXB) | hexosaminidase B (beta polypeptide) | 0.0008251 | 0.0233 | 1565.14 | 1884.72 | 1044.29 | (3, 2) |
| [203181_x_at](https://www.affymetrix.com/LinkServlet?probeset=203181_x_at) | [SRPK2](http://www.ncbi.nlm.nih.gov/entrez/query.fcgi?cmd=search&db=gene&term=SRPK2) | SFRS protein kinase 2 | 0.0008294 | 0.0234 | 2056.35 | 1206.68 | 1673.65 | (2, 1), (2, 3) |
| [242292_at](https://www.affymetrix.com/LinkServlet?probeset=242292_at) | [CXorf50B](http://www.ncbi.nlm.nih.gov/entrez/query.fcgi?cmd=search&db=gene&term=CXorf50B) | chromosome X open reading frame 50B | 0.0008331 | 0.0235 | 15.06 | 43.52 | 48.5 | (1, 2), (1, 3) |
| [235292_at](https://www.affymetrix.com/LinkServlet?probeset=235292_at) | [FLJ32255](http://www.ncbi.nlm.nih.gov/entrez/query.fcgi?cmd=search&db=gene&term=FLJ32255) | hypothetical protein LOC643977 | 0.0008349 | 0.0235 | 8.71 | 9.39 | 6.22 | (3, 1), (3, 2) |
| [212762_s_at](https://www.affymetrix.com/LinkServlet?probeset=212762_s_at) | [TCF7L2](http://www.ncbi.nlm.nih.gov/entrez/query.fcgi?cmd=search&db=gene&term=TCF7L2) | transcription factor 7-like 2 (T-cell specific, HMG-box) | 0.0008385 | 0.0236 | 69.75 | 41.91 | 101.01 | (2, 3) |
| [238455_at](https://www.affymetrix.com/LinkServlet?probeset=238455_at) | [NA](http://www.ncbi.nlm.nih.gov/entrez/query.fcgi?cmd=search&db=gene&term=NA) | NA | 0.0008414 | 0.0236 | 124.2 | 169.13 | 56.95 | (3, 2) |
| [206401_s_at](https://www.affymetrix.com/LinkServlet?probeset=206401_s_at) | [MAPT](http://www.ncbi.nlm.nih.gov/entrez/query.fcgi?cmd=search&db=gene&term=MAPT) | microtubule-associated protein tau | 0.0008447 | 0.0237 | 156.86 | 136.35 | 308.28 | (1, 3), (2, 3) |
| [238615_at](https://www.affymetrix.com/LinkServlet?probeset=238615_at) | [ERLIN2](http://www.ncbi.nlm.nih.gov/entrez/query.fcgi?cmd=search&db=gene&term=ERLIN2) | ER lipid raft associated 2 | 0.0008487 | 0.0238 | 22.71 | 32.45 | 20.21 | (3, 2) |
| [234472_at](https://www.affymetrix.com/LinkServlet?probeset=234472_at) | [GALNT13](http://www.ncbi.nlm.nih.gov/entrez/query.fcgi?cmd=search&db=gene&term=GALNT13) | UDP-N-acetyl-alpha-D-galactosamine:polypeptide N-acetylgalactosaminyltransferase 13 (GalNAc-T13) | 0.0008494 | 0.0238 | 43.32 | 141.88 | 212.17 | (1, 3) |
| [230259_at](https://www.affymetrix.com/LinkServlet?probeset=230259_at) | [C10orf125](http://www.ncbi.nlm.nih.gov/entrez/query.fcgi?cmd=search&db=gene&term=C10orf125) | chromosome 10 open reading frame 125 | 0.0008499 | 0.0238 | 17.17 | 25.4 | 11.53 | (3, 2) |
| [235433_at](https://www.affymetrix.com/LinkServlet?probeset=235433_at) | [APOOL](http://www.ncbi.nlm.nih.gov/entrez/query.fcgi?cmd=search&db=gene&term=APOOL) | apolipoprotein O-like | 0.0008533 | 0.0238 | 310.43 | 177.84 | 249 | (2, 1), (2, 3) |
| [222833_at](https://www.affymetrix.com/LinkServlet?probeset=222833_at) | [LPCAT2](http://www.ncbi.nlm.nih.gov/entrez/query.fcgi?cmd=search&db=gene&term=LPCAT2) | lysophosphatidylcholine acyltransferase 2 | 0.0008543 | 0.0238 | 57.94 | 80.98 | 41.19 | (3, 2) |
| [223075_s_at](https://www.affymetrix.com/LinkServlet?probeset=223075_s_at) | [AIF1L](http://www.ncbi.nlm.nih.gov/entrez/query.fcgi?cmd=search&db=gene&term=AIF1L) | allograft inflammatory factor 1-like | 0.0008613 | 0.024 | 1822.54 | 1063.52 | 673.73 | (3, 1) |
| [202868_s_at](https://www.affymetrix.com/LinkServlet?probeset=202868_s_at) | [POP4](http://www.ncbi.nlm.nih.gov/entrez/query.fcgi?cmd=search&db=gene&term=POP4) | processing of precursor 4, ribonuclease P/MRP subunit (S. cerevisiae) | 0.000863 | 0.024 | 595.95 | 744.78 | 484.49 | (3, 2) |
| [212495_at](https://www.affymetrix.com/LinkServlet?probeset=212495_at) | [KDM4B](http://www.ncbi.nlm.nih.gov/entrez/query.fcgi?cmd=search&db=gene&term=KDM4B) | lysine (K)-specific demethylase 4B | 0.0008631 | 0.024 | 111.55 | 76.38 | 126.08 | (2, 3) |
| [203928_x_at](https://www.affymetrix.com/LinkServlet?probeset=203928_x_at) | [MAPT](http://www.ncbi.nlm.nih.gov/entrez/query.fcgi?cmd=search&db=gene&term=MAPT) | microtubule-associated protein tau | 0.0008648 | 0.024 | 196.62 | 190.33 | 393.93 | (1, 3), (2, 3) |
| [241344_at](https://www.affymetrix.com/LinkServlet?probeset=241344_at) | [NA](http://www.ncbi.nlm.nih.gov/entrez/query.fcgi?cmd=search&db=gene&term=NA) | NA | 0.0008665 | 0.024 | 117.57 | 137.87 | 181.16 | (1, 3) |
| [241804_at](https://www.affymetrix.com/LinkServlet?probeset=241804_at) | [NA](http://www.ncbi.nlm.nih.gov/entrez/query.fcgi?cmd=search&db=gene&term=NA) | NA | 0.0008682 | 0.024 | 19.05 | 51.58 | 42.97 | (1, 2), (1, 3) |
| [211100_x_at](https://www.affymetrix.com/LinkServlet?probeset=211100_x_at) | [LILRA2](http://www.ncbi.nlm.nih.gov/entrez/query.fcgi?cmd=search&db=gene&term=LILRA2) | leukocyte immunoglobulin-like receptor, subfamily A (with TM domain), member 2 | 0.00087 | 0.024 | 13.52 | 17.37 | 10.53 | (3, 2) |
| [205640_at](https://www.affymetrix.com/LinkServlet?probeset=205640_at) | [ALDH3B1](http://www.ncbi.nlm.nih.gov/entrez/query.fcgi?cmd=search&db=gene&term=ALDH3B1) | aldehyde dehydrogenase 3 family, member B1 | 0.0008713 | 0.024 | 49.66 | 24.42 | 18.56 | (3, 1) |
| [218019_s_at](https://www.affymetrix.com/LinkServlet?probeset=218019_s_at) | [PDXK](http://www.ncbi.nlm.nih.gov/entrez/query.fcgi?cmd=search&db=gene&term=PDXK) | pyridoxal (pyridoxine, vitamin B6) kinase | 0.000874 | 0.0241 | 480.11 | 247.37 | 337.31 | (2, 1) |
| [228641_at](https://www.affymetrix.com/LinkServlet?probeset=228641_at) | [CARD8](http://www.ncbi.nlm.nih.gov/entrez/query.fcgi?cmd=search&db=gene&term=CARD8) | caspase recruitment domain family, member 8 | 0.0008773 | 0.0241 | 53.5 | 88.01 | 50.05 | (1, 2), (3, 2) |
| [239082_at](https://www.affymetrix.com/LinkServlet?probeset=239082_at) | [NA](http://www.ncbi.nlm.nih.gov/entrez/query.fcgi?cmd=search&db=gene&term=NA) | NA | 0.0008775 | 0.0241 | 452.37 | 368.16 | 653.68 | (2, 3) |
| [201954_at](https://www.affymetrix.com/LinkServlet?probeset=201954_at) | [ARPC1B](http://www.ncbi.nlm.nih.gov/entrez/query.fcgi?cmd=search&db=gene&term=ARPC1B) | actin related protein 2/3 complex, subunit 1B, 41kDa | 0.0008777 | 0.0241 | 1598.47 | 1299.87 | 685.69 | (3, 1), (3, 2) |
| [1561856_at](https://www.affymetrix.com/LinkServlet?probeset=1561856_at) | [NA](http://www.ncbi.nlm.nih.gov/entrez/query.fcgi?cmd=search&db=gene&term=NA) | NA | 0.0008779 | 0.0241 | 17.93 | 8.04 | 34.16 | (2, 3) |
| [213137_s_at](https://www.affymetrix.com/LinkServlet?probeset=213137_s_at) | [PTPN2](http://www.ncbi.nlm.nih.gov/entrez/query.fcgi?cmd=search&db=gene&term=PTPN2) | protein tyrosine phosphatase, non-receptor type 2 | 0.0008815 | 0.0241 | 81.09 | 134.06 | 88.59 | (1, 2), (3, 2) |
| [228568_at](https://www.affymetrix.com/LinkServlet?probeset=228568_at) | [GCOM1](http://www.ncbi.nlm.nih.gov/entrez/query.fcgi?cmd=search&db=gene&term=GCOM1) | GRINL1A complex locus | 0.0008824 | 0.0241 | 22.28 | 86.89 | 95.99 | (1, 2), (1, 3) |
| [218654_s_at](https://www.affymetrix.com/LinkServlet?probeset=218654_s_at) | [MRPS33](http://www.ncbi.nlm.nih.gov/entrez/query.fcgi?cmd=search&db=gene&term=MRPS33) | mitochondrial ribosomal protein S33 | 0.0008857 | 0.0242 | 963.88 | 880.57 | 622.06 | (3, 1), (3, 2) |
| [213982_s_at](https://www.affymetrix.com/LinkServlet?probeset=213982_s_at) | [RABGAP1L](http://www.ncbi.nlm.nih.gov/entrez/query.fcgi?cmd=search&db=gene&term=RABGAP1L) | RAB GTPase activating protein 1-like | 0.0008881 | 0.0242 | 202.34 | 326.08 | 166.34 | (3, 2) |
| [224693_at](https://www.affymetrix.com/LinkServlet?probeset=224693_at) | [C20orf108](http://www.ncbi.nlm.nih.gov/entrez/query.fcgi?cmd=search&db=gene&term=C20orf108) | chromosome 20 open reading frame 108 | 0.0008896 | 0.0242 | 101.38 | 67.95 | 50.64 | (3, 1) |
| [206381_at](https://www.affymetrix.com/LinkServlet?probeset=206381_at) | [SCN2A](http://www.ncbi.nlm.nih.gov/entrez/query.fcgi?cmd=search&db=gene&term=SCN2A) | sodium channel, voltage-gated, type II, alpha subunit | 0.0008916 | 0.0243 | 79.91 | 150.33 | 252.86 | (1, 3) |
| [215115_x_at](https://www.affymetrix.com/LinkServlet?probeset=215115_x_at) | [NTRK3](http://www.ncbi.nlm.nih.gov/entrez/query.fcgi?cmd=search&db=gene&term=NTRK3) | neurotrophic tyrosine kinase, receptor, type 3 | 0.0008923 | 0.0243 | 124.63 | 150.66 | 313.12 | (1, 3), (2, 3) |
| [220272_at](https://www.affymetrix.com/LinkServlet?probeset=220272_at) | [BNC2](http://www.ncbi.nlm.nih.gov/entrez/query.fcgi?cmd=search&db=gene&term=BNC2) | basonuclin 2 | 0.0008999 | 0.0244 | 14.15 | 11 | 5.92 | (3, 1), (3, 2) |
| [233668_at](https://www.affymetrix.com/LinkServlet?probeset=233668_at) | [NA](http://www.ncbi.nlm.nih.gov/entrez/query.fcgi?cmd=search&db=gene&term=NA) | NA | 0.0009001 | 0.0244 | 6.79 | 6.51 | 13.94 | (1, 3), (2, 3) |
| [226435_at](https://www.affymetrix.com/LinkServlet?probeset=226435_at) | [PAPLN](http://www.ncbi.nlm.nih.gov/entrez/query.fcgi?cmd=search&db=gene&term=PAPLN) | papilin, proteoglycan-like sulfated glycoprotein | 0.0009005 | 0.0244 | 51.68 | 75.15 | 19.8 | (3, 2) |
| [221002_s_at](https://www.affymetrix.com/LinkServlet?probeset=221002_s_at) | [TSPAN14](http://www.ncbi.nlm.nih.gov/entrez/query.fcgi?cmd=search&db=gene&term=TSPAN14) | tetraspanin 14 | 0.0009025 | 0.0244 | 152.88 | 151.31 | 96.86 | (3, 1), (3, 2) |
| [226337_at](https://www.affymetrix.com/LinkServlet?probeset=226337_at) | [GORAB](http://www.ncbi.nlm.nih.gov/entrez/query.fcgi?cmd=search&db=gene&term=GORAB) | golgin, RAB6-interacting | 0.0009028 | 0.0244 | 166.59 | 323.22 | 230.45 | (1, 2) |
| [203389_at](https://www.affymetrix.com/LinkServlet?probeset=203389_at) | [KIF3C](http://www.ncbi.nlm.nih.gov/entrez/query.fcgi?cmd=search&db=gene&term=KIF3C) | kinesin family member 3C | 0.0009035 | 0.0244 | 166.21 | 105.85 | 182.58 | (2, 3) |
| [1556462_a_at](https://www.affymetrix.com/LinkServlet?probeset=1556462_a_at) | [NA](http://www.ncbi.nlm.nih.gov/entrez/query.fcgi?cmd=search&db=gene&term=NA) | NA | 0.0009042 | 0.0244 | 10.67 | 12.8 | 26.74 | (1, 3), (2, 3) |
| [210292_s_at](https://www.affymetrix.com/LinkServlet?probeset=210292_s_at) | [NA](http://www.ncbi.nlm.nih.gov/entrez/query.fcgi?cmd=search&db=gene&term=NA) | NA | 0.0009079 | 0.0244 | 26.13 | 12.03 | 64.48 | (2, 3) |
| [239417_x_at](https://www.affymetrix.com/LinkServlet?probeset=239417_x_at) | [C6orf52](http://www.ncbi.nlm.nih.gov/entrez/query.fcgi?cmd=search&db=gene&term=C6orf52) | chromosome 6 open reading frame 52 | 0.0009184 | 0.0247 | 13.6 | 12.33 | 7.9 | (3, 1), (3, 2) |
| [235112_at](https://www.affymetrix.com/LinkServlet?probeset=235112_at) | [NA](http://www.ncbi.nlm.nih.gov/entrez/query.fcgi?cmd=search&db=gene&term=NA) | NA | 0.0009239 | 0.0248 | 212.37 | 201.92 | 400.35 | (1, 3), (2, 3) |
| [235061_at](https://www.affymetrix.com/LinkServlet?probeset=235061_at) | [PPM1K](http://www.ncbi.nlm.nih.gov/entrez/query.fcgi?cmd=search&db=gene&term=PPM1K) | protein phosphatase 1K (PP2C domain containing) | 0.000924 | 0.0248 | 373.81 | 328.94 | 611.35 | (1, 3), (2, 3) |
| [230781_at](https://www.affymetrix.com/LinkServlet?probeset=230781_at) | [NA](http://www.ncbi.nlm.nih.gov/entrez/query.fcgi?cmd=search&db=gene&term=NA) | NA | 0.0009241 | 0.0248 | 28.68 | 126.27 | 221.6 | (1, 3) |
| [222216_s_at](https://www.affymetrix.com/LinkServlet?probeset=222216_s_at) | [MRPL17](http://www.ncbi.nlm.nih.gov/entrez/query.fcgi?cmd=search&db=gene&term=MRPL17) | mitochondrial ribosomal protein L17 | 0.0009268 | 0.0248 | 696.09 | 716.77 | 484.69 | (3, 1), (3, 2) |
| [231103_at](https://www.affymetrix.com/LinkServlet?probeset=231103_at) | [NA](http://www.ncbi.nlm.nih.gov/entrez/query.fcgi?cmd=search&db=gene&term=NA) | NA | 0.0009271 | 0.0248 | 30.02 | 32.75 | 109.65 | (1, 3), (2, 3) |
| [244327_at](https://www.affymetrix.com/LinkServlet?probeset=244327_at) | [NA](http://www.ncbi.nlm.nih.gov/entrez/query.fcgi?cmd=search&db=gene&term=NA) | NA | 0.0009311 | 0.0248 | 10.54 | 20.84 | 35.85 | (1, 3) |
| [219143_s_at](https://www.affymetrix.com/LinkServlet?probeset=219143_s_at) | [RPP25](http://www.ncbi.nlm.nih.gov/entrez/query.fcgi?cmd=search&db=gene&term=RPP25) | ribonuclease P/MRP 25kDa subunit | 0.000932 | 0.0248 | 9.63 | 16.4 | 8.36 | (3, 2) |
| [207869_s_at](https://www.affymetrix.com/LinkServlet?probeset=207869_s_at) | [CACNA1G](http://www.ncbi.nlm.nih.gov/entrez/query.fcgi?cmd=search&db=gene&term=CACNA1G) | calcium channel, voltage-dependent, T type, alpha 1G subunit | 0.000935 | 0.0249 | 38.1 | 19.78 | 71.66 | (2, 3) |
| [1562733_at](https://www.affymetrix.com/LinkServlet?probeset=1562733_at) | [NCRNA00092](http://www.ncbi.nlm.nih.gov/entrez/query.fcgi?cmd=search&db=gene&term=NCRNA00092) | non-protein coding RNA 92 | 0.0009362 | 0.0249 | 20.98 | 26.1 | 38.57 | (1, 3) |
| [217033_x_at](https://www.affymetrix.com/LinkServlet?probeset=217033_x_at) | [NTRK3](http://www.ncbi.nlm.nih.gov/entrez/query.fcgi?cmd=search&db=gene&term=NTRK3) | neurotrophic tyrosine kinase, receptor, type 3 | 0.0009382 | 0.0249 | 90.41 | 117.69 | 242.19 | (1, 3), (2, 3) |
| [213094_at](https://www.affymetrix.com/LinkServlet?probeset=213094_at) | [GPR126](http://www.ncbi.nlm.nih.gov/entrez/query.fcgi?cmd=search&db=gene&term=GPR126) | G protein-coupled receptor 126 | 0.0009407 | 0.0249 | 57 | 32.24 | 14.7 | (3, 1) |
| [224196_x_at](https://www.affymetrix.com/LinkServlet?probeset=224196_x_at) | [DPH5](http://www.ncbi.nlm.nih.gov/entrez/query.fcgi?cmd=search&db=gene&term=DPH5) | DPH5 homolog (S. cerevisiae) | 0.0009415 | 0.0249 | 138.93 | 191.34 | 124.65 | (3, 2) |
| [243713_at](https://www.affymetrix.com/LinkServlet?probeset=243713_at) | [NA](http://www.ncbi.nlm.nih.gov/entrez/query.fcgi?cmd=search&db=gene&term=NA) | NA | 0.0009426 | 0.0249 | 9.77 | 7.57 | 22.1 | (2, 3) |
| [219451_at](https://www.affymetrix.com/LinkServlet?probeset=219451_at) | [MSRB2](http://www.ncbi.nlm.nih.gov/entrez/query.fcgi?cmd=search&db=gene&term=MSRB2) | methionine sulfoxide reductase B2 | 0.0009427 | 0.0249 | 491.83 | 393.98 | 643.43 | (2, 3) |
| [228811_at](https://www.affymetrix.com/LinkServlet?probeset=228811_at) | [NA](http://www.ncbi.nlm.nih.gov/entrez/query.fcgi?cmd=search&db=gene&term=NA) | NA | 0.0009429 | 0.0249 | 65.72 | 45.18 | 74.36 | (2, 3) |
| [221588_x_at](https://www.affymetrix.com/LinkServlet?probeset=221588_x_at) | [ALDH6A1](http://www.ncbi.nlm.nih.gov/entrez/query.fcgi?cmd=search&db=gene&term=ALDH6A1) | aldehyde dehydrogenase 6 family, member A1 | 0.000943 | 0.0249 | 648.04 | 434.89 | 776.7 | (2, 3) |
| [213922_at](https://www.affymetrix.com/LinkServlet?probeset=213922_at) | [TTBK2](http://www.ncbi.nlm.nih.gov/entrez/query.fcgi?cmd=search&db=gene&term=TTBK2) | tau tubulin kinase 2 | 0.0009459 | 0.0249 | 221.28 | 213.23 | 378.89 | (1, 3), (2, 3) |
| [213511_s_at](https://www.affymetrix.com/LinkServlet?probeset=213511_s_at) | [MTMR1](http://www.ncbi.nlm.nih.gov/entrez/query.fcgi?cmd=search&db=gene&term=MTMR1) | myotubularin related protein 1 | 0.0009535 | 0.0251 | 307.86 | 380.25 | 499.59 | (1, 3) |
| [226133_s_at](https://www.affymetrix.com/LinkServlet?probeset=226133_s_at) | [TBC1D10A](http://www.ncbi.nlm.nih.gov/entrez/query.fcgi?cmd=search&db=gene&term=TBC1D10A) | TBC1 domain family, member 10A | 0.0009569 | 0.0251 | 105.07 | 69 | 56.99 | (3, 1) |
| [209618_at](https://www.affymetrix.com/LinkServlet?probeset=209618_at) | [CTNND2](http://www.ncbi.nlm.nih.gov/entrez/query.fcgi?cmd=search&db=gene&term=CTNND2) | catenin (cadherin-associated protein), delta 2 (neural plakophilin-related arm-repeat protein) | 0.0009575 | 0.0251 | 1418.5 | 873.92 | 1708.32 | (2, 3) |
| [242064_at](https://www.affymetrix.com/LinkServlet?probeset=242064_at) | [SDK2](http://www.ncbi.nlm.nih.gov/entrez/query.fcgi?cmd=search&db=gene&term=SDK2) | sidekick homolog 2 (chicken) | 0.0009602 | 0.0252 | 106.3 | 25.34 | 101.73 | (2, 1), (2, 3) |
| [208300_at](https://www.affymetrix.com/LinkServlet?probeset=208300_at) | [PTPRH](http://www.ncbi.nlm.nih.gov/entrez/query.fcgi?cmd=search&db=gene&term=PTPRH) | protein tyrosine phosphatase, receptor type, H | 0.0009638 | 0.0252 | 8.36 | 10.53 | 5.32 | (3, 2) |
| [206081_at](https://www.affymetrix.com/LinkServlet?probeset=206081_at) | [SLC24A1](http://www.ncbi.nlm.nih.gov/entrez/query.fcgi?cmd=search&db=gene&term=SLC24A1) | solute carrier family 24 (sodium/potassium/calcium exchanger), member 1 | 0.0009656 | 0.0252 | 118.52 | 91.01 | 64.45 | (3, 1) |
| [207677_s_at](https://www.affymetrix.com/LinkServlet?probeset=207677_s_at) | [NCF4](http://www.ncbi.nlm.nih.gov/entrez/query.fcgi?cmd=search&db=gene&term=NCF4) | neutrophil cytosolic factor 4, 40kDa | 0.0009662 | 0.0252 | 42.75 | 72 | 26.27 | (3, 2) |
| [229367_s_at](https://www.affymetrix.com/LinkServlet?probeset=229367_s_at) | [GIMAP6](http://www.ncbi.nlm.nih.gov/entrez/query.fcgi?cmd=search&db=gene&term=GIMAP6) | GTPase, IMAP family member 6 | 0.0009702 | 0.0252 | 176.13 | 156.44 | 81.27 | (3, 1), (3, 2) |
| [238327_at](https://www.affymetrix.com/LinkServlet?probeset=238327_at) | [ODF3B](http://www.ncbi.nlm.nih.gov/entrez/query.fcgi?cmd=search&db=gene&term=ODF3B) | outer dense fiber of sperm tails 3B | 0.0009706 | 0.0252 | 21.93 | 42.59 | 22.18 | (1, 2), (3, 2) |
| [213515_x_at](https://www.affymetrix.com/LinkServlet?probeset=213515_x_at) | [NA](http://www.ncbi.nlm.nih.gov/entrez/query.fcgi?cmd=search&db=gene&term=NA) | NA | 0.0009726 | 0.0252 | 33.13 | 83.03 | 17.93 | (3, 2) |
| [203857_s_at](https://www.affymetrix.com/LinkServlet?probeset=203857_s_at) | [PDIA5](http://www.ncbi.nlm.nih.gov/entrez/query.fcgi?cmd=search&db=gene&term=PDIA5) | protein disulfide isomerase family A, member 5 | 0.0009751 | 0.0252 | 131.39 | 123.52 | 54.28 | (3, 1), (3, 2) |
| [215314_at](https://www.affymetrix.com/LinkServlet?probeset=215314_at) | [ANK3](http://www.ncbi.nlm.nih.gov/entrez/query.fcgi?cmd=search&db=gene&term=ANK3) | ankyrin 3, node of Ranvier (ankyrin G) | 0.0009753 | 0.0252 | 30.08 | 57.9 | 117.89 | (1, 3) |
| [205194_at](https://www.affymetrix.com/LinkServlet?probeset=205194_at) | [PSPH](http://www.ncbi.nlm.nih.gov/entrez/query.fcgi?cmd=search&db=gene&term=PSPH) | phosphoserine phosphatase | 0.0009754 | 0.0252 | 226.84 | 226.03 | 120.34 | (3, 1), (3, 2) |
| [230863_at](https://www.affymetrix.com/LinkServlet?probeset=230863_at) | [LRP2](http://www.ncbi.nlm.nih.gov/entrez/query.fcgi?cmd=search&db=gene&term=LRP2) | low density lipoprotein-related protein 2 | 0.0009755 | 0.0252 | 28.49 | 8.31 | 8.3 | (2, 1), (3, 1) |
| [204681_s_at](https://www.affymetrix.com/LinkServlet?probeset=204681_s_at) | [RAPGEF5](http://www.ncbi.nlm.nih.gov/entrez/query.fcgi?cmd=search&db=gene&term=RAPGEF5) | Rap guanine nucleotide exchange factor (GEF) 5 | 0.0009756 | 0.0252 | 372.41 | 88.05 | 89.51 | (2, 1), (3, 1) |
| [224925_at](https://www.affymetrix.com/LinkServlet?probeset=224925_at) | [PREX1](http://www.ncbi.nlm.nih.gov/entrez/query.fcgi?cmd=search&db=gene&term=PREX1) | phosphatidylinositol-3,4,5-trisphosphate-dependent Rac exchange factor 1 | 0.0009797 | 0.0253 | 799.65 | 765.32 | 1126.06 | (1, 3), (2, 3) |
| [205139_s_at](https://www.affymetrix.com/LinkServlet?probeset=205139_s_at) | [UST](http://www.ncbi.nlm.nih.gov/entrez/query.fcgi?cmd=search&db=gene&term=UST) | uronyl-2-sulfotransferase | 0.0009824 | 0.0253 | 560.9 | 705.26 | 1343.07 | (1, 3), (2, 3) |
| [1554821_a_at](https://www.affymetrix.com/LinkServlet?probeset=1554821_a_at) | [ZBED1](http://www.ncbi.nlm.nih.gov/entrez/query.fcgi?cmd=search&db=gene&term=ZBED1) | zinc finger, BED-type containing 1 | 0.0009839 | 0.0253 | 97.86 | 76.4 | 161.83 | (2, 3) |
| [219687_at](https://www.affymetrix.com/LinkServlet?probeset=219687_at) | [HHAT](http://www.ncbi.nlm.nih.gov/entrez/query.fcgi?cmd=search&db=gene&term=HHAT) | hedgehog acyltransferase | 0.0009842 | 0.0253 | 19.54 | 10.99 | 15.53 | (2, 1), (2, 3) |
| [229984_at](https://www.affymetrix.com/LinkServlet?probeset=229984_at) | [DTWD1](http://www.ncbi.nlm.nih.gov/entrez/query.fcgi?cmd=search&db=gene&term=DTWD1) | DTW domain containing 1 | 0.0009848 | 0.0253 | 51.25 | 57.23 | 34.44 | (3, 2) |
| [226243_at](https://www.affymetrix.com/LinkServlet?probeset=226243_at) | [C2orf79](http://www.ncbi.nlm.nih.gov/entrez/query.fcgi?cmd=search&db=gene&term=C2orf79) | chromosome 2 open reading frame 79 | 0.0009866 | 0.0253 | 663.73 | 871.71 | 565.54 | (3, 2) |
| [219416_at](https://www.affymetrix.com/LinkServlet?probeset=219416_at) | [SCARA3](http://www.ncbi.nlm.nih.gov/entrez/query.fcgi?cmd=search&db=gene&term=SCARA3) | scavenger receptor class A, member 3 | 0.0009869 | 0.0253 | 40.16 | 34.11 | 90.92 | (1, 3), (2, 3) |
| [1554930_a_at](https://www.affymetrix.com/LinkServlet?probeset=1554930_a_at) | [FUT8](http://www.ncbi.nlm.nih.gov/entrez/query.fcgi?cmd=search&db=gene&term=FUT8) | fucosyltransferase 8 (alpha (1,6) fucosyltransferase) | 0.0009875 | 0.0253 | 39.2 | 20.15 | 19.64 | (2, 1), (3, 1) |
| [225383_at](https://www.affymetrix.com/LinkServlet?probeset=225383_at) | [ZNF275](http://www.ncbi.nlm.nih.gov/entrez/query.fcgi?cmd=search&db=gene&term=ZNF275) | zinc finger protein 275 | 0.0009903 | 0.0253 | 371.68 | 250.59 | 480.12 | (2, 3) |
| [225270_at](https://www.affymetrix.com/LinkServlet?probeset=225270_at) | [NEO1](http://www.ncbi.nlm.nih.gov/entrez/query.fcgi?cmd=search&db=gene&term=NEO1) | neogenin homolog 1 (chicken) | 0.0009905 | 0.0253 | 194.59 | 167.72 | 278.1 | (2, 3) |
| [205903_s_at](https://www.affymetrix.com/LinkServlet?probeset=205903_s_at) | [KCNN3](http://www.ncbi.nlm.nih.gov/entrez/query.fcgi?cmd=search&db=gene&term=KCNN3) | potassium intermediate/small conductance calcium-activated channel, subfamily N, member 3 | 0.0009907 | 0.0253 | 32.17 | 25.47 | 86.91 | (1, 3), (2, 3) |
| [238002_at](https://www.affymetrix.com/LinkServlet?probeset=238002_at) | [NA](http://www.ncbi.nlm.nih.gov/entrez/query.fcgi?cmd=search&db=gene&term=NA) | NA | 0.0009909 | 0.0253 | 2617.2 | 3875.53 | 4147.26 | (1, 2), (1, 3) |
| [41644_at](https://www.affymetrix.com/LinkServlet?probeset=41644_at) | [SASH1](http://www.ncbi.nlm.nih.gov/entrez/query.fcgi?cmd=search&db=gene&term=SASH1) | SAM and SH3 domain containing 1 | 0.0009926 | 0.0253 | 503.67 | 326.46 | 736.47 | (2, 3) |
| [209107_x_at](https://www.affymetrix.com/LinkServlet?probeset=209107_x_at) | [NCOA1](http://www.ncbi.nlm.nih.gov/entrez/query.fcgi?cmd=search&db=gene&term=NCOA1) | nuclear receptor coactivator 1 | 0.0009971 | 0.0254 | 698.47 | 620.42 | 989.42 | (2, 3) |
| [238350_at](https://www.affymetrix.com/LinkServlet?probeset=238350_at) | [UBN2](http://www.ncbi.nlm.nih.gov/entrez/query.fcgi?cmd=search&db=gene&term=UBN2) | ubinuclein 2 | 0.0009982 | 0.0254 | 31.8 | 27.56 | 60.66 | (1, 3), (2, 3) |
