# Supplementary material for: Transcriptional profiles of pilocytic astrocytoma are related to their three different locations, but not to radiological tumor features
Source: BMC Cancer. 2015 Oct 24;15:778. doi: 10.1186/s12885-015-1810-z (PMC4619381; doi:10.1186/s12885-015-1810-z)
Supplement: Additional file 4: Table S4. — List of genes differentiating between pilocytic astrocytomas in the three most frequently observed locations. Comparison of all supratentorial and infratentorial tumors. M1/ M2, supratentorial tumors; M3, infratentorial tumors. The microarray data discussed in this publication have been deposited in the NCBI’s Gene Expression Omnibus. The accession number is GSE73066 and the link to freely access is is http://www.ncbi.nlm.nih.gov/geo/query/acc.cgi?acc=GSE73066 (DOCX 72 kb) [file 12885_2015_1810_MOESM4_ESM.docx]

| **Probe set** | **Gene symbol** | **Description** | | **Parametric *p*-value** | **Geom mean of intensities**  **in class M1/M2** | **Geom mean of intensities in class**  **M3** | **Fold-change** |
| --- | --- | --- | --- | --- | --- | --- | --- |
| [231666_at](https://www.affymetrix.com/LinkServlet?probeset=231666_at) | [PAX3](http://www.ncbi.nlm.nih.gov/entrez/query.fcgi?cmd=search&db=gene&term=PAX3) | | paired box 3 | < 1e-07 | 6.28 | 279.28 | 0.022 |
| [228462_at](https://www.affymetrix.com/LinkServlet?probeset=228462_at) | [IRX2](http://www.ncbi.nlm.nih.gov/entrez/query.fcgi?cmd=search&db=gene&term=IRX2) | | iroquois homeobox 2 | < 1e-07 | 18.18 | 470.74 | 0.039 |
| [206140_at](https://www.affymetrix.com/LinkServlet?probeset=206140_at) | [LHX2](http://www.ncbi.nlm.nih.gov/entrez/query.fcgi?cmd=search&db=gene&term=LHX2) | | LIM homeobox 2 | < 1e-07 | 2155.8 | 11.02 | 195.66 |
| [211219_s_at](https://www.affymetrix.com/LinkServlet?probeset=211219_s_at) | [LHX2](http://www.ncbi.nlm.nih.gov/entrez/query.fcgi?cmd=search&db=gene&term=LHX2) | | LIM homeobox 2 | < 1e-07 | 87.43 | 5.49 | 15.93 |
| [238727_at](https://www.affymetrix.com/LinkServlet?probeset=238727_at) | [LOC440934](http://www.ncbi.nlm.nih.gov/entrez/query.fcgi?cmd=search&db=gene&term=LOC440934) | | hypothetical LOC440934 | < 1e-07 | 18.27 | 258.93 | 0.071 |
| [223582_at](https://www.affymetrix.com/LinkServlet?probeset=223582_at) | [GPR98](http://www.ncbi.nlm.nih.gov/entrez/query.fcgi?cmd=search&db=gene&term=GPR98) | | G protein-coupled receptor 98 | < 1e-07 | 91.01 | 15.08 | 6.03 |
| [1554784_at](https://www.affymetrix.com/LinkServlet?probeset=1554784_at) | [CNTN1](http://www.ncbi.nlm.nih.gov/entrez/query.fcgi?cmd=search&db=gene&term=CNTN1) | | contactin 1 | < 1e-07 | 32.38 | 379.96 | 0.085 |
| [1554507_at](https://www.affymetrix.com/LinkServlet?probeset=1554507_at) | [NAALAD2](http://www.ncbi.nlm.nih.gov/entrez/query.fcgi?cmd=search&db=gene&term=NAALAD2) | | N-acetylated alpha-linked acidic dipeptidase 2 | < 1e-07 | 9.67 | 69.52 | 0.14 |
| [210239_at](https://www.affymetrix.com/LinkServlet?probeset=210239_at) | [IRX5](http://www.ncbi.nlm.nih.gov/entrez/query.fcgi?cmd=search&db=gene&term=IRX5) | | iroquois homeobox 5 | < 1e-07 | 15.13 | 100.36 | 0.15 |
| [229831_at](https://www.affymetrix.com/LinkServlet?probeset=229831_at) | [CNTN3](http://www.ncbi.nlm.nih.gov/entrez/query.fcgi?cmd=search&db=gene&term=CNTN3) | | contactin 3 (plasmacytoma associated) | < 1e-07 | 14.11 | 176.8 | 0.08 |
| [232054_at](https://www.affymetrix.com/LinkServlet?probeset=232054_at) | [PCDH20](http://www.ncbi.nlm.nih.gov/entrez/query.fcgi?cmd=search&db=gene&term=PCDH20) | | protocadherin 20 | < 1e-07 | 32.97 | 277.59 | 0.12 |
| [227202_at](https://www.affymetrix.com/LinkServlet?probeset=227202_at) | [CNTN1](http://www.ncbi.nlm.nih.gov/entrez/query.fcgi?cmd=search&db=gene&term=CNTN1) | | contactin 1 | < 1e-07 | 107.17 | 1717.72 | 0.062 |
| [243932_at](https://www.affymetrix.com/LinkServlet?probeset=243932_at) | [NA](http://www.ncbi.nlm.nih.gov/entrez/query.fcgi?cmd=search&db=gene&term=NA) | | NA | < 1e-07 | 10.74 | 60.51 | 0.18 |
| [244420_at](https://www.affymetrix.com/LinkServlet?probeset=244420_at) | [NA](http://www.ncbi.nlm.nih.gov/entrez/query.fcgi?cmd=search&db=gene&term=NA) | | NA | 1e-07 | 10.24 | 48.92 | 0.21 |
| [208464_at](https://www.affymetrix.com/LinkServlet?probeset=208464_at) | [GRIA4](http://www.ncbi.nlm.nih.gov/entrez/query.fcgi?cmd=search&db=gene&term=GRIA4) | | glutamate receptor, ionotrophic, AMPA 4 | 1e-07 | 10.4 | 41.53 | 0.25 |
| [230008_at](https://www.affymetrix.com/LinkServlet?probeset=230008_at) | [THSD7A](http://www.ncbi.nlm.nih.gov/entrez/query.fcgi?cmd=search&db=gene&term=THSD7A) | | thrombospondin, type I, domain containing 7A | 1e-07 | 66.56 | 294.74 | 0.23 |
| [1553765_a_at](https://www.affymetrix.com/LinkServlet?probeset=1553765_a_at) | [KLHL32](http://www.ncbi.nlm.nih.gov/entrez/query.fcgi?cmd=search&db=gene&term=KLHL32) | | kelch-like 32 (Drosophila) | 1e-07 | 37.55 | 234.02 | 0.16 |
| [1558508_a_at](https://www.affymetrix.com/LinkServlet?probeset=1558508_a_at) | [C1orf53](http://www.ncbi.nlm.nih.gov/entrez/query.fcgi?cmd=search&db=gene&term=C1orf53) | | chromosome 1 open reading frame 53 | 1e-07 | 43.99 | 10.9 | 4.04 |
| [224215_s_at](https://www.affymetrix.com/LinkServlet?probeset=224215_s_at) | [DLL1](http://www.ncbi.nlm.nih.gov/entrez/query.fcgi?cmd=search&db=gene&term=DLL1) | | delta-like 1 (Drosophila) | 1e-07 | 48.76 | 227.9 | 0.21 |
| [223422_s_at](https://www.affymetrix.com/LinkServlet?probeset=223422_s_at) | [ARHGAP24](http://www.ncbi.nlm.nih.gov/entrez/query.fcgi?cmd=search&db=gene&term=ARHGAP24) | | Rho GTPase activating protein 24 | 1e-07 | 204.86 | 63.56 | 3.22 |
| [214920_at](https://www.affymetrix.com/LinkServlet?probeset=214920_at) | [THSD7A](http://www.ncbi.nlm.nih.gov/entrez/query.fcgi?cmd=search&db=gene&term=THSD7A) | | thrombospondin, type I, domain containing 7A | 1e-07 | 96.64 | 404.64 | 0.24 |
| [230472_at](https://www.affymetrix.com/LinkServlet?probeset=230472_at) | [IRX1](http://www.ncbi.nlm.nih.gov/entrez/query.fcgi?cmd=search&db=gene&term=IRX1) | | iroquois homeobox 1 | 1e-07 | 29.42 | 205.19 | 0.14 |
| [230458_at](https://www.affymetrix.com/LinkServlet?probeset=230458_at) | [SLC45A1](http://www.ncbi.nlm.nih.gov/entrez/query.fcgi?cmd=search&db=gene&term=SLC45A1) | | solute carrier family 45, member 1 | 1e-07 | 30.27 | 58.66 | 0.52 |
| [205858_at](https://www.affymetrix.com/LinkServlet?probeset=205858_at) | [NGFR](http://www.ncbi.nlm.nih.gov/entrez/query.fcgi?cmd=search&db=gene&term=NGFR) | | nerve growth factor receptor (TNFR superfamily, member 16) | 2e-07 | 80.9 | 14.67 | 5.52 |
| [213894_at](https://www.affymetrix.com/LinkServlet?probeset=213894_at) | [THSD7A](http://www.ncbi.nlm.nih.gov/entrez/query.fcgi?cmd=search&db=gene&term=THSD7A) | | thrombospondin, type I, domain containing 7A | 2e-07 | 55.29 | 264.53 | 0.21 |
| [1552386_at](https://www.affymetrix.com/LinkServlet?probeset=1552386_at) | [GAPT](http://www.ncbi.nlm.nih.gov/entrez/query.fcgi?cmd=search&db=gene&term=GAPT) | | GRB2-binding adaptor protein, transmembrane | 2e-07 | 76.53 | 24.36 | 3.14 |
| [221030_s_at](https://www.affymetrix.com/LinkServlet?probeset=221030_s_at) | [ARHGAP24](http://www.ncbi.nlm.nih.gov/entrez/query.fcgi?cmd=search&db=gene&term=ARHGAP24) | | Rho GTPase activating protein 24 | 2e-07 | 38.96 | 15.59 | 2.5 |
| [235494_at](https://www.affymetrix.com/LinkServlet?probeset=235494_at) | [NA](http://www.ncbi.nlm.nih.gov/entrez/query.fcgi?cmd=search&db=gene&term=NA) | | NA | 3e-07 | 1386.62 | 2650.31 | 0.52 |
| [227209_at](https://www.affymetrix.com/LinkServlet?probeset=227209_at) | [CNTN1](http://www.ncbi.nlm.nih.gov/entrez/query.fcgi?cmd=search&db=gene&term=CNTN1) | | contactin 1 | 3e-07 | 79.89 | 933.25 | 0.086 |
| [228646_at](https://www.affymetrix.com/LinkServlet?probeset=228646_at) | [PPP1R1C](http://www.ncbi.nlm.nih.gov/entrez/query.fcgi?cmd=search&db=gene&term=PPP1R1C) | | protein phosphatase 1, regulatory (inhibitor) subunit 1C | 3e-07 | 86.17 | 17.97 | 4.8 |
| [218974_at](https://www.affymetrix.com/LinkServlet?probeset=218974_at) | [SOBP](http://www.ncbi.nlm.nih.gov/entrez/query.fcgi?cmd=search&db=gene&term=SOBP) | | sine oculis binding protein homolog (Drosophila) | 3e-07 | 1308.14 | 2529.1 | 0.52 |
| [224331_s_at](https://www.affymetrix.com/LinkServlet?probeset=224331_s_at) | [MRPL36](http://www.ncbi.nlm.nih.gov/entrez/query.fcgi?cmd=search&db=gene&term=MRPL36) | | mitochondrial ribosomal protein L36 | 4e-07 | 2302.71 | 1467.88 | 1.57 |
| [205528_s_at](https://www.affymetrix.com/LinkServlet?probeset=205528_s_at) | [RUNX1T1](http://www.ncbi.nlm.nih.gov/entrez/query.fcgi?cmd=search&db=gene&term=RUNX1T1) | | runt-related transcription factor 1; translocated to, 1 (cyclin D-related) | 5e-07 | 84.98 | 228.34 | 0.37 |
| [202834_at](https://www.affymetrix.com/LinkServlet?probeset=202834_at) | [AGT](http://www.ncbi.nlm.nih.gov/entrez/query.fcgi?cmd=search&db=gene&term=AGT) | | angiotensinogen (serpin peptidase inhibitor, clade A, member 8) | 5e-07 | 990.72 | 3882.66 | 0.26 |
| [238022_at](https://www.affymetrix.com/LinkServlet?probeset=238022_at) | [CRNDE](http://www.ncbi.nlm.nih.gov/entrez/query.fcgi?cmd=search&db=gene&term=CRNDE) | | colorectal neoplasia differentially expressed (non-protein coding) | 7e-07 | 28.76 | 134.25 | 0.21 |
| [219197_s_at](https://www.affymetrix.com/LinkServlet?probeset=219197_s_at) | [SCUBE2](http://www.ncbi.nlm.nih.gov/entrez/query.fcgi?cmd=search&db=gene&term=SCUBE2) | | signal peptide, CUB domain, EGF-like 2 | 7e-07 | 43.94 | 180.8 | 0.24 |
| [229034_at](https://www.affymetrix.com/LinkServlet?probeset=229034_at) | [SOBP](http://www.ncbi.nlm.nih.gov/entrez/query.fcgi?cmd=search&db=gene&term=SOBP) | | sine oculis binding protein homolog (Drosophila) | 7e-07 | 135.35 | 295.79 | 0.46 |
| [220595_at](https://www.affymetrix.com/LinkServlet?probeset=220595_at) | [PDZRN4](http://www.ncbi.nlm.nih.gov/entrez/query.fcgi?cmd=search&db=gene&term=PDZRN4) | | PDZ domain containing ring finger 4 | 8e-07 | 20.3 | 115.23 | 0.18 |
| [220117_at](https://www.affymetrix.com/LinkServlet?probeset=220117_at) | [ZNF385D](http://www.ncbi.nlm.nih.gov/entrez/query.fcgi?cmd=search&db=gene&term=ZNF385D) | | zinc finger protein 385D | 8e-07 | 42.93 | 116.16 | 0.37 |
| [213478_at](https://www.affymetrix.com/LinkServlet?probeset=213478_at) | [RP1-21O18.1](http://www.ncbi.nlm.nih.gov/entrez/query.fcgi?cmd=search&db=gene&term=RP1-21O18.1) | | kazrin | 8e-07 | 225.68 | 482.21 | 0.47 |
| [226487_at](https://www.affymetrix.com/LinkServlet?probeset=226487_at) | [C12orf34](http://www.ncbi.nlm.nih.gov/entrez/query.fcgi?cmd=search&db=gene&term=C12orf34) | | chromosome 12 open reading frame 34 | 8e-07 | 58.88 | 141 | 0.42 |
| [227481_at](https://www.affymetrix.com/LinkServlet?probeset=227481_at) | [CNKSR3](http://www.ncbi.nlm.nih.gov/entrez/query.fcgi?cmd=search&db=gene&term=CNKSR3) | | CNKSR family member 3 | 9e-07 | 207.88 | 394.33 | 0.53 |
| [207443_at](https://www.affymetrix.com/LinkServlet?probeset=207443_at) | [NR2E1](http://www.ncbi.nlm.nih.gov/entrez/query.fcgi?cmd=search&db=gene&term=NR2E1) | | nuclear receptor subfamily 2, group E, member 1 | 9e-07 | 29.44 | 5.02 | 5.87 |
| [214841_at](https://www.affymetrix.com/LinkServlet?probeset=214841_at) | [CNIH3](http://www.ncbi.nlm.nih.gov/entrez/query.fcgi?cmd=search&db=gene&term=CNIH3) | | cornichon homolog 3 (Drosophila) | 9e-07 | 296.65 | 85.46 | 3.47 |
| [230802_at](https://www.affymetrix.com/LinkServlet?probeset=230802_at) | [ARHGAP24](http://www.ncbi.nlm.nih.gov/entrez/query.fcgi?cmd=search&db=gene&term=ARHGAP24) | | Rho GTPase activating protein 24 | 9e-07 | 36.2 | 13.79 | 2.63 |
| [231223_at](https://www.affymetrix.com/LinkServlet?probeset=231223_at) | [CSMD1](http://www.ncbi.nlm.nih.gov/entrez/query.fcgi?cmd=search&db=gene&term=CSMD1) | | CUB and Sushi multiple domains 1 | 9e-07 | 113.94 | 409.94 | 0.28 |
| [234996_at](https://www.affymetrix.com/LinkServlet?probeset=234996_at) | [CALCRL](http://www.ncbi.nlm.nih.gov/entrez/query.fcgi?cmd=search&db=gene&term=CALCRL) | | calcitonin receptor-like | 1e-06 | 90.37 | 281.9 | 0.32 |
| [214761_at](https://www.affymetrix.com/LinkServlet?probeset=214761_at) | [ZNF423](http://www.ncbi.nlm.nih.gov/entrez/query.fcgi?cmd=search&db=gene&term=ZNF423) | | zinc finger protein 423 | 1e-06 | 427.84 | 1140.35 | 0.38 |
| [229638_at](https://www.affymetrix.com/LinkServlet?probeset=229638_at) | [IRX3](http://www.ncbi.nlm.nih.gov/entrez/query.fcgi?cmd=search&db=gene&term=IRX3) | | iroquois homeobox 3 | 1e-06 | 29.77 | 145.48 | 0.2 |
| [222895_s_at](https://www.affymetrix.com/LinkServlet?probeset=222895_s_at) | [BCL11B](http://www.ncbi.nlm.nih.gov/entrez/query.fcgi?cmd=search&db=gene&term=BCL11B) | | B-cell CLL/lymphoma 11B (zinc finger protein) | 1.2e-06 | 11.45 | 40.03 | 0.29 |
| [219528_s_at](https://www.affymetrix.com/LinkServlet?probeset=219528_s_at) | [BCL11B](http://www.ncbi.nlm.nih.gov/entrez/query.fcgi?cmd=search&db=gene&term=BCL11B) | | B-cell CLL/lymphoma 11B (zinc finger protein) | 1.3e-06 | 13.19 | 36.89 | 0.36 |
| [225706_at](https://www.affymetrix.com/LinkServlet?probeset=225706_at) | [GLCCI1](http://www.ncbi.nlm.nih.gov/entrez/query.fcgi?cmd=search&db=gene&term=GLCCI1) | | glucocorticoid induced transcript 1 | 1.4e-06 | 312.39 | 638.43 | 0.49 |
| [208221_s_at](https://www.affymetrix.com/LinkServlet?probeset=208221_s_at) | [SLIT1](http://www.ncbi.nlm.nih.gov/entrez/query.fcgi?cmd=search&db=gene&term=SLIT1) | | slit homolog 1 (Drosophila) | 1.8e-06 | 12.71 | 6 | 2.12 |
| [238021_s_at](https://www.affymetrix.com/LinkServlet?probeset=238021_s_at) | [CRNDE](http://www.ncbi.nlm.nih.gov/entrez/query.fcgi?cmd=search&db=gene&term=CRNDE) | | colorectal neoplasia differentially expressed (non-protein coding) | 1.8e-06 | 154.56 | 1216.52 | 0.13 |
| [208017_s_at](https://www.affymetrix.com/LinkServlet?probeset=208017_s_at) | [MCF2](http://www.ncbi.nlm.nih.gov/entrez/query.fcgi?cmd=search&db=gene&term=MCF2) | | MCF.2 cell line derived transforming sequence | 2e-06 | 28.6 | 147.94 | 0.19 |
| [222484_s_at](https://www.affymetrix.com/LinkServlet?probeset=222484_s_at) | [CXCL14](http://www.ncbi.nlm.nih.gov/entrez/query.fcgi?cmd=search&db=gene&term=CXCL14) | | chemokine (C-X-C motif) ligand 14 | 2e-06 | 583.97 | 45.69 | 12.78 |
| [228307_at](https://www.affymetrix.com/LinkServlet?probeset=228307_at) | [EMILIN3](http://www.ncbi.nlm.nih.gov/entrez/query.fcgi?cmd=search&db=gene&term=EMILIN3) | | elastin microfibril interfacer 3 | 2.2e-06 | 41.71 | 168.05 | 0.25 |
| [213752_at](https://www.affymetrix.com/LinkServlet?probeset=213752_at) | [RP1-21O18.1](http://www.ncbi.nlm.nih.gov/entrez/query.fcgi?cmd=search&db=gene&term=RP1-21O18.1) | | kazrin | 2.3e-06 | 90.9 | 209.82 | 0.43 |
| [239782_at](https://www.affymetrix.com/LinkServlet?probeset=239782_at) | [RBP1](http://www.ncbi.nlm.nih.gov/entrez/query.fcgi?cmd=search&db=gene&term=RBP1) | | retinol binding protein 1, cellular | 2.3e-06 | 9.28 | 16.36 | 0.57 |
| [240067_at](https://www.affymetrix.com/LinkServlet?probeset=240067_at) | [NA](http://www.ncbi.nlm.nih.gov/entrez/query.fcgi?cmd=search&db=gene&term=NA) | | NA | 2.7e-06 | 26.47 | 146.23 | 0.18 |
| [235236_at](https://www.affymetrix.com/LinkServlet?probeset=235236_at) | [LOC100131897](http://www.ncbi.nlm.nih.gov/entrez/query.fcgi?cmd=search&db=gene&term=LOC100131897) | | Uncharacterized protein LOC100131897 | 2.8e-06 | 26.05 | 158.79 | 0.16 |
| [229159_at](https://www.affymetrix.com/LinkServlet?probeset=229159_at) | [THSD7A](http://www.ncbi.nlm.nih.gov/entrez/query.fcgi?cmd=search&db=gene&term=THSD7A) | | thrombospondin, type I, domain containing 7A | 3e-06 | 19.65 | 71.42 | 0.28 |
| [243879_at](https://www.affymetrix.com/LinkServlet?probeset=243879_at) | [NA](http://www.ncbi.nlm.nih.gov/entrez/query.fcgi?cmd=search&db=gene&term=NA) | | NA | 3e-06 | 65.13 | 225.57 | 0.29 |
| [243139_at](https://www.affymetrix.com/LinkServlet?probeset=243139_at) | [NA](http://www.ncbi.nlm.nih.gov/entrez/query.fcgi?cmd=search&db=gene&term=NA) | | NA | 3e-06 | 11.33 | 40.36 | 0.28 |
| [210347_s_at](https://www.affymetrix.com/LinkServlet?probeset=210347_s_at) | [BCL11A](http://www.ncbi.nlm.nih.gov/entrez/query.fcgi?cmd=search&db=gene&term=BCL11A) | | B-cell CLL/lymphoma 11A (zinc finger protein) | 3.2e-06 | 25.6 | 6.5 | 3.94 |
| [238846_at](https://www.affymetrix.com/LinkServlet?probeset=238846_at) | [TNFRSF11A](http://www.ncbi.nlm.nih.gov/entrez/query.fcgi?cmd=search&db=gene&term=TNFRSF11A) | | tumor necrosis factor receptor superfamily, member 11a, NFKB activator | 3.2e-06 | 84.06 | 32.03 | 2.62 |
| [244694_at](https://www.affymetrix.com/LinkServlet?probeset=244694_at) | [IGLON5](http://www.ncbi.nlm.nih.gov/entrez/query.fcgi?cmd=search&db=gene&term=IGLON5) | | IgLON family member 5 | 3.9e-06 | 9.17 | 34.76 | 0.26 |
| [1563561_at](https://www.affymetrix.com/LinkServlet?probeset=1563561_at) | [NA](http://www.ncbi.nlm.nih.gov/entrez/query.fcgi?cmd=search&db=gene&term=NA) | | NA | 4e-06 | 5.35 | 14.47 | 0.37 |
| [219537_x_at](https://www.affymetrix.com/LinkServlet?probeset=219537_x_at) | [DLL3](http://www.ncbi.nlm.nih.gov/entrez/query.fcgi?cmd=search&db=gene&term=DLL3) | | delta-like 3 (Drosophila) | 4.1e-06 | 19.88 | 77.64 | 0.26 |
| [207250_at](https://www.affymetrix.com/LinkServlet?probeset=207250_at) | [SIX6](http://www.ncbi.nlm.nih.gov/entrez/query.fcgi?cmd=search&db=gene&term=SIX6) | | SIX homeobox 6 | 4.4e-06 | 50.64 | 4.75 | 10.66 |
| [228728_at](https://www.affymetrix.com/LinkServlet?probeset=228728_at) | [C7orf58](http://www.ncbi.nlm.nih.gov/entrez/query.fcgi?cmd=search&db=gene&term=C7orf58) | | chromosome 7 open reading frame 58 | 4.5e-06 | 440.89 | 178.44 | 2.47 |
| [229714_at](https://www.affymetrix.com/LinkServlet?probeset=229714_at) | [HS6ST3](http://www.ncbi.nlm.nih.gov/entrez/query.fcgi?cmd=search&db=gene&term=HS6ST3) | | heparan sulfate 6-O-sulfotransferase 3 | 5e-06 | 20.08 | 75.06 | 0.27 |
| [213285_at](https://www.affymetrix.com/LinkServlet?probeset=213285_at) | [TMEM30B](http://www.ncbi.nlm.nih.gov/entrez/query.fcgi?cmd=search&db=gene&term=TMEM30B) | | transmembrane protein 30B | 5.3e-06 | 14.75 | 5.08 | 2.91 |
| [232275_s_at](https://www.affymetrix.com/LinkServlet?probeset=232275_s_at) | [HS6ST3](http://www.ncbi.nlm.nih.gov/entrez/query.fcgi?cmd=search&db=gene&term=HS6ST3) | | heparan sulfate 6-O-sulfotransferase 3 | 5.8e-06 | 9.76 | 39.85 | 0.24 |
| [224970_at](https://www.affymetrix.com/LinkServlet?probeset=224970_at) | [NFIA](http://www.ncbi.nlm.nih.gov/entrez/query.fcgi?cmd=search&db=gene&term=NFIA) | | nuclear factor I/A | 5.9e-06 | 1438.1 | 2560.05 | 0.56 |
| [1555867_at](https://www.affymetrix.com/LinkServlet?probeset=1555867_at) | [GNG4](http://www.ncbi.nlm.nih.gov/entrez/query.fcgi?cmd=search&db=gene&term=GNG4) | | guanine nucleotide binding protein (G protein), gamma 4 | 6.2e-06 | 56.06 | 250.53 | 0.22 |
| [235591_at](https://www.affymetrix.com/LinkServlet?probeset=235591_at) | [SSTR1](http://www.ncbi.nlm.nih.gov/entrez/query.fcgi?cmd=search&db=gene&term=SSTR1) | | somatostatin receptor 1 | 6.4e-06 | 31.36 | 203.32 | 0.15 |
| [218002_s_at](https://www.affymetrix.com/LinkServlet?probeset=218002_s_at) | [CXCL14](http://www.ncbi.nlm.nih.gov/entrez/query.fcgi?cmd=search&db=gene&term=CXCL14) | | chemokine (C-X-C motif) ligand 14 | 6.5e-06 | 674.45 | 53.24 | 12.67 |
| [1557745_at](https://www.affymetrix.com/LinkServlet?probeset=1557745_at) | [NA](http://www.ncbi.nlm.nih.gov/entrez/query.fcgi?cmd=search&db=gene&term=NA) | | NA | 6.5e-06 | 18.99 | 117.81 | 0.16 |
| [201571_s_at](https://www.affymetrix.com/LinkServlet?probeset=201571_s_at) | [DCTD](http://www.ncbi.nlm.nih.gov/entrez/query.fcgi?cmd=search&db=gene&term=DCTD) | | dCMP deaminase | 6.7e-06 | 79.33 | 36.51 | 2.17 |
| [218796_at](https://www.affymetrix.com/LinkServlet?probeset=218796_at) | [FERMT1](http://www.ncbi.nlm.nih.gov/entrez/query.fcgi?cmd=search&db=gene&term=FERMT1) | | fermitin family homolog 1 (Drosophila) | 6.7e-06 | 49.2 | 241.78 | 0.2 |
| [205529_s_at](https://www.affymetrix.com/LinkServlet?probeset=205529_s_at) | [RUNX1T1](http://www.ncbi.nlm.nih.gov/entrez/query.fcgi?cmd=search&db=gene&term=RUNX1T1) | | runt-related transcription factor 1; translocated to, 1 (cyclin D-related) | 7.1e-06 | 159.4 | 469.32 | 0.34 |
| [227282_at](https://www.affymetrix.com/LinkServlet?probeset=227282_at) | [PCDH19](http://www.ncbi.nlm.nih.gov/entrez/query.fcgi?cmd=search&db=gene&term=PCDH19) | | protocadherin 19 | 7.2e-06 | 136.62 | 487.79 | 0.28 |
| [207705_s_at](https://www.affymetrix.com/LinkServlet?probeset=207705_s_at) | [NINL](http://www.ncbi.nlm.nih.gov/entrez/query.fcgi?cmd=search&db=gene&term=NINL) | | ninein-like | 7.5e-06 | 99.2 | 167.77 | 0.59 |
| [1553972_a_at](https://www.affymetrix.com/LinkServlet?probeset=1553972_a_at) | [CBS](http://www.ncbi.nlm.nih.gov/entrez/query.fcgi?cmd=search&db=gene&term=CBS) | | cystathionine-beta-synthase | 7.5e-06 | 41.45 | 82.76 | 0.5 |
| [212208_at](https://www.affymetrix.com/LinkServlet?probeset=212208_at) | [MED13L](http://www.ncbi.nlm.nih.gov/entrez/query.fcgi?cmd=search&db=gene&term=MED13L) | | mediator complex subunit 13-like | 7.7e-06 | 120.82 | 200.65 | 0.6 |
| [57588_at](https://www.affymetrix.com/LinkServlet?probeset=57588_at) | [SLC24A3](http://www.ncbi.nlm.nih.gov/entrez/query.fcgi?cmd=search&db=gene&term=SLC24A3) | | solute carrier family 24 (sodium/potassium/calcium exchanger), member 3 | 8.5e-06 | 116.88 | 369.33 | 0.32 |
| [205240_at](https://www.affymetrix.com/LinkServlet?probeset=205240_at) | [GPSM2](http://www.ncbi.nlm.nih.gov/entrez/query.fcgi?cmd=search&db=gene&term=GPSM2) | | G-protein signaling modulator 2 (AGS3-like, C. elegans) | 8.7e-06 | 99.14 | 196.22 | 0.51 |
| [229160_at](https://www.affymetrix.com/LinkServlet?probeset=229160_at) | [MUM1L1](http://www.ncbi.nlm.nih.gov/entrez/query.fcgi?cmd=search&db=gene&term=MUM1L1) | | melanoma associated antigen (mutated) 1-like 1 | 8.8e-06 | 13.27 | 43.05 | 0.31 |
| [213601_at](https://www.affymetrix.com/LinkServlet?probeset=213601_at) | [SLIT1](http://www.ncbi.nlm.nih.gov/entrez/query.fcgi?cmd=search&db=gene&term=SLIT1) | | slit homolog 1 (Drosophila) | 9e-06 | 477.83 | 105.38 | 4.53 |
| [216086_at](https://www.affymetrix.com/LinkServlet?probeset=216086_at) | [SV2C](http://www.ncbi.nlm.nih.gov/entrez/query.fcgi?cmd=search&db=gene&term=SV2C) | | synaptic vesicle glycoprotein 2C | 9.5e-06 | 11.46 | 48.72 | 0.24 |
| [212239_at](https://www.affymetrix.com/LinkServlet?probeset=212239_at) | [PIK3R1](http://www.ncbi.nlm.nih.gov/entrez/query.fcgi?cmd=search&db=gene&term=PIK3R1) | | phosphoinositide-3-kinase, regulatory subunit 1 (alpha) | 9.6e-06 | 2754.8 | 4694.65 | 0.59 |
| [244764_at](https://www.affymetrix.com/LinkServlet?probeset=244764_at) | [NA](http://www.ncbi.nlm.nih.gov/entrez/query.fcgi?cmd=search&db=gene&term=NA) | | NA | 9.6e-06 | 149.98 | 77.52 | 1.93 |
| [226806_s_at](https://www.affymetrix.com/LinkServlet?probeset=226806_s_at) | [NFIA](http://www.ncbi.nlm.nih.gov/entrez/query.fcgi?cmd=search&db=gene&term=NFIA) | | nuclear factor I/A | 9.8e-06 | 2554.53 | 4254.38 | 0.6 |
| [209035_at](https://www.affymetrix.com/LinkServlet?probeset=209035_at) | [MDK](http://www.ncbi.nlm.nih.gov/entrez/query.fcgi?cmd=search&db=gene&term=MDK) | | midkine (neurite growth-promoting factor 2) | 1.01e-05 | 125.63 | 46.6 | 2.7 |
| [219501_at](https://www.affymetrix.com/LinkServlet?probeset=219501_at) | [ENOX1](http://www.ncbi.nlm.nih.gov/entrez/query.fcgi?cmd=search&db=gene&term=ENOX1) | | ecto-NOX disulfide-thiol exchanger 1 | 1.02e-05 | 52.53 | 122.13 | 0.43 |
| [60474_at](https://www.affymetrix.com/LinkServlet?probeset=60474_at) | [FERMT1](http://www.ncbi.nlm.nih.gov/entrez/query.fcgi?cmd=search&db=gene&term=FERMT1) | | fermitin family homolog 1 (Drosophila) | 1.03e-05 | 59.85 | 281.77 | 0.21 |
| [207231_at](https://www.affymetrix.com/LinkServlet?probeset=207231_at) | [DZIP3](http://www.ncbi.nlm.nih.gov/entrez/query.fcgi?cmd=search&db=gene&term=DZIP3) | | DAZ interacting protein 3, zinc finger | 1.07e-05 | 97.19 | 158.2 | 0.61 |
| [209815_at](https://www.affymetrix.com/LinkServlet?probeset=209815_at) | [PTCH1](http://www.ncbi.nlm.nih.gov/entrez/query.fcgi?cmd=search&db=gene&term=PTCH1) | | patched homolog 1 (Drosophila) | 1.07e-05 | 527.69 | 1153.95 | 0.46 |
| [242319_at](https://www.affymetrix.com/LinkServlet?probeset=242319_at) | [DGKG](http://www.ncbi.nlm.nih.gov/entrez/query.fcgi?cmd=search&db=gene&term=DGKG) | | diacylglycerol kinase, gamma 90kDa | 1.11e-05 | 7.01 | 24.9 | 0.28 |
| [201572_x_at](https://www.affymetrix.com/LinkServlet?probeset=201572_x_at) | [DCTD](http://www.ncbi.nlm.nih.gov/entrez/query.fcgi?cmd=search&db=gene&term=DCTD) | | dCMP deaminase | 1.15e-05 | 309.48 | 199.42 | 1.55 |
| [240312_at](https://www.affymetrix.com/LinkServlet?probeset=240312_at) | [LOC389895](http://www.ncbi.nlm.nih.gov/entrez/query.fcgi?cmd=search&db=gene&term=LOC389895) | | hypothetical LOC389895 | 1.16e-05 | 9.93 | 28.42 | 0.35 |
| [229163_at](https://www.affymetrix.com/LinkServlet?probeset=229163_at) | [CAMK2N1](http://www.ncbi.nlm.nih.gov/entrez/query.fcgi?cmd=search&db=gene&term=CAMK2N1) | | calcium/calmodulin-dependent protein kinase II inhibitor 1 | 1.2e-05 | 182.9 | 337.45 | 0.54 |
| [225928_at](https://www.affymetrix.com/LinkServlet?probeset=225928_at) | [NA](http://www.ncbi.nlm.nih.gov/entrez/query.fcgi?cmd=search&db=gene&term=NA) | | NA | 1.25e-05 | 62.45 | 39.73 | 1.57 |
| [214460_at](https://www.affymetrix.com/LinkServlet?probeset=214460_at) | [LSAMP](http://www.ncbi.nlm.nih.gov/entrez/query.fcgi?cmd=search&db=gene&term=LSAMP) | | limbic system-associated membrane protein | 1.28e-05 | 217.62 | 431.13 | 0.5 |
| [204570_at](https://www.affymetrix.com/LinkServlet?probeset=204570_at) | [COX7A1](http://www.ncbi.nlm.nih.gov/entrez/query.fcgi?cmd=search&db=gene&term=COX7A1) | | cytochrome c oxidase subunit VIIa polypeptide 1 (muscle) | 1.36e-05 | 524.29 | 134.65 | 3.89 |
| [212240_s_at](https://www.affymetrix.com/LinkServlet?probeset=212240_s_at) | [PIK3R1](http://www.ncbi.nlm.nih.gov/entrez/query.fcgi?cmd=search&db=gene&term=PIK3R1) | | phosphoinositide-3-kinase, regulatory subunit 1 (alpha) | 1.38e-05 | 626.41 | 1245.74 | 0.5 |
| [204082_at](https://www.affymetrix.com/LinkServlet?probeset=204082_at) | [PBX3](http://www.ncbi.nlm.nih.gov/entrez/query.fcgi?cmd=search&db=gene&term=PBX3) | | pre-B-cell leukemia homeobox 3 | 1.39e-05 | 317.27 | 559.81 | 0.57 |
| [235567_at](https://www.affymetrix.com/LinkServlet?probeset=235567_at) | [RORA](http://www.ncbi.nlm.nih.gov/entrez/query.fcgi?cmd=search&db=gene&term=RORA) | | RAR-related orphan receptor A | 1.44e-05 | 34.23 | 117.05 | 0.29 |
| [222787_s_at](https://www.affymetrix.com/LinkServlet?probeset=222787_s_at) | [TMEM106B](http://www.ncbi.nlm.nih.gov/entrez/query.fcgi?cmd=search&db=gene&term=TMEM106B) | | transmembrane protein 106B | 1.45e-05 | 749.37 | 1282.15 | 0.58 |
| [228425_at](https://www.affymetrix.com/LinkServlet?probeset=228425_at) | [LOC654433](http://www.ncbi.nlm.nih.gov/entrez/query.fcgi?cmd=search&db=gene&term=LOC654433) | | hypothetical LOC654433 | 1.46e-05 | 16.91 | 5.04 | 3.36 |
| [229740_at](https://www.affymetrix.com/LinkServlet?probeset=229740_at) | [LOC643008](http://www.ncbi.nlm.nih.gov/entrez/query.fcgi?cmd=search&db=gene&term=LOC643008) | | hypothetical protein LOC643008 | 1.49e-05 | 86.12 | 26.72 | 3.22 |
| [230418_s_at](https://www.affymetrix.com/LinkServlet?probeset=230418_s_at) | [GALNTL1](http://www.ncbi.nlm.nih.gov/entrez/query.fcgi?cmd=search&db=gene&term=GALNTL1) | | UDP-N-acetyl-alpha-D-galactosamine | 1.57e-05 | 117.19 | 383.07 | 0.31 |
| [204214_s_at](https://www.affymetrix.com/LinkServlet?probeset=204214_s_at) | [RAB32](http://www.ncbi.nlm.nih.gov/entrez/query.fcgi?cmd=search&db=gene&term=RAB32) | | RAB32, member RAS oncogene family | 1.57e-05 | 198.89 | 81.2 | 2.45 |
| [210137_s_at](https://www.affymetrix.com/LinkServlet?probeset=210137_s_at) | [DCTD](http://www.ncbi.nlm.nih.gov/entrez/query.fcgi?cmd=search&db=gene&term=DCTD) | | dCMP deaminase | 1.57e-05 | 245.76 | 154.64 | 1.59 |
| [220010_at](https://www.affymetrix.com/LinkServlet?probeset=220010_at) | [KCNE1L](http://www.ncbi.nlm.nih.gov/entrez/query.fcgi?cmd=search&db=gene&term=KCNE1L) | | KCNE1-like | 1.62e-05 | 16.69 | 64.57 | 0.26 |
| [221044_s_at](https://www.affymetrix.com/LinkServlet?probeset=221044_s_at) | [NA](http://www.ncbi.nlm.nih.gov/entrez/query.fcgi?cmd=search&db=gene&term=NA) | | NA | 1.63e-05 | 89.2 | 40.8 | 2.19 |
| [1561985_at](https://www.affymetrix.com/LinkServlet?probeset=1561985_at) | [C14orf39](http://www.ncbi.nlm.nih.gov/entrez/query.fcgi?cmd=search&db=gene&term=C14orf39) | | chromosome 14 open reading frame 39 | 1.63e-05 | 27.92 | 4.76 | 5.87 |
| [205932_s_at](https://www.affymetrix.com/LinkServlet?probeset=205932_s_at) | [MSX1](http://www.ncbi.nlm.nih.gov/entrez/query.fcgi?cmd=search&db=gene&term=MSX1) | | msh homeobox 1 | 1.63e-05 | 177.8 | 60.55 | 2.94 |
| [218870_at](https://www.affymetrix.com/LinkServlet?probeset=218870_at) | [ARHGAP15](http://www.ncbi.nlm.nih.gov/entrez/query.fcgi?cmd=search&db=gene&term=ARHGAP15) | | Rho GTPase activating protein 15 | 1.67e-05 | 191.59 | 88.64 | 2.16 |
| [235024_at](https://www.affymetrix.com/LinkServlet?probeset=235024_at) | [PHF17](http://www.ncbi.nlm.nih.gov/entrez/query.fcgi?cmd=search&db=gene&term=PHF17) | | PHD finger protein 17 | 1.69e-05 | 27.89 | 49.34 | 0.57 |
| [205330_at](https://www.affymetrix.com/LinkServlet?probeset=205330_at) | [MN1](http://www.ncbi.nlm.nih.gov/entrez/query.fcgi?cmd=search&db=gene&term=MN1) | | meningioma (disrupted in balanced translocation) 1 | 1.76e-05 | 120.15 | 405.26 | 0.3 |
| [214633_at](https://www.affymetrix.com/LinkServlet?probeset=214633_at) | [SOX3](http://www.ncbi.nlm.nih.gov/entrez/query.fcgi?cmd=search&db=gene&term=SOX3) | | SRY (sex determining region Y)-box 3 | 1.93e-05 | 14.49 | 36.25 | 0.4 |
| [1552430_at](https://www.affymetrix.com/LinkServlet?probeset=1552430_at) | [WDR17](http://www.ncbi.nlm.nih.gov/entrez/query.fcgi?cmd=search&db=gene&term=WDR17) | | WD repeat domain 17 | 1.96e-05 | 17.8 | 40.06 | 0.44 |
| [219331_s_at](https://www.affymetrix.com/LinkServlet?probeset=219331_s_at) | [KLHDC8A](http://www.ncbi.nlm.nih.gov/entrez/query.fcgi?cmd=search&db=gene&term=KLHDC8A) | | kelch domain containing 8A | 1.96e-05 | 63.94 | 23.85 | 2.68 |
| [227474_at](https://www.affymetrix.com/LinkServlet?probeset=227474_at) | [LOC654433](http://www.ncbi.nlm.nih.gov/entrez/query.fcgi?cmd=search&db=gene&term=LOC654433) | | hypothetical LOC654433 | 1.97e-05 | 29.26 | 10.52 | 2.78 |
| [232874_at](https://www.affymetrix.com/LinkServlet?probeset=232874_at) | [DOCK9](http://www.ncbi.nlm.nih.gov/entrez/query.fcgi?cmd=search&db=gene&term=DOCK9) | | dedicator of cytokinesis 9 | 1.98e-05 | 28.82 | 122.92 | 0.23 |
| [204304_s_at](https://www.affymetrix.com/LinkServlet?probeset=204304_s_at) | [PROM1](http://www.ncbi.nlm.nih.gov/entrez/query.fcgi?cmd=search&db=gene&term=PROM1) | | prominin 1 | 2e-05 | 68.07 | 350.45 | 0.19 |
| [219557_s_at](https://www.affymetrix.com/LinkServlet?probeset=219557_s_at) | [NRIP3](http://www.ncbi.nlm.nih.gov/entrez/query.fcgi?cmd=search&db=gene&term=NRIP3) | | nuclear receptor interacting protein 3 | 2.06e-05 | 60.07 | 208.18 | 0.29 |
| [224975_at](https://www.affymetrix.com/LinkServlet?probeset=224975_at) | [NFIA](http://www.ncbi.nlm.nih.gov/entrez/query.fcgi?cmd=search&db=gene&term=NFIA) | | nuclear factor I/A | 2.14e-05 | 1503.18 | 2456.56 | 0.61 |
| [230068_s_at](https://www.affymetrix.com/LinkServlet?probeset=230068_s_at) | [PEG3](http://www.ncbi.nlm.nih.gov/entrez/query.fcgi?cmd=search&db=gene&term=PEG3) | | paternally expressed 3 | 2.15e-05 | 55.97 | 118.56 | 0.47 |
| [219090_at](https://www.affymetrix.com/LinkServlet?probeset=219090_at) | [SLC24A3](http://www.ncbi.nlm.nih.gov/entrez/query.fcgi?cmd=search&db=gene&term=SLC24A3) | | solute carrier family 24 (sodium/potassium/calcium exchanger), member 3 | 2.18e-05 | 135.71 | 385.73 | 0.35 |
| [230496_at](https://www.affymetrix.com/LinkServlet?probeset=230496_at) | [FAM123A](http://www.ncbi.nlm.nih.gov/entrez/query.fcgi?cmd=search&db=gene&term=FAM123A) | | family with sequence similarity 123A | 2.21e-05 | 472.44 | 1820.6 | 0.26 |
| [242628_at](https://www.affymetrix.com/LinkServlet?probeset=242628_at) | [KLRB1](http://www.ncbi.nlm.nih.gov/entrez/query.fcgi?cmd=search&db=gene&term=KLRB1) | | killer cell lectin-like receptor subfamily B, member 1 | 2.37e-05 | 10.27 | 33.27 | 0.31 |
| [219355_at](https://www.affymetrix.com/LinkServlet?probeset=219355_at) | [CXorf57](http://www.ncbi.nlm.nih.gov/entrez/query.fcgi?cmd=search&db=gene&term=CXorf57) | | chromosome X open reading frame 57 | 2.42e-05 | 63.29 | 209.52 | 0.3 |
| [206984_s_at](https://www.affymetrix.com/LinkServlet?probeset=206984_s_at) | [RIT2](http://www.ncbi.nlm.nih.gov/entrez/query.fcgi?cmd=search&db=gene&term=RIT2) | | Ras-like without CAAX 2 | 2.46e-05 | 162.67 | 831.82 | 0.2 |
| [239907_at](https://www.affymetrix.com/LinkServlet?probeset=239907_at) | [NA](http://www.ncbi.nlm.nih.gov/entrez/query.fcgi?cmd=search&db=gene&term=NA) | | NA | 2.46e-05 | 49.62 | 204.24 | 0.24 |
| [228218_at](https://www.affymetrix.com/LinkServlet?probeset=228218_at) | [NA](http://www.ncbi.nlm.nih.gov/entrez/query.fcgi?cmd=search&db=gene&term=NA) | | NA | 2.46e-05 | 790.11 | 1334.04 | 0.59 |
| [244461_at](https://www.affymetrix.com/LinkServlet?probeset=244461_at) | [CYTSB](http://www.ncbi.nlm.nih.gov/entrez/query.fcgi?cmd=search&db=gene&term=CYTSB) | | cytospin B | 2.48e-05 | 46.1 | 24.29 | 1.9 |
| [205081_at](https://www.affymetrix.com/LinkServlet?probeset=205081_at) | [CRIP1](http://www.ncbi.nlm.nih.gov/entrez/query.fcgi?cmd=search&db=gene&term=CRIP1) | | cysteine-rich protein 1 (intestinal) | 2.52e-05 | 94.78 | 32.64 | 2.9 |
| [222942_s_at](https://www.affymetrix.com/LinkServlet?probeset=222942_s_at) | [TIAM2](http://www.ncbi.nlm.nih.gov/entrez/query.fcgi?cmd=search&db=gene&term=TIAM2) | | T-cell lymphoma invasion and metastasis 2 | 2.53e-05 | 61.58 | 204.18 | 0.3 |
| [222891_s_at](https://www.affymetrix.com/LinkServlet?probeset=222891_s_at) | [BCL11A](http://www.ncbi.nlm.nih.gov/entrez/query.fcgi?cmd=search&db=gene&term=BCL11A) | | B-cell CLL/lymphoma 11A (zinc finger protein) | 2.55e-05 | 30.71 | 8.97 | 3.43 |
| [1555462_at](https://www.affymetrix.com/LinkServlet?probeset=1555462_at) | [PPP1R1C](http://www.ncbi.nlm.nih.gov/entrez/query.fcgi?cmd=search&db=gene&term=PPP1R1C) | | protein phosphatase 1, regulatory (inhibitor) subunit 1C | 2.61e-05 | 16.22 | 5.07 | 3.2 |
| [208296_x_at](https://www.affymetrix.com/LinkServlet?probeset=208296_x_at) | [TNFAIP8](http://www.ncbi.nlm.nih.gov/entrez/query.fcgi?cmd=search&db=gene&term=TNFAIP8) | | tumor necrosis factor, alpha-induced protein 8 | 2.75e-05 | 123.28 | 54.65 | 2.26 |
| [239146_at](https://www.affymetrix.com/LinkServlet?probeset=239146_at) | [CLDND1](http://www.ncbi.nlm.nih.gov/entrez/query.fcgi?cmd=search&db=gene&term=CLDND1) | | claudin domain containing 1 | 2.9e-05 | 15.74 | 7.85 | 2 |
| [221922_at](https://www.affymetrix.com/LinkServlet?probeset=221922_at) | [GPSM2](http://www.ncbi.nlm.nih.gov/entrez/query.fcgi?cmd=search&db=gene&term=GPSM2) | | G-protein signaling modulator 2 (AGS3-like, C. elegans) | 2.93e-05 | 736 | 1371.89 | 0.54 |
| [227657_at](https://www.affymetrix.com/LinkServlet?probeset=227657_at) | [RNF150](http://www.ncbi.nlm.nih.gov/entrez/query.fcgi?cmd=search&db=gene&term=RNF150) | | ring finger protein 150 | 2.95e-05 | 146.89 | 307.61 | 0.48 |
| [228827_at](https://www.affymetrix.com/LinkServlet?probeset=228827_at) | [NA](http://www.ncbi.nlm.nih.gov/entrez/query.fcgi?cmd=search&db=gene&term=NA) | | NA | 3.03e-05 | 145.13 | 347.41 | 0.42 |
| [212207_at](https://www.affymetrix.com/LinkServlet?probeset=212207_at) | [MED13L](http://www.ncbi.nlm.nih.gov/entrez/query.fcgi?cmd=search&db=gene&term=MED13L) | | mediator complex subunit 13-like | 3.07e-05 | 179.67 | 285.48 | 0.63 |
| [206811_at](https://www.affymetrix.com/LinkServlet?probeset=206811_at) | [ADCY8](http://www.ncbi.nlm.nih.gov/entrez/query.fcgi?cmd=search&db=gene&term=ADCY8) | | adenylate cyclase 8 (brain) | 3.08e-05 | 12.22 | 49.58 | 0.25 |
| [235831_at](https://www.affymetrix.com/LinkServlet?probeset=235831_at) | [NA](http://www.ncbi.nlm.nih.gov/entrez/query.fcgi?cmd=search&db=gene&term=NA) | | NA | 3.11e-05 | 29.67 | 104.47 | 0.28 |
| [225102_at](https://www.affymetrix.com/LinkServlet?probeset=225102_at) | [MGLL](http://www.ncbi.nlm.nih.gov/entrez/query.fcgi?cmd=search&db=gene&term=MGLL) | | monoglyceride lipase | 3.12e-05 | 659.69 | 1701.41 | 0.39 |
| [230417_at](https://www.affymetrix.com/LinkServlet?probeset=230417_at) | [GALNTL1](http://www.ncbi.nlm.nih.gov/entrez/query.fcgi?cmd=search&db=gene&term=GALNTL1) | | UDP-N-acetyl-alpha-D-galactosamine | 3.13e-05 | 27.91 | 81.51 | 0.34 |
| [218403_at](https://www.affymetrix.com/LinkServlet?probeset=218403_at) | [TRIAP1](http://www.ncbi.nlm.nih.gov/entrez/query.fcgi?cmd=search&db=gene&term=TRIAP1) | | TP53 regulated inhibitor of apoptosis 1 | 3.26e-05 | 836.29 | 555.84 | 1.5 |
| [209047_at](https://www.affymetrix.com/LinkServlet?probeset=209047_at) | [AQP1](http://www.ncbi.nlm.nih.gov/entrez/query.fcgi?cmd=search&db=gene&term=AQP1) | | aquaporin 1 (Colton blood group) | 3.38e-05 | 132.13 | 1022.89 | 0.13 |
| [221587_s_at](https://www.affymetrix.com/LinkServlet?probeset=221587_s_at) | [C19orf24](http://www.ncbi.nlm.nih.gov/entrez/query.fcgi?cmd=search&db=gene&term=C19orf24) | | chromosome 19 open reading frame 24 | 3.42e-05 | 46.5 | 28.99 | 1.6 |
| [207857_at](https://www.affymetrix.com/LinkServlet?probeset=207857_at) | [LILRA2](http://www.ncbi.nlm.nih.gov/entrez/query.fcgi?cmd=search&db=gene&term=LILRA2) | | leukocyte immunoglobulin-like receptor, subfamily A (with TM domain), member 2 | 3.43e-05 | 165.8 | 80.35 | 2.06 |
| [236902_at](https://www.affymetrix.com/LinkServlet?probeset=236902_at) | [FLJ43390](http://www.ncbi.nlm.nih.gov/entrez/query.fcgi?cmd=search&db=gene&term=FLJ43390) | | hypothetical LOC646113 | 3.73e-05 | 11.87 | 52.39 | 0.23 |
| [237268_at](https://www.affymetrix.com/LinkServlet?probeset=237268_at) | [DSCAM](http://www.ncbi.nlm.nih.gov/entrez/query.fcgi?cmd=search&db=gene&term=DSCAM) | | Down syndrome cell adhesion molecule | 3.77e-05 | 107.7 | 379.96 | 0.28 |
| [227632_at](https://www.affymetrix.com/LinkServlet?probeset=227632_at) | [TBC1D24](http://www.ncbi.nlm.nih.gov/entrez/query.fcgi?cmd=search&db=gene&term=TBC1D24) | | TBC1 domain family, member 24 | 3.91e-05 | 48.08 | 96.35 | 0.5 |
| [206869_at](https://www.affymetrix.com/LinkServlet?probeset=206869_at) | [CHAD](http://www.ncbi.nlm.nih.gov/entrez/query.fcgi?cmd=search&db=gene&term=CHAD) | | chondroadherin | 3.99e-05 | 26.5 | 125.92 | 0.21 |
| [209472_at](https://www.affymetrix.com/LinkServlet?probeset=209472_at) | [CCBL2](http://www.ncbi.nlm.nih.gov/entrez/query.fcgi?cmd=search&db=gene&term=CCBL2) | | cysteine conjugate-beta lyase 2 | 4.13e-05 | 492.74 | 328.03 | 1.5 |
| [242172_at](https://www.affymetrix.com/LinkServlet?probeset=242172_at) | [MEIS1](http://www.ncbi.nlm.nih.gov/entrez/query.fcgi?cmd=search&db=gene&term=MEIS1) | | Meis homeobox 1 | 4.2e-05 | 9.51 | 32.44 | 0.29 |
| [221704_s_at](https://www.affymetrix.com/LinkServlet?probeset=221704_s_at) | [VPS37B](http://www.ncbi.nlm.nih.gov/entrez/query.fcgi?cmd=search&db=gene&term=VPS37B) | | vacuolar protein sorting 37 homolog B (S. cerevisiae) | 4.22e-05 | 191.6 | 100.14 | 1.91 |
| [211026_s_at](https://www.affymetrix.com/LinkServlet?probeset=211026_s_at) | [MGLL](http://www.ncbi.nlm.nih.gov/entrez/query.fcgi?cmd=search&db=gene&term=MGLL) | | monoglyceride lipase | 4.29e-05 | 613.34 | 1689.96 | 0.36 |
| [240218_at](https://www.affymetrix.com/LinkServlet?probeset=240218_at) | [DSCAM](http://www.ncbi.nlm.nih.gov/entrez/query.fcgi?cmd=search&db=gene&term=DSCAM) | | Down syndrome cell adhesion molecule | 4.31e-05 | 93.89 | 353.24 | 0.27 |
| [211484_s_at](https://www.affymetrix.com/LinkServlet?probeset=211484_s_at) | [DSCAM](http://www.ncbi.nlm.nih.gov/entrez/query.fcgi?cmd=search&db=gene&term=DSCAM) | | Down syndrome cell adhesion molecule | 4.33e-05 | 68.62 | 246.92 | 0.28 |
| [215311_at](https://www.affymetrix.com/LinkServlet?probeset=215311_at) | [NTRK3](http://www.ncbi.nlm.nih.gov/entrez/query.fcgi?cmd=search&db=gene&term=NTRK3) | | neurotrophic tyrosine kinase, receptor, type 3 | 4.52e-05 | 417.21 | 1333.53 | 0.31 |
| [225504_at](https://www.affymetrix.com/LinkServlet?probeset=225504_at) | [NA](http://www.ncbi.nlm.nih.gov/entrez/query.fcgi?cmd=search&db=gene&term=NA) | | NA | 4.53e-05 | 221.41 | 402.17 | 0.55 |
| [206622_at](https://www.affymetrix.com/LinkServlet?probeset=206622_at) | [TRH](http://www.ncbi.nlm.nih.gov/entrez/query.fcgi?cmd=search&db=gene&term=TRH) | | thyrotropin-releasing hormone | 4.54e-05 | 10.04 | 56.65 | 0.18 |
| [220134_x_at](https://www.affymetrix.com/LinkServlet?probeset=220134_x_at) | [FAM176B](http://www.ncbi.nlm.nih.gov/entrez/query.fcgi?cmd=search&db=gene&term=FAM176B) | | family with sequence similarity 176, member B | 4.55e-05 | 86.71 | 52.52 | 1.65 |
| [232235_at](https://www.affymetrix.com/LinkServlet?probeset=232235_at) | [DSEL](http://www.ncbi.nlm.nih.gov/entrez/query.fcgi?cmd=search&db=gene&term=DSEL) | | dermatan sulfate epimerase-like | 4.65e-05 | 1158.72 | 1957.76 | 0.59 |
| [205173_x_at](https://www.affymetrix.com/LinkServlet?probeset=205173_x_at) | [CD58](http://www.ncbi.nlm.nih.gov/entrez/query.fcgi?cmd=search&db=gene&term=CD58) | | CD58 molecule | 4.66e-05 | 643.66 | 261.28 | 2.46 |
| [219497_s_at](https://www.affymetrix.com/LinkServlet?probeset=219497_s_at) | [BCL11A](http://www.ncbi.nlm.nih.gov/entrez/query.fcgi?cmd=search&db=gene&term=BCL11A) | | B-cell CLL/lymphoma 11A (zinc finger protein) | 4.7e-05 | 20.82 | 6.64 | 3.13 |
| [223842_s_at](https://www.affymetrix.com/LinkServlet?probeset=223842_s_at) | [SCARA3](http://www.ncbi.nlm.nih.gov/entrez/query.fcgi?cmd=search&db=gene&term=SCARA3) | | scavenger receptor class A, member 3 | 4.95e-05 | 30.04 | 79.97 | 0.38 |
| [221710_x_at](https://www.affymetrix.com/LinkServlet?probeset=221710_x_at) | [FAM176B](http://www.ncbi.nlm.nih.gov/entrez/query.fcgi?cmd=search&db=gene&term=FAM176B) | | family with sequence similarity 176, member B | 4.95e-05 | 91.65 | 57.22 | 1.6 |
| [215014_at](https://www.affymetrix.com/LinkServlet?probeset=215014_at) | [KCND3](http://www.ncbi.nlm.nih.gov/entrez/query.fcgi?cmd=search&db=gene&term=KCND3) | | potassium voltage-gated channel, Shal-related subfamily, member 3 | 4.97e-05 | 163.57 | 367.16 | 0.45 |
| [230463_at](https://www.affymetrix.com/LinkServlet?probeset=230463_at) | [NA](http://www.ncbi.nlm.nih.gov/entrez/query.fcgi?cmd=search&db=gene&term=NA) | | NA | 5.02e-05 | 202.89 | 489.19 | 0.41 |
| [235465_at](https://www.affymetrix.com/LinkServlet?probeset=235465_at) | [FAM123A](http://www.ncbi.nlm.nih.gov/entrez/query.fcgi?cmd=search&db=gene&term=FAM123A) | | family with sequence similarity 123A | 5.03e-05 | 263.08 | 947.1 | 0.28 |
| [240735_at](https://www.affymetrix.com/LinkServlet?probeset=240735_at) | [CDC42BPA](http://www.ncbi.nlm.nih.gov/entrez/query.fcgi?cmd=search&db=gene&term=CDC42BPA) | | CDC42 binding protein kinase alpha (DMPK-like) | 5.03e-05 | 7.78 | 18.48 | 0.42 |
| [234314_at](https://www.affymetrix.com/LinkServlet?probeset=234314_at) | [C20orf74](http://www.ncbi.nlm.nih.gov/entrez/query.fcgi?cmd=search&db=gene&term=C20orf74) | | chromosome 20 open reading frame 74 | 5.04e-05 | 5.78 | 63.96 | 0.09 |
| [226415_at](https://www.affymetrix.com/LinkServlet?probeset=226415_at) | [VAT1L](http://www.ncbi.nlm.nih.gov/entrez/query.fcgi?cmd=search&db=gene&term=VAT1L) | | vesicle amine transport protein 1 homolog (T. californica)-like | 5.07e-05 | 571.25 | 1444.34 | 0.4 |
| [232276_at](https://www.affymetrix.com/LinkServlet?probeset=232276_at) | [HS6ST3](http://www.ncbi.nlm.nih.gov/entrez/query.fcgi?cmd=search&db=gene&term=HS6ST3) | | heparan sulfate 6-O-sulfotransferase 3 | 5.08e-05 | 52.67 | 189.65 | 0.28 |
| [243756_at](https://www.affymetrix.com/LinkServlet?probeset=243756_at) | [NA](http://www.ncbi.nlm.nih.gov/entrez/query.fcgi?cmd=search&db=gene&term=NA) | | NA | 5.08e-05 | 16.61 | 70.87 | 0.23 |
| [215425_at](https://www.affymetrix.com/LinkServlet?probeset=215425_at) | [BTG3](http://www.ncbi.nlm.nih.gov/entrez/query.fcgi?cmd=search&db=gene&term=BTG3) | | BTG family, member 3 | 5.14e-05 | 38.91 | 20.55 | 1.89 |
| [238009_at](https://www.affymetrix.com/LinkServlet?probeset=238009_at) | [NA](http://www.ncbi.nlm.nih.gov/entrez/query.fcgi?cmd=search&db=gene&term=NA) | | NA | 5.15e-05 | 484.54 | 890.72 | 0.54 |
| [205110_s_at](https://www.affymetrix.com/LinkServlet?probeset=205110_s_at) | [FGF13](http://www.ncbi.nlm.nih.gov/entrez/query.fcgi?cmd=search&db=gene&term=FGF13) | | fibroblast growth factor 13 | 5.58e-05 | 42.29 | 206.23 | 0.21 |
| [223396_at](https://www.affymetrix.com/LinkServlet?probeset=223396_at) | [TMEM60](http://www.ncbi.nlm.nih.gov/entrez/query.fcgi?cmd=search&db=gene&term=TMEM60) | | transmembrane protein 60 | 5.62e-05 | 527.92 | 333.84 | 1.58 |
| [204072_s_at](https://www.affymetrix.com/LinkServlet?probeset=204072_s_at) | [FRY](http://www.ncbi.nlm.nih.gov/entrez/query.fcgi?cmd=search&db=gene&term=FRY) | | furry homolog (Drosophila) | 5.67e-05 | 597.97 | 1028.38 | 0.58 |
| [1557345_at](https://www.affymetrix.com/LinkServlet?probeset=1557345_at) | [LOC283516](http://www.ncbi.nlm.nih.gov/entrez/query.fcgi?cmd=search&db=gene&term=LOC283516) | | hypothetical protein LOC283516 | 5.7e-05 | 6.6 | 10.51 | 0.63 |
| [203911_at](https://www.affymetrix.com/LinkServlet?probeset=203911_at) | [RAP1GAP](http://www.ncbi.nlm.nih.gov/entrez/query.fcgi?cmd=search&db=gene&term=RAP1GAP) | | RAP1 GTPase activating protein | 5.73e-05 | 29.98 | 90.37 | 0.33 |
| [201944_at](https://www.affymetrix.com/LinkServlet?probeset=201944_at) | [HEXB](http://www.ncbi.nlm.nih.gov/entrez/query.fcgi?cmd=search&db=gene&term=HEXB) | | hexosaminidase B (beta polypeptide) | 5.83e-05 | 1733.54 | 1044.29 | 1.66 |
| [224325_at](https://www.affymetrix.com/LinkServlet?probeset=224325_at) | [FZD8](http://www.ncbi.nlm.nih.gov/entrez/query.fcgi?cmd=search&db=gene&term=FZD8) | | frizzled homolog 8 (Drosophila) | 5.88e-05 | 157.4 | 48.75 | 3.23 |
| [216942_s_at](https://www.affymetrix.com/LinkServlet?probeset=216942_s_at) | [CD58](http://www.ncbi.nlm.nih.gov/entrez/query.fcgi?cmd=search&db=gene&term=CD58) | | CD58 molecule | 6.14e-05 | 249.53 | 86.02 | 2.9 |
| [203732_at](https://www.affymetrix.com/LinkServlet?probeset=203732_at) | [TRIP4](http://www.ncbi.nlm.nih.gov/entrez/query.fcgi?cmd=search&db=gene&term=TRIP4) | | thyroid hormone receptor interactor 4 | 6.15e-05 | 222.17 | 148.84 | 1.49 |
| [204959_at](https://www.affymetrix.com/LinkServlet?probeset=204959_at) | [MNDA](http://www.ncbi.nlm.nih.gov/entrez/query.fcgi?cmd=search&db=gene&term=MNDA) | | myeloid cell nuclear differentiation antigen | 6.16e-05 | 532.69 | 215.78 | 2.47 |
| [212816_s_at](https://www.affymetrix.com/LinkServlet?probeset=212816_s_at) | [CBS](http://www.ncbi.nlm.nih.gov/entrez/query.fcgi?cmd=search&db=gene&term=CBS) | | cystathionine-beta-synthase | 6.31e-05 | 50.3 | 95.14 | 0.53 |
| [228602_at](https://www.affymetrix.com/LinkServlet?probeset=228602_at) | [SGCD](http://www.ncbi.nlm.nih.gov/entrez/query.fcgi?cmd=search&db=gene&term=SGCD) | | sarcoglycan, delta (35kDa dystrophin-associated glycoprotein) | 6.32e-05 | 106.66 | 241.44 | 0.44 |
| [213997_at](https://www.affymetrix.com/LinkServlet?probeset=213997_at) | [FAM189A1](http://www.ncbi.nlm.nih.gov/entrez/query.fcgi?cmd=search&db=gene&term=FAM189A1) | | family with sequence similarity 189, member A1 | 6.34e-05 | 28.43 | 14.85 | 1.91 |
| [218829_s_at](https://www.affymetrix.com/LinkServlet?probeset=218829_s_at) | [CHD7](http://www.ncbi.nlm.nih.gov/entrez/query.fcgi?cmd=search&db=gene&term=CHD7) | | chromodomain helicase DNA binding protein 7 | 6.39e-05 | 472.03 | 807.19 | 0.58 |
| [1552798_a_at](https://www.affymetrix.com/LinkServlet?probeset=1552798_a_at) | [TLR4](http://www.ncbi.nlm.nih.gov/entrez/query.fcgi?cmd=search&db=gene&term=TLR4) | | toll-like receptor 4 | 6.42e-05 | 12.6 | 7.5 | 1.68 |
| [223611_s_at](https://www.affymetrix.com/LinkServlet?probeset=223611_s_at) | [LNX1](http://www.ncbi.nlm.nih.gov/entrez/query.fcgi?cmd=search&db=gene&term=LNX1) | | ligand of numb-protein X 1 | 6.44e-05 | 186.52 | 899.21 | 0.21 |
| [224831_at](https://www.affymetrix.com/LinkServlet?probeset=224831_at) | [CPEB4](http://www.ncbi.nlm.nih.gov/entrez/query.fcgi?cmd=search&db=gene&term=CPEB4) | | cytoplasmic polyadenylation element binding protein 4 | 6.57e-05 | 1071.78 | 1729.4 | 0.62 |
| [203126_at](https://www.affymetrix.com/LinkServlet?probeset=203126_at) | [IMPA2](http://www.ncbi.nlm.nih.gov/entrez/query.fcgi?cmd=search&db=gene&term=IMPA2) | | inositol(myo)-1(or 4)-monophosphatase 2 | 6.62e-05 | 35.01 | 16.23 | 2.16 |
| [225445_at](https://www.affymetrix.com/LinkServlet?probeset=225445_at) | [UBN2](http://www.ncbi.nlm.nih.gov/entrez/query.fcgi?cmd=search&db=gene&term=UBN2) | | ubinuclein 2 | 6.66e-05 | 261.84 | 458.35 | 0.57 |
| [204897_at](https://www.affymetrix.com/LinkServlet?probeset=204897_at) | [PTGER4](http://www.ncbi.nlm.nih.gov/entrez/query.fcgi?cmd=search&db=gene&term=PTGER4) | | prostaglandin E receptor 4 (subtype EP4) | 6.73e-05 | 617.53 | 201.86 | 3.06 |
| [236536_at](https://www.affymetrix.com/LinkServlet?probeset=236536_at) | [GALNT13](http://www.ncbi.nlm.nih.gov/entrez/query.fcgi?cmd=search&db=gene&term=GALNT13) | | UDP-N-acetyl-alpha-D-galactosamine | 6.77e-05 | 53.01 | 176.73 | 0.3 |
| [217004_s_at](https://www.affymetrix.com/LinkServlet?probeset=217004_s_at) | [MCF2](http://www.ncbi.nlm.nih.gov/entrez/query.fcgi?cmd=search&db=gene&term=MCF2) | | MCF.2 cell line derived transforming sequence | 6.78e-05 | 8.69 | 18.72 | 0.46 |
| [218223_s_at](https://www.affymetrix.com/LinkServlet?probeset=218223_s_at) | [PLEKHO1](http://www.ncbi.nlm.nih.gov/entrez/query.fcgi?cmd=search&db=gene&term=PLEKHO1) | | pleckstrin homology domain containing, family O member 1 | 6.82e-05 | 370.97 | 259.04 | 1.43 |
| [211744_s_at](https://www.affymetrix.com/LinkServlet?probeset=211744_s_at) | [CD58](http://www.ncbi.nlm.nih.gov/entrez/query.fcgi?cmd=search&db=gene&term=CD58) | | CD58 molecule | 6.88e-05 | 291.96 | 95.25 | 3.07 |
| [205363_at](https://www.affymetrix.com/LinkServlet?probeset=205363_at) | [BBOX1](http://www.ncbi.nlm.nih.gov/entrez/query.fcgi?cmd=search&db=gene&term=BBOX1) | | butyrobetaine (gamma), 2-oxoglutarate dioxygenase | 7.08e-05 | 120.94 | 555.36 | 0.22 |
| [219263_at](https://www.affymetrix.com/LinkServlet?probeset=219263_at) | [RNF128](http://www.ncbi.nlm.nih.gov/entrez/query.fcgi?cmd=search&db=gene&term=RNF128) | | ring finger protein 128 | 7.11e-05 | 127.12 | 622.73 | 0.2 |
| [226525_at](https://www.affymetrix.com/LinkServlet?probeset=226525_at) | [STK17B](http://www.ncbi.nlm.nih.gov/entrez/query.fcgi?cmd=search&db=gene&term=STK17B) | | serine/threonine kinase 17b | 7.11e-05 | 219.6 | 383.76 | 0.57 |
| [237939_at](https://www.affymetrix.com/LinkServlet?probeset=237939_at) | [EPHA5](http://www.ncbi.nlm.nih.gov/entrez/query.fcgi?cmd=search&db=gene&term=EPHA5) | | EPH receptor A5 | 7.17e-05 | 23.82 | 110.24 | 0.22 |
| [239272_at](https://www.affymetrix.com/LinkServlet?probeset=239272_at) | [MMP28](http://www.ncbi.nlm.nih.gov/entrez/query.fcgi?cmd=search&db=gene&term=MMP28) | | matrix metallopeptidase 28 | 7.18e-05 | 19.75 | 8.52 | 2.32 |
| [236373_at](https://www.affymetrix.com/LinkServlet?probeset=236373_at) | [NA](http://www.ncbi.nlm.nih.gov/entrez/query.fcgi?cmd=search&db=gene&term=NA) | | NA | 7.21e-05 | 40.88 | 185.58 | 0.22 |
| [222871_at](https://www.affymetrix.com/LinkServlet?probeset=222871_at) | [KLHDC8A](http://www.ncbi.nlm.nih.gov/entrez/query.fcgi?cmd=search&db=gene&term=KLHDC8A) | | kelch domain containing 8A | 7.24e-05 | 362.4 | 141.94 | 2.55 |
| [212538_at](https://www.affymetrix.com/LinkServlet?probeset=212538_at) | [DOCK9](http://www.ncbi.nlm.nih.gov/entrez/query.fcgi?cmd=search&db=gene&term=DOCK9) | | dedicator of cytokinesis 9 | 7.26e-05 | 419.89 | 829.8 | 0.51 |
| [1553720_a_at](https://www.affymetrix.com/LinkServlet?probeset=1553720_a_at) | [FAM123A](http://www.ncbi.nlm.nih.gov/entrez/query.fcgi?cmd=search&db=gene&term=FAM123A) | | family with sequence similarity 123A | 7.26e-05 | 137.7 | 375.54 | 0.37 |
| [231015_at](https://www.affymetrix.com/LinkServlet?probeset=231015_at) | [KLF15](http://www.ncbi.nlm.nih.gov/entrez/query.fcgi?cmd=search&db=gene&term=KLF15) | | Kruppel-like factor 15 | 7.3e-05 | 180.18 | 387.21 | 0.47 |
| [204602_at](https://www.affymetrix.com/LinkServlet?probeset=204602_at) | [DKK1](http://www.ncbi.nlm.nih.gov/entrez/query.fcgi?cmd=search&db=gene&term=DKK1) | | dickkopf homolog 1 (Xenopus laevis) | 7.3e-05 | 38.48 | 5.71 | 6.74 |
| [229463_at](https://www.affymetrix.com/LinkServlet?probeset=229463_at) | [NTRK2](http://www.ncbi.nlm.nih.gov/entrez/query.fcgi?cmd=search&db=gene&term=NTRK2) | | neurotrophic tyrosine kinase, receptor, type 2 | 7.41e-05 | 45.23 | 118.47 | 0.38 |
| [238344_at](https://www.affymetrix.com/LinkServlet?probeset=238344_at) | [NA](http://www.ncbi.nlm.nih.gov/entrez/query.fcgi?cmd=search&db=gene&term=NA) | | NA | 7.65e-05 | 52.01 | 175.34 | 0.3 |
| [209839_at](https://www.affymetrix.com/LinkServlet?probeset=209839_at) | [DNM3](http://www.ncbi.nlm.nih.gov/entrez/query.fcgi?cmd=search&db=gene&term=DNM3) | | dynamin 3 | 7.71e-05 | 343.57 | 801.16 | 0.43 |
| [210260_s_at](https://www.affymetrix.com/LinkServlet?probeset=210260_s_at) | [TNFAIP8](http://www.ncbi.nlm.nih.gov/entrez/query.fcgi?cmd=search&db=gene&term=TNFAIP8) | | tumor necrosis factor, alpha-induced protein 8 | 7.74e-05 | 177.13 | 75.85 | 2.34 |
| [207242_s_at](https://www.affymetrix.com/LinkServlet?probeset=207242_s_at) | [GRIK1](http://www.ncbi.nlm.nih.gov/entrez/query.fcgi?cmd=search&db=gene&term=GRIK1) | | glutamate receptor, ionotropic, kainate 1 | 7.78e-05 | 68.47 | 191.31 | 0.36 |
| [206309_at](https://www.affymetrix.com/LinkServlet?probeset=206309_at) | [LECT1](http://www.ncbi.nlm.nih.gov/entrez/query.fcgi?cmd=search&db=gene&term=LECT1) | | leukocyte cell derived chemotaxin 1 | 7.8e-05 | 14.09 | 35.62 | 0.4 |
| [1569178_at](https://www.affymetrix.com/LinkServlet?probeset=1569178_at) | [GRIA4](http://www.ncbi.nlm.nih.gov/entrez/query.fcgi?cmd=search&db=gene&term=GRIA4) | | glutamate receptor, ionotrophic, AMPA 4 | 7.85e-05 | 29.41 | 82.3 | 0.36 |
| [219406_at](https://www.affymetrix.com/LinkServlet?probeset=219406_at) | [C1orf50](http://www.ncbi.nlm.nih.gov/entrez/query.fcgi?cmd=search&db=gene&term=C1orf50) | | chromosome 1 open reading frame 50 | 7.88e-05 | 159.48 | 108.44 | 1.47 |
| [224997_x_at](https://www.affymetrix.com/LinkServlet?probeset=224997_x_at) | [H19](http://www.ncbi.nlm.nih.gov/entrez/query.fcgi?cmd=search&db=gene&term=H19) | | H19, imprinted maternally expressed transcript (non-protein coding) | 7.94e-05 | 6.46 | 19.92 | 0.32 |
| [1566638_at](https://www.affymetrix.com/LinkServlet?probeset=1566638_at) | [NA](http://www.ncbi.nlm.nih.gov/entrez/query.fcgi?cmd=search&db=gene&term=NA) | | NA | 7.94e-05 | 15.27 | 70.55 | 0.22 |
| [204741_at](https://www.affymetrix.com/LinkServlet?probeset=204741_at) | [BICD1](http://www.ncbi.nlm.nih.gov/entrez/query.fcgi?cmd=search&db=gene&term=BICD1) | | bicaudal D homolog 1 (Drosophila) | 7.96e-05 | 179.71 | 111.56 | 1.61 |
| [214954_at](https://www.affymetrix.com/LinkServlet?probeset=214954_at) | [SUSD5](http://www.ncbi.nlm.nih.gov/entrez/query.fcgi?cmd=search&db=gene&term=SUSD5) | | sushi domain containing 5 | 8e-05 | 415.27 | 1603.52 | 0.26 |
| [228444_at](https://www.affymetrix.com/LinkServlet?probeset=228444_at) | [NA](http://www.ncbi.nlm.nih.gov/entrez/query.fcgi?cmd=search&db=gene&term=NA) | | NA | 8.02e-05 | 23.7 | 15.4 | 1.54 |
| [213373_s_at](https://www.affymetrix.com/LinkServlet?probeset=213373_s_at) | [CASP8](http://www.ncbi.nlm.nih.gov/entrez/query.fcgi?cmd=search&db=gene&term=CASP8) | | caspase 8, apoptosis-related cysteine peptidase | 8.28e-05 | 148.92 | 74.12 | 2.01 |
| [1563217_at](https://www.affymetrix.com/LinkServlet?probeset=1563217_at) | [NA](http://www.ncbi.nlm.nih.gov/entrez/query.fcgi?cmd=search&db=gene&term=NA) | | NA | 8.29e-05 | 22.55 | 70.11 | 0.32 |
| [203608_at](https://www.affymetrix.com/LinkServlet?probeset=203608_at) | [ALDH5A1](http://www.ncbi.nlm.nih.gov/entrez/query.fcgi?cmd=search&db=gene&term=ALDH5A1) | | aldehyde dehydrogenase 5 family, member A1 | 8.29e-05 | 946.91 | 1668.17 | 0.57 |
| [225384_at](https://www.affymetrix.com/LinkServlet?probeset=225384_at) | [DOCK7](http://www.ncbi.nlm.nih.gov/entrez/query.fcgi?cmd=search&db=gene&term=DOCK7) | | dedicator of cytokinesis 7 | 8.37e-05 | 965.31 | 470.82 | 2.05 |
| [207542_s_at](https://www.affymetrix.com/LinkServlet?probeset=207542_s_at) | [AQP1](http://www.ncbi.nlm.nih.gov/entrez/query.fcgi?cmd=search&db=gene&term=AQP1) | | aquaporin 1 (Colton blood group) | 8.58e-05 | 32.88 | 188.59 | 0.17 |
| [206960_at](https://www.affymetrix.com/LinkServlet?probeset=206960_at) | [LPAR4](http://www.ncbi.nlm.nih.gov/entrez/query.fcgi?cmd=search&db=gene&term=LPAR4) | | lysophosphatidic acid receptor 4 | 8.65e-05 | 74.85 | 226.76 | 0.33 |
| [242532_at](https://www.affymetrix.com/LinkServlet?probeset=242532_at) | [NA](http://www.ncbi.nlm.nih.gov/entrez/query.fcgi?cmd=search&db=gene&term=NA) | | NA | 8.66e-05 | 20 | 85.73 | 0.23 |
| [219691_at](https://www.affymetrix.com/LinkServlet?probeset=219691_at) | [SAMD9](http://www.ncbi.nlm.nih.gov/entrez/query.fcgi?cmd=search&db=gene&term=SAMD9) | | sterile alpha motif domain containing 9 | 8.69e-05 | 53.34 | 29.36 | 1.82 |
| [232282_at](https://www.affymetrix.com/LinkServlet?probeset=232282_at) | [WNK3](http://www.ncbi.nlm.nih.gov/entrez/query.fcgi?cmd=search&db=gene&term=WNK3) | | WNK lysine deficient protein kinase 3 | 8.87e-05 | 198 | 392.07 | 0.51 |
| [226682_at](https://www.affymetrix.com/LinkServlet?probeset=226682_at) | [RORA](http://www.ncbi.nlm.nih.gov/entrez/query.fcgi?cmd=search&db=gene&term=RORA) | | RAR-related orphan receptor A | 8.97e-05 | 533.37 | 1112.55 | 0.48 |
| [204341_at](https://www.affymetrix.com/LinkServlet?probeset=204341_at) | [TRIM16](http://www.ncbi.nlm.nih.gov/entrez/query.fcgi?cmd=search&db=gene&term=TRIM16) | | tripartite motif-containing 16 | 9.06e-05 | 75.55 | 43.03 | 1.76 |
| [225292_at](https://www.affymetrix.com/LinkServlet?probeset=225292_at) | [COL27A1](http://www.ncbi.nlm.nih.gov/entrez/query.fcgi?cmd=search&db=gene&term=COL27A1) | | collagen, type XXVII, alpha 1 | 9.16e-05 | 29.22 | 61.92 | 0.47 |
| [206690_at](https://www.affymetrix.com/LinkServlet?probeset=206690_at) | [ACCN1](http://www.ncbi.nlm.nih.gov/entrez/query.fcgi?cmd=search&db=gene&term=ACCN1) | | amiloride-sensitive cation channel 1, neuronal | 9.45e-05 | 40.7 | 92.63 | 0.44 |
| [204811_s_at](https://www.affymetrix.com/LinkServlet?probeset=204811_s_at) | [CACNA2D2](http://www.ncbi.nlm.nih.gov/entrez/query.fcgi?cmd=search&db=gene&term=CACNA2D2) | | calcium channel, voltage-dependent, alpha 2/delta subunit 2 | 9.46e-05 | 23.87 | 60.75 | 0.39 |
| [230773_at](https://www.affymetrix.com/LinkServlet?probeset=230773_at) | [NA](http://www.ncbi.nlm.nih.gov/entrez/query.fcgi?cmd=search&db=gene&term=NA) | | NA | 9.68e-05 | 30.47 | 72.84 | 0.42 |
| [206342_x_at](https://www.affymetrix.com/LinkServlet?probeset=206342_x_at) | [IDS](http://www.ncbi.nlm.nih.gov/entrez/query.fcgi?cmd=search&db=gene&term=IDS) | | iduronate 2-sulfatase | 9.71e-05 | 120.01 | 69.8 | 1.72 |
| [228821_at](https://www.affymetrix.com/LinkServlet?probeset=228821_at) | [ST6GAL2](http://www.ncbi.nlm.nih.gov/entrez/query.fcgi?cmd=search&db=gene&term=ST6GAL2) | | ST6 beta-galactosamide alpha-2,6-sialyltranferase 2 | 9.77e-05 | 20.97 | 115.09 | 0.18 |
| [209243_s_at](https://www.affymetrix.com/LinkServlet?probeset=209243_s_at) | [NA](http://www.ncbi.nlm.nih.gov/entrez/query.fcgi?cmd=search&db=gene&term=NA) | | NA | 9.8e-05 | 632.9 | 1561.08 | 0.41 |
| [239155_at](https://www.affymetrix.com/LinkServlet?probeset=239155_at) | [NA](http://www.ncbi.nlm.nih.gov/entrez/query.fcgi?cmd=search&db=gene&term=NA) | | NA | 9.81e-05 | 43.48 | 15.75 | 2.76 |
| [226690_at](https://www.affymetrix.com/LinkServlet?probeset=226690_at) | [ADCYAP1R1](http://www.ncbi.nlm.nih.gov/entrez/query.fcgi?cmd=search&db=gene&term=ADCYAP1R1) | | adenylate cyclase activating polypeptide 1 (pituitary) receptor type I | 9.83e-05 | 227.39 | 838.03 | 0.27 |
| [221900_at](https://www.affymetrix.com/LinkServlet?probeset=221900_at) | [COL8A2](http://www.ncbi.nlm.nih.gov/entrez/query.fcgi?cmd=search&db=gene&term=COL8A2) | | collagen, type VIII, alpha 2 | 0.0001002 | 41.95 | 132.65 | 0.32 |
| [215983_s_at](https://www.affymetrix.com/LinkServlet?probeset=215983_s_at) | [UBXN8](http://www.ncbi.nlm.nih.gov/entrez/query.fcgi?cmd=search&db=gene&term=UBXN8) | | UBX domain protein 8 | 0.0001006 | 258.95 | 134.64 | 1.92 |
| [203408_s_at](https://www.affymetrix.com/LinkServlet?probeset=203408_s_at) | [SATB1](http://www.ncbi.nlm.nih.gov/entrez/query.fcgi?cmd=search&db=gene&term=SATB1) | | SATB homeobox 1 | 0.0001015 | 1057.08 | 2247.3 | 0.47 |
| [215134_at](https://www.affymetrix.com/LinkServlet?probeset=215134_at) | [PI4K2A](http://www.ncbi.nlm.nih.gov/entrez/query.fcgi?cmd=search&db=gene&term=PI4K2A) | | phosphatidylinositol 4-kinase type 2 alpha | 0.0001032 | 14.84 | 9.5 | 1.56 |
| [223612_s_at](https://www.affymetrix.com/LinkServlet?probeset=223612_s_at) | [LNX1](http://www.ncbi.nlm.nih.gov/entrez/query.fcgi?cmd=search&db=gene&term=LNX1) | | ligand of numb-protein X 1 | 0.0001055 | 27.23 | 73.53 | 0.37 |
| [202781_s_at](https://www.affymetrix.com/LinkServlet?probeset=202781_s_at) | [INPP5K](http://www.ncbi.nlm.nih.gov/entrez/query.fcgi?cmd=search&db=gene&term=INPP5K) | | inositol polyphosphate-5-phosphatase K | 0.0001059 | 29.11 | 19.92 | 1.46 |
| [220311_at](https://www.affymetrix.com/LinkServlet?probeset=220311_at) | [N6AMT1](http://www.ncbi.nlm.nih.gov/entrez/query.fcgi?cmd=search&db=gene&term=N6AMT1) | | N-6 adenine-specific DNA methyltransferase 1 (putative) | 0.000106 | 35.85 | 18.63 | 1.92 |
| [219926_at](https://www.affymetrix.com/LinkServlet?probeset=219926_at) | [POPDC3](http://www.ncbi.nlm.nih.gov/entrez/query.fcgi?cmd=search&db=gene&term=POPDC3) | | popeye domain containing 3 | 0.0001088 | 45.99 | 9.91 | 4.64 |
| [203570_at](https://www.affymetrix.com/LinkServlet?probeset=203570_at) | [LOXL1](http://www.ncbi.nlm.nih.gov/entrez/query.fcgi?cmd=search&db=gene&term=LOXL1) | | lysyl oxidase-like 1 | 0.0001096 | 186.57 | 52.52 | 3.55 |
| [241404_at](https://www.affymetrix.com/LinkServlet?probeset=241404_at) | [NA](http://www.ncbi.nlm.nih.gov/entrez/query.fcgi?cmd=search&db=gene&term=NA) | | NA | 0.0001098 | 10.12 | 26.67 | 0.38 |
| [205051_s_at](https://www.affymetrix.com/LinkServlet?probeset=205051_s_at) | [KIT](http://www.ncbi.nlm.nih.gov/entrez/query.fcgi?cmd=search&db=gene&term=KIT) | | v-kit Hardy-Zuckerman 4 feline sarcoma viral oncogene homolog | 0.0001101 | 60.43 | 217.11 | 0.28 |
| [229065_at](https://www.affymetrix.com/LinkServlet?probeset=229065_at) | [SLC35F3](http://www.ncbi.nlm.nih.gov/entrez/query.fcgi?cmd=search&db=gene&term=SLC35F3) | | solute carrier family 35, member F3 | 0.0001111 | 28.4 | 91.66 | 0.31 |
| [226548_at](https://www.affymetrix.com/LinkServlet?probeset=226548_at) | [SBK1](http://www.ncbi.nlm.nih.gov/entrez/query.fcgi?cmd=search&db=gene&term=SBK1) | | SH3-binding domain kinase 1 | 0.0001114 | 41.36 | 108.53 | 0.38 |
| [238669_at](https://www.affymetrix.com/LinkServlet?probeset=238669_at) | [PTGS1](http://www.ncbi.nlm.nih.gov/entrez/query.fcgi?cmd=search&db=gene&term=PTGS1) | | prostaglandin-endoperoxide synthase 1 (prostaglandin G/H synthase and cyclooxygenase) | 0.0001116 | 105.88 | 60.18 | 1.76 |
| [243929_at](https://www.affymetrix.com/LinkServlet?probeset=243929_at) | [NA](http://www.ncbi.nlm.nih.gov/entrez/query.fcgi?cmd=search&db=gene&term=NA) | | NA | 0.000113 | 5.12 | 25.64 | 0.2 |
| [223843_at](https://www.affymetrix.com/LinkServlet?probeset=223843_at) | [SCARA3](http://www.ncbi.nlm.nih.gov/entrez/query.fcgi?cmd=search&db=gene&term=SCARA3) | | scavenger receptor class A, member 3 | 0.0001133 | 77.36 | 221.79 | 0.35 |
| [225666_at](https://www.affymetrix.com/LinkServlet?probeset=225666_at) | [TMTC4](http://www.ncbi.nlm.nih.gov/entrez/query.fcgi?cmd=search&db=gene&term=TMTC4) | | transmembrane and tetratricopeptide repeat containing 4 | 0.0001134 | 416.36 | 166.8 | 2.5 |
| [228910_at](https://www.affymetrix.com/LinkServlet?probeset=228910_at) | [NA](http://www.ncbi.nlm.nih.gov/entrez/query.fcgi?cmd=search&db=gene&term=NA) | | NA | 0.0001152 | 92.8 | 251.47 | 0.37 |
| [203299_s_at](https://www.affymetrix.com/LinkServlet?probeset=203299_s_at) | [AP1S2](http://www.ncbi.nlm.nih.gov/entrez/query.fcgi?cmd=search&db=gene&term=AP1S2) | | adaptor-related protein complex 1, sigma 2 subunit | 0.0001158 | 785.48 | 400.77 | 1.96 |
| [219440_at](https://www.affymetrix.com/LinkServlet?probeset=219440_at) | [RAI2](http://www.ncbi.nlm.nih.gov/entrez/query.fcgi?cmd=search&db=gene&term=RAI2) | | retinoic acid induced 2 | 0.0001164 | 95.4 | 199.26 | 0.48 |
| [231856_at](https://www.affymetrix.com/LinkServlet?probeset=231856_at) | [KIAA1244](http://www.ncbi.nlm.nih.gov/entrez/query.fcgi?cmd=search&db=gene&term=KIAA1244) | | KIAA1244 | 0.0001171 | 83.41 | 174.65 | 0.48 |
| [209665_at](https://www.affymetrix.com/LinkServlet?probeset=209665_at) | [CYB561D2](http://www.ncbi.nlm.nih.gov/entrez/query.fcgi?cmd=search&db=gene&term=CYB561D2) | | cytochrome b-561 domain containing 2 | 0.0001171 | 114.55 | 73.21 | 1.56 |
| [205738_s_at](https://www.affymetrix.com/LinkServlet?probeset=205738_s_at) | [FABP3](http://www.ncbi.nlm.nih.gov/entrez/query.fcgi?cmd=search&db=gene&term=FABP3) | | fatty acid binding protein 3, muscle and heart (mammary-derived growth inhibitor) | 0.000119 | 79.73 | 28.5 | 2.8 |
| [207103_at](https://www.affymetrix.com/LinkServlet?probeset=207103_at) | [KCND2](http://www.ncbi.nlm.nih.gov/entrez/query.fcgi?cmd=search&db=gene&term=KCND2) | | potassium voltage-gated channel, Shal-related subfamily, member 2 | 0.0001191 | 163.33 | 773.52 | 0.21 |
| [242228_at](https://www.affymetrix.com/LinkServlet?probeset=242228_at) | [NA](http://www.ncbi.nlm.nih.gov/entrez/query.fcgi?cmd=search&db=gene&term=NA) | | NA | 0.000121 | 87.11 | 48.66 | 1.79 |
| [226137_at](https://www.affymetrix.com/LinkServlet?probeset=226137_at) | [ZFHX3](http://www.ncbi.nlm.nih.gov/entrez/query.fcgi?cmd=search&db=gene&term=ZFHX3) | | zinc finger homeobox 3 | 0.0001222 | 502.91 | 305.29 | 1.65 |
| [206018_at](https://www.affymetrix.com/LinkServlet?probeset=206018_at) | [FOXG1](http://www.ncbi.nlm.nih.gov/entrez/query.fcgi?cmd=search&db=gene&term=FOXG1) | | forkhead box G1 | 0.0001242 | 91.91 | 4.93 | 18.63 |
| [218589_at](https://www.affymetrix.com/LinkServlet?probeset=218589_at) | [LPAR6](http://www.ncbi.nlm.nih.gov/entrez/query.fcgi?cmd=search&db=gene&term=LPAR6) | | lysophosphatidic acid receptor 6 | 0.0001246 | 711.57 | 281.01 | 2.53 |
| [239671_at](https://www.affymetrix.com/LinkServlet?probeset=239671_at) | [NA](http://www.ncbi.nlm.nih.gov/entrez/query.fcgi?cmd=search&db=gene&term=NA) | | NA | 0.0001257 | 132.18 | 446.14 | 0.3 |
| [204831_at](https://www.affymetrix.com/LinkServlet?probeset=204831_at) | [CDK8](http://www.ncbi.nlm.nih.gov/entrez/query.fcgi?cmd=search&db=gene&term=CDK8) | | cyclin-dependent kinase 8 | 0.0001257 | 199.21 | 399.47 | 0.5 |
| [222609_s_at](https://www.affymetrix.com/LinkServlet?probeset=222609_s_at) | [EXOSC1](http://www.ncbi.nlm.nih.gov/entrez/query.fcgi?cmd=search&db=gene&term=EXOSC1) | | exosome component 1 | 0.0001257 | 321.29 | 215.07 | 1.49 |
| [213362_at](https://www.affymetrix.com/LinkServlet?probeset=213362_at) | [PTPRD](http://www.ncbi.nlm.nih.gov/entrez/query.fcgi?cmd=search&db=gene&term=PTPRD) | | protein tyrosine phosphatase, receptor type, D | 0.0001275 | 172.23 | 442.37 | 0.39 |
| [218935_at](https://www.affymetrix.com/LinkServlet?probeset=218935_at) | [EHD3](http://www.ncbi.nlm.nih.gov/entrez/query.fcgi?cmd=search&db=gene&term=EHD3) | | EH-domain containing 3 | 0.0001303 | 448.95 | 1087.07 | 0.41 |
| [204689_at](https://www.affymetrix.com/LinkServlet?probeset=204689_at) | [HHEX](http://www.ncbi.nlm.nih.gov/entrez/query.fcgi?cmd=search&db=gene&term=HHEX) | | hematopoietically expressed homeobox | 0.0001306 | 51.41 | 27.94 | 1.84 |
| [204447_at](https://www.affymetrix.com/LinkServlet?probeset=204447_at) | [ProSAPiP1](http://www.ncbi.nlm.nih.gov/entrez/query.fcgi?cmd=search&db=gene&term=ProSAPiP1) | | ProSAPiP1 protein | 0.0001329 | 249.57 | 527.53 | 0.47 |
| [218409_s_at](https://www.affymetrix.com/LinkServlet?probeset=218409_s_at) | [DNAJC1](http://www.ncbi.nlm.nih.gov/entrez/query.fcgi?cmd=search&db=gene&term=DNAJC1) | | DnaJ (Hsp40) homolog, subfamily C, member 1 | 0.000133 | 356.35 | 212.37 | 1.68 |
| [222957_at](https://www.affymetrix.com/LinkServlet?probeset=222957_at) | [NEU4](http://www.ncbi.nlm.nih.gov/entrez/query.fcgi?cmd=search&db=gene&term=NEU4) | | sialidase 4 | 0.0001346 | 72.21 | 218.21 | 0.33 |
| [229584_at](https://www.affymetrix.com/LinkServlet?probeset=229584_at) | [LRRK2](http://www.ncbi.nlm.nih.gov/entrez/query.fcgi?cmd=search&db=gene&term=LRRK2) | | leucine-rich repeat kinase 2 | 0.0001379 | 320.61 | 730.74 | 0.44 |
| [206584_at](https://www.affymetrix.com/LinkServlet?probeset=206584_at) | [LY96](http://www.ncbi.nlm.nih.gov/entrez/query.fcgi?cmd=search&db=gene&term=LY96) | | lymphocyte antigen 96 | 0.0001379 | 827.73 | 350.94 | 2.36 |
| [242826_at](https://www.affymetrix.com/LinkServlet?probeset=242826_at) | [NA](http://www.ncbi.nlm.nih.gov/entrez/query.fcgi?cmd=search&db=gene&term=NA) | | NA | 0.0001382 | 127.85 | 212.85 | 0.6 |
| [210479_s_at](https://www.affymetrix.com/LinkServlet?probeset=210479_s_at) | [RORA](http://www.ncbi.nlm.nih.gov/entrez/query.fcgi?cmd=search&db=gene&term=RORA) | | RAR-related orphan receptor A | 0.0001398 | 39.37 | 123.29 | 0.32 |
| [230362_at](https://www.affymetrix.com/LinkServlet?probeset=230362_at) | [INPP5F](http://www.ncbi.nlm.nih.gov/entrez/query.fcgi?cmd=search&db=gene&term=INPP5F) | | inositol polyphosphate-5-phosphatase F | 0.0001402 | 46.93 | 22.29 | 2.11 |
| [226158_at](https://www.affymetrix.com/LinkServlet?probeset=226158_at) | [KLHL24](http://www.ncbi.nlm.nih.gov/entrez/query.fcgi?cmd=search&db=gene&term=KLHL24) | | kelch-like 24 (Drosophila) | 0.0001425 | 711.96 | 1029 | 0.69 |
| [235419_at](https://www.affymetrix.com/LinkServlet?probeset=235419_at) | [NA](http://www.ncbi.nlm.nih.gov/entrez/query.fcgi?cmd=search&db=gene&term=NA) | | NA | 0.0001439 | 15.2 | 41.31 | 0.37 |
| [206715_at](https://www.affymetrix.com/LinkServlet?probeset=206715_at) | [TFEC](http://www.ncbi.nlm.nih.gov/entrez/query.fcgi?cmd=search&db=gene&term=TFEC) | | transcription factor EC | 0.000144 | 50.15 | 16.33 | 3.07 |
| [204040_at](https://www.affymetrix.com/LinkServlet?probeset=204040_at) | [RNF144A](http://www.ncbi.nlm.nih.gov/entrez/query.fcgi?cmd=search&db=gene&term=RNF144A) | | ring finger protein 144A | 0.0001461 | 181.29 | 407.46 | 0.44 |
| [228531_at](https://www.affymetrix.com/LinkServlet?probeset=228531_at) | [SAMD9](http://www.ncbi.nlm.nih.gov/entrez/query.fcgi?cmd=search&db=gene&term=SAMD9) | | sterile alpha motif domain containing 9 | 0.0001462 | 227.02 | 125.18 | 1.81 |
| [225579_at](https://www.affymetrix.com/LinkServlet?probeset=225579_at) | [PQLC3](http://www.ncbi.nlm.nih.gov/entrez/query.fcgi?cmd=search&db=gene&term=PQLC3) | | PQ loop repeat containing 3 | 0.0001474 | 429.27 | 197.98 | 2.17 |
| [224916_at](https://www.affymetrix.com/LinkServlet?probeset=224916_at) | [TMEM173](http://www.ncbi.nlm.nih.gov/entrez/query.fcgi?cmd=search&db=gene&term=TMEM173) | | transmembrane protein 173 | 0.0001483 | 18.24 | 11.54 | 1.58 |
| [213418_at](https://www.affymetrix.com/LinkServlet?probeset=213418_at) | [HSPA6](http://www.ncbi.nlm.nih.gov/entrez/query.fcgi?cmd=search&db=gene&term=HSPA6) | | heat shock 70kDa protein 6 (HSP70B') | 0.0001489 | 140.02 | 44.64 | 3.14 |
| [225293_at](https://www.affymetrix.com/LinkServlet?probeset=225293_at) | [COL27A1](http://www.ncbi.nlm.nih.gov/entrez/query.fcgi?cmd=search&db=gene&term=COL27A1) | | collagen, type XXVII, alpha 1 | 0.0001494 | 291.76 | 725.52 | 0.4 |
| [230363_s_at](https://www.affymetrix.com/LinkServlet?probeset=230363_s_at) | [INPP5F](http://www.ncbi.nlm.nih.gov/entrez/query.fcgi?cmd=search&db=gene&term=INPP5F) | | inositol polyphosphate-5-phosphatase F | 0.00015 | 138.57 | 71.36 | 1.94 |
| [1558692_at](https://www.affymetrix.com/LinkServlet?probeset=1558692_at) | [C1orf85](http://www.ncbi.nlm.nih.gov/entrez/query.fcgi?cmd=search&db=gene&term=C1orf85) | | chromosome 1 open reading frame 85 | 0.0001551 | 217.69 | 130.21 | 1.67 |
| [219905_at](https://www.affymetrix.com/LinkServlet?probeset=219905_at) | [ERMAP](http://www.ncbi.nlm.nih.gov/entrez/query.fcgi?cmd=search&db=gene&term=ERMAP) | | erythroblast membrane-associated protein (Scianna blood group) | 0.0001568 | 86.8 | 41.45 | 2.09 |
| [236045_x_at](https://www.affymetrix.com/LinkServlet?probeset=236045_x_at) | [NA](http://www.ncbi.nlm.nih.gov/entrez/query.fcgi?cmd=search&db=gene&term=NA) | | NA | 0.0001573 | 27.61 | 103.05 | 0.27 |
| [228347_at](https://www.affymetrix.com/LinkServlet?probeset=228347_at) | [SIX1](http://www.ncbi.nlm.nih.gov/entrez/query.fcgi?cmd=search&db=gene&term=SIX1) | | SIX homeobox 1 | 0.0001602 | 90.5 | 9.17 | 9.87 |
| [205905_s_at](https://www.affymetrix.com/LinkServlet?probeset=205905_s_at) | [NA](http://www.ncbi.nlm.nih.gov/entrez/query.fcgi?cmd=search&db=gene&term=NA) | | NA | 0.0001616 | 20.41 | 12.05 | 1.69 |
| [223282_at](https://www.affymetrix.com/LinkServlet?probeset=223282_at) | [TSHZ1](http://www.ncbi.nlm.nih.gov/entrez/query.fcgi?cmd=search&db=gene&term=TSHZ1) | | teashirt zinc finger homeobox 1 | 0.000162 | 920.78 | 1479.27 | 0.62 |
| [204069_at](https://www.affymetrix.com/LinkServlet?probeset=204069_at) | [MEIS1](http://www.ncbi.nlm.nih.gov/entrez/query.fcgi?cmd=search&db=gene&term=MEIS1) | | Meis homeobox 1 | 0.000162 | 143.92 | 330.35 | 0.44 |
| [210319_x_at](https://www.affymetrix.com/LinkServlet?probeset=210319_x_at) | [MSX2](http://www.ncbi.nlm.nih.gov/entrez/query.fcgi?cmd=search&db=gene&term=MSX2) | | msh homeobox 2 | 0.000163 | 7.06 | 19.02 | 0.37 |
| [218844_at](https://www.affymetrix.com/LinkServlet?probeset=218844_at) | [ACSF2](http://www.ncbi.nlm.nih.gov/entrez/query.fcgi?cmd=search&db=gene&term=ACSF2) | | acyl-CoA synthetase family member 2 | 0.0001654 | 92.22 | 247.87 | 0.37 |
| [213479_at](https://www.affymetrix.com/LinkServlet?probeset=213479_at) | [NPTX2](http://www.ncbi.nlm.nih.gov/entrez/query.fcgi?cmd=search&db=gene&term=NPTX2) | | neuronal pentraxin II | 0.0001659 | 1124.85 | 128.98 | 8.72 |
| [201486_at](https://www.affymetrix.com/LinkServlet?probeset=201486_at) | [RCN2](http://www.ncbi.nlm.nih.gov/entrez/query.fcgi?cmd=search&db=gene&term=RCN2) | | reticulocalbin 2, EF-hand calcium binding domain | 0.0001695 | 2757.63 | 1637.99 | 1.68 |
| [227145_at](https://www.affymetrix.com/LinkServlet?probeset=227145_at) | [LOXL4](http://www.ncbi.nlm.nih.gov/entrez/query.fcgi?cmd=search&db=gene&term=LOXL4) | | lysyl oxidase-like 4 | 0.0001707 | 20.26 | 8.64 | 2.34 |
| [1556627_at](https://www.affymetrix.com/LinkServlet?probeset=1556627_at) | [DRP2](http://www.ncbi.nlm.nih.gov/entrez/query.fcgi?cmd=search&db=gene&term=DRP2) | | dystrophin related protein 2 | 0.0001733 | 23.47 | 62.36 | 0.38 |
| [219119_at](https://www.affymetrix.com/LinkServlet?probeset=219119_at) | [LSM8](http://www.ncbi.nlm.nih.gov/entrez/query.fcgi?cmd=search&db=gene&term=LSM8) | | LSM8 homolog, U6 small nuclear RNA associated (S. cerevisiae) | 0.0001758 | 563.2 | 338.67 | 1.66 |
| [228051_at](https://www.affymetrix.com/LinkServlet?probeset=228051_at) | [LOC202451](http://www.ncbi.nlm.nih.gov/entrez/query.fcgi?cmd=search&db=gene&term=LOC202451) | | hypothetical protein LOC202451 | 0.0001763 | 209.82 | 441.67 | 0.48 |
| [213600_at](https://www.affymetrix.com/LinkServlet?probeset=213600_at) | [SIPA1L3](http://www.ncbi.nlm.nih.gov/entrez/query.fcgi?cmd=search&db=gene&term=SIPA1L3) | | signal-induced proliferation-associated 1 like 3 | 0.0001772 | 55.64 | 80.46 | 0.69 |
| [218309_at](https://www.affymetrix.com/LinkServlet?probeset=218309_at) | [CAMK2N1](http://www.ncbi.nlm.nih.gov/entrez/query.fcgi?cmd=search&db=gene&term=CAMK2N1) | | calcium/calmodulin-dependent protein kinase II inhibitor 1 | 0.0001781 | 1478.66 | 2373.32 | 0.62 |
| [227792_at](https://www.affymetrix.com/LinkServlet?probeset=227792_at) | [ITPRIPL2](http://www.ncbi.nlm.nih.gov/entrez/query.fcgi?cmd=search&db=gene&term=ITPRIPL2) | | inositol 1,4,5-triphosphate receptor interacting protein-like 2 | 0.0001786 | 1864.92 | 1160.37 | 1.61 |
| [1556629_a_at](https://www.affymetrix.com/LinkServlet?probeset=1556629_a_at) | [SNAP25](http://www.ncbi.nlm.nih.gov/entrez/query.fcgi?cmd=search&db=gene&term=SNAP25) | | synaptosomal-associated protein, 25kDa | 0.0001793 | 37.45 | 125.82 | 0.3 |
| [204932_at](https://www.affymetrix.com/LinkServlet?probeset=204932_at) | [TNFRSF11B](http://www.ncbi.nlm.nih.gov/entrez/query.fcgi?cmd=search&db=gene&term=TNFRSF11B) | | tumor necrosis factor receptor superfamily, member 11b | 0.0001819 | 52.73 | 147.14 | 0.36 |
| [201234_at](https://www.affymetrix.com/LinkServlet?probeset=201234_at) | [ILK](http://www.ncbi.nlm.nih.gov/entrez/query.fcgi?cmd=search&db=gene&term=ILK) | | integrin-linked kinase | 0.0001827 | 203.27 | 129.29 | 1.57 |
| [237665_at](https://www.affymetrix.com/LinkServlet?probeset=237665_at) | [NA](http://www.ncbi.nlm.nih.gov/entrez/query.fcgi?cmd=search&db=gene&term=NA) | | NA | 0.0001838 | 9.39 | 20.59 | 0.46 |
| [239726_at](https://www.affymetrix.com/LinkServlet?probeset=239726_at) | [ANK3](http://www.ncbi.nlm.nih.gov/entrez/query.fcgi?cmd=search&db=gene&term=ANK3) | | ankyrin 3, node of Ranvier (ankyrin G) | 0.0001865 | 49.52 | 154.25 | 0.32 |
| [219837_s_at](https://www.affymetrix.com/LinkServlet?probeset=219837_s_at) | [CYTL1](http://www.ncbi.nlm.nih.gov/entrez/query.fcgi?cmd=search&db=gene&term=CYTL1) | | cytokine-like 1 | 0.0001879 | 123.46 | 34.12 | 3.62 |
| [206243_at](https://www.affymetrix.com/LinkServlet?probeset=206243_at) | [TIMP4](http://www.ncbi.nlm.nih.gov/entrez/query.fcgi?cmd=search&db=gene&term=TIMP4) | | TIMP metallopeptidase inhibitor 4 | 0.0001885 | 776.84 | 1988.13 | 0.39 |
| [236038_at](https://www.affymetrix.com/LinkServlet?probeset=236038_at) | [NA](http://www.ncbi.nlm.nih.gov/entrez/query.fcgi?cmd=search&db=gene&term=NA) | | NA | 0.000189 | 264.92 | 518.75 | 0.51 |
| [207012_at](https://www.affymetrix.com/LinkServlet?probeset=207012_at) | [MMP16](http://www.ncbi.nlm.nih.gov/entrez/query.fcgi?cmd=search&db=gene&term=MMP16) | | matrix metallopeptidase 16 (membrane-inserted) | 0.0001907 | 70.87 | 179.56 | 0.39 |
| [204203_at](https://www.affymetrix.com/LinkServlet?probeset=204203_at) | [CEBPG](http://www.ncbi.nlm.nih.gov/entrez/query.fcgi?cmd=search&db=gene&term=CEBPG) | | CCAAT/enhancer binding protein (C/EBP), gamma | 0.0001916 | 180.13 | 106.05 | 1.7 |
| [239064_at](https://www.affymetrix.com/LinkServlet?probeset=239064_at) | [NA](http://www.ncbi.nlm.nih.gov/entrez/query.fcgi?cmd=search&db=gene&term=NA) | | NA | 0.0001926 | 32.04 | 20.82 | 1.54 |
| [228799_at](https://www.affymetrix.com/LinkServlet?probeset=228799_at) | [NA](http://www.ncbi.nlm.nih.gov/entrez/query.fcgi?cmd=search&db=gene&term=NA) | | NA | 0.0001954 | 73.77 | 34.42 | 2.14 |
| [231867_at](https://www.affymetrix.com/LinkServlet?probeset=231867_at) | [ODZ2](http://www.ncbi.nlm.nih.gov/entrez/query.fcgi?cmd=search&db=gene&term=ODZ2) | | odz, odd Oz/ten-m homolog 2 (Drosophila) | 0.0001963 | 15.05 | 75.35 | 0.2 |
| [213035_at](https://www.affymetrix.com/LinkServlet?probeset=213035_at) | [ANKRD28](http://www.ncbi.nlm.nih.gov/entrez/query.fcgi?cmd=search&db=gene&term=ANKRD28) | | ankyrin repeat domain 28 | 0.0001963 | 268.16 | 430.77 | 0.62 |
| [231001_at](https://www.affymetrix.com/LinkServlet?probeset=231001_at) | [FIBIN](http://www.ncbi.nlm.nih.gov/entrez/query.fcgi?cmd=search&db=gene&term=FIBIN) | | fin bud initiation factor homolog (zebrafish) | 0.0001969 | 130.56 | 314.84 | 0.41 |
| [236828_at](https://www.affymetrix.com/LinkServlet?probeset=236828_at) | [NA](http://www.ncbi.nlm.nih.gov/entrez/query.fcgi?cmd=search&db=gene&term=NA) | | NA | 0.0001976 | 11.57 | 5.07 | 2.28 |
| [207336_at](https://www.affymetrix.com/LinkServlet?probeset=207336_at) | [SOX5](http://www.ncbi.nlm.nih.gov/entrez/query.fcgi?cmd=search&db=gene&term=SOX5) | | SRY (sex determining region Y)-box 5 | 0.000198 | 24.69 | 54.01 | 0.46 |
| [52940_at](https://www.affymetrix.com/LinkServlet?probeset=52940_at) | [SIGIRR](http://www.ncbi.nlm.nih.gov/entrez/query.fcgi?cmd=search&db=gene&term=SIGIRR) | | single immunoglobulin and toll-interleukin 1 receptor (TIR) domain | 0.0002033 | 134.17 | 64.11 | 2.09 |
| [227821_at](https://www.affymetrix.com/LinkServlet?probeset=227821_at) | [LGI4](http://www.ncbi.nlm.nih.gov/entrez/query.fcgi?cmd=search&db=gene&term=LGI4) | | leucine-rich repeat LGI family, member 4 | 0.0002042 | 104.34 | 33.77 | 3.09 |
| [218454_at](https://www.affymetrix.com/LinkServlet?probeset=218454_at) | [PLBD1](http://www.ncbi.nlm.nih.gov/entrez/query.fcgi?cmd=search&db=gene&term=PLBD1) | | phospholipase B domain containing 1 | 0.0002065 | 143.19 | 57.53 | 2.49 |
| [240138_at](https://www.affymetrix.com/LinkServlet?probeset=240138_at) | [NA](http://www.ncbi.nlm.nih.gov/entrez/query.fcgi?cmd=search&db=gene&term=NA) | | NA | 0.000207 | 77.76 | 317.34 | 0.25 |
| [205902_at](https://www.affymetrix.com/LinkServlet?probeset=205902_at) | [KCNN3](http://www.ncbi.nlm.nih.gov/entrez/query.fcgi?cmd=search&db=gene&term=KCNN3) | | potassium intermediate/small conductance calcium-activated channel | 0.0002071 | 173.85 | 699.93 | 0.25 |
| [1558170_at](https://www.affymetrix.com/LinkServlet?probeset=1558170_at) | [NA](http://www.ncbi.nlm.nih.gov/entrez/query.fcgi?cmd=search&db=gene&term=NA) | | NA | 0.0002074 | 71.32 | 372.75 | 0.19 |
| [227297_at](https://www.affymetrix.com/LinkServlet?probeset=227297_at) | [ITGA9](http://www.ncbi.nlm.nih.gov/entrez/query.fcgi?cmd=search&db=gene&term=ITGA9) | | integrin, alpha 9 | 0.0002097 | 85.45 | 194.98 | 0.44 |
